# Integrative and interpretable machine learning framework for early non-invasive detection of clinically significant liver fibrosis

**DOI:** 10.3389/fmed.2026.1736295

**Published:** 2026-06-23

**Authors:** Dong Cao, Junjie Wang, Chenxi Hou, Jingyu Zeng, Baiyue Tian, Yuan Liu, Xing Luo, Jiaxin Tian, Mingbo Zhou, Pan Li, Huilong Fang, Ze Liu, Zheng Gong

**Affiliations:** 1 School of Basic Medical Sciences, Xiangnan University, Chenzhou, China; 2 School of Pharmaceutical Sciences, Xiangnan University, Chenzhou, China; 3 School of Nursing, Xiangnan University, Chenzhou, China

**Keywords:** aspartate aminotransferase, Body Mass Index, liver fibrosis, machine learning, NHANES, predictive model, SHAP values, waist circumference

## Abstract

**Background:**

Liver fibrosis is a critical predictor of adverse liver-related outcomes, yet its early detection remains challenging. This research aimed to construct a predictive model for the early detection of clinically significant liver fibrosis, and identify its key risk factors, using clinical data from the NHANES 2017–2020 cycles.

**Methods:**

We analyzed a cohort of 6,164 participants, classifying them into a clinically significant liver fibrosis group and control group based on liver stiffness measurements. Data were preprocessed, with imputation for missing values and normalization of numerical variables. We employed recursive feature elimination and benchmarked 29 machine learning models, ultimately selecting the Gamboost model due to its excellent performance. The model's robustness was validated with an external dataset of 684 samples.

**Results:**

Key predictors included body mass index (BMI), aspartate aminotransferase (AST), and waist circumference (WC), among others. In the training cohort, the model reached an AUC of 0.824 (95% CI: 0.798–0.849), while in the testing cohort it yielded 0.872 (95% CI: 0.838–0.905), maintaining robust performance with an AUC of 0.848 (95% CI: 0.798–0.898) during external validation. Furthermore, SHAP and PDP analyses revealed that elevated AST and WC synergistically increased the predicted risk of fibrosis.

**Conclusion:**

By utilizing routinely accessible clinical data, our interpretable machine learning model offers a highly practical tool for the early identification of clinically significant liver fibrosis. This non-invasive approach can be readily implemented in primary care, facilitating timely interventions and supporting precision medicine initiatives.

## 1 Introduction

Liver fibrosis is a progressive pathological condition that not only leads to severe hepatic complications but also contributes to global mortality. It represents a critical transition between chronic hepatic illness and the development of cirrhosis or primary liver cancer. Without intervention, liver fibrosis can progress to cirrhosis, hepatocellular carcinoma, or even end-stage liver failure (1). While lifestyle interventions remain the cornerstone of management, and recent breakthrough targeted therapies (e.g., resmetirom) have only just received conditional approval for specific etiologies like metabolic dysfunction-associated steatohepatitis (MASH), broadly applicable and highly effective anti-fibrotic medications remain a critical unmet clinical need (2). Therefore, early diagnosis, careful monitoring and timely intervention are critical (3). However, the current diagnostic methods face challenges in terms of limited precision for early detection and the reliance on invasive procedures which reduces their feasibility for routine examinations. This limitation hinders timely intervention and treatment (4). Given the potential progression of severe fibrosis to cirrhosis and liver cancer, timely assessment of fibrosis levels and accurate diagnosis of advanced fibrosis are imperative.

Contemporary research point out the importance of identifying and quantifying risk variables contributing to liver fibrosis. For example, the fibrosis stage has shown prognostic value in metabolic dysfunction-associated steatotic liver disease (MASLD) patients (5). Dynamic evaluations of liver fibrosis by indices like FIB-4 can predict the risk of hepatocellular carcinoma in individuals with chronic hepatitis C and sustained virologic response (6). Metabolic and genetic factors have been shown to strongly influence the progression and severity of alcohol-induced liver fibrosis (7). Obesity is a significant risk factor, because excessive adipose tissue promotes systemic inflammation and insulin resistance, which are key drivers of fibrogenesis. Insulin resistance, a key feature of metabolic syndrome, contributes critically to stellate cell activation and collagen deposition in the liver (8). Genetic predispositions have also been shown to modulate the risk and progression of fibrosis, such as polymorphisms in genes related to lipid metabolism and inflammation (9). For example, in MASLD patients the PNPLA3 gene variations associated with the risk of fibrosis (10). Environmental factors, such as chronic alcohol consumption and hepatotoxic chemicals exposure, further accelerate liver damage and fibrosis (11). Additionally, chronic infections with hepatitis B and C viruses are key contributors to the development of fibrosis; the persistent inflammatory response to viral antigens leads to ongoing liver injury and scar formation (12). Therefore, research on the risk factors is crucial for risk stratification and early intervention strategies.

The complex interplay among these factors emphasizes the multifactorial pathogenesis of liver fibrosis, highlighting the critical need for integrated risk assessment frameworks. There is an urgent need for diagnostic tools that are accessible, affordable, and reliable. They can be used effectively in primary care environments without extensive training or sophisticated equipment. Such advancements would enable quick and widespread screening, which is particularly crucial for early intervention in high-risk populations.

The traditional diagnosis of liver fibrosis relied on liver biopsy. However, this technique has several drawbacks: it is invasive, expensive, and prone to sampling errors and complications (13). Non-invasive approaches, including transient elastography and magnetic resonance elastography, have been introduced as safer and less painful alternatives (14, 15). These techniques also face limitations, including high costs, limited availability, and the need for specialized equipment and trained personnel. While transient elastography boasts high sensitivity and specificity, its results must be interpreted with caution in the presence of liver inflammation, which leads to false-positive measurements (16). The complex pathophysiology of liver fibrosis, which involves various factors such as iron overload, further complicates the diagnostic challenge (17). Furthermore, obesity negatively affects transient elastography, leading to increased failure rates and decreased reliability in patients with a body mass index (BMI) >30 kg/m^2^ (18). Magnetic resonance elastography (MRE) remains accurate and useful for obese patients and those with ascites, but hepatic iron deposition can interfere with its measurements (19). Because it is expensive and time-consuming, MRE is not routinely used in clinical practice (20). Serum biomarkers such as MMP7 and CHI3L1 have also shown promise in non-invasive diagnosis, but their sensitivity and specificity can vary in different stages of fibrosis (21, 22). In addition to the above biomarkers, simple serological scores such as FIB-4 (Fibrosis-4 index) and APRI (AST-to-Platelet Ratio Index) have become first-line, non-invasive tools for liver fibrosis assessment in routine clinical practice (23–25). Their advantages include low cost, wide availability, and reliance on commonly measured laboratory parameters (AST, ALT, platelets), making them particularly suitable for primary care and large-scale screening. However, both indices have inherent limitations. A substantial proportion of patients fall into the indeterminate “gray zone”, where the scores are neither low enough to exclude nor high enough to rule in significant fibrosis, necessitating further confirmatory testing. Moreover, the diagnostic accuracy of FIB-4 and APRI declines in elderly populations, in patients with low platelet counts from non-hepatic causes, or in those with concurrent muscle wasting that alters transaminase levels. These shortcomings highlight the need for more robust, non-linear, and adaptable predictive tools that can capture complex interactions among risk factors without being constrained by fixed mathematical formulas. Therefore, we developed a machine learning (Gamboost) model to overcome the limitations of conventional scores, offering improved accuracy and interpretability for early detection of clinically significant liver fibrosis.

Our study aims not only to develop a diagnostic model for clinically significant liver fibrosis, but also to identify significant risk factors associated with fibrosis through the modeling process. Utilizing clinical inquiry and blood test data, we focus primarily on demographic and laboratory parameters. A key advantage of this model is its seamless clinical deployability—it relies on routine data, bypassing the need for advanced diagnostic imaging or specialized training. It can be easily applied in most primary healthcare environments, facilitating the quick screening of at-risk populations and support to the prevention and control of liver fibrosis progression. Using a dataset collected in NHANES 2017–2020, excluding individuals under 18 years old, we analyzed liver ultrasound data from the NHANES Liver Ultrasound Elastography Subsample (LUXSMED) project to determine fibrosis levels. The samples were categorized into clinically significant liver fibrosis cases (liver stiffness over 8 kPa) and controls. We compiled demographic and laboratory test parameters as independent variables for the analysis.

Feature selection was conducted using seven machine learning (ML) methods under the mlr3 framework: Extreme Gradient Boosting (XGBoost-RFE), Support Vector Machine (SVM-RFE), Gradient Boosting Machine (GBM-RFE), and other ensemble or tree-based approaches such as Rpart-RFE, LightGBM, Ranger-RFE, and CatBoost-RFE. These methods were employed to identify significant predictors of clinically significant liver fibrosis. A Venn diagram was utilized to determine the optimal variables. Additionally, we benchmarked 29 ML methods to identify the best-performing model, followed by grid tuning to further refine the selected model. The performance of the model was assessed by Receiver Operating Characteristic analysis and calculating the corresponding area under the curve (AUC). Model interpretation was conducted through Permutation Feature Importance (PFI), Accumulated Local Effects (ALE), Partial Dependence Plot (PDP), and SHapley Additive exPlanations (SHAP) methods.

In summary, our study endeavors to generate a non-invasive, highly accessible diagnostic model for clinically significant liver fibrosis while simultaneously identifying significant risk factors associated with the condition. Leveraging NHANES data and state-of-the-art ML techniques, this approach promises to enhance early detection and intervention strategies, thereby mitigating the progression of liver fibrosis to more severe liver conditions.

## 2 Methods

### 2.1 Study cohort (training and testing dataset)

Data were obtained from the National Health and Nutrition Examination Survey (NHANES), a nationally representative, ongoing, cross-sectional program designed to evaluate the health and nutrition in the US population. This survey is administered by the Centers for Disease Control and Prevention (CDC) and was approved by the National Center for Health Statistics (NCHS). All participants provided written informed consent. Because the present study involves secondary analysis of completely de-identified public data, no additional institutional review board (IRB) approval was required.

Data from the 2017–2020 NHANES survey cycles were used, integrating demographic, dietary, examination, laboratory, and questionnaire datasets. Participants were categorized into clinically significant liver fibrosis and control groups based on the median stiffness (E) measured in kilopascals (kPa) using Liver Ultrasound Transient Elastography (LUXSMED variable). Samples with missing LUXSMED data were excluded, and those with median stiffness >8 kPa were classified as clinically significant liver fibrosis cases (26–28). Participants under 18 years old were also excluded, and variables related to Liver Ultrasound Transient Elastography and Oral Health were removed. Data labeled as missing, “don't know,” or “refused” were coded as NA. Variables exceeding 20% missingness were discarded, and samples with over 10% missing data were removed. The final dataset included 6,164 samples.

### 2.2 External validation cohort (outtest dataset)

An independent external validation cohort was established using clinical data collected from the Affiliated Hospital of Xiangnan University (Hunan, China) between January 2018 and December 2023 to evaluate the generalizability of the developed model. All participants were adults (≥18 years old) who underwent comprehensive physical and biochemical examinations as part of routine health checkups or liver disease evaluations. The inclusion criteria were: (1) availability of complete demographic, anthropometric, and biochemical measurements corresponding to variables used in the NHANES-based model; and (2) clear clinical classification of liver fibrosis stage based on transient elastography or imaging-supported clinical diagnosis. Exclusion criteria included: (1) acute or chronic infectious liver diseases other than metabolic or cryptogenic etiologies; (2) malignancies or autoimmune disorders; (3) missing essential biochemical data; and (4) excessive alcohol consumption (>30 g/day for men and >20 g/day for women). After applying these criteria, a total of 684 participants were included in the final external validation cohort. The detailed step-by-step patient enrollment cascade is provided in Supplementary file 1. Additionally, the contemporary etiological distribution of the clinically significant liver fibrosis cases is illustrated in Supplementary file 2. The baseline clinical characteristics of the final cohort are summarized in Table 1. Data collection and analysis followed institutional ethical standards and the Declaration of Helsinki, with approval from the Ethics Committee of the Affiliated Hospital of Xiangnan University. Patient informed consent was waived owing strictly to the retrospective, observational nature of the electronic medical record analysis.

**Table 1:** Descriptive statistics of the independent external validation cohort used for model generalizability assessment.

Characteristic	Overall *N* = 684^a^	Control *N* = 606^a^	Case *N* = 78^a^	*p*-value^b^
Gender				
Male	346 (51%)	300 (50%)	46 (59%)	0.12
Female	338 (49%)	306 (50%)	32 (41%)	
Standing height (cm)	167.41 (9.84)	167.16 (9.73)	169.35 (10.54)	0.084
Weight (kg)	83.40 (22.31)	80.69 (19.61)	104.53 (29.79)	< 0.001
Waist circumference (cm)	100.29 (16.96)	98.14 (15.25)	117.20 (20.05)	< 0.001
BMI (kg/m^2^)	29.71 (7.24)	28.86 (6.46)	36.30 (9.32)	< 0.001
AST (U/L)	21.63 (13.05)	20.62 (7.75)	29.58 (31.24)	0.018
Age	49.80 (17.34)	49.17 (17.52)	54.77 (15.04)	0.007
GGT (U/L)	28.73 (37.80)	25.04 (23.58)	57.36 (85.82)	< 0.001
Urine albumin (ug/ml)	48.31 (222.82)	45.09 (219.62)	73.11 (246.19)	0.011
Platelet count (1,000 cells/uL)	245.27 (65.81)	247.75 (65.37)	226.01 (66.41)	0.029
CPK (IU/L)	172.06 (297.37)	173.19 (311.98)	163.17 (136.11)	0.6
ALT (U/L)	21.53 (15.27)	20.34 (11.89)	30.71 (29.32)	< 0.001
Albumin (g/L)	40.70 (3.28)	40.85 (3.17)	39.54 (3.86)	0.002
Total bilirubin (umol/L)	7.70 (4.64)	7.44 (4.29)	9.67 (6.48)	0.015
ALP (IU/L)	76.64 (24.63)	75.06 (21.99)	89.12 (37.66)	0.002
Total protein (g/L)	71.28 (4.52)	71.21 (4.45)	71.80 (5.01)	0.7

^a^n (%); mean (SD).

^b^Pearson's Chi-squared test; Wilcoxon rank sum test.

### 2.3 Data pre-processing

Variables with over 20% missing data were excluded. Within the mlr3 framework, the functions “imputehist” and “regr.kknn” were used to impute remaining missing data. Skewed variables underwent log2 transformation, and numerical variables were standardized via *Z*-score scaling using the *scale* function. Class imbalance was addressed by testing undersampling, oversampling, and SMOTE, with the optimal strategy selected based on the highest F-beta score. Multicollinearity was examined by correlation analysis and VIF, with features showing VIF>10 excluded.

### 2.4 Machine learning pipeline and model selection

**Figure 1:**
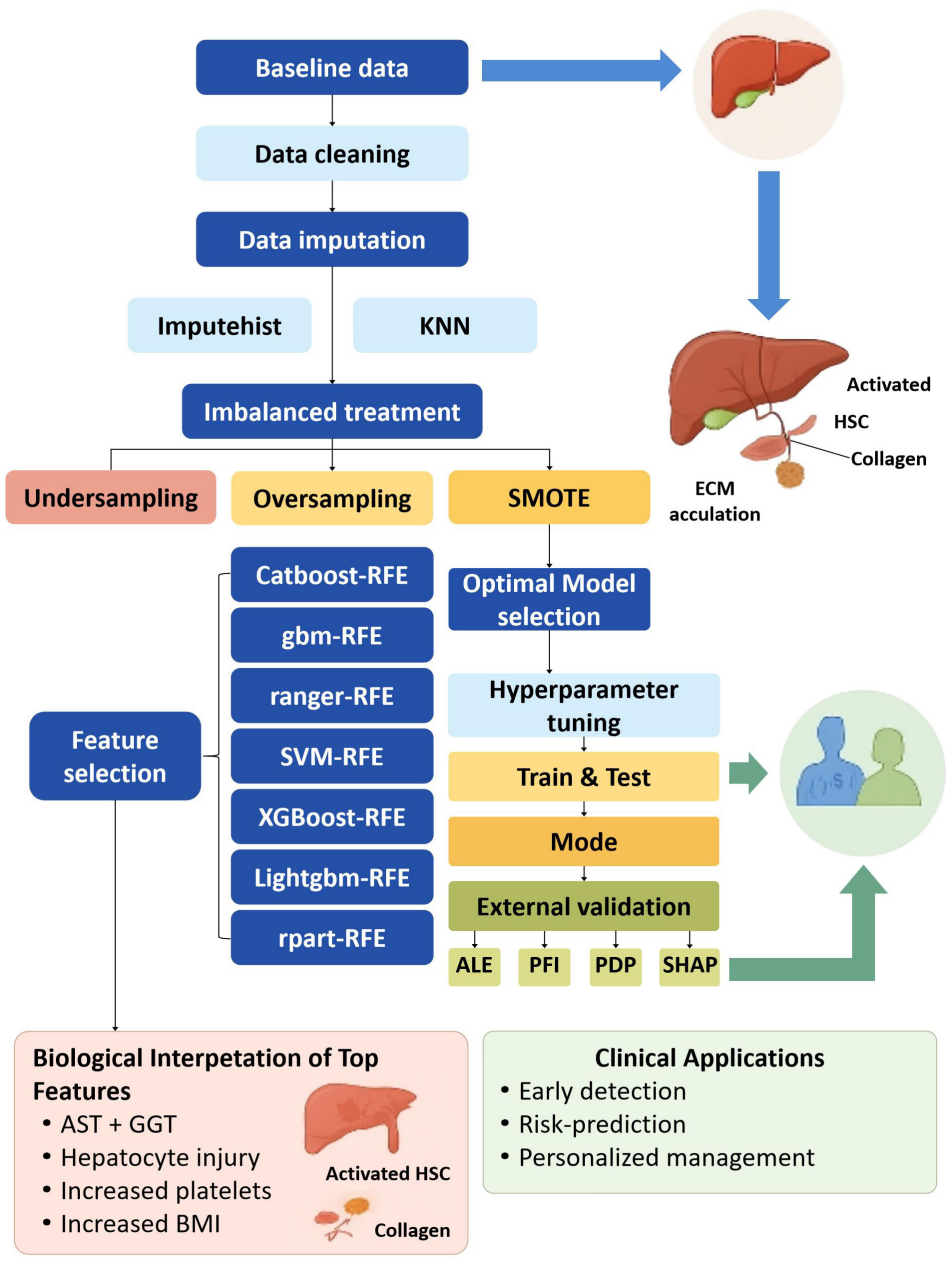
Study workflow diagram. The diagram outlines the methodological framework used in this study to develop a predictive model for clinically significant liver fibrosis. The process begins with baseline data collection and data cleaning, followed by data imputation using “imputehist” and “regr.kknn” functions to handle missing values. The imbalanced data were addressed through undersampling, oversampling, and SMOTE techniques, with undersampling selected for optimal results. Feature selection was conducted using several recursive feature elimination (RFE) methods, including CatBoost-RFE, gbm-RFE, ranger-RFE, SVM-RFE, XGBoost-RFE, LightGBM-RFE, and rpart-RFE. The optimal model was chosen based on performance metrics, and hyperparameter tuning was performed. The model was trained and externally validated, with interpretive techniques such as Accumulated Local Effects (ALE), Permutation Feature Importance (PFI), Partial Dependence Plot (PDP), and SHapley Additive exPlanations (SHAP) used to understand the model's behavior.

Feature selection and model construction were guided by the workflow outlined in Figure 1. To prevent overfitting and eliminate methodological redundancy, a unified, overarching five-fold cross-validation strategy was systematically applied across all phases of model development. This included recursive feature elimination (RFE) for selecting the most robust predictors and internal performance validation. A comprehensive suite of 29 machine learning classification models—encompassing linear algorithms, tree-based ensembles, and support vector machines—was benchmarked using the mlr3 framework.

The Gradient Boosting with Component-wise Linear Models (Gamboost) algorithm was ultimately selected as the optimal predictive framework. Hyperparameter tuning for Gamboost was conducted via grid search (parameters explored: *dfbase* = 2–20, *mstop* = 1–1,000, and *nu* = 0.01–1), optimizing for the highest Area Under the Curve (AUC). The clinical rationale for prioritizing Gamboost was twofold: it demonstrated exceptional discriminative performance, and its component-wise generalized additive structure provides intrinsic variable selection while allowing for the transparent modeling of non-linear physiological relationships, making it highly suited for clinical decision-making.

### 2.5 Approaches for model interpretation

Model interpretability was assessed using Shapley Additive Explanation (SHAP) values and Partial Dependence Plots (PDP). SHAP values, derived with the iml R package and visualized using the SHAP Python library, elucidated the contribution of each feature. PDPs, generated using the PDPbox Python library, depicted interactions among predictors. Additional interpretative tools, such as ALE plots and PFI scores, were used to understanding how the model functions and to evaluate the importance of each individual feature.

### 2.6 Model evaluation and statistical analysis

Multivariable classification models were evaluated using ROC curves and AUC metrics. The selected optimal model was further refined through grid search and five-fold cross-validation to optimize tuning parameters and enhance model discrimination. Once the optimal model was established, its parameters were applied to both the test group and the external validation group to evaluate performance consistency across different datasets. To ensure rigorous evaluation and statistical transparency, all key performance metrics—including AUC, accuracy, sensitivity, and specificity—were reported alongside their corresponding 95% confidence intervals (CIs). The 95% CIs for the AUCs were computed using DeLong's method, whereas the 95% CIs for confusion matrix-derived metrics were calculated utilizing the Clopper-Pearson exact binomial method. Continuous data were presented as means ± standard deviations (SDs) when the distribution was approximately normal, and as medians with interquartile ranges when the data were skewed or not normally distributed. Categorical data were presented as numbers and frequency (%). Continuous variables were compared using one-way ANOVA for normally distributed data or Mann–Whitney U test otherwise. The categorical variables were compared using chi-square analysis, and statistical significance was defined as a two-sided *p*-value under 0.05. All statistical analyses were performed using R software (version 4.3.1).

### 2.7 Calculation of non-invasive fibrosis scores and model comparison

To benchmark the predictive performance of the proposed Gamboost model against established clinical indices, three traditional non-invasive liver fibrosis scores were calculated for each participant: the Fibrosis-4 (FIB-4) index, the AST to Platelet Ratio Index (APRI), and the NAFLD Fibrosis Score (NFS) (29, 30). The APRI was calculated using an upper limit of normal (ULN) for AST set at 40 U/L. For the NFS calculation, impaired fasting glucose (IFG) or diabetes was defined as either a self-reported history of diabetes via questionnaire or a glycated hemoglobin (HbA1c) level of ≥5.7%.

ROC curves were constructed to evaluate the discriminatory ability of the models. The AUC for the Gamboost model was statistically compared in a head-to-head manner against those of the FIB-4, APRI, and NFS using DeLong's test. Two-sided *p*-values < 0.05 were considered to indicate a statistically significant difference in model performance. These comparative analyses and ROC visualizations were performed using the pROC package in R.

### 2.8 Model calibration, recalibration, and clinical utility assessment

To rigorously evaluate the agreement between the predicted probabilities and the actual observed frequencies of liver fibrosis, model calibration was assessed using the Brier score (measuring overall prediction error) and the Hosmer-Lemeshow goodness-of-fit test (grouping data into 10 deciles).

Because the Gamboost model was derived from a 1:1 undersampled training cohort to prioritize sensitivity—implicitly assuming a 50% prior probability of disease—applying these raw probabilities directly to real-world datasets would mathematically lead to systematic overestimation of risk. To address this, a standard Bayesian prior-correction (recalibration) was systematically applied. The predicted odds were mathematically adjusted to align with the true natural prevalence of the NHANES testing set and the observed prevalence of the external hospital validation cohort. Calibration curves were then plotted using these recalibrated probabilities, with 95% confidence intervals for the observed proportions calculated via the Clopper-Pearson exact binomial test.

Furthermore, the practical clinical value of the model was evaluated using Decision Curve Analysis (DCA). DCA calculates the net clinical benefit of utilizing the model to guide non-invasive screening decisions across a continuum of threshold probabilities, benchmarking it against default strategies of assuming all patients have the disease (“treat-all”) or no patients have the disease (“treat-none”). To prevent the artificial inflation of false-positive penalties, the recalibrated probabilities were utilized for the DCA. These advanced statistical analyses and visualizations were conducted using custom scripts and the *ResourceSelection* package in R.

## 3 Results

### 3.1 Study population characteristics

Following data cleaning, our study population comprised 6,164 participants from the NHANES dataset, with 448 variables included (Supplementary files 3–9). Statistically significant variation was detected between the controls and the clinically significant liver fibrosis cases in gender, birthplace, education level (AGE >20), BMI, and age (*p* < 0.01). Additionally, notable differences were identified between the control group (*n* = 5,458) and the clinically significant liver fibrosis group (*n* = 706) in laboratory indices such as alkaline phosphatase, gamma-glutamyl transferase (GGT), urinary albumin, aspartate aminotransferase (AST), and alanine aminotransferase (*p* < 0.01).

### 3.2 Addressing class imbalance

**Figure 2:**
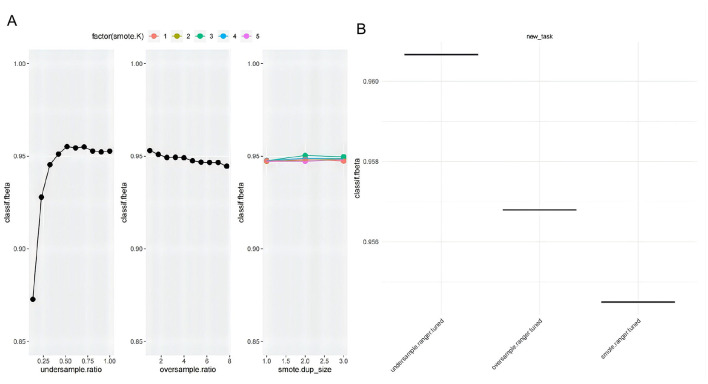
Sensitivity analysis of strategies for addressing class imbalance. (**A)** Optimization of resampling hyperparameters. The performance of three distinct resampling techniques (undersampling, oversampling, and SMOTE) was evaluated across varying parameter ratios. To align with the clinical priority of minimizing false negatives in liver fibrosis screening, the F-beta score was utilized as the primary optimization metric. The panels illustrate the impact of the undersample ratio **(left)**, oversample ratio **(middle)**, and SMOTE duplication size **(right)** on the F-beta score. **(B)** Comparative performance of the optimized resampling methods. The chart compares the maximum F-beta scores achieved by each strategy. Undersampling yielded the most favorable outcome, achieving the highest F-beta score (0.961) when coupled with the ranger classifier. Consequently, an undersampling approach (at a 1:1 ratio) was selected as the optimal strategy to construct the balanced training cohort for downstream predictive modeling.

We observed a significant class imbalance in our dataset, with clinically significant liver fibrosis cases being markedly outnumbered by controls, a factor that can adversely affect the predictive performance of machine learning algorithms. To systematically address this, we conducted a sensitivity analysis comparing three distinct class-imbalance handling strategies: undersampling, oversampling, and the Synthetic Minority Over-sampling Technique (SMOTE; Figure 2A).

The optimization process was executed within the mlr3 ecosystem using a 3-fold cross-validation approach. Crucially, model optimization and strategy selection were guided by the F-beta score rather than overall accuracy. In the clinical context of primary liver fibrosis screening, the consequence of a false negative (failing to identify a patient with progressive fibrosis) substantially outweighs the cost of a false positive (which typically only triggers secondary, non-invasive confirmatory testing). Because the F-beta metric heavily penalizes false negatives, it was prioritized to ensure maximum sensitivity for detecting the minority positive class.

As illustrated in Figure 2B, the sensitivity analysis demonstrated that undersampling objectively outperformed both oversampling and SMOTE, achieving the highest F-beta score (0.961). Consequently, undersampling was employed to balance the dataset, yielding an equalized training cohort of 1,412 samples (706 cases and 706 controls). Although undersampling inherently involves discarding a portion of the majority class, this retained sample volume remained highly robust for training the Gamboost framework to learn stable decision boundaries without severe overfitting. Furthermore, the model's subsequent high performance in the independent, naturally imbalanced external validation cohort (AUC = 0.848) confirms that this undersampling strategy effectively preserved model stability and generalizability without detrimental information loss.

### 3.3 Feature selection and identification of risk factors

**Figure 3:**
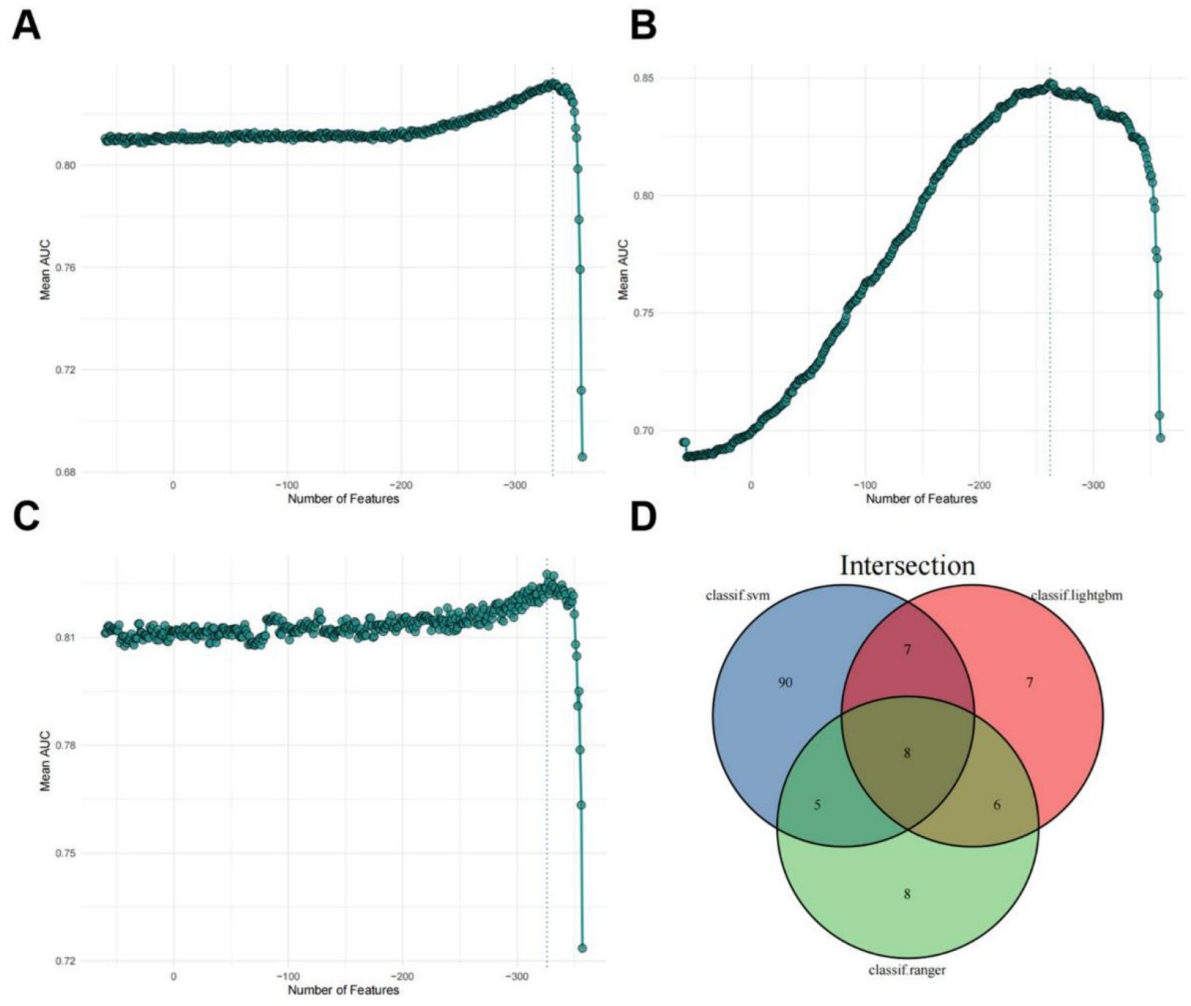
Feature selection using RFE and identification of key variables. This figure presents the results of Recursive Feature Elimination (RFE) conducted using different machine learning methods to identify key features associated with clinically significant liver fibrosis. **(A–C)** display the RFE analysis results for the Ranger-RFE, SVM-RFE, and LightGBM-RFE models, respectively, which achieved the top three highest AUC values. The x-axis represents the number of features selected, while the y-axis shows the mean AUC score. In **(D)**, a Venn diagram illustrates the intersection of the top features identified by LightGBM-RFE, Ranger-RFE, and SVM-RFE, revealing eight critical variables common to all three models. These variables were selected for further analysis in the predictive model.

**Figure 4:**
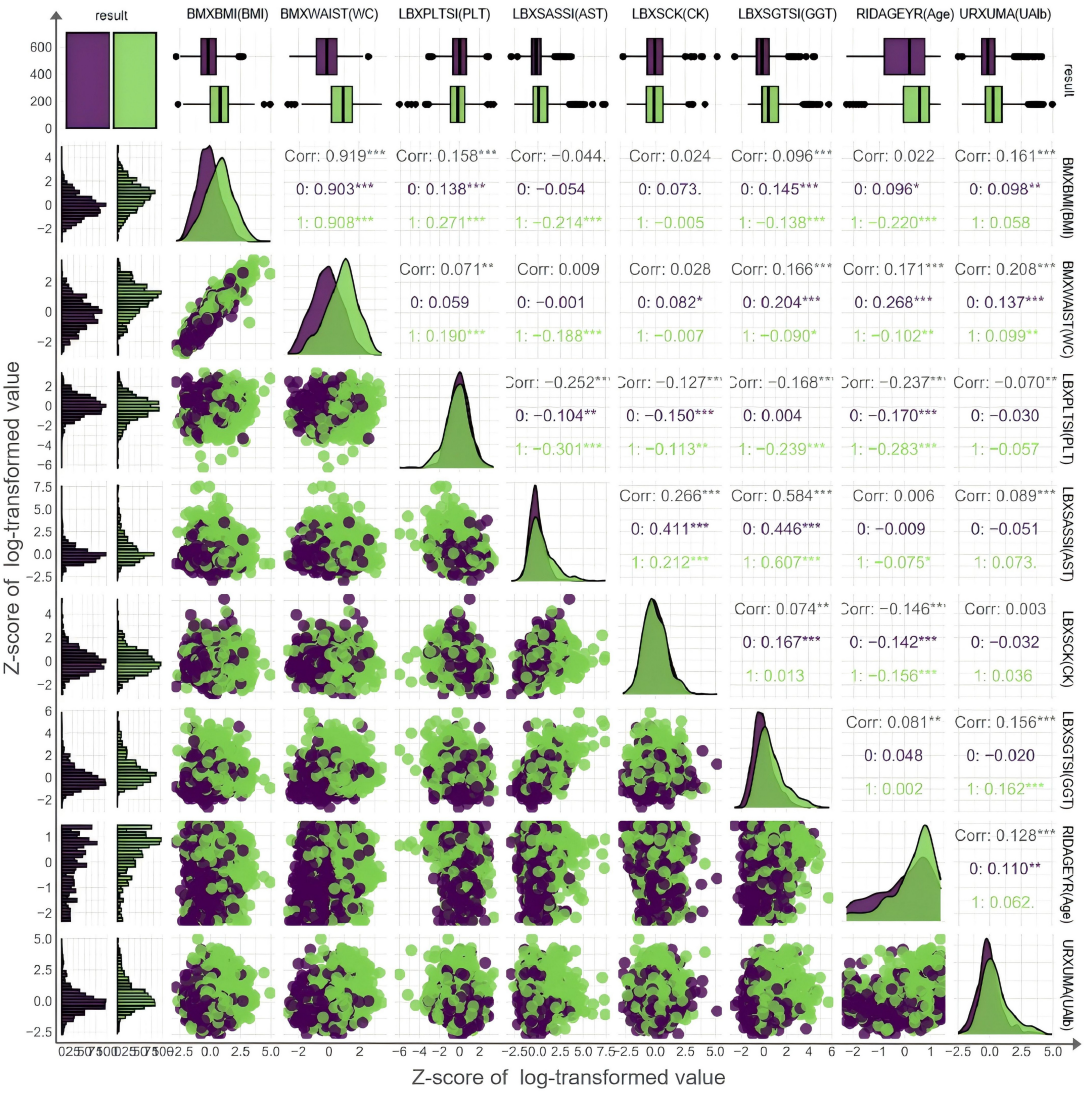
Correlation analysis and distribution of key variables across groups. This figure illustrates the correlation analysis conducted on the eight key variables identified as predictors of clinically significant liver fibrosis. The heatmap and scatter plots compare the correlations among these variables in the overall dataset, the control group, and the clinically significant liver fibrosis group. Histograms and boxplots on the diagonal show the distribution of each variable within the different groups. The strength and direction of the correlations are annotated within the scatter plots, revealing distinct patterns of association between variables across different patient groups. This comprehensive analysis highlights the relationships among the selected features and their differing behaviors in patients with and without clinically significant liver fibrosis. (0: control group. 1: clinically significant liver fibrosis group). All continuous variables were log-transformed and standardized (Z-score normalization) prior to analysis; thus, axes represent Z-scores of log-transformed values rather than original units.

Recursive feature elimination (RFE) was used for feature selection. A total of seven machine learning feature selection methods—CatBoost-RFE, Ranger-RFE, SVM-RFE, GBM-RFE, LightGBM-RFE, XGBoost-RFE, and Rpart-RFE—were used to screen important variables from the comprehensive liver fibrosis characteristics (Supplementary file 10). Following initial variable screening, we used univariate ROC curves and AUC values to assess the predictive capability of different variable combinations (Supplementary file 11). The univariate ROC analysis revealed the feature count corresponding to the highest AUC value. Among them, Ranger-RFE, LightGBM-RFE, and SVM-RFE demonstrated superior performance (Figures 3A–C). The Venn diagram (Figure 3D) illustrates the overlapping key features determined by Ranger-RFE, LightGBM-RFE, and SVM-RFE, identifying eight critical variables: body mass index (BMXBMI), waist circumference (BMXWAIST), platelet count (LBXPLTSI), aspartate aminotransferase (LBXSASSI), creatine kinase (LBXSCK), gamma-glutamyl transferase (LBXSGTSI), age (RIDAGEYR), and urinary albumin (URXUMA). Full definitions and corresponding names for these variable abbreviations are detailed in Table 2. We conducted correlation analysis among all candidate features as well as within the ultimately chosen variables (Figure 4). Comparative analysis between the control group and the clinically significant fibrosis group demonstrated significant predictive value of these eight variables (Figure 4). Furthermore, VIF analysis was applied to assess multicollinearity, showing no significant multicollinearity among the selected variables (all VIF < 10; Table 3).

**Table 2:** NHANES codes and their corresponding clinical terms (full name and abbreviation).

NHANES code	Clinical term (full name and abbreviation)	Abbreviation
BMXWAIST	Waist circumference	WC
LBXSASSI	Aspartate aminotransferase	AST
BMXBMI	Body mass index	BMI
LBXPLTSI	Platelet count	PLT
LBXSCK	Creatine kinase	CK
LBXSGTSI	Gamma-glutamyl transferase	GGT
RIDAGEYR	Age	Age
URXUMA	Urine albumin	UAIb

**Table 3:** Variance inflation factor (VIF) coefficient is used to calculate the variance inflation factor (VIF) index (all VIF<10) to diagnose its multicollinearity.

Index	BMXBMI	BMXWAIST	LBXPLTSI	LBXSASSI	LBXSCK	LBXSGTSI	RIDAGEYR	URXUMA
VIF	7.6012	7.8963	1.1899	1.7128	1.1337	1.6220	1.2719	1.0737

### 3.4 Benchmarking and choice of models

**Figure 5:**
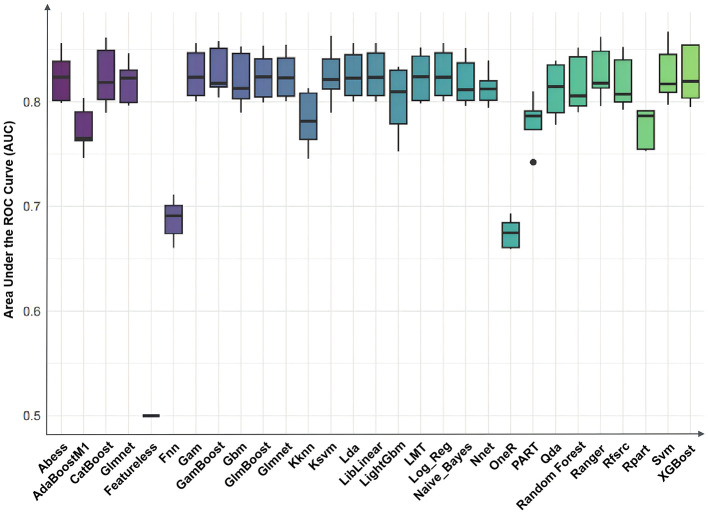
Benchmarking machine learning models using key variables. This figure presents the benchmarking of 29 different machine learning (ML) classification models based on the eight key variables identified in this study. The boxplots display the distribution of AUC values across different models, with the y-axis representing the classifier AUC. The Gamboost model outperformed the others, demonstrating the highest median AUC value among all models tested. Consequently, the Gamboost model was selected for further analysis and refinement in predicting clinically significant liver fibrosis, emphasizing its superior discriminative ability and robustness. AUC is a dimensionless metric ranging from 0 to 1; therefore, no physical units are shown on the y-axis.

We compared 29 different ML classification models based on the eight key variables identified in this study, including abess, AdaBoost, CatBoost, and glmnet. Internal cross-validation was conducted (Supplementary file 12). Table 4 presents the AUC, classification error (CE), sensitivity, specificity, FNR, and FPR for each of the 29 models, and a box plot was used to more intuitively display the AUC values of the models (Figure 5). While several models demonstrated comparable predictive capabilities, the Gamboost (Gradient Boosting with Component-wise Linear Models) algorithm yielded the top median AUC (AUC = 0.829).

**Table 4:** All constructed predictive models were developed without the use of data enhancement techniques.

No.	Learner_id	AUC	CE	Sensitivity	Specificity	FNR	FPR
1	abess	0.8236	0.2472	0.7411	0.7653	0.2589	0.2347
2	AdaBoostM1	0.7736	0.2783	0.7378	0.7071	0.2622	0.2929
3	CatBoost	0.8241	0.2465	0.7505	0.7567	0.2495	0.2433
4	cv_glmnet	0.8189	0.2479	0.7316	0.7745	0.2684	0.2255
5	featureless	0.5000	0.5184	0.4000	0.6000	0.6000	0.4000
6	fnn	0.6875	0.3130	0.6848	0.6902	0.3152	0.3098
7	gam	0.8265	0.2436	0.7483	0.7651	0.2517	0.2349
8	gamboost	0.8291	0.2408	0.7695	0.7503	0.2305	0.2497
9	gbm	0.8208	0.2507	0.7580	0.7414	0.2420	0.2586
10	glmboost	0.8245	0.2458	0.7372	0.7723	0.2628	0.2277
11	glmnet	0.8250	0.2443	0.7399	0.7725	0.2601	0.2275
12	kknn	0.7824	0.2861	0.7076	0.7213	0.2924	0.2787
13	ksvm	0.8254	0.2394	0.7526	0.7694	0.2474	0.2306
14	lda	0.8260	0.2458	0.7442	0.7652	0.2558	0.2348
15	liblinear	0.8264	0.2450	0.7468	0.7637	0.2532	0.2363
16	LightGBM	0.8009	0.2741	0.7346	0.7185	0.2654	0.2815
17	LMT	0.8238	0.2500	0.7453	0.7546	0.2547	0.2454
18	log_reg	0.8265	0.2436	0.7483	0.7651	0.2517	0.2349
19	naive_bayes	0.8195	0.2535	0.7777	0.7164	0.2223	0.2836
20	nnet	0.8134	0.2578	0.7328	0.7525	0.2672	0.2475
21	OneR	0.6744	0.3265	0.7179	0.6310	0.2821	0.3690
22	PART	0.7806	0.2684	0.7181	0.7461	0.2819	0.2539
23	qda	0.8112	0.2635	0.7898	0.6851	0.2102	0.3149
24	randomForest	0.8172	0.2458	0.7485	0.7615	0.2515	0.2385
25	ranger.tuned	0.8275	0.2387	0.7482	0.7758	0.2518	0.2242
26	rfsrc	0.8183	0.2465	0.7396	0.7683	0.2604	0.2317
27	rpart.tuned	0.7754	0.2797	0.7055	0.7372	0.2945	0.2628
28	svm.tuned	0.8271	0.2380	0.7666	0.7576	0.2334	0.2424
29	xgboost.tuned	0.8254	0.2443	0.7580	0.7539	0.2420	0.2461

AUC, the area under the receiver operating curve; Ce, cross entropy; FNR, false negative rate; FPR, false positive rate.

Have a good sense of sensitivity, specificity.

Importantly, the final selection of the Gamboost model was not solely based on its marginal superiority in AUC. Rather, it was strategically chosen because it effectively balances high predictive performance with excellent clinical interpretability. Unlike traditional “black-box” models (e.g., deep neural networks or complex random forests), Gamboost explicitly models non-linear relationships using smooth functions—which perfectly aligns with the non-linear physiological impacts of variables such as age and BMI—and performs intrinsic variable selection. This makes the model inherently more transparent and uniquely suited for clinical environments, where understanding the physiological “why” behind a prediction is as critical as the prediction itself. Therefore, Gamboost was selected as the optimal framework for downstream classification analyses.

### 3.5 Model development and model performance evaluation

**Figure 6:**
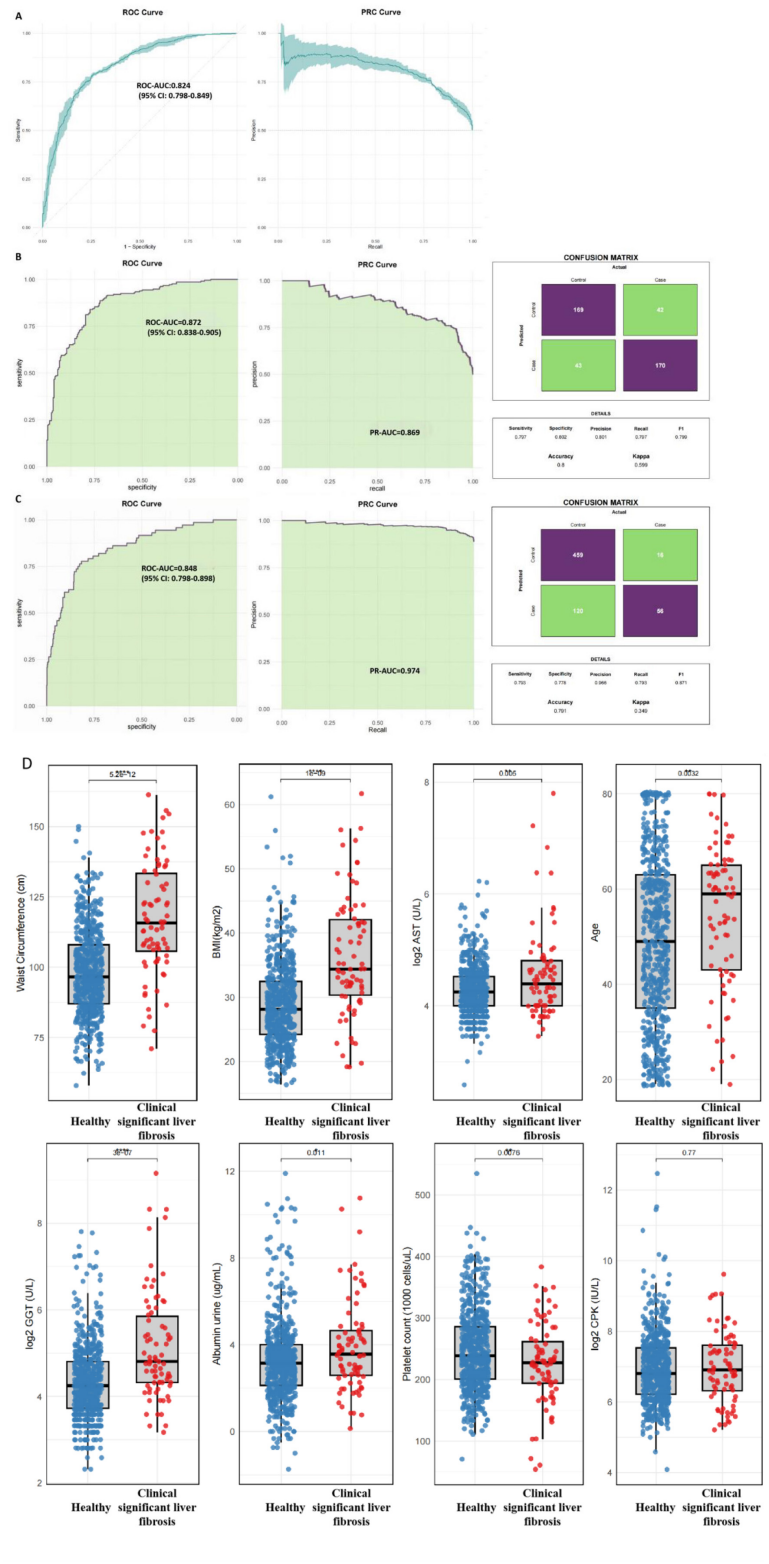
Gamboost model development, evaluation, and validation. This figure illustrates the process and performance of the Gamboost model developed for predicting clinically significant liver fibrosis. **(A)** shows the results from the training set after tuning the Gamboost model. The left graph displays the ROC curve, representing the model's sensitivity vs. specificity, while the right graph shows the Precision-Recall Curve (PRC), both indicating robust performance during model development. **(B)** demonstrates the model's performance on the test set. The left graph presents the ROC curve, the middle graph shows the PRC curve, and the right section displays the confusion matrix, summarizing the classification results. The confusion matrix reveals strong model accuracy and reliable sensitivity and specificity metrics. **(C)** evaluates the model using an external validation set. The left graph shows the ROC curve, the middle graph illustrates the PRC curve, and the right panel provides the confusion matrix. The model maintains high discriminative ability with consistent performance across different datasets, confirming its generalizability and robustness in identifying clinically significant liver fibrosis. **(D)** Baseline clinical and biochemical characteristics between healthy and liver fibrosis groups in the external validation cohort. Boxplots illustrate the distribution of key anthropometric and laboratory variables-including waist circumference, BMI, age, platelet count, urine albumin, and log2-transformed AST, GGT, and CPK-across study groups (blue = Healthy; red = Clinically significant liver fibrosis).

Participants were randomly partitioned designating 80% of cases for training and the remaining 20% for testing, and AUC values of 29 machine learning algorithms were computed on both subsets (Table 5). The training set was used to construct the model and fine-tune hyperparameters via internal cross-validation, whereas the testing set was strictly reserved for evaluating final model performance. We analyzed the ROC and PRC curves of the training, internal testing, and external validation datasets to assess the classification model's performance (Figure 6). The imbalance in the target variable was addressed through undersampling. Optimal hyperparameters were determined via grid search with five-fold cross-validation. For the Gamboost classifier, we set the tuning range to dfbase = 2–20, mstop = 1–1,000, and nu = 0.01–1. We explored 1,000 parameter combinations, using cross-validation within the five folds for model development, and based on the cross-validation AUC, we identified the best-performing parameter set. The best parameters were dfbase = 3, mstop = 112, and nu = 0.45, with the model achieving an median AUC of 0.824 (95% CI: 0.798–0.849) on the training set (Figure 6A). We utilized ROC and confusion matrix on testing data set and external validation dataset to evaluate model performance. The final optimized Gamboost model yielded an AUC of 0.872 (95% CI: 0.838–0.905) on the internal testing dataset (Figure 6B). The confusion matrix reflected the accuracy of traditional ML algorithms, with correctly classified data along the diagonal. Misclassifications were reflected in off-diagonal areas. The confusion matrix revealed a sensitivity of 0.797 (95% CI: 0.737–0.849), specificity of 0.802 (95% CI: 0.742–0.853), precision of 0.801, recall of 0.797, F-Score of 0.799, accuracy of 0.8 (95% CI: 0.758–0.837), and Kappa of 0.599 in the testing dataset, with a corresponding false-negative rate (Figure 6B).

**Table 5:** Shows the AUC values of 29 different ML classification models in the training data set and the test data set.

Learner_id	Auc_train	Auc_test
abess	0.8278	0.8236
AdaBoostM1	0.8042	0.7736
CatBoost	0.9787	0.8241
cv_glmnet	0.8216	0.8189
featureless	0.5000	0.5000
fnn	1.0000	0.6875
gam	0.8288	0.8265
gamboost	0.8376	0.8291
gbm	0.8514	0.8208
glmboost	0.8274	0.8245
glmnet	0.8280	0.8250
kknn	0.9710	0.7824
ksvm	0.8651	0.8254
lda	0.8285	0.8260
liblinear	0.8288	0.8264
LightGBM	1.0000	0.8009
LMT	0.8296	0.8238
log_reg	0.8288	0.8265
naive_bayes	0.8221	0.8195
nnet	0.8497	0.8134
OneR	0.7381	0.6744
PART	0.8354	0.7806
qda	0.8263	0.8112
randomForest	1.0000	0.8172
ranger.tuned	0.8898	0.8275
rfsrc	1.0000	0.8183
rpart.tuned	0.8330	0.7754
svm.tuned	0.8416	0.8271
xgboost.tuned	0.8942	0.8254

The higher the AUC value, the better the performance of the model, and the AUC value of the test data set is more stable than that of the training data set.

### 3.6 External validation

To further assess model generalizability, an independent external validation dataset comprising 684 participants was applied (Table 1). This dataset was collected from the Affiliated Hospital of Xiangnan University (Hunan, China) and represented a distinct real-world clinical population independent of the NHANES cohort. Using the finalized Gamboost model, the external validation yielded an AUC of 0.848 (95% CI: 0.798–0.898), closely aligning with internal test performance and confirming the model's reproducibility across cohorts (Figure 6C).

In this external validation, the model achieved a sensitivity of 0.793 (95% CI: 0.757–0.825), a specificity of 0.778 (95% CI: 0.664–0.867), a precision of 0.966, a F-score of 0.871, an accuracy of 0.791 (95% CI: 0.758–0.822), and a Kappa of 0.349, reflecting a lower false-negative rate and an improved true-positive detection compared with internal testing (Figure 6C). These results demonstrated the model's robustness and its potential clinical applicability for identifying clinically significant liver fibrosis in independent patient populations. The consistency of predictive performance across datasets supports the clinical transferability of the proposed interpretable AI framework.

Furthermore, baseline comparison of the external validation cohort (Figure 6D) revealed distinct anthropometric and biochemical profiles between the clinically significant liver fibrosis and healthy groups. Participants with clinically significant liver fibrosis exhibited significantly higher waist circumference, BMI, age, log2-transformed AST, log2-transformed GGT, and urine albumin, and lower platelet counts; CPK showed no significant between-group difference. These differences are consistent with the metabolic and hepatic dysfunction patterns characteristic of clinically significant liver fibrosis, reinforcing the biological plausibility of the validation dataset and supporting the interpretability of the model's predictions.

### 3.7 Model interpretation

**Figure 7:**
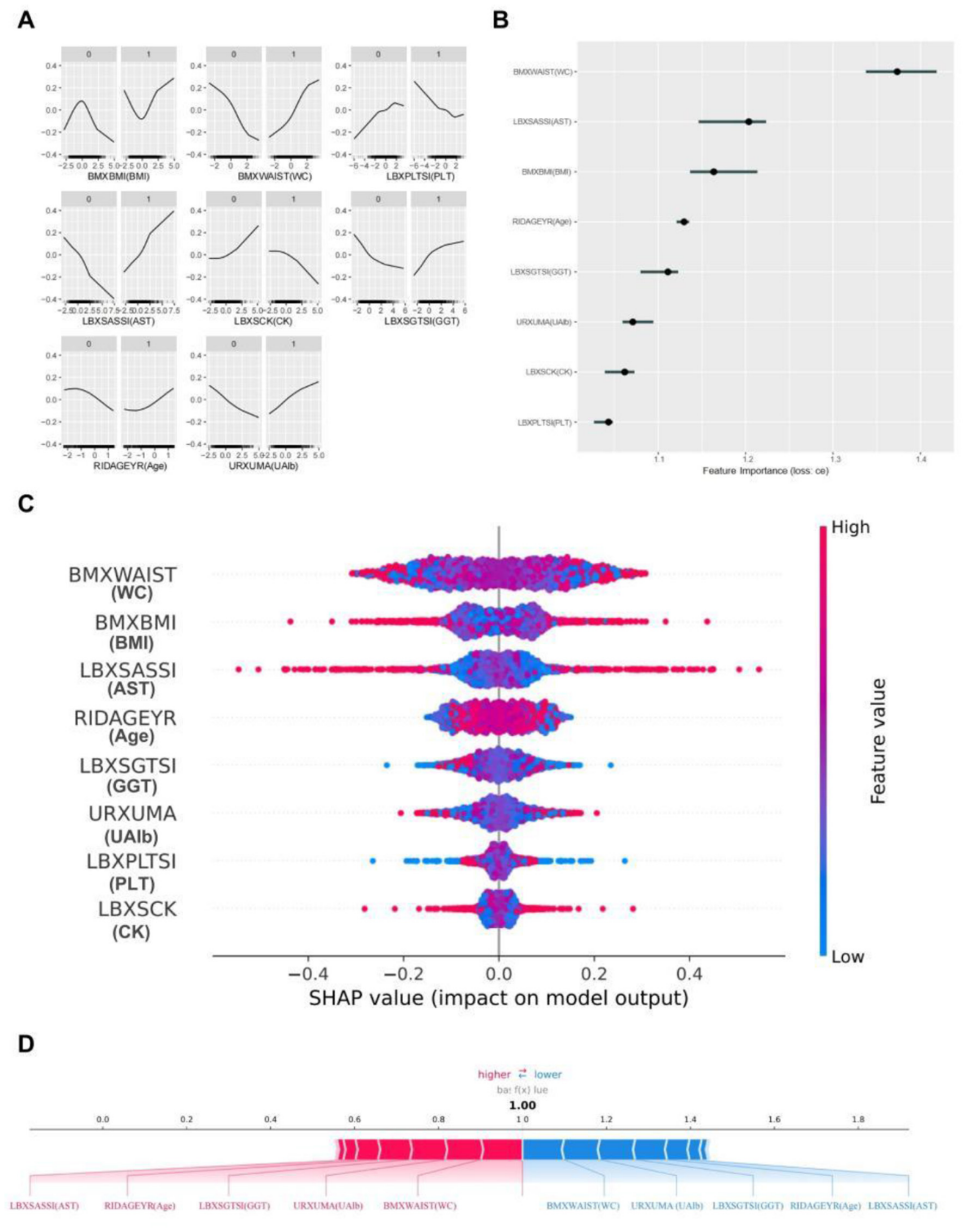
Interpretation and explanation of the Gamboost model using multiple techniques. This figure presents comprehensive interpretative analyses of the Gamboost model developed for predicting clinically significant liver fibrosis, employing various model explanation methods to understand feature impacts and model behavior. **(A)** Accumulated local effects (ALE) plots: the ALE plots illustrate the average effect of each predictor variable on the model's prediction while accounting for interactions between features. Each plot depicts how changes in a specific feature influence the predicted probability of clinically significant liver fibrosis across its range. Notably, increases in waist circumference (BMXWAIST), aspartate aminotransferase (LBXSASSI), and body mass index (BMXBMI) are associated with a higher predicted risk, indicating their strong positive contribution to fibrosis prediction. These plots provide insight into the model's localized responses and help identify non-linear relationships between predictors and the outcome. (0: control group. 1: clinically significant liver fibrosis group). **(B)** Permutation feature importance (PFI) scores: the PFI bar chart ranks the relative importance of each feature by measuring the decrease in model performance when the feature's values are randomly permuted. Waist circumference (BMXWAIST) emerges as the most influential predictor, followed by aspartate aminotransferase (LBXSASSI) and body mass index (BMXBMI). This analysis confirms the dominant role of these variables in accurately predicting clinically significant liver fibrosis and assists in prioritizing features for clinical assessment and intervention strategies. **(C)** *SHAP summary plot:* the SHapley Additive exPlanations (SHAP) summary plot combines feature importance with the direction and magnitude of each feature's impact on individual predictions. Each point represents a single prediction, colored by the feature's value (red for high, blue for low). Features such as BMXWAIST, LBXSASSI, and BMXBMI show a strong positive SHAP value correlation, indicating that higher values significantly increase the predicted risk of fibrosis. This plot highlights how individual feature values contribute across the entire dataset, providing a holistic view of feature effects and interactions. **(D)** SHAP force plot: the SHAP force plot provides a detailed explanation for a single prediction by visualizing how each feature contributes to pushing the prediction higher or lower relative to the base value, allowing for a more nuanced understanding of individual predictions (1.00: Clinically significant liver fibrosis).

To enhance the final model's interpretability, the iml R package was applied to produce ALE and PFI plots, revealing that waist circumference (WC) and aspartate aminotransferase (AST) were the most influential predictors of liver fibrosis, with body mass index (BMI) having a moderate effect, and age having a lesser impact (Figures 7A, B). SHAP values further illustrated the role of each variable in model output (Figure 7C), and Shapley analysis validated these findings (Figure 7D), demonstrating the transparency and clinical logic of the model's decision-making process.

### 3.8 Synergistic effects of key variables

**Figure 8:**
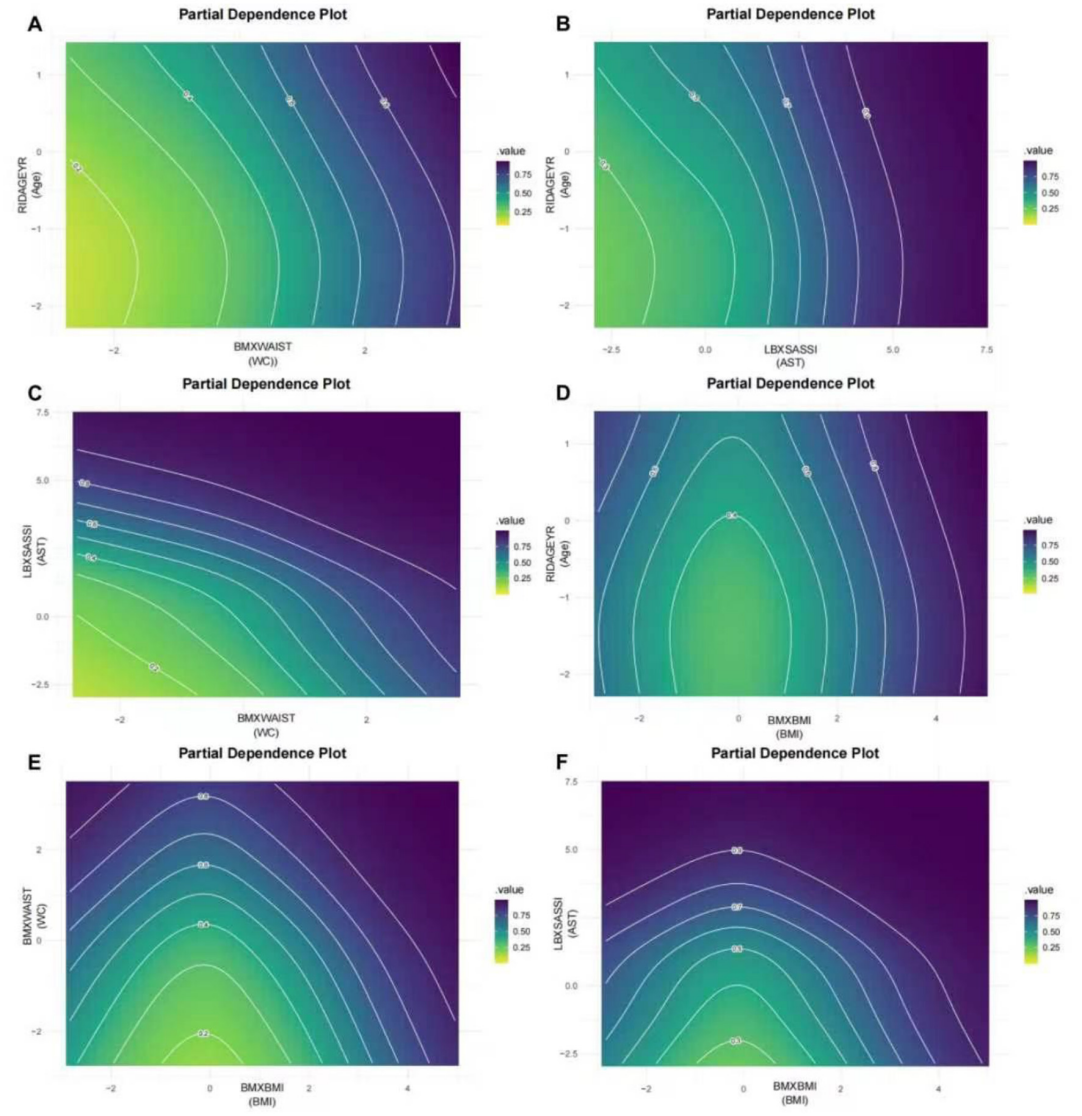
Partial dependence plots (PDPs) illustrating the interaction dynamics between selected features and their impact on the model's prediction for clinically significant liver fibrosis. **(A–F)** Each plot represents the effect of two interacting features on the prediction probability, showcasing how variations in one feature influence the predictive outcome while holding the other feature constant. These plots provide insights into the complex relationships between features such as waist circumference (BMXWAIST), aspartate aminotransferase (LBXSASSI), and BMI (BMXBMI) with the risk of clinically significant liver fibrosis. The color gradient represents the predicted probability, where warmer colors (yellow) indicate lower risk and cooler colors (blue) indicate higher risk.

Partial Dependence Plots (PDPs) illustrate the feature interactions captured by the model (Figure 8). The plots illustrated the synergistic effects of age, BMI, AST, and WC on liver fibrosis risk. For example, Figures 8A, B show that after excluding extreme values, the predicted value of liver fibrosis increases with rising log-transformed age and waist circumference. Figure 8C indicates that the predicted value increases with rising log-transformed LBXSASSI and BMXWAIST. These results suggest that waist circumference and aspartate aminotransferase levels significantly influence the prediction of liver fibrosis risk. Figures 8D–F further illustrate the predictive synergy between these key variables, emphasizing the sensitivity of fibrosis risk prediction to waist circumference and aspartate aminotransferase.

### 3.9 Model calibration, clinical utility, and comparison with established indices

To comprehensively validate the Gamboost model, we assessed its calibration, clinical utility, and comparative performance against traditional clinical scores using the external validation cohort.

First, the Gamboost model demonstrated low overall prediction error, achieving Brier scores of 0.1483 and 0.1579 in the internal testing and external validation sets, respectively. Because the model was trained on a 1:1 undersampled cohort to maximize sensitivity, a standard Bayesian prior-correction was applied to the external validation set to adjust the predicted probabilities to the actual population prevalence (~11%). Following this recalibration, the model exhibited excellent calibration agreement with the observed incidence (Brier score = 0.0727; Hosmer-Lemeshow test, *p*-value= 0.6829; Supplementary file 13).

Second, Decision Curve Analysis (DCA) was employed to evaluate the clinical consequences and practical utility of using the Gamboost model. Because the calculation of net benefit in DCA is highly sensitive to the absolute accuracy of predicted risk, we utilized the Bayesian prior-corrected probabilities for the external validation cohort to reflect the true real-world disease prevalence (11%), thereby preventing the artificial inflation of false-positive penalties. The recalibrated DCA confirmed that utilizing the Gamboost model to guide clinical interventions provided a substantially higher net clinical benefit compared to both the default “treat-all” and “treat-none” strategies. Notably, as illustrated in the decision curves, this superior net benefit was robustly sustained across a broad and clinically relevant range of threshold probabilities (from >0 to 80%). This indicates that in real-world screening scenarios, the model can effectively maximize the identification of high-risk patients while safely minimizing unnecessary downstream diagnostic procedures (such as liver biopsies) or overmedication (Supplementary file 14).

Finally, to benchmark our machine learning approach, we directly compared its discriminative performance against three widely adopted traditional non-invasive scoring systems: FIB-4, APRI, and NFS. In the external validation dataset, the Gamboost model demonstrated exceptional predictive capability, achieving an AUC of 0.848 (95% CI: 0.798–0.898). In contrast, the traditional clinical indices exhibited substantially lower discriminatory power (FIB-4 AUC: 0.616; APRI AUC: 0.616; NFS AUC: 0.725). Pairwise statistical analyses utilizing DeLong's test confirmed that the Gamboost model significantly outperformed all three conventional non-invasive models (*P* < 0.05). The consolidated ROC curves visually illustrating this comparative superiority are presented in Supplementary file 15.

## 4 Discussion

Our study aimed to develop a robust diagnostic model for clinically significant liver fibrosis by leveraging an advanced machine learning framework to integrate widely accessible clinical data. While the optimal Gamboost model demonstrated exceptional discriminative performance—yielding an internal testing AUC of 0.872 and an external validation AUC of 0.848—our primary contribution extends beyond mere predictive accuracy. Importantly, we do not claim the discovery of novel standalone biomarkers. The core predictors integrated by our recursive feature elimination (RFE) process—including waist circumference (WC), body mass index (BMI), aspartate aminotransferase (AST), and platelet count (PLT)—are well-established clinical correlates of liver fibrosis. Rather, the true translational value of our study lies in the robust integration and profound interpretability of these established indices. By employing tools such as SHapley Additive exPlanations (SHAP) and Partial Dependence Plots (PDPs), we effectively translated an AI “black box” into transparent clinical insights. Crucially, this interpretable framework allowed us to reveal and quantify complex, non-linear synergistic effects—such as the nuanced interaction between aging (RIDAGEYR) and central adiposity (BMXWAIST)—that traditional linear regression models frequently fail to capture.

Unlike conventional linear models, our machine learning approach effectively captures complex, non-linear interactions among diagnostic risk factors, providing a more robust framework for risk assessment. Machine learning facilitated the capture of complicated relationships between different features, making our prediction model more accurate and practical. Importantly, the model is designed for use in primary healthcare settings without reliance on specialized equipment or extensive training, representing a practical advance in fibrosis management. Early detection of high-risk patients facilitates prompt intervention, thereby reducing complications and improving outcomes. This approach facilitates risk-stratified management, representing a meaningful step forward in the precision care of liver fibrosis.

It is important to acknowledge that the primary clinical predictors identified by our Gamboost model—namely body mass index (BMI), waist circumference, aspartate aminotransferase (AST), and platelet count—are well-established correlates of liver fibrosis. Accordingly, the core contribution of the present study lies not in the discovery of novel standalone biomarkers, but rather in the robust, algorithm-driven integration of these widely accessible clinical indices. By leveraging an advanced machine learning framework alongside tools such as SHAP and Partial Dependence Plots (PDPs), we translate a highly complex “black-box” prediction into a transparent, clinically interpretable system. Crucially, this approach allowed us to uncover and quantify complex, non-linear synergistic effects—such as the nuanced interaction between aging and central adiposity—that traditional linear regression models frequently fail to capture.

Waist circumference (WC) has become recognized as one of the most important indicators of liver fibrosis. Waist circumference reflects the abdominal obesity and visceral fat accumulation, which are strongly linked to liver fibrosis. While the cross-sectional and observational nature of our dataset precludes causal inferences, the mechanistic underpinnings linking these algorithmically selected variables to fibrogenesis are strongly supported by existing literature. For instance, expanded waist circumference and elevated BMI are profoundly associated with visceral adiposity and systemic insulin resistance. Visceral fat accumulation may reflect a state of chronic low-grade inflammation. In this context, the increased localized release of pro-inflammatory cytokines (e.g., TNF-α and IL-6) and dysregulated adipokines has been widely implicated in the activation of hepatic stellate cells, which serves as a pivotal driver for extracellular matrix deposition and progressive liver fibrosis (31–33).

Body mass index (BMI) is commonly applied to evaluate general obesity, and it also stood out as an important predictor in our analysis (34). Elevated BMI increases susceptibility to MASLD, and its progression to liver fibrosis (35). The BMI influences liver fibrosis primarily through insulin resistance, systemic inflammation, and adipokine dysregulation (36). Obesity induces a pro-inflammatory state, with increased levels of inflammatory factors like TNF-α and IL-6. This environment encourages the stimulation of stellate cells, leading to liver fibrosis (37). The strong correlation between BMI and liver fibrosis, emphasizes the need for weight management as a precaution and prevention strategy for liver fibrosis (38).

Aspartate aminotransferase (AST) was another key feature in our model, which is consistent with its well-established association with hepatocellular injury and fibrogenic progression (39). Elevated AST levels are indicative of hepatocellular membrane damage and mitochondrial dysfunction, processes that frequently accompany the transition from simple steatosis to clinically significant liver fibrosis (40). Increased AST levels reflect the leakage of intracellular enzymes caused by necro-inflammatory injury, which subsequently triggers hepatic stellate cell activation and extracellular matrix deposition—central events in the pathogenesis of fibrosis (41). Moreover, a disproportionate elevation of AST relative to alanine aminotransferase often indicates advanced fibrotic remodeling, as mitochondrial AST predominates in settings of chronic injury where hepatocyte regeneration is impaired (42). Many studies from population-based and biopsy-validated cohorts have demonstrated that higher AST levels or an elevated AST/ALT ratio are associated with the severity of fibrosis in patients with MASLD and viral hepatitis (43). Consistent with these findings, our model identified AST as a key predictor, highlighting its dual role: it serves both as a biochemical marker for hepatocellular injury and a surrogate indicator for fibrogenic progression. These results show the potential value of routine AST monitoring in the early identification and risk stratification of patients at risk of progressing to clinically significant liver fibrosis.

Platelet count (PLT) has long been regarded as a reliable clinical marker in liver disease assessment. Thrombocytopenia, or low platelet count, is commonly observed in liver fibrosis and cirrhosis due to splenic sequestration and decreased thrombopoietin production by the liver (44). The platelet count serves as an indirect measure of portal hypertension and liver synthetic function (45). Studies have shown that lower platelet counts are indicative of more severe fibrosis, making it a valuable non-invasive marker for liver fibrosis staging (46). Similarly, the inclusion of AST and platelet count by our predictive framework aligns cohesively with recognized pathophysiological cascades. Elevated AST frequently serves as a reliable surrogate marker for ongoing hepatocyte injury and mitochondrial dysfunction, processes that are intrinsically linked to active fibrotic remodeling (47). Conversely, a decline in platelet count is a well-documented consequence of advanced liver disease, likely reflecting a combination of portal hypertension-induced splenic sequestration and diminished hepatic production of thrombopoietin (48). Ultimately, our interpretable AI model essentially mirrors these established biological pathways, validating its clinical logic while maximizing diagnostic accuracy through optimal feature weighting.

Creatine kinase (CK), typically associated with muscle damage, has also been linked to liver fibrosis. Elevated levels of creatine kinase may indicate muscle inflammation or wasting, conditions often seen in liver fibrosis (49). The inclusion of LBXSCK in our predictive model suggests that muscle-related biomarkers may provide additional insights into the systemic effects of liver disease. This finding aligns with studies indicating significant alterations in muscle metabolism in chronic liver diseases (50).

Gamma-glutamyl transferase (GGT) is a crucial enzyme in glutathione metabolism and is often elevated in individuals with liver disease (51). GGT levels tend to increase with the severity of fibrosis because higher GGT indicates greater oxidative stress and inflammation in the liver (52). The role of GGT in liver fibrosis is supported by its involvement in oxidative stress pathways that promote hepatic stellate cells into an activated state and enhance collagen accumulation (53). This suggests that monitoring GGT levels may also be useful in the evaluation of liver function and fibrosis risk (54).

Age is a significant factor influencing liver fibrosis progression. Older individuals are more susceptible to fibrogenesis due to cumulative liver damage and reduced regenerative capacity (55). Age-related changes in the liver, such as reduced regenerative capacity and increased susceptibility to oxidative stress, tend to accelerate the progression of fibrosis (56). Given the strong association between age and liver fibrosis, there is a crucial need to focus on interventions in older populations to prevent advanced liver disease (57).

Urinary albumin (UAIb), often measured as the urinary albumin-to-creatinine ratio (UACR), is an important marker in liver fibrosis assessment (58). Elevated urinary albumin levels, known as microalbuminuria, have been associated with liver fibrosis, particularly in the context of MASLD (59). Microalbuminuria reflects endothelial dysfunction and increased vascular permeability, which is common in chronic liver diseases and worsens the prognoses. The presence of microalbuminuria indicates increased susceptibility to severe fibrosis, making it a useful marker for identification of patients prone to advanced liver disease at an earlier stage (59).

Our study's strengths include the use of a large, diverse dataset from NHANES, the application of advanced machine learning techniques, and the comprehensive analysis of multiple risk factors. By leveraging the latest mlr3 framework, we constructed a predictive model based on routine examination and blood test data. This approach requires no specialized equipment or extensive training, ensuring high predictive accuracy and generalizability, which is advantageous for primary healthcare applications. However, several critical limitations must be transparently acknowledged. First, the cross-sectional and observational nature of the NHANES database precludes the establishment of causal relationships. Consequently, our model provides diagnostic risk stratification rather than predicting the longitudinal progression of liver fibrosis or future liver-related clinical events. Second, as is inherent in community-based datasets, the NHANES cohort encompasses a high heterogeneity of underlying etiologies. Applying a universal transient elastography stiffness cutoff across this mixed-etiology population carries a theoretical risk of label noise and potential misclassification. Third, to maximize data utility and preserve sample size, missing clinical variables were addressed using standard mlr3 imputation algorithms (e.g., k-nearest neighbors); nevertheless, such imputation inherently carries the risk of introducing statistical bias or artificial correlations. Furthermore, residual confounding remains possible due to unmeasured covariates such as detailed medication history. Finally, while the independent external cohort strongly validated the model's trans-regional transportability, it was derived retrospectively from a single clinical center. Therefore, future prospective, multi-center studies are warranted to fully confirm the model's global clinical applicability.

Cohort differences and model generalizability. A notable strength of the present study lies in the profound demographic and clinical contrasts between the derivation and external validation cohorts across three key dimensions: population setting, disease spectrum, and ethnicity. The NHANES training set represents a heterogeneous, multi-ethnic, community-based cross-section of the general United States population, where the majority of individuals are asymptomatic and present with a broad, unclassified mix of etiologies. Conversely, the external validation cohort comprises a homogenous, entirely Asian (Han Chinese) clinical population derived from a hospital setting in China. This clinical cohort inherently carries a higher pre-test probability of liver abnormalities and is highly enriched for specific metabolic etiologies; notably, when re-evaluated using the latest 2023 AASLD/EASL Delphi consensus criteria, the disease spectrum was predominantly driven by Pure MASLD and Alcohol-Associated Liver Disease (ALD). Rather than a limitation, these striking disparities serve as a rigorous “stress-test” for the model's external validity. The observation that the Gamboost framework—trained on a highly mixed, Western community population—sustained robust diagnostic accuracy (AUC = 0.848, 95% CI: 0.798–0.898) and superior clinical net benefit when applied to an Eastern, hospital-based metabolic cohort strongly underscores its trans-regional and trans-etiological generalizability. This robust transportability suggests that the core features integrated into our model (such as waist circumference, body mass index, and AST) capture the fundamental mechanisms of fibrogenesis and metabolic dysregulation that are globally conserved across different ethnicities and healthcare settings.

In summary, our study successfully identified and validated key predictors of clinically significant liver fibrosis through machine learning–based modeling. The identified features—waist circumference, AST, BMI, platelet count, creatine kinase, GGT, age and urine albumin—offer valuable insights into the multifactorial nature of liver fibrosis. The significant impact of BMI, aspartate aminotransferase, and waist circumference on liver fibrosis underscores the importance of targeting these modifiable risk factors in clinical strategies.

## 5 Conclusion

In conclusion, we established a reliable machine learning model for predicting clinically significant liver fibrosis using readily available examination and blood test data. Our model is easy to apply in primary healthcare and provides accurate results. Ultimately, by facilitating early identification and timely intervention, this framework translates the concepts of precision medicine into actionable clinical strategies.

## Data Availability

The original contributions presented in the study are included in the article/Supplementary material, further inquiries can be directed to the corresponding authors.

## Supplementary material

Supplementary file 1 Flowchart of the patient enrollment and selection process for the independent external validation cohort. A total of 1,625 patients evaluated at the Affiliated Hospital of Xiangnan University between January 2018 and December 2023 were initially screened. Patients were sequentially excluded based on two primary criteria: the presence of specific or competing liver etiologies (*n* = 351), including viral hepatitis B or C (*n* = 214), autoimmune liver disease or drug-induced liver injury (*n* = 42), and heavy alcohol consumption confounding strict subtyping (*n* = 95); and **(****2****)** incomplete clinical data (*n* = 590), specifically missing reliable liver stiffness measurement (LSM)/elastography data (*n* = 412) or essential demographic and laboratory parameters (*n* = 178). The final external validation cohort comprised 684 eligible participants, consisting of 606 controls (non-significant liver fibrosis) and 78 cases (significant liver fibrosis) utilized for downstream model validation.

Supplementary file 2 Contemporary etiological distribution of the external validation cohort based on the 2023 Steatotic Liver Disease (SLD) criteria. Patients with clinically significant liver fibrosis in the real-world hospital cohort were retrospectively re-evaluated using the latest AASLD/EASL Delphi consensus. The disease spectrum was predominantly driven by Pure MASLD (55.1%, *n* = 43) and Alcohol-Associated Liver Disease (ALD; 15.4%, *n* = 12), with the newly defined MetALD accounting for 1.3% (*n* = 1). Owing to the inherent limitations of retrospective electronic medical records—specifically the absence of precise quantitative alcohol consumption logs required for strict contemporary subtyping-−28.2% (*n* = 22) of the participants were categorized as other/unclassified.

Supplementary file 3 The figure shows the percentage distribution of clinically significant liver fibrosis cases and control cases across different categories of each categorical variable.

Supplementary file 4 The figure shows the frequency distribution of different categories for each categorical variable included in this study, and intuitively presents the number of samples corresponding to different categories of each categorical variable.

Supplementary file 5 This figure presents the numerical distribution characteristics of each continuous variable included in this study in the clinically significant liver fibrosis case group (T) and the control case group (C) through a collection of box plots, including the median, quartiles (25th and 75th percentiles), maximum value, minimum value, and potential outliers of the data.

Supplementary file 6 This document presents a series of box plots derived from the logarithmic transformation results of the original continuous variables in “**Supplementary file 5**”. Each subplot corresponds to one indicator, and by comparing the box plots of the control group (C) and the clinically significant liver fibrosis case group (T), it intuitively displays the median, quartiles, and distribution range of the transformed data.

Supplementary file 7 This figure presents the distribution characteristics of the original values of relevant continuous variables in the clinically significant liver fibrosis study through a series of histograms. Each subplot corresponds to one indicator, where the horizontal axis represents the original measured values of the variable, and the vertical axis shows the frequency of occurrence of the values. These histograms intuitively display the laws of data distribution, facilitating a quick judgment on the degree of variable skewness and providing a basis for subsequent data analysis— the more significant the skewness of a variable, the more necessary it is to perform data transformation to meet the model assumptions.

Supplementary file 8 This figure presents the numerical distribution characteristics of relevant continuous variables in the clinically significant liver fibrosis study after logarithmic transformation through a series of histograms. Each subplot corresponds to one indicator, intuitively showing the frequency distribution patterns of the transformed data, and providing an optimized data distribution reference for machine learning models in feature processing and analyzing the correlation between variables and clinically significant liver fibrosis.

Supplementary file 9 This table provides detailed baseline demographic, anthropometric, and clinical laboratory characteristics for the entire NHANES 2017-2020 cohort (*N* = 6164) after data cleaning, Data are stratified according to clinically significant liver fibrosis status (Control vs. Case). Categorical variables are presented as *n* (%), and continuous variables are presented as mean ± standard deviation (SD) or median (interquartile range, IQR), as appropriate. Between-group differences were evaluated using Pearson's Chi-squared test or Fisher's exact test for categorical variables, and the Wilcoxon rank sum test for continuous variables.

Supplementary file 10 We employed the Recursive Feature Elimination (RFE) method for feature screening. Using the Area Under the Curve (AUC) as the core indicator, we evaluated the predictive performance of seven machine learning models (Gbm-RFE, LightGBM-RFE, Ranger-RFE, Rpart-RFE, Svm-RFE, Xgboost-RFE, and CatBoost-RFE) with changes in the number of features. The chart intuitively presents the changing trends of AUC values for different models as the number of features decreases during the feature selection process, providing a basis for selecting the optimal feature combination and model.

Supplementary file 11 We adopted the Recursive Feature Elimination (RFE) method for feature selection, with the goal of maximizing the AUC value, and used five-fold cross-validation to evaluate the performance and effectiveness of feature selection. The table shows the best features (in the “best” column) and classification error rates (in the “ce” column) obtained after feature selection by different RFE models.

Supplementary file 12 The figure shows that during the benchmark model selection process, we used the Precision-Recall curve (PR curve) and the Receiver Operating Characteristic curve (ROC curve) to compare the clinically significant liver fibrosis classification performance of 29 machine learning classification models. All models were constructed based on the eight key variables identified through feature selection in the study and internally evaluated via five-fold cross-validation, aiming to intuitively demonstrate the classification and discriminative predictive ability of different models for clinically significant liver fibrosis patients in the population at the benchmark level.

Supplementary file 13 Calibration plots of the Gamboost model across internal and external cohorts. **(A)** Calibration curve in the internal testing set, demonstrating initial model fit (Brier score = 0.1483; Hosmer-Lemeshow [H-L] test *P* = 0.0309). **(B)** Calibration curve in the external validation cohort prior to prevalence adjustment (Brier score = 0.1579; H-L test *P* < 0.001). **(C)** Recalibrated curve for the external validation cohort following Bayesian prior-correction. The mathematically adjusted model exhibited excellent agreement between predicted probabilities and actual observed frequencies of clinically significant liver fibrosis, evidenced by a substantially minimized Brier score (0.0727) and a non-significant H-L goodness-of-fit test (*P* = 0.6829).

Supplementary file 14 Decision curve analysis (DCA) evaluating the clinical utility of the Gamboost model. The graphs depict the net clinical benefit of utilizing the Gamboost predictive framework (red solid line) to guide interventions, compared against the default strategies of treating all patients (dashed grey line) or treating no patients (solid black line) across a continuum of threshold probabilities. **(A)** Internal Testing Set. In the 1:1 undersampled internal cohort, the Gamboost model demonstrated a substantial net clinical benefit over default strategies across the entire spectrum of risk thresholds. **(B)** External Validation Cohort. DCA was conducted after applying a Bayesian prior-correction (prevalence calibration) to strictly reflect the real-world incidence rate of advanced liver fibrosis in the hospital setting. The recalibrated Gamboost model consistently yielded a superior net clinical benefit across clinically relevant threshold probabilities. This confirms that implementing the Gamboost model for non-invasive risk stratification provides tangible, real-world value for clinical decision-making.

Supplementary file 15 Head-to-head comparison of discriminative performance between the Gamboost framework and traditional non-invasive fibrosis scores. Receiver operating characteristic curves illustrate the predictive accuracy of the Gamboost model compared against FIB-4, APRI, and the NAFLD Fibrosis Score (NFS). **(A)** In the internal testing set, Gamboost (AUC = 0.872) significantly outperformed all traditional indices (FIB-4: 0.616; APRI: 0.616; NFS: 0.725). **(B)** In the independent external validation cohort, the Gamboost model maintained robust predictive superiority (AUC = 0.848) over FIB-4 (0.618), APRI (0.611), and NFS (0.749), confirming its exceptional cross-cohort generalizability and clinical utility.
