# Supplementary figures and images for: Integrative and interpretable machine learning framework for early non-invasive detection of clinically significant liver fibrosis
Source: Front Med (Lausanne). 2026 Jun 23;13:1736295. doi: 10.3389/fmed.2026.1736295 (PMC13337473; doi:10.3389/fmed.2026.1736295)

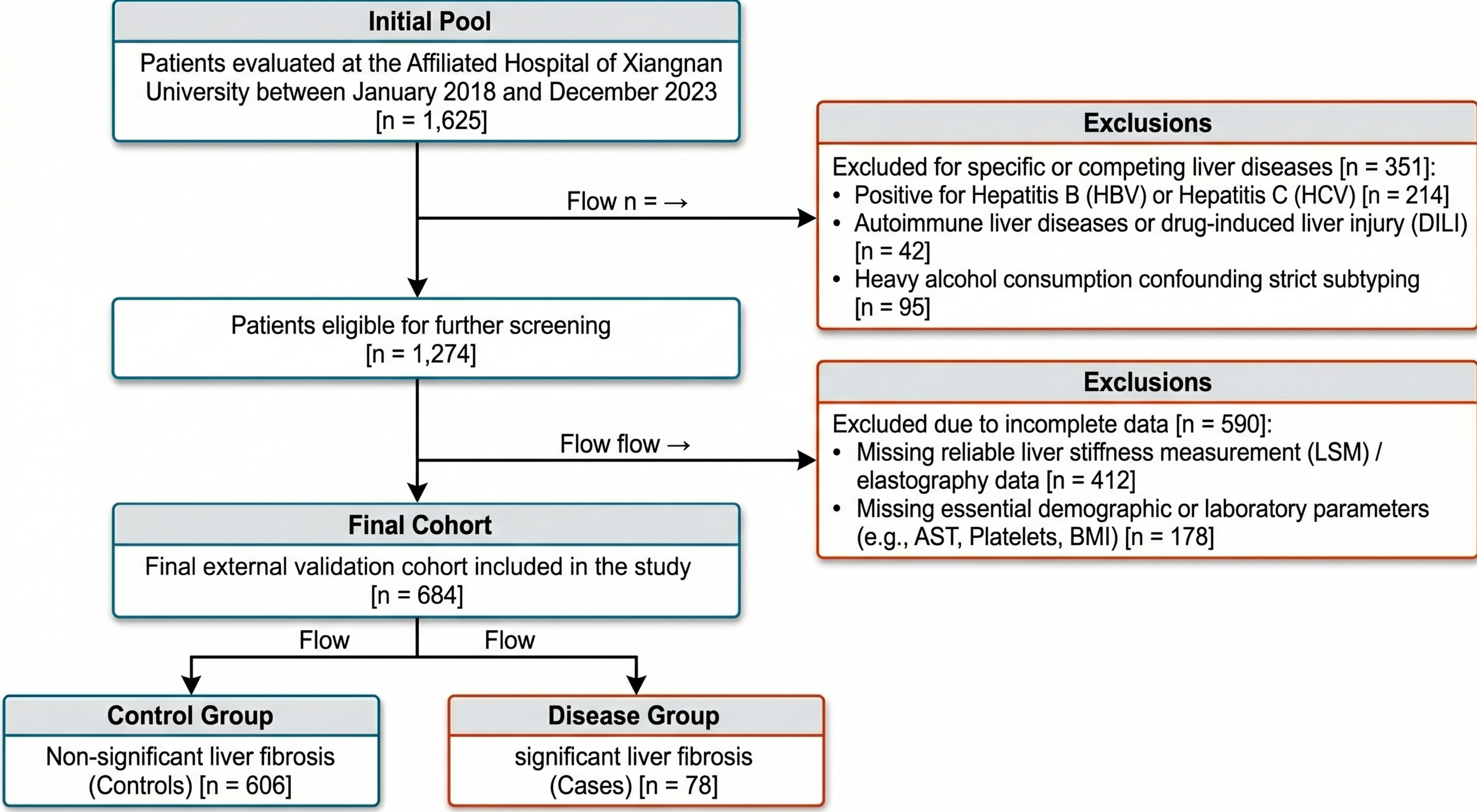

Supplement: Supplementary file 1 — Flowchart of the patient enrollment and selection process for the independent external validation cohort. A total of 1,625 patients evaluated at the Affiliated Hospital of Xiangnan University between January 2018 and December 2023 were initially screened. Patients were sequentially excluded based on two primary criteria: the presence of specific or competing liver etiologies (n = 351), including viral hepatitis B or C (n = 214), autoimmune liver disease or drug-induced liver injury (n = 42), and heavy alcohol consumption confounding strict subtyping (n = 95); and (2) incomplete clinical data (n = 590), specifically missing reliable liver stiffness measurement (LSM)/elastography data (n = 412) or essential demographic and laboratory parameters (n = 178). The final external validation cohort comprised 684 eligible participants, consisting of 606 controls (non-significant liver fibrosis) and 78 cases (significant liver fibrosis) utilized for downstream model validation. [file Data_Sheet_1.pdf]

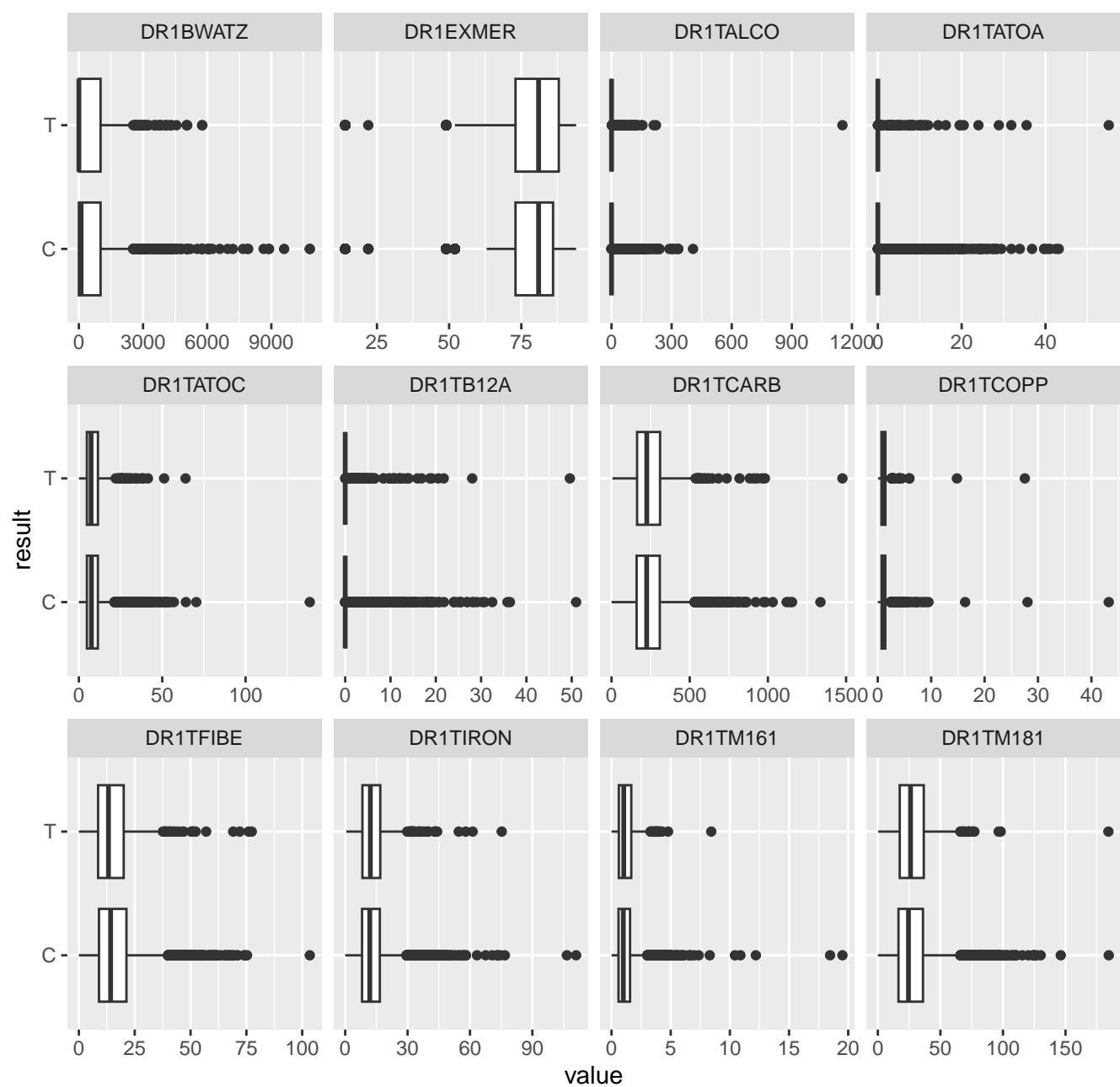

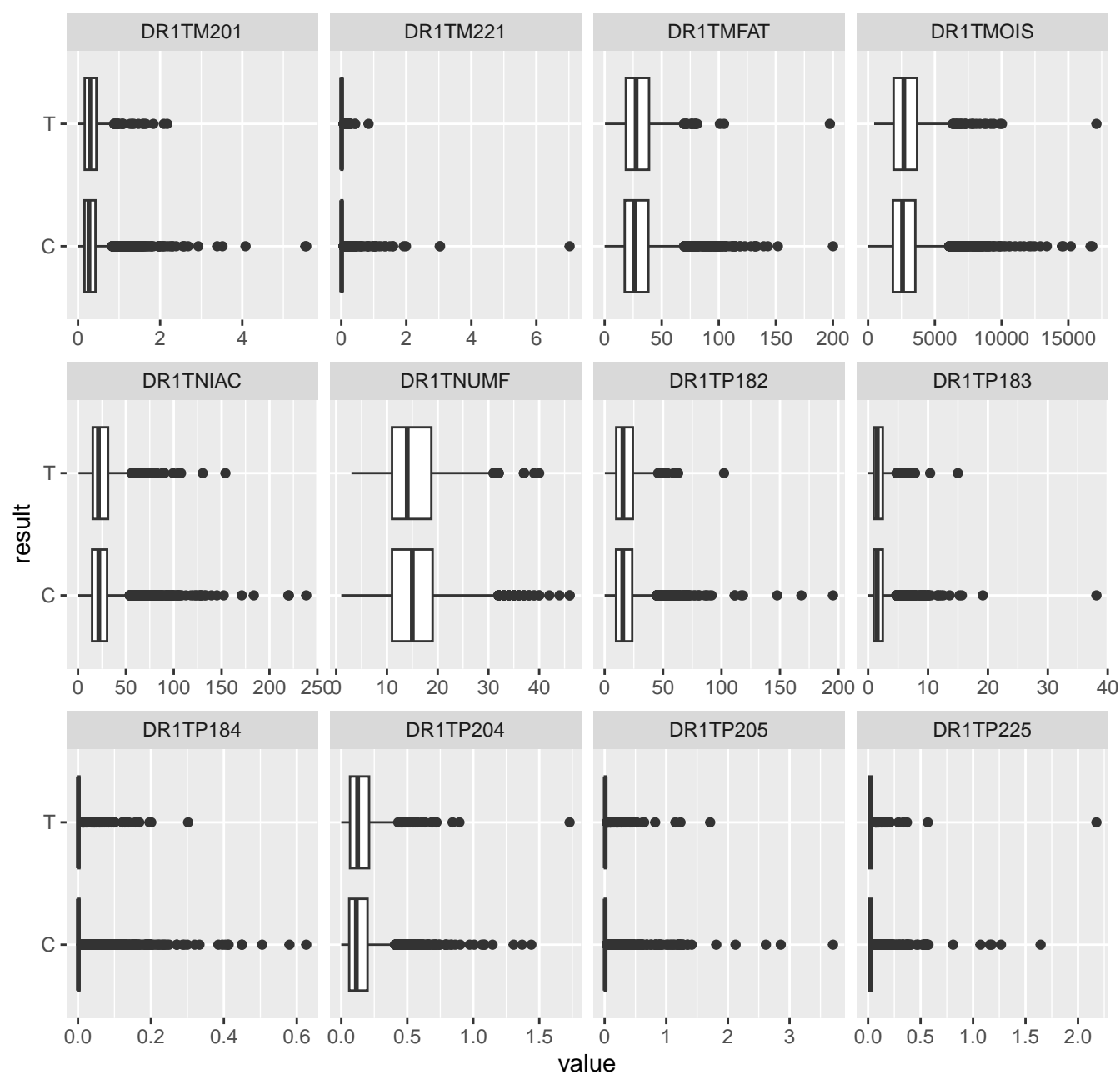

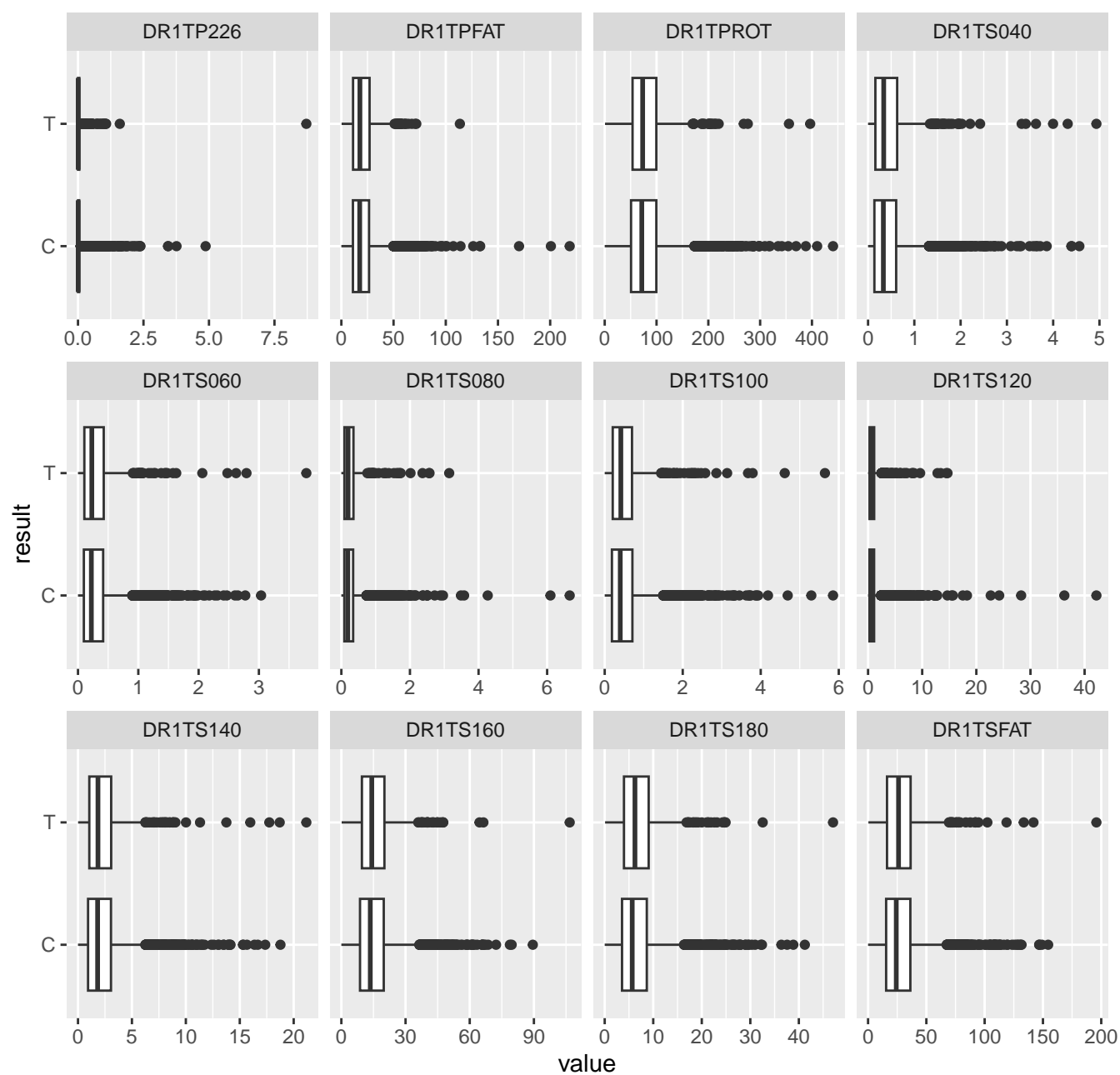

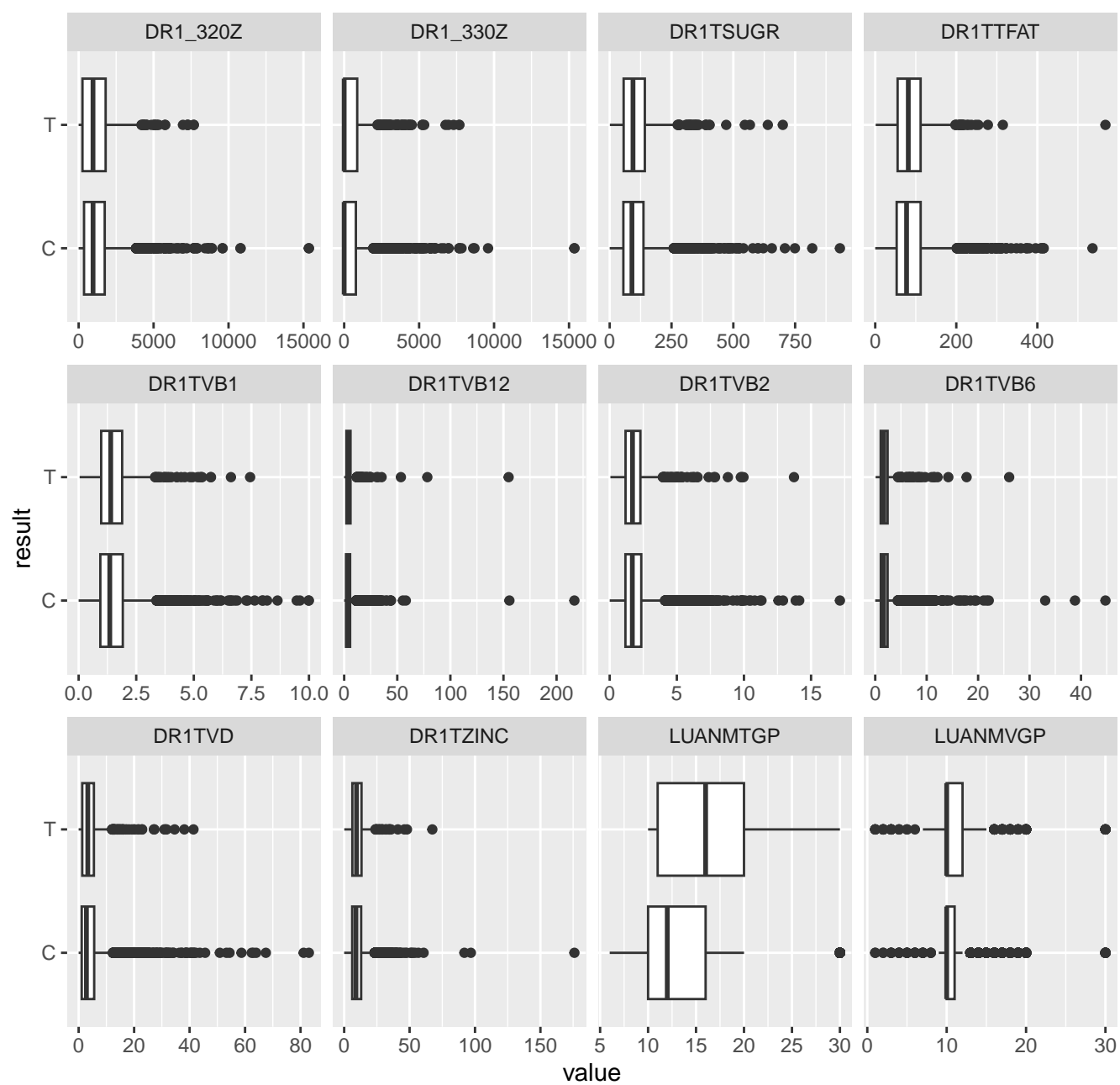

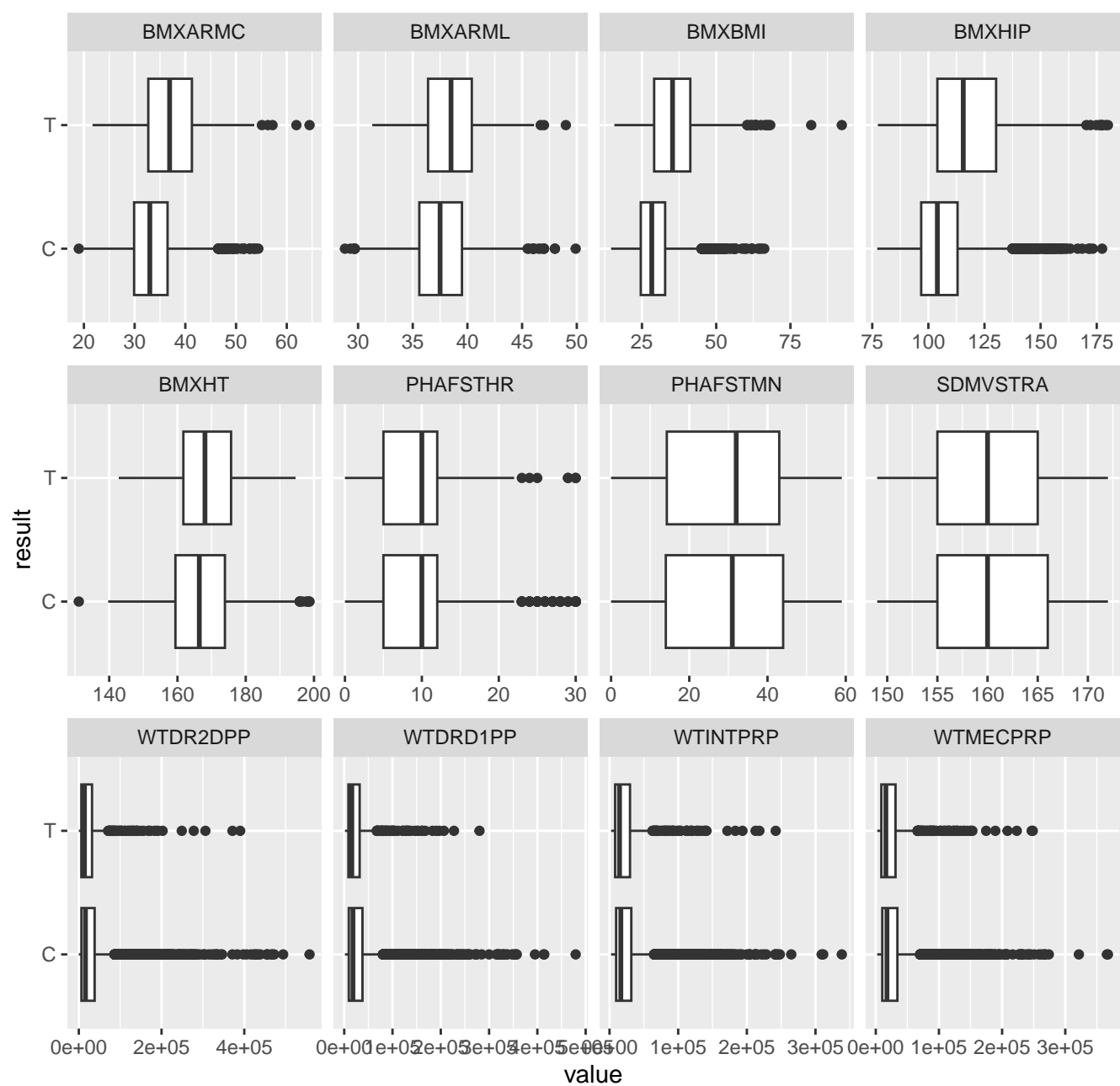

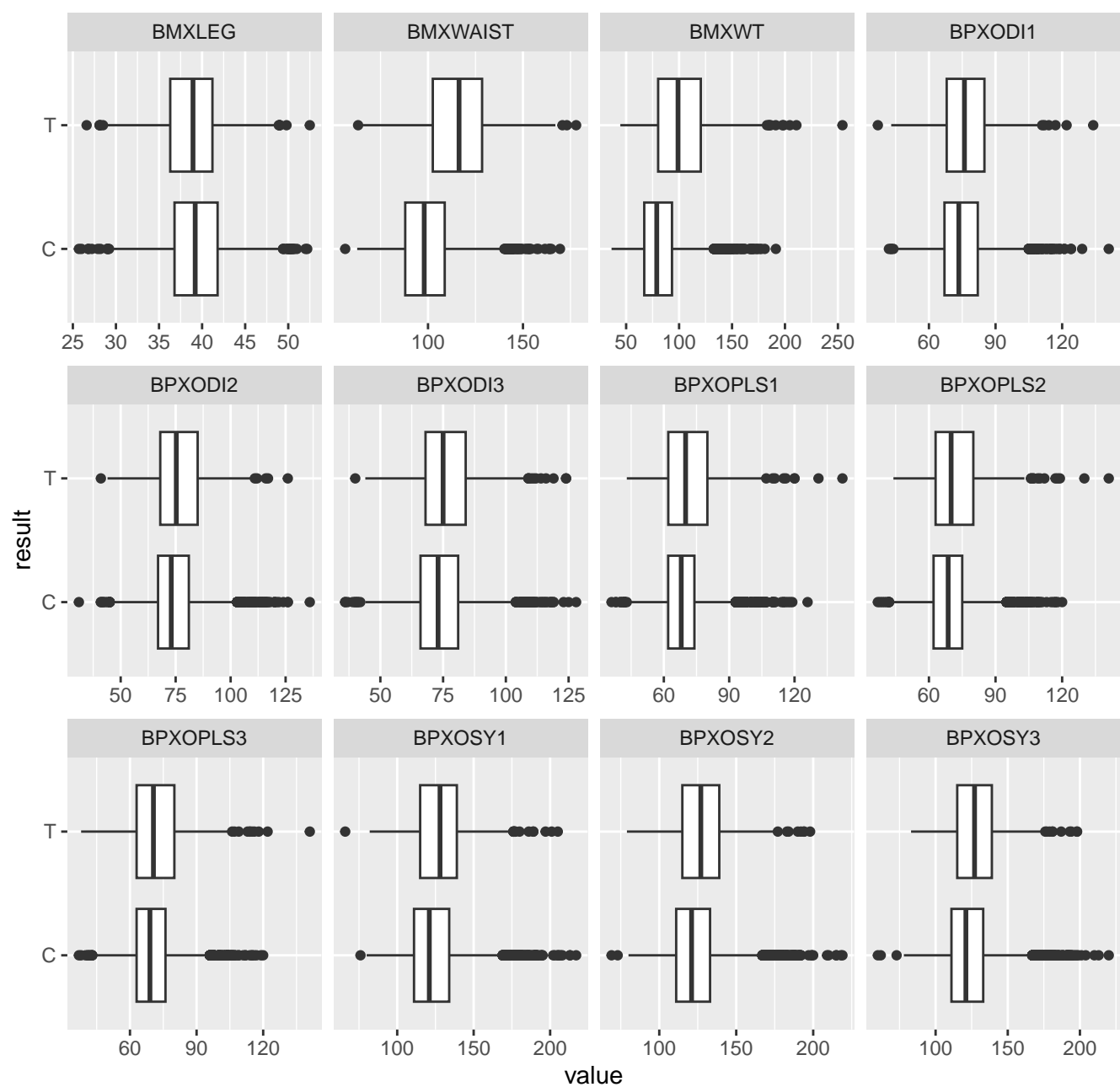

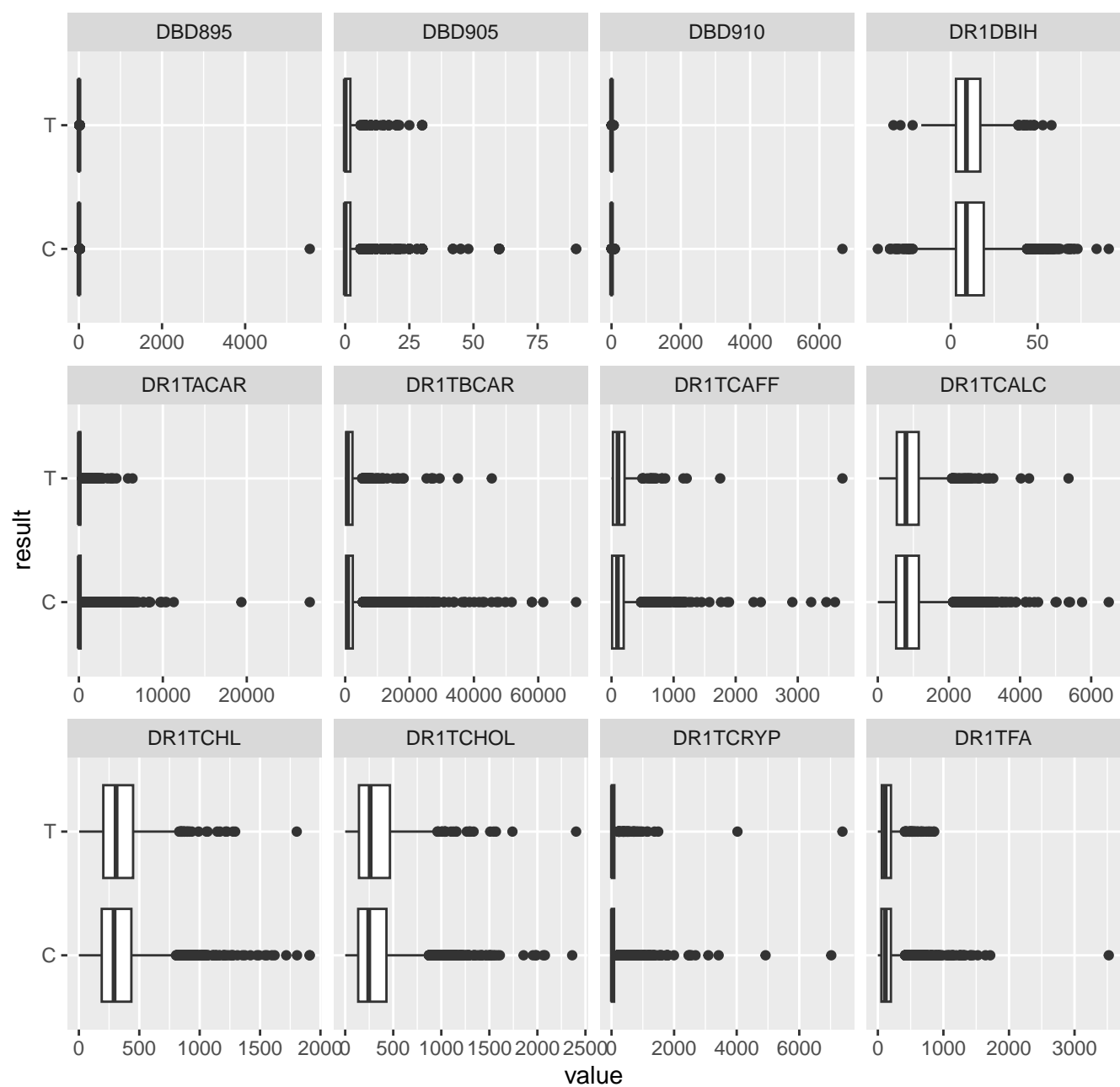

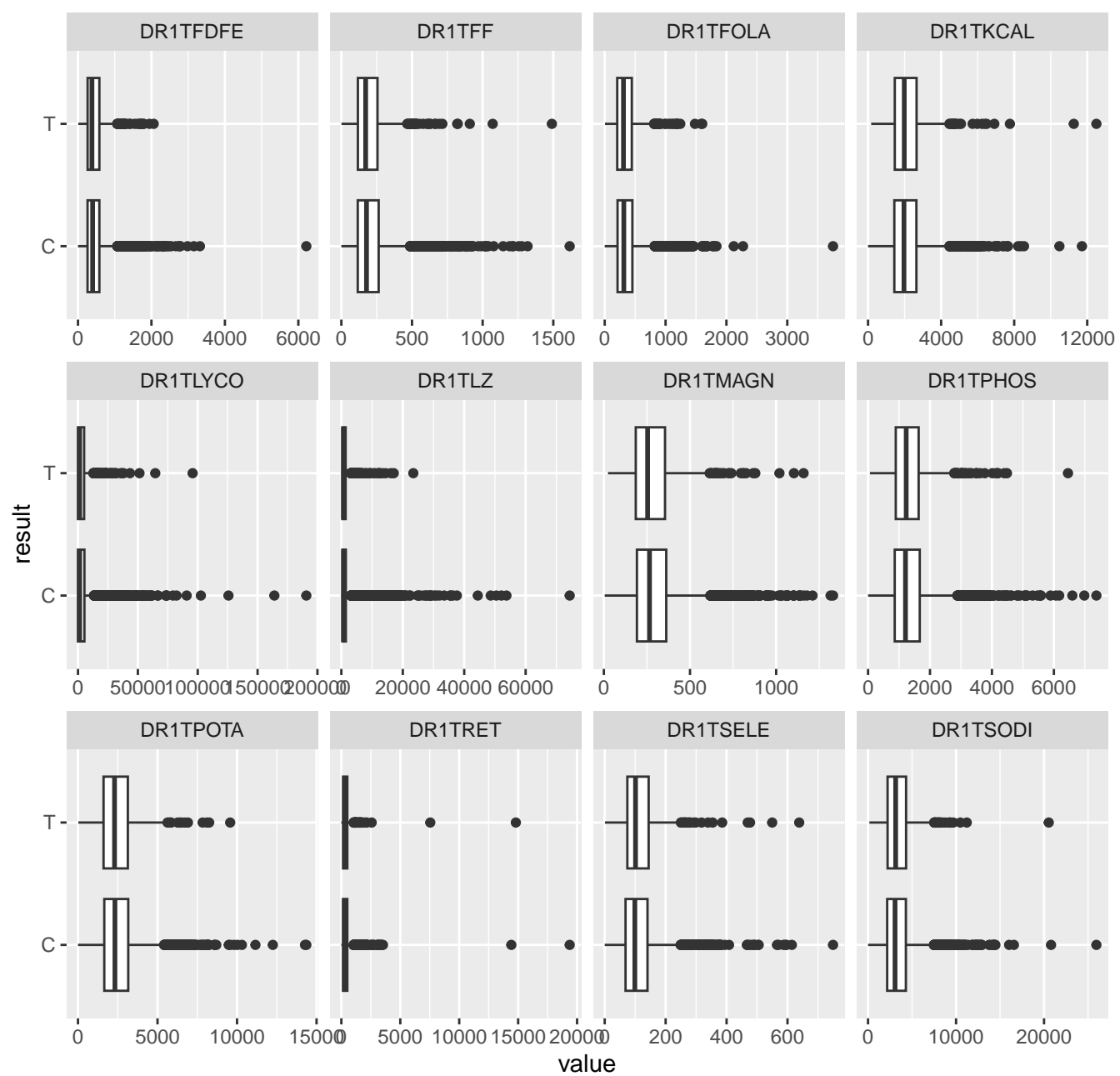

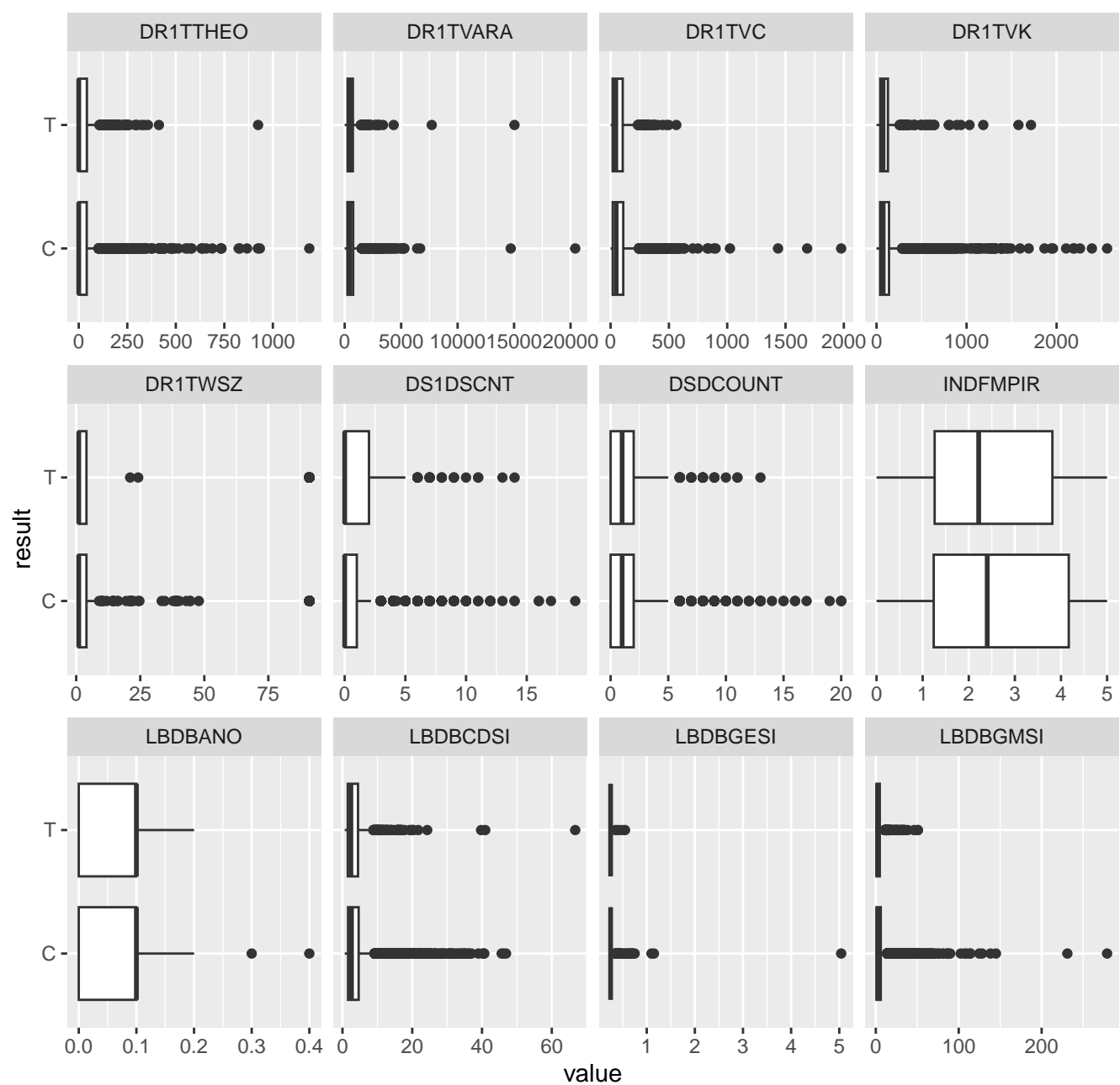

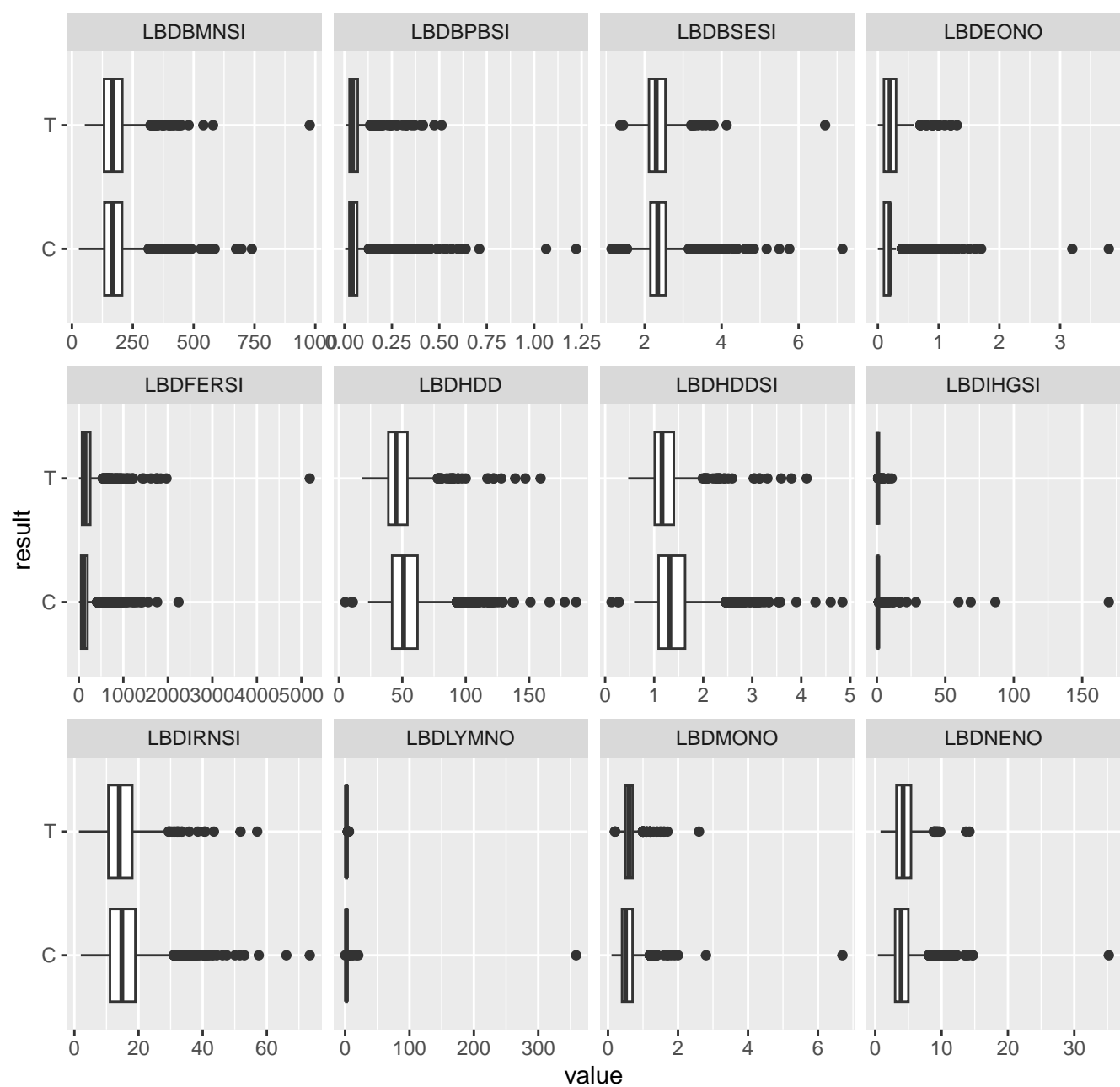

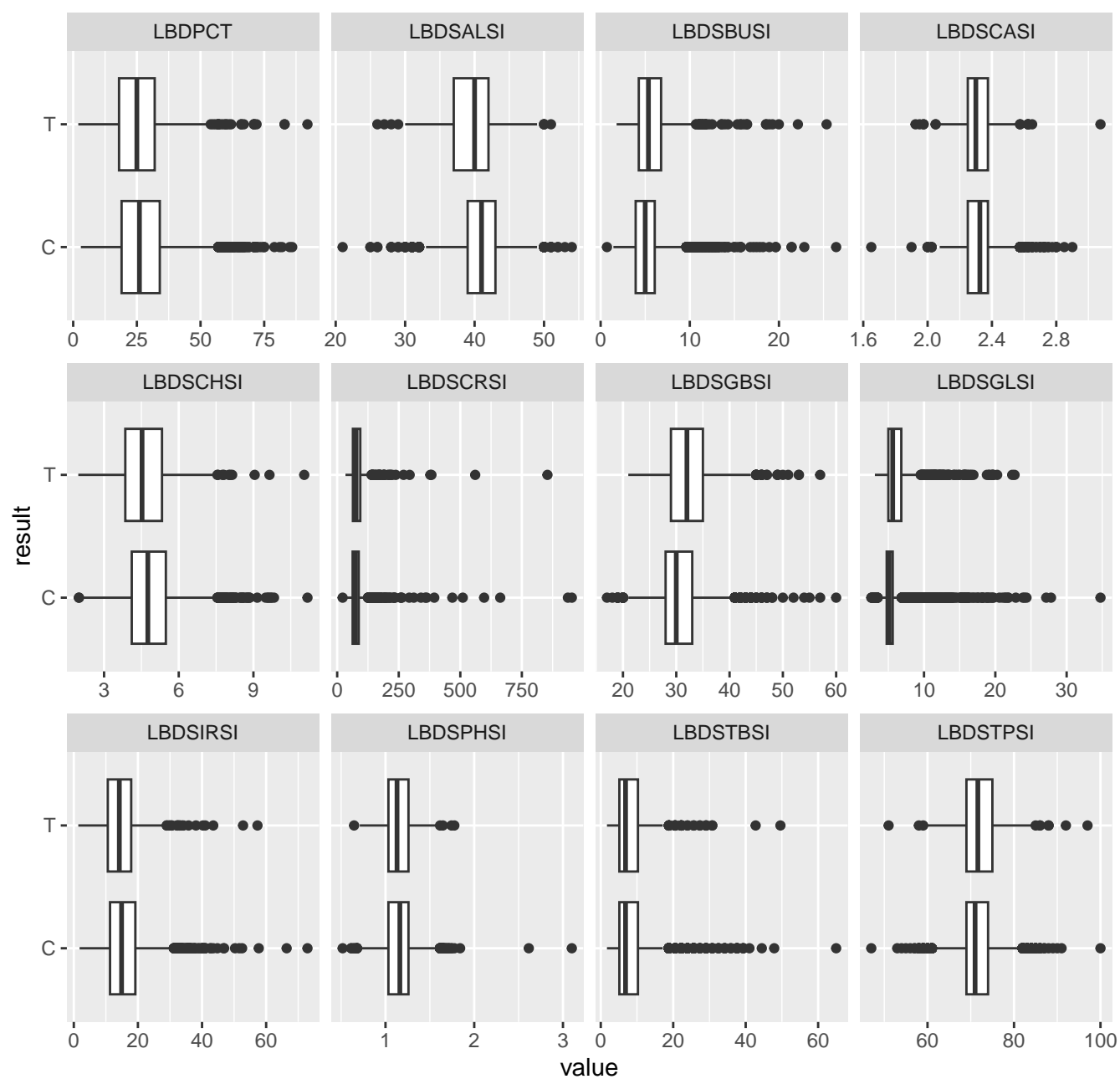

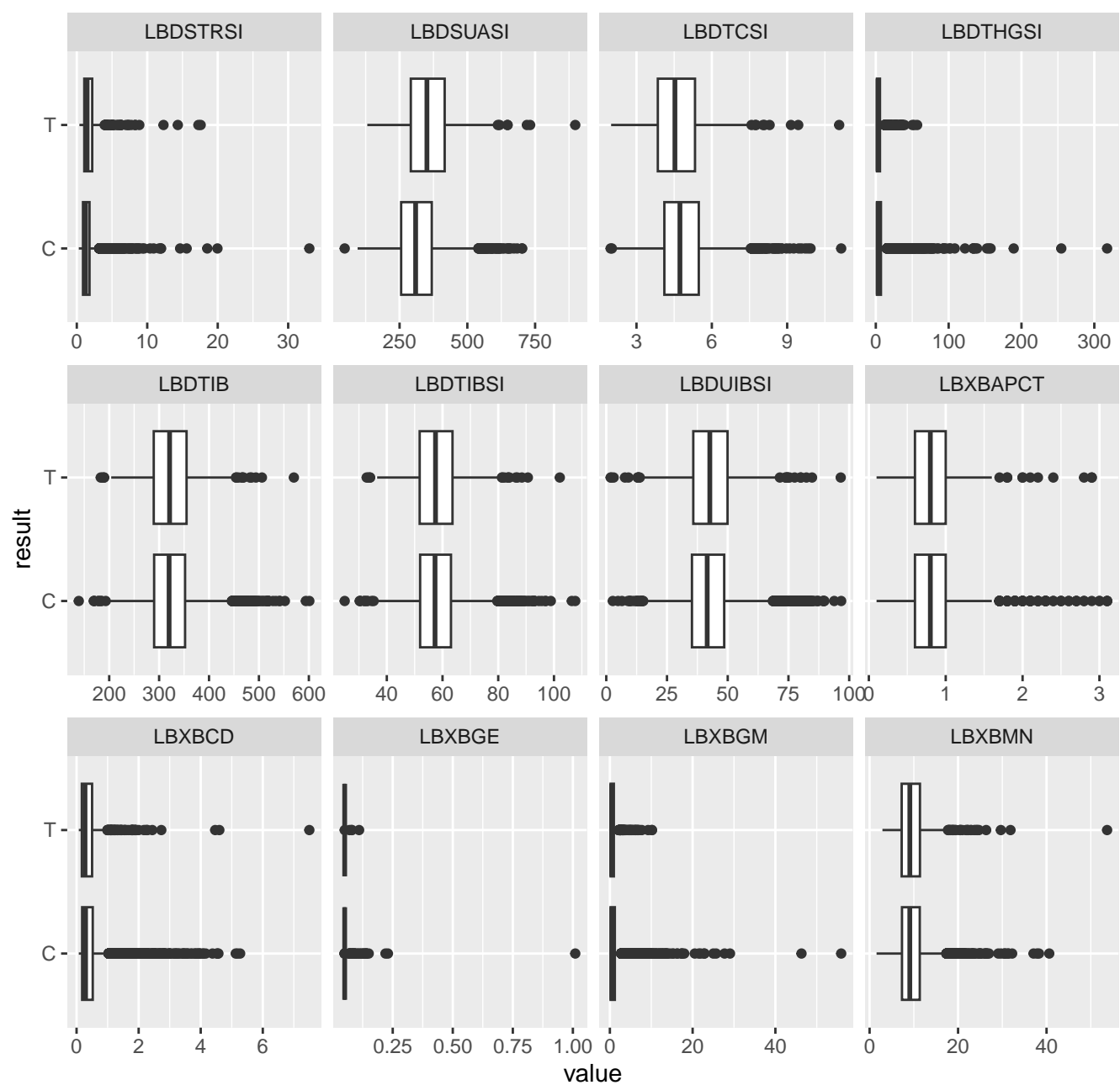

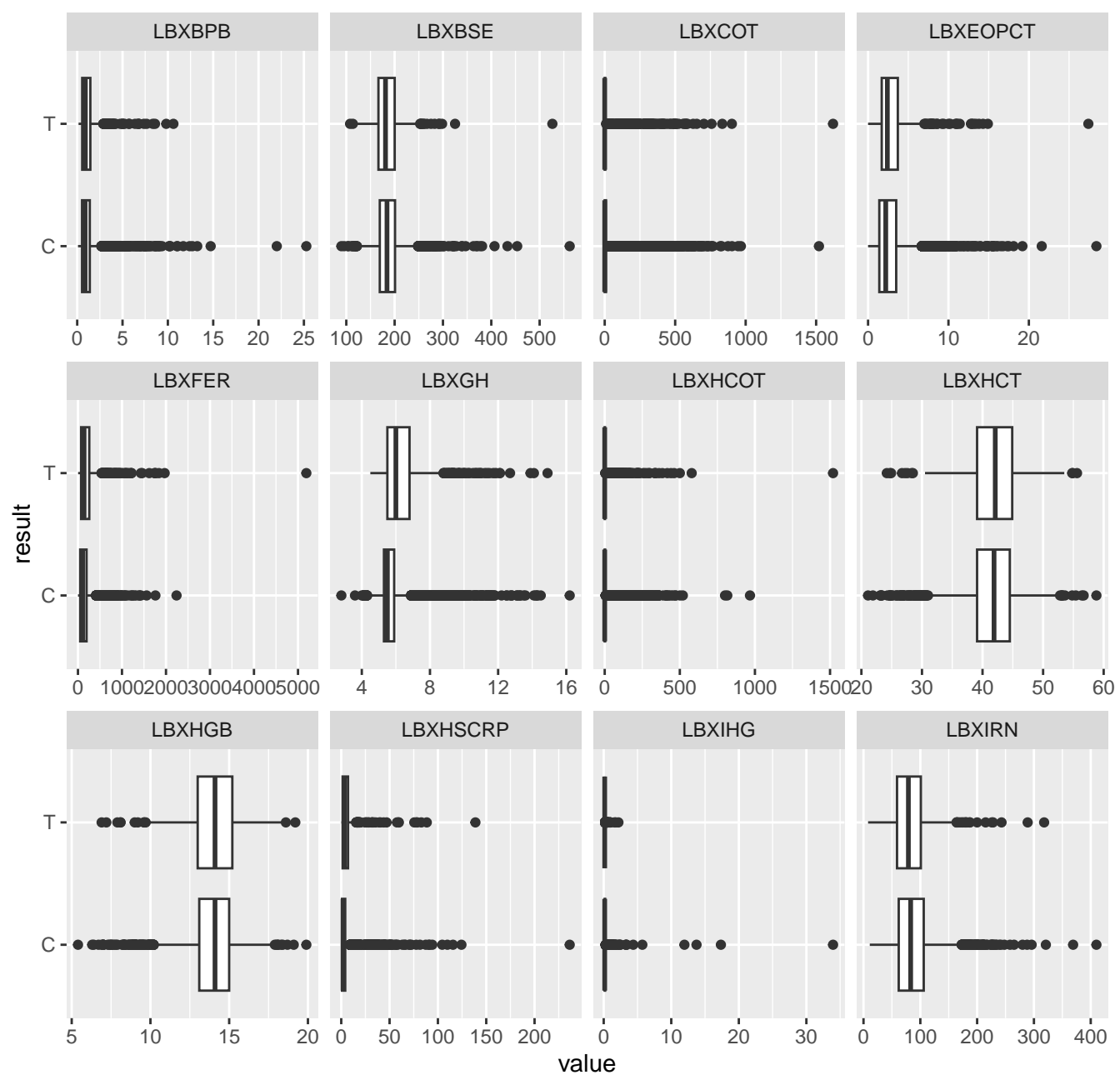

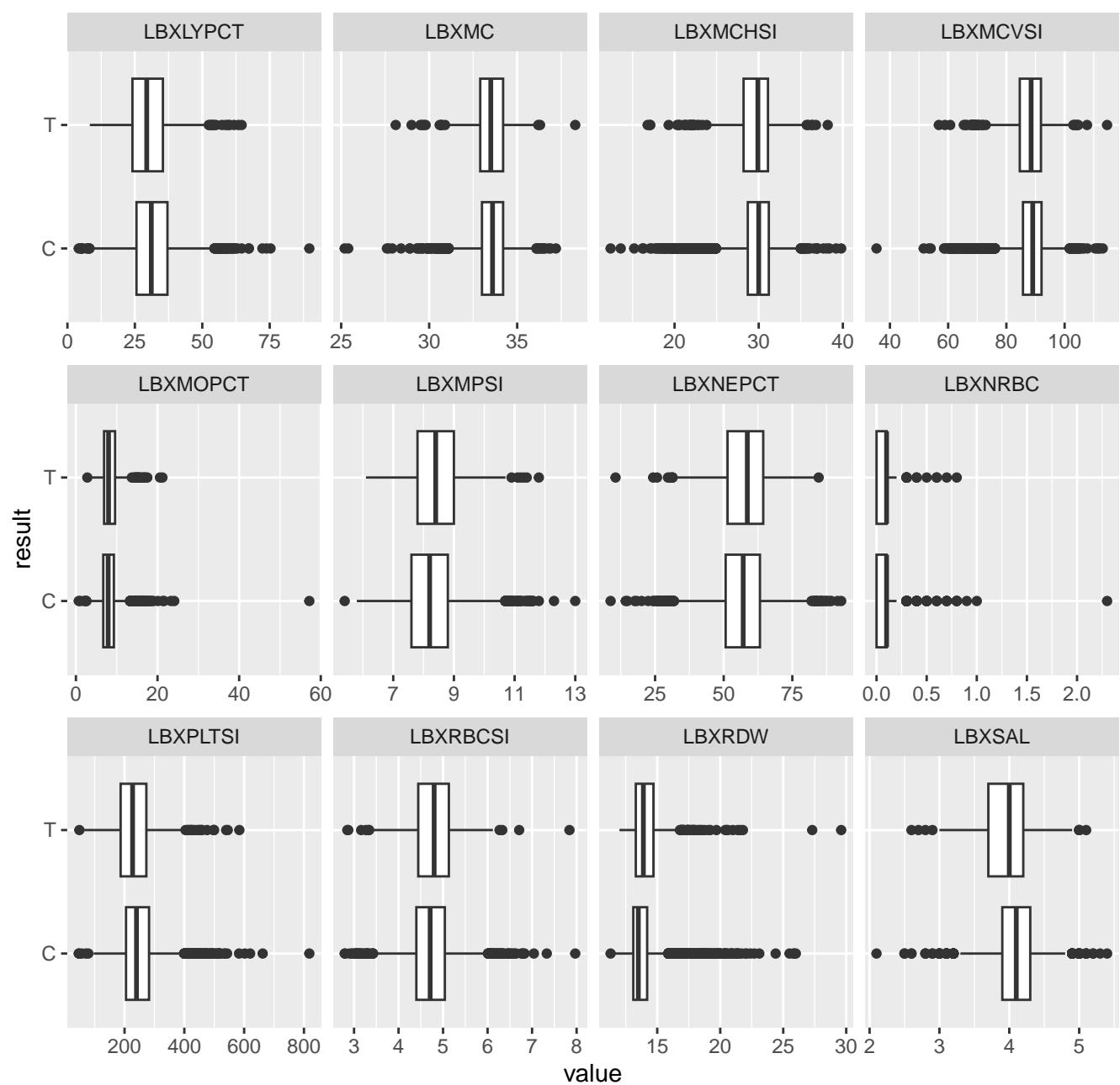

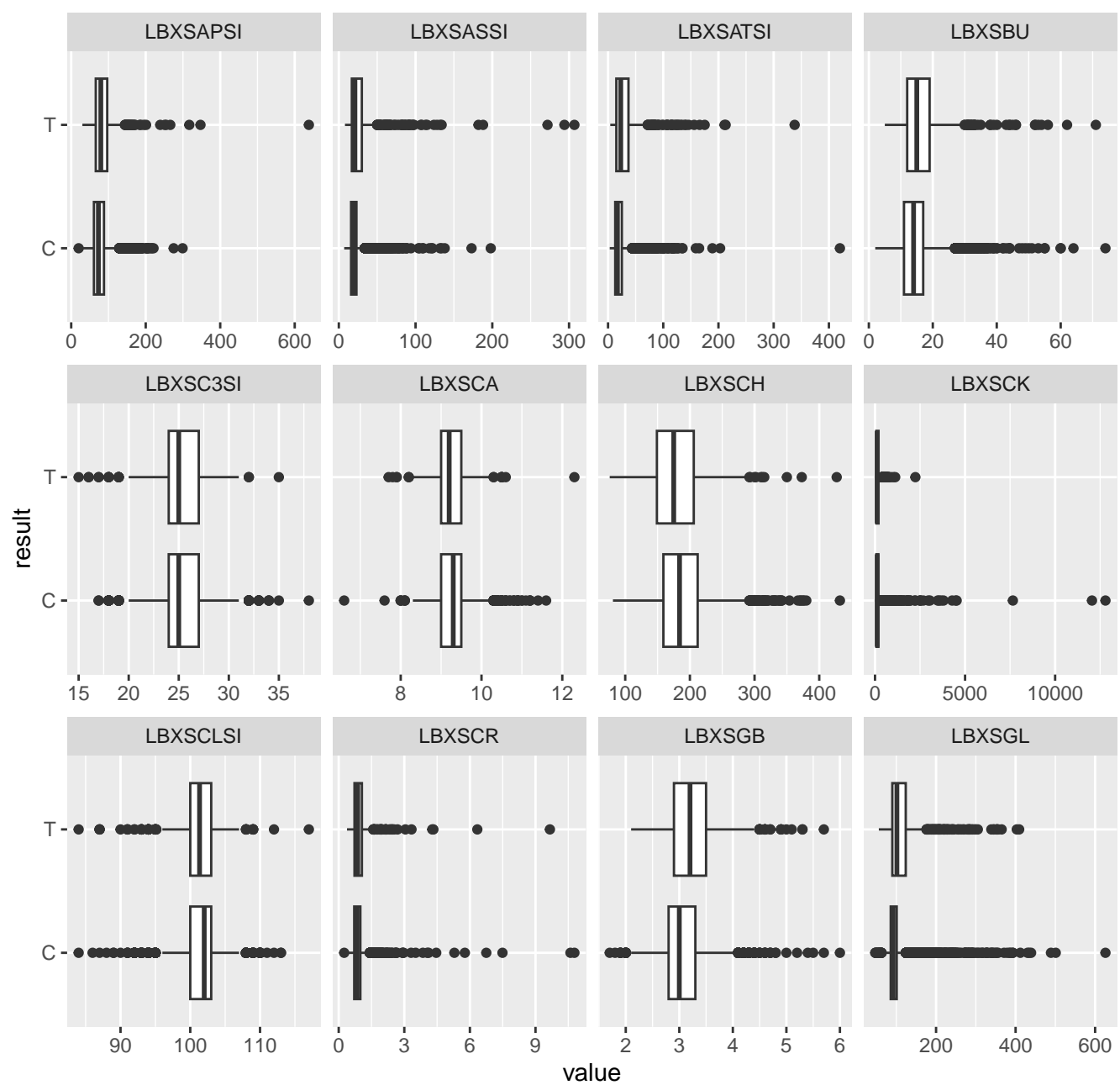

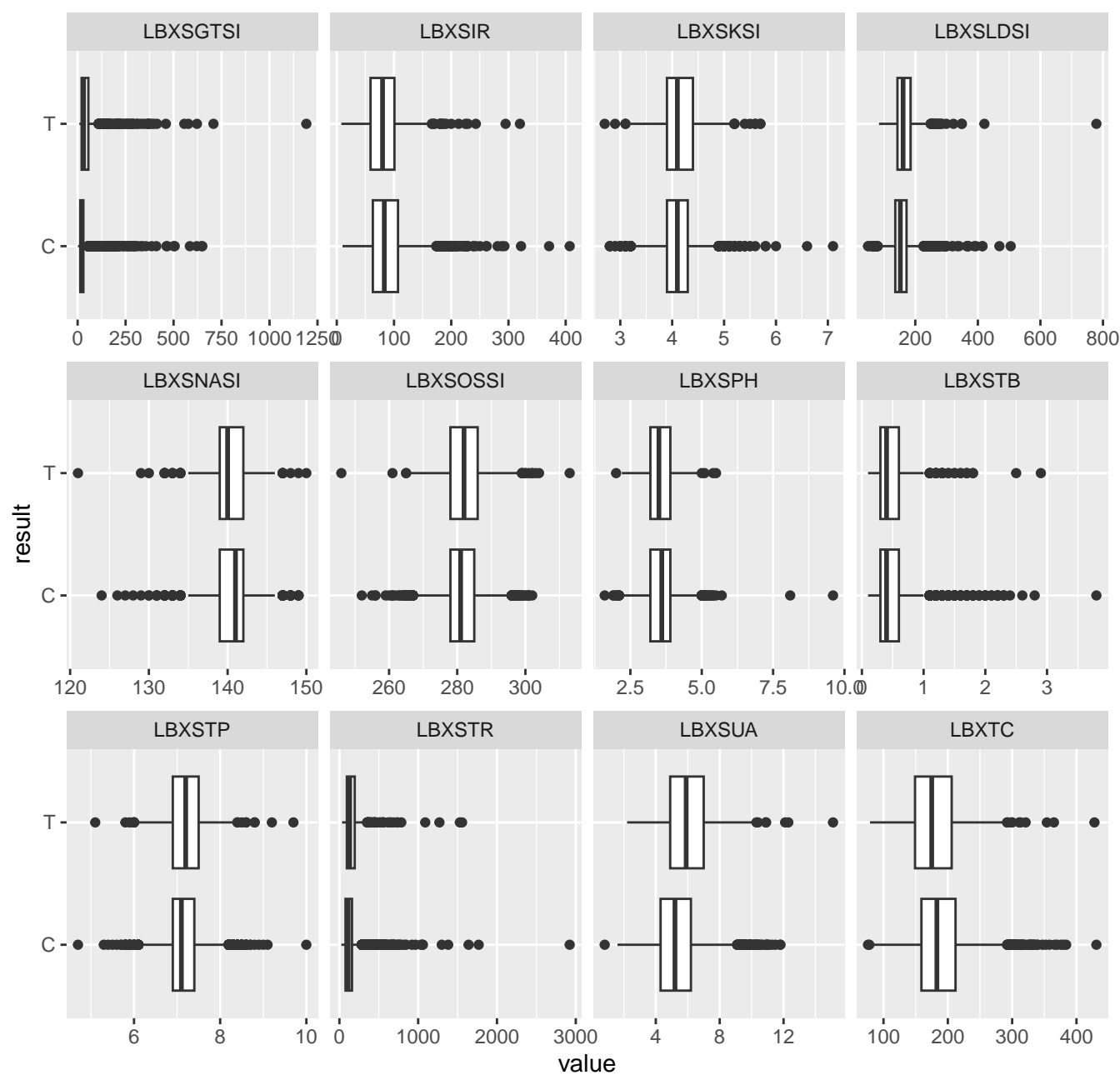

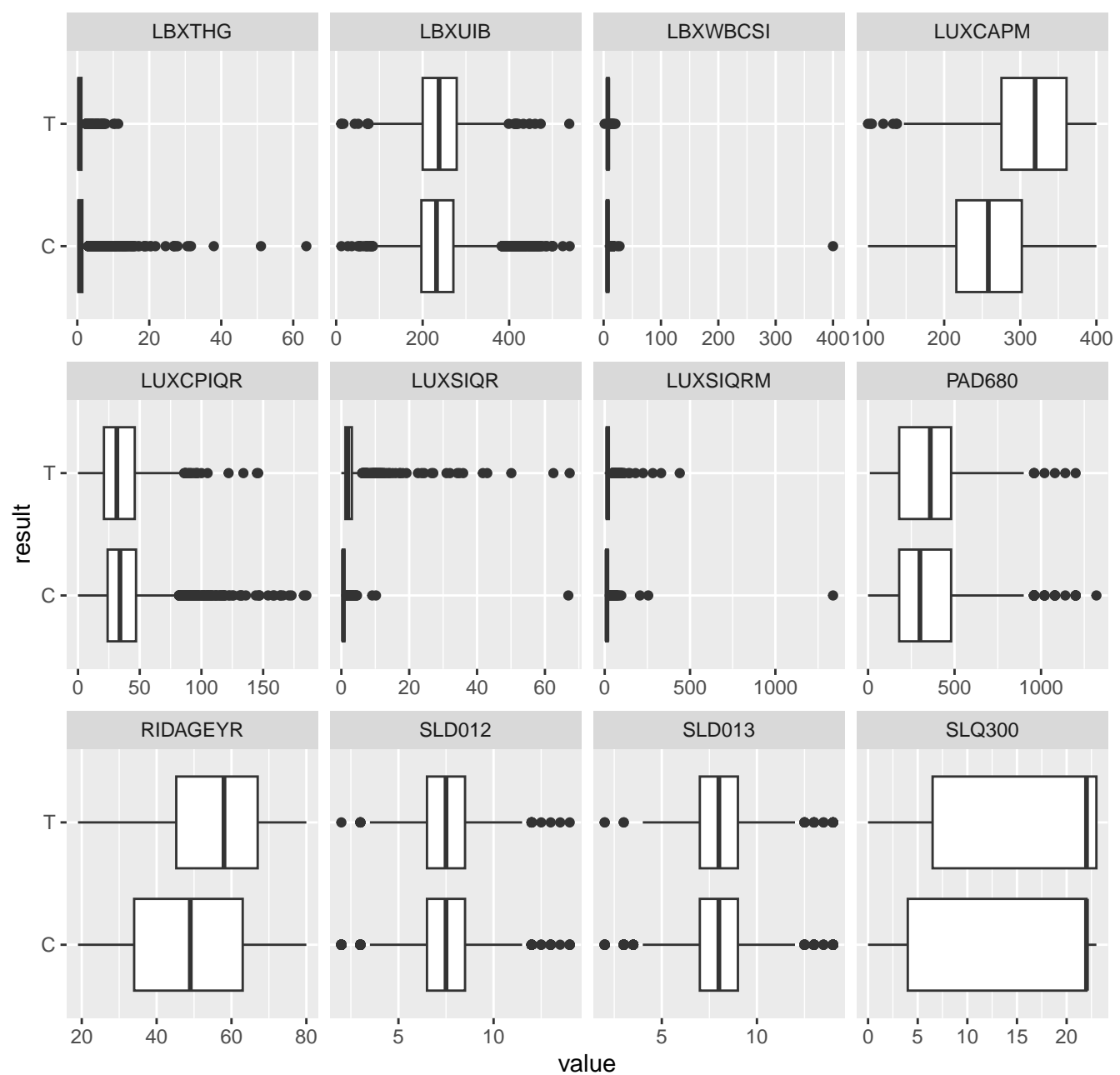

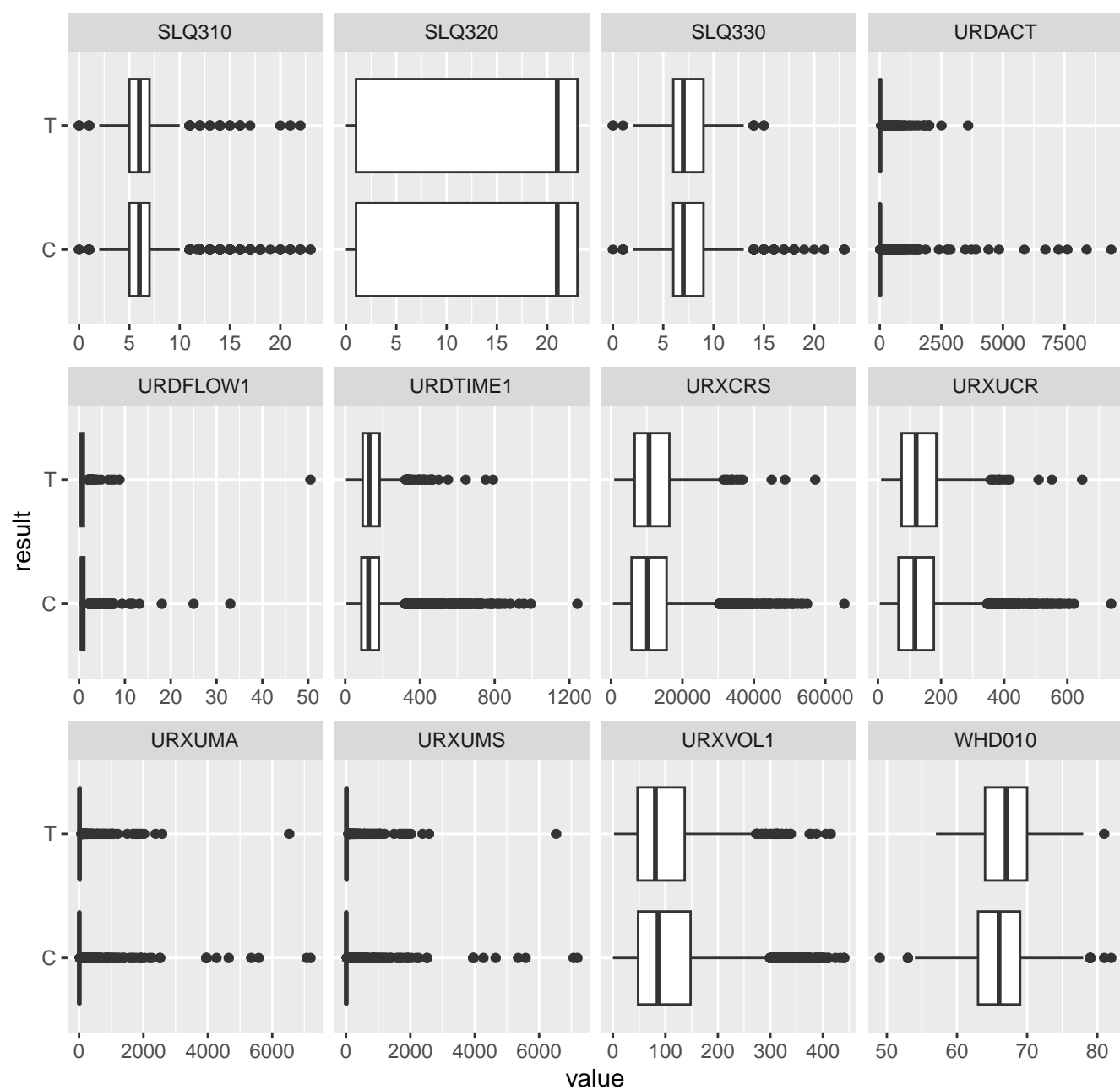

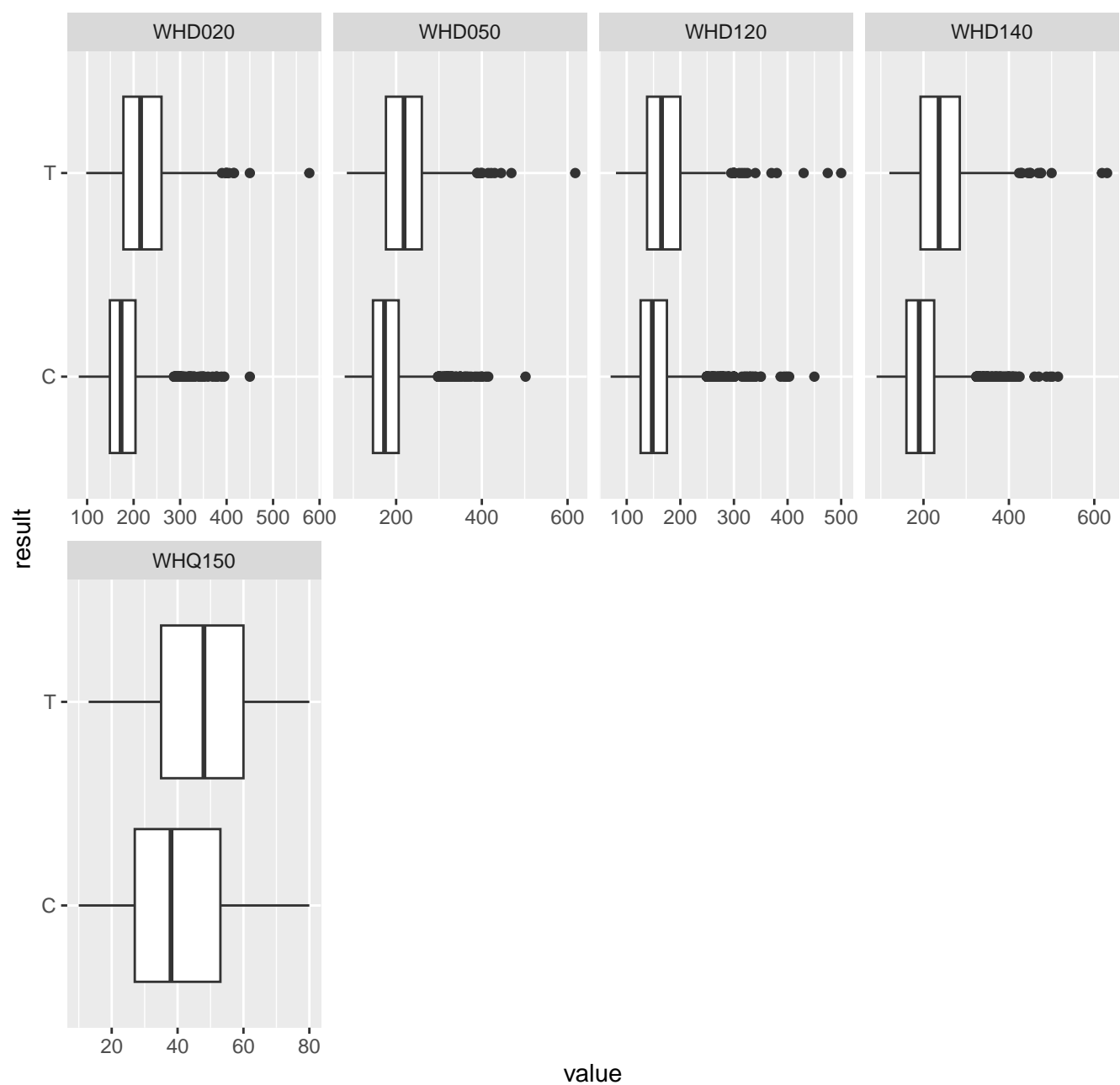

Supplement: Supplementary file 5 — This figure presents the numerical distribution characteristics of each continuous variable included in this study in the clinically significant liver fibrosis case group (T) and the control case group (C) through a collection of box plots, including the median, quartiles (25th and 75th percentiles), maximum value, minimum value, and potential outliers of the data. [file Data_Sheet_5.pdf]

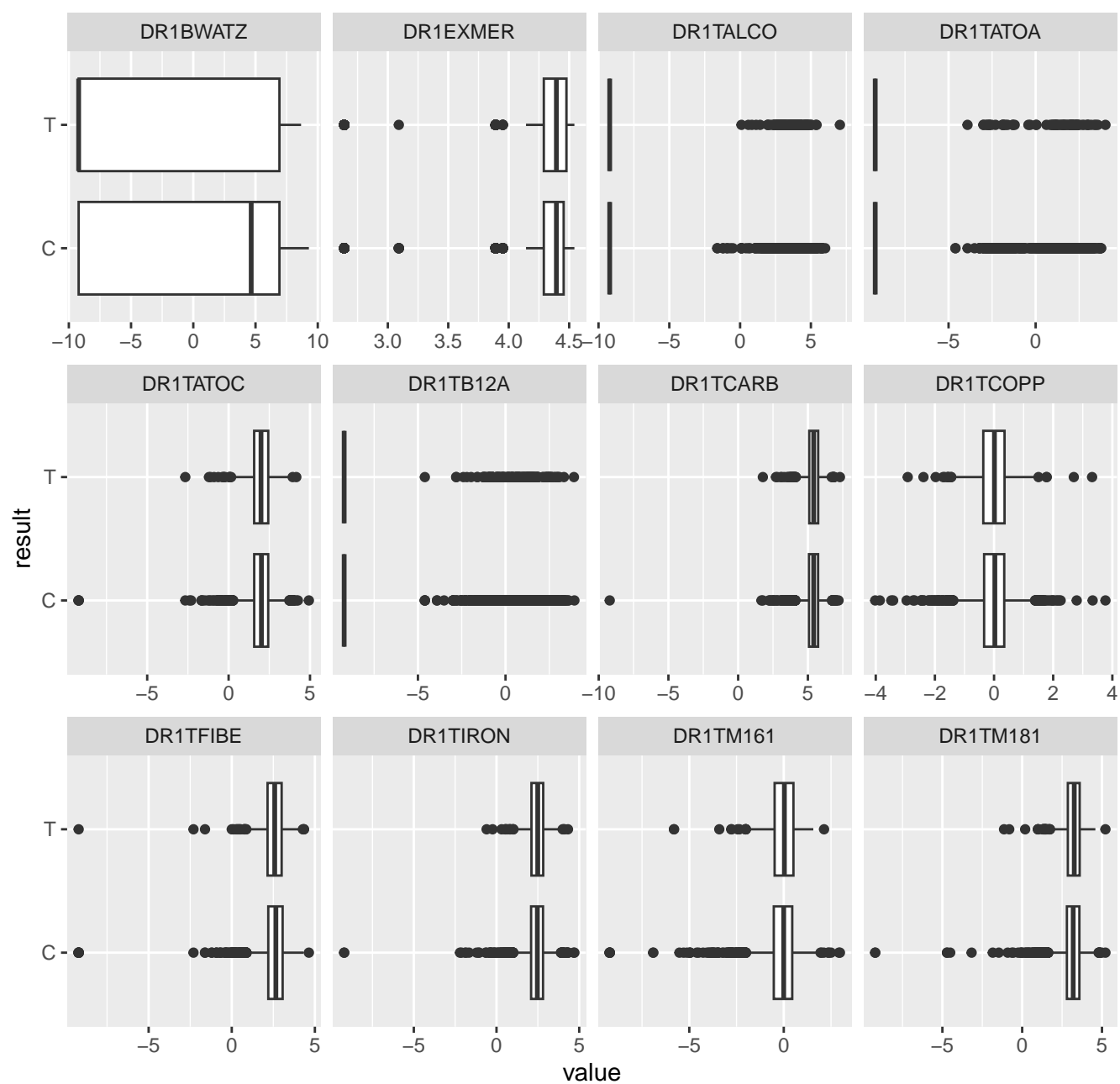

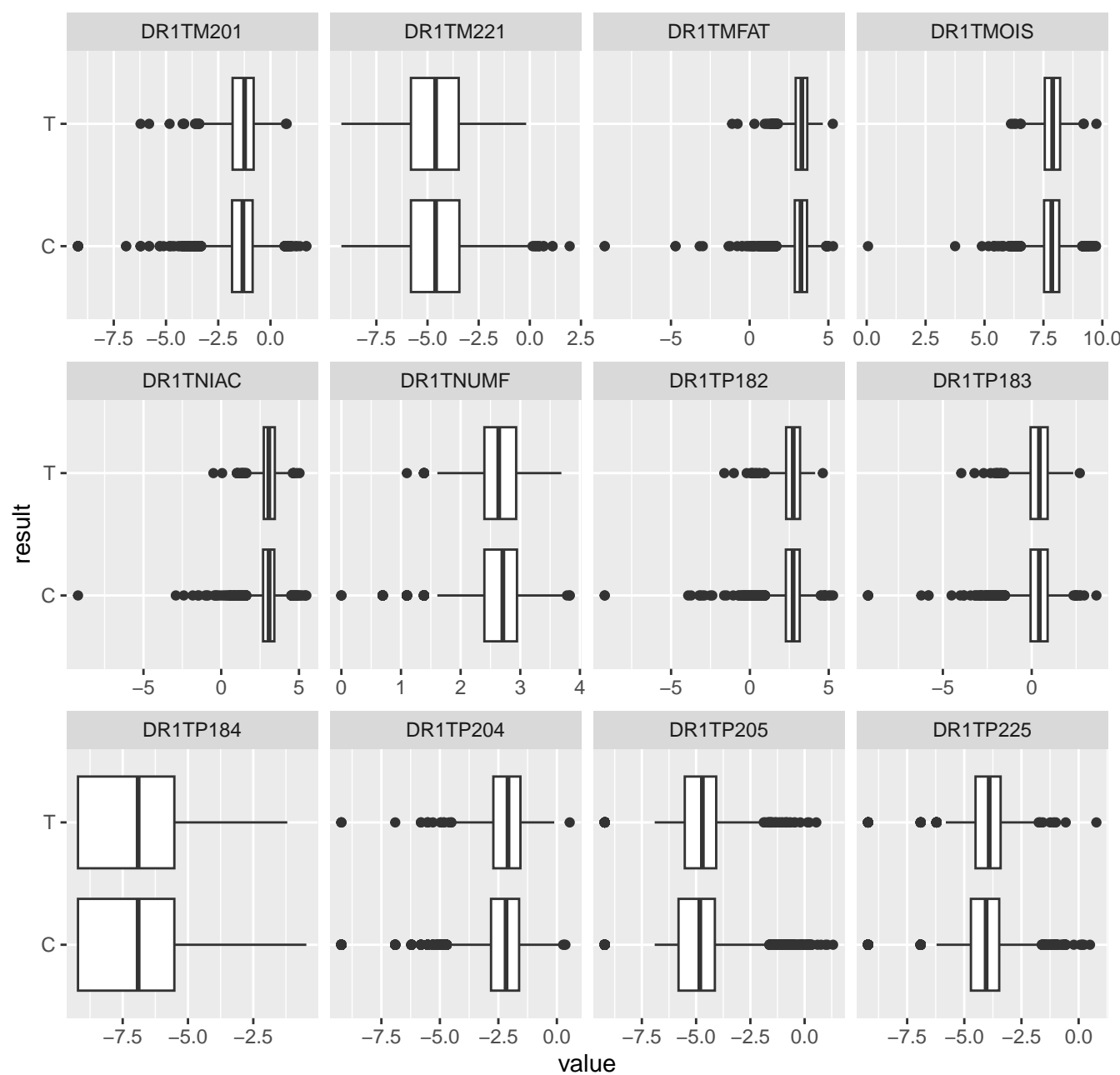

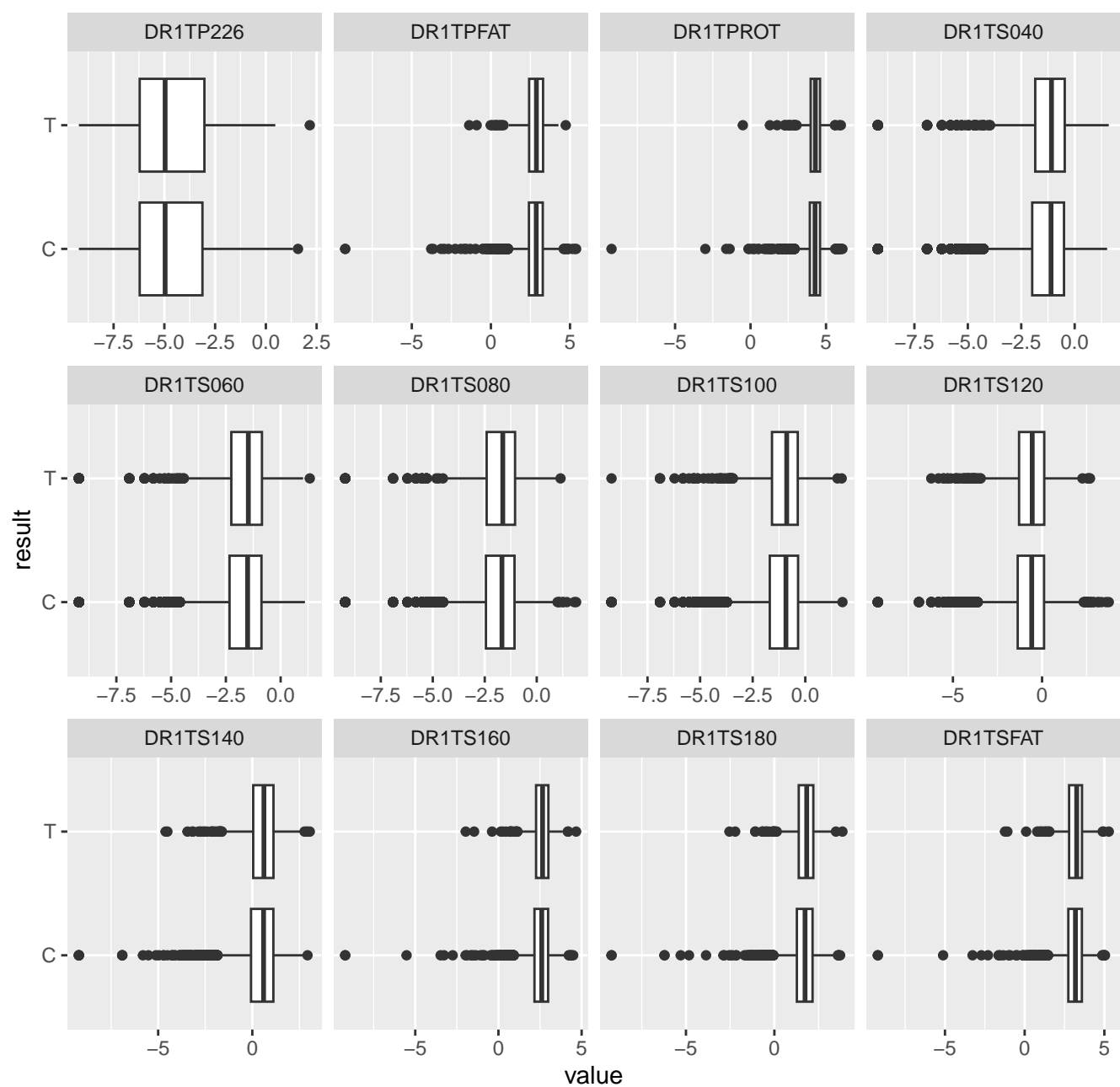

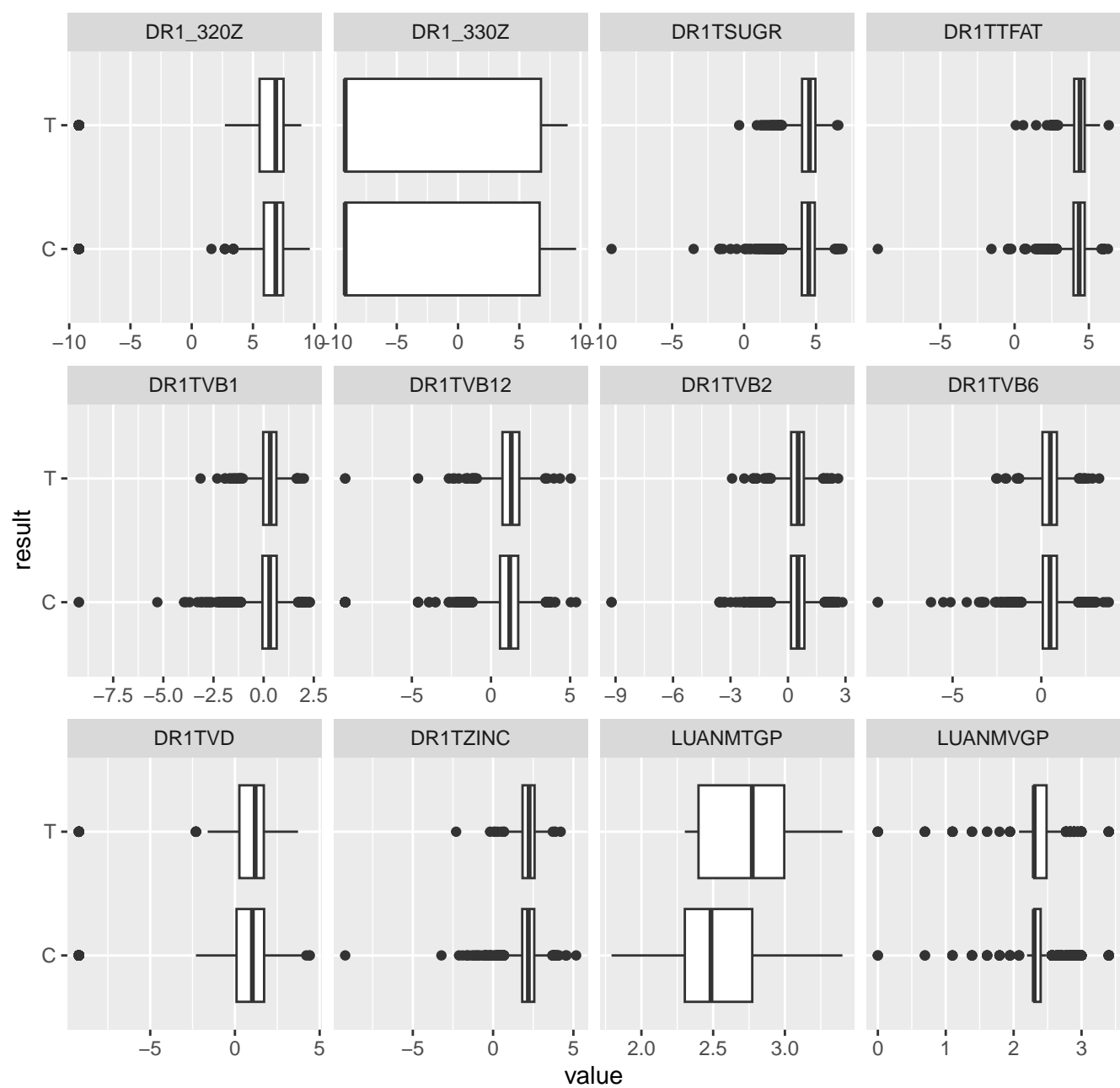

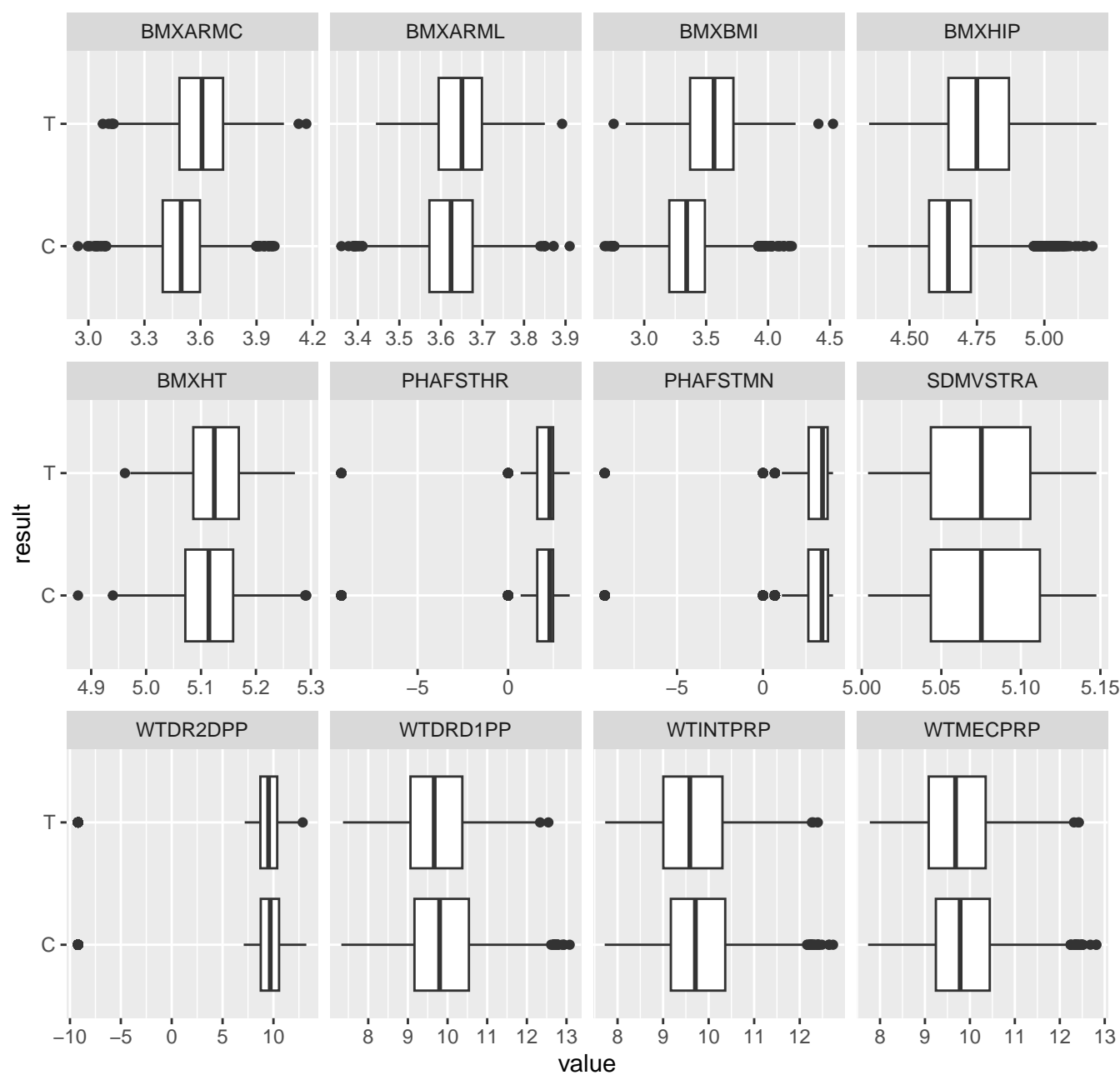

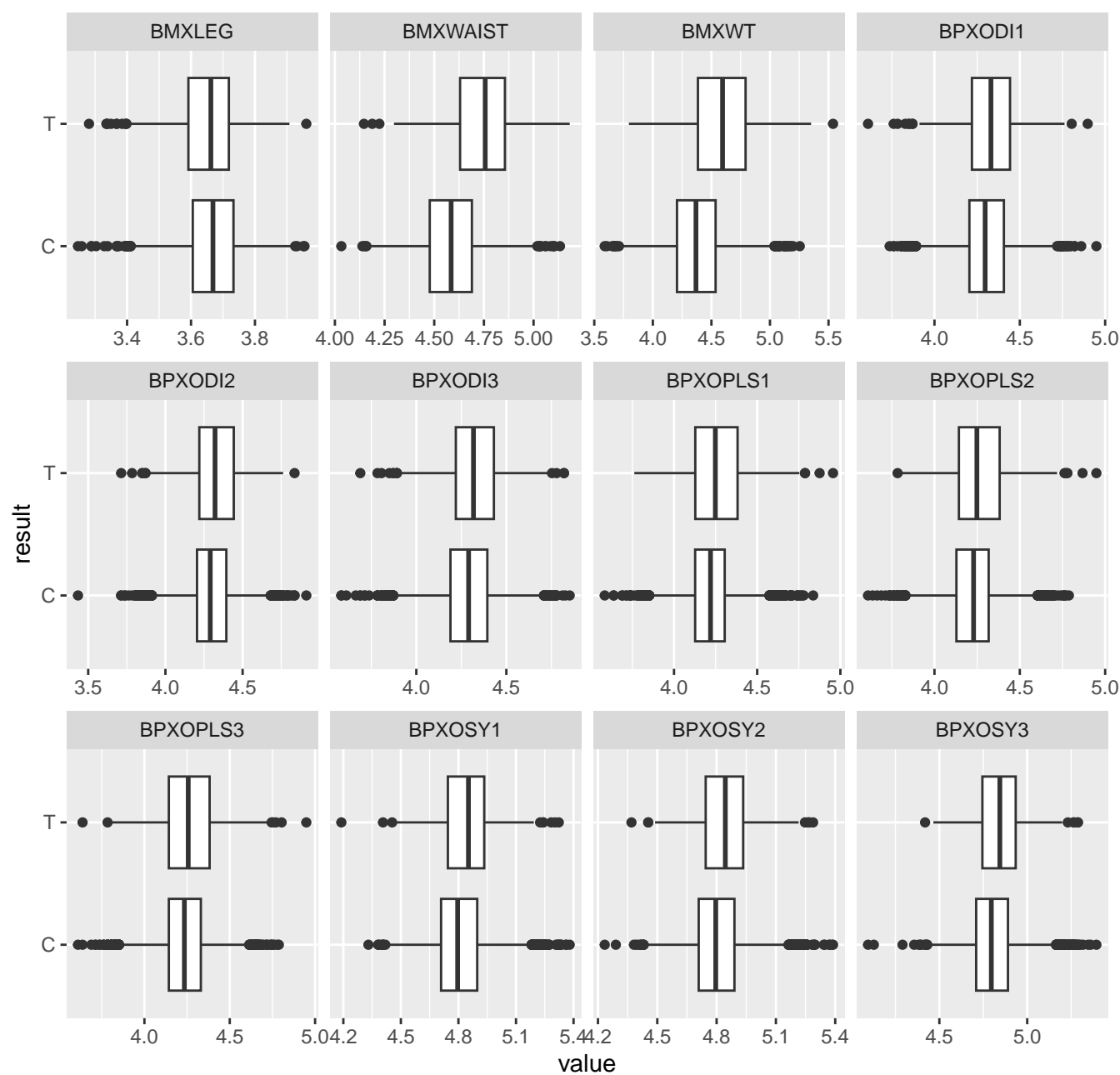

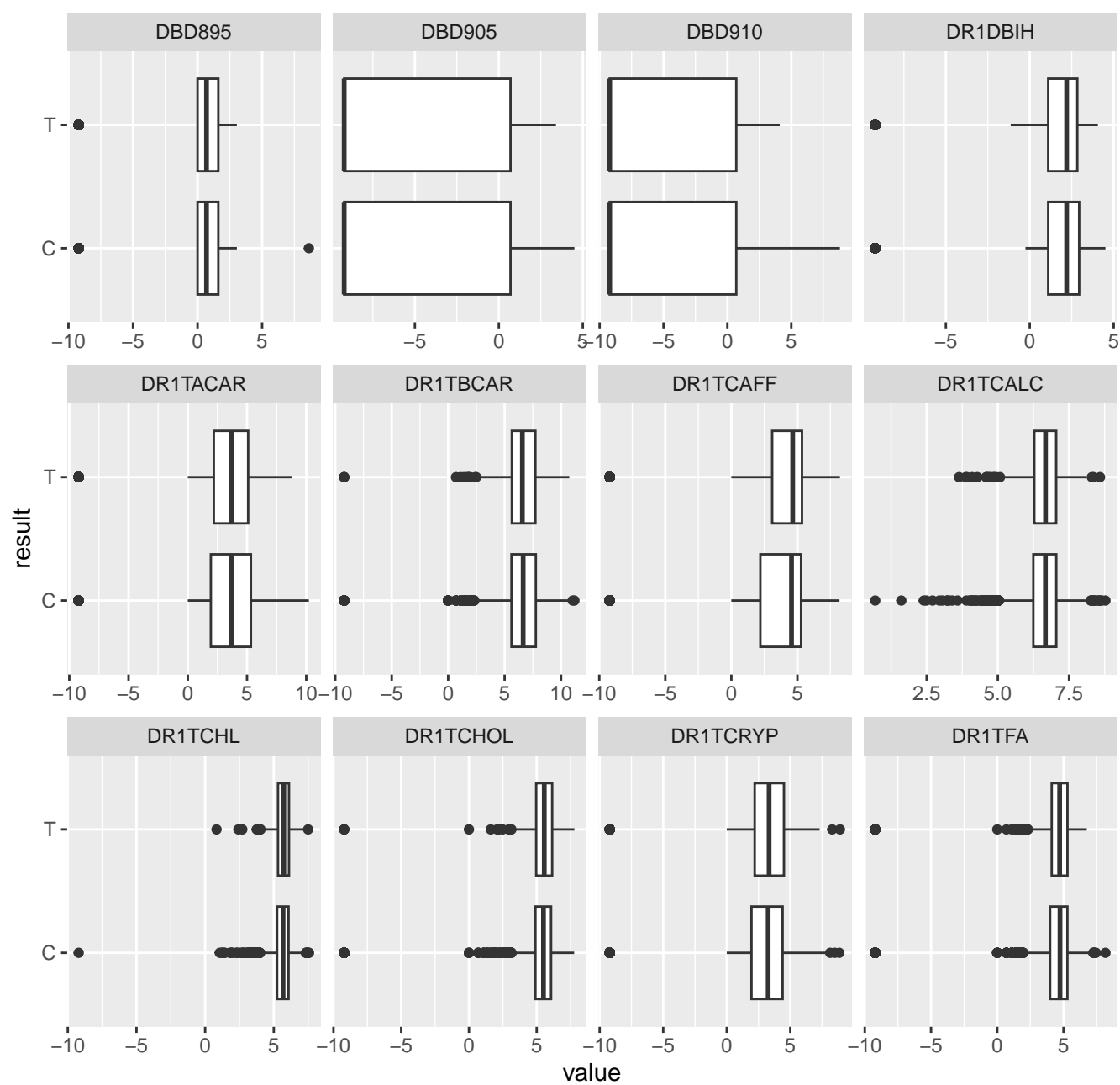

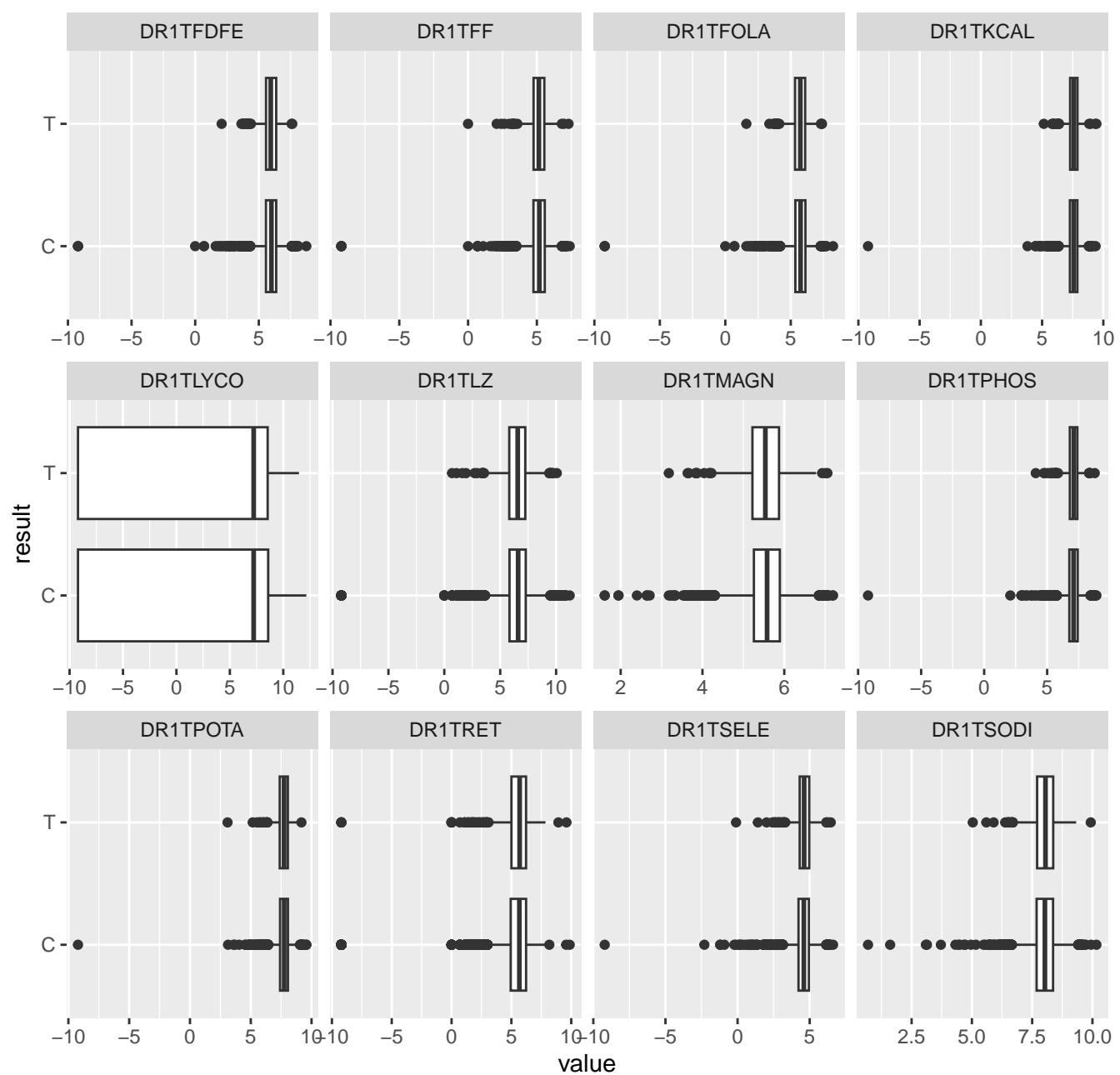

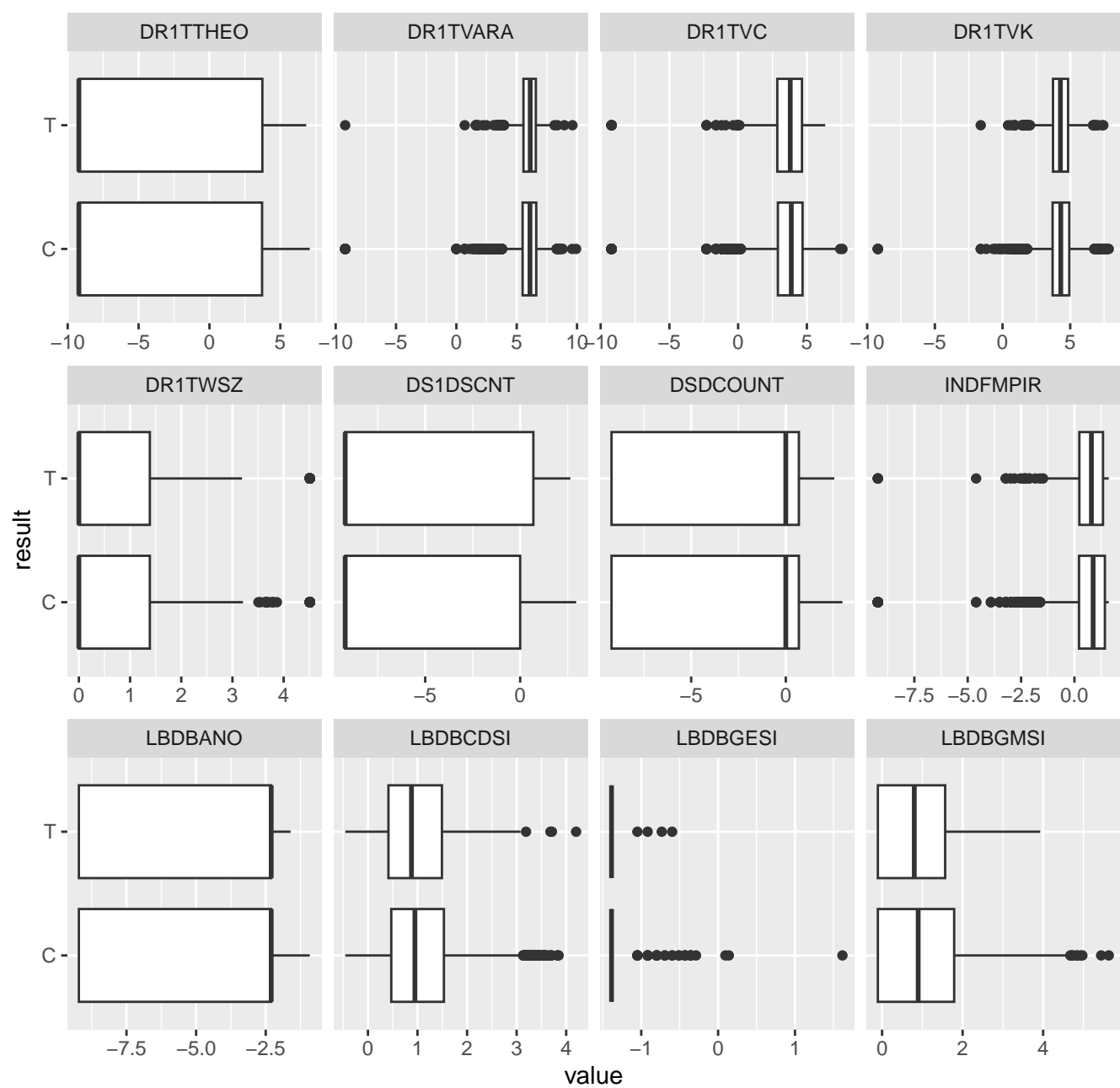

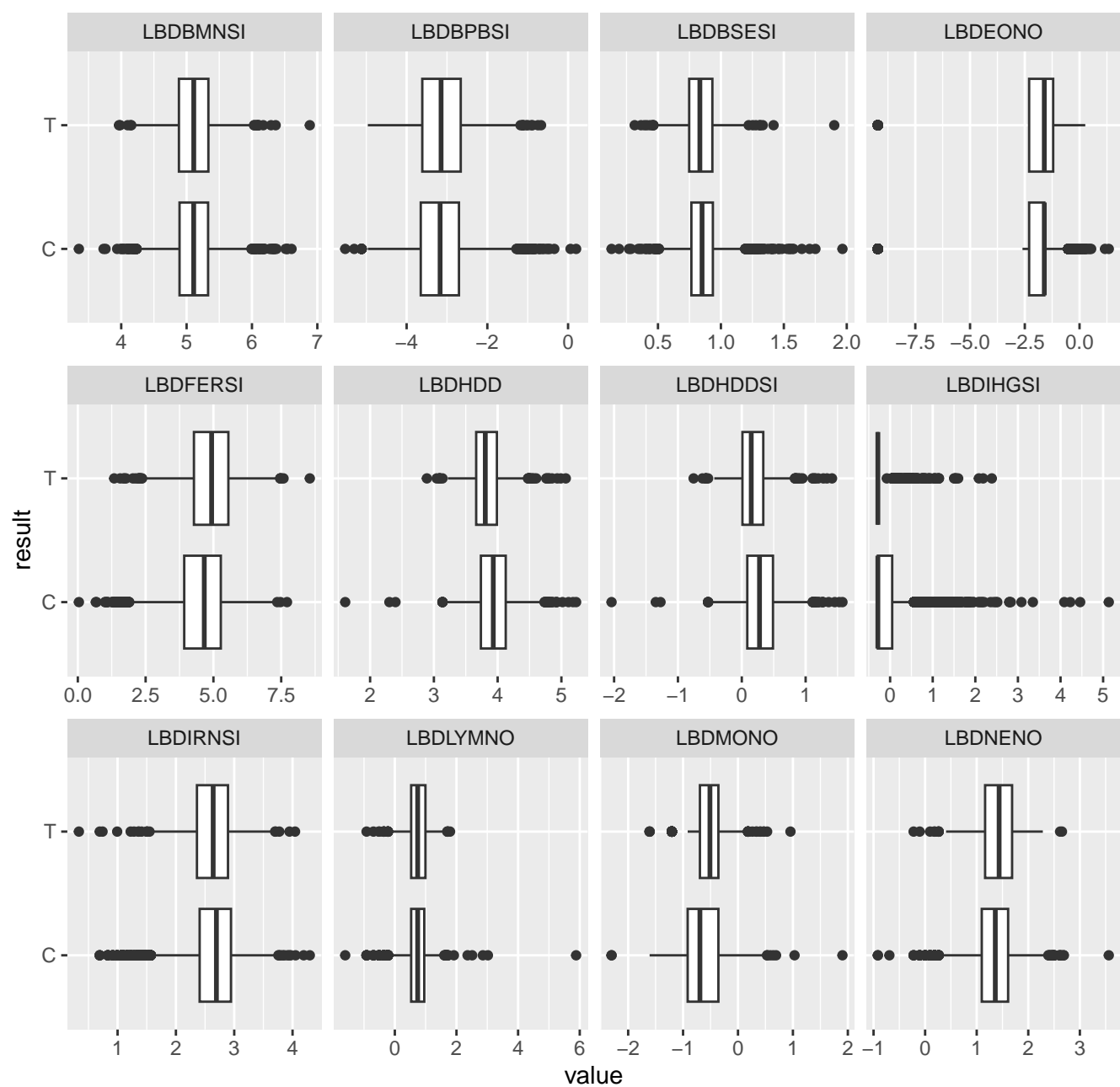

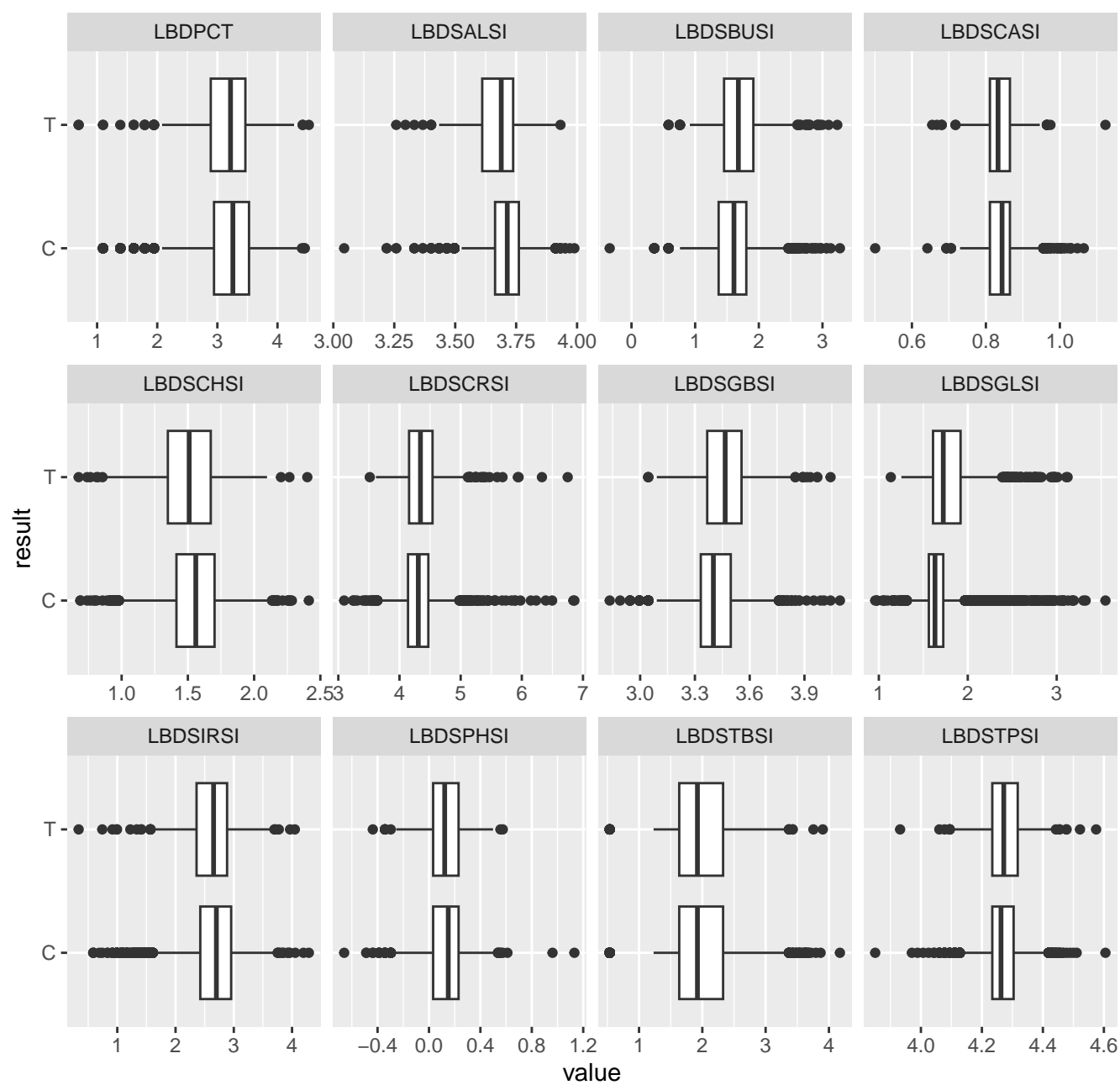

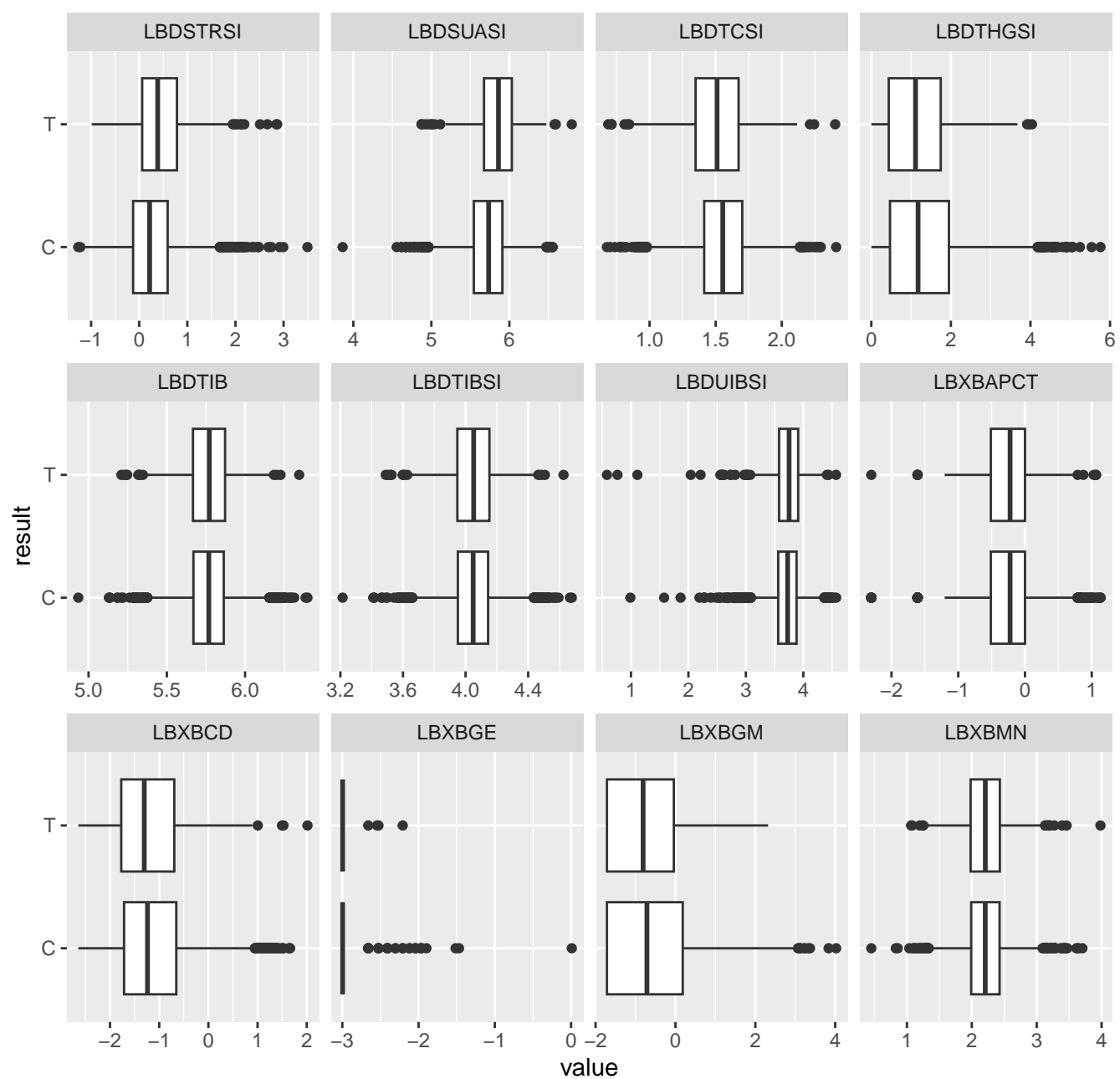

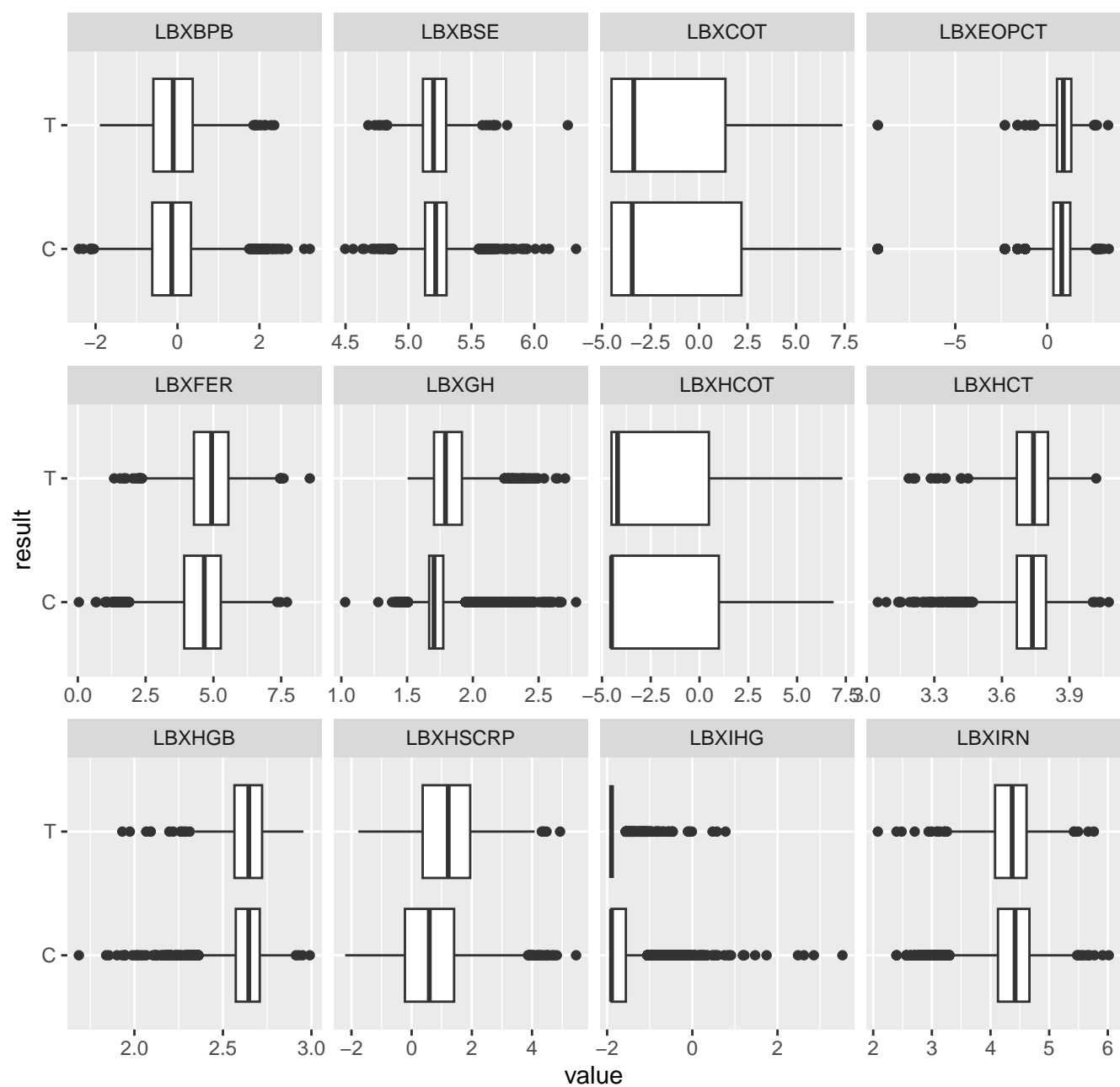

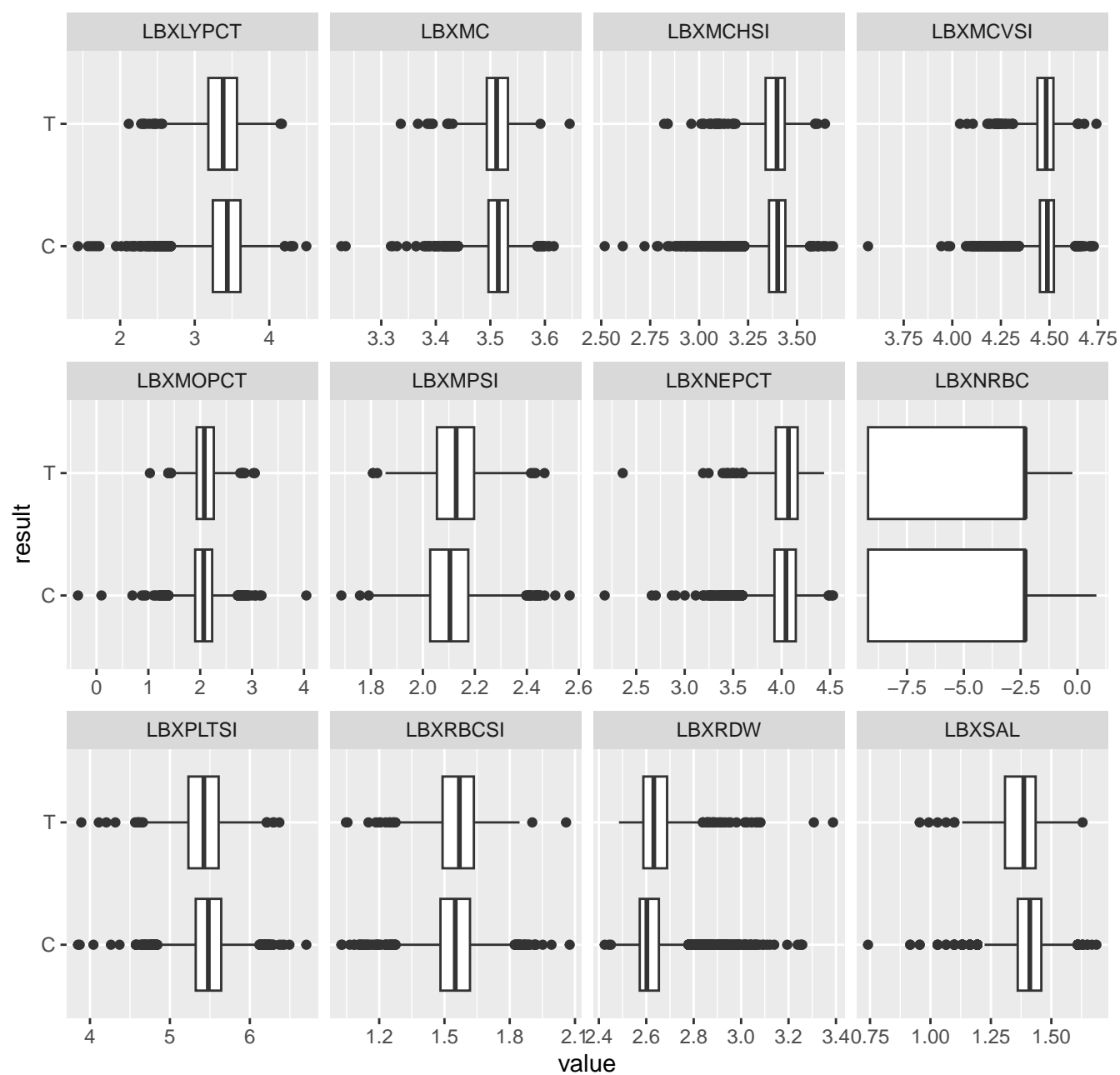

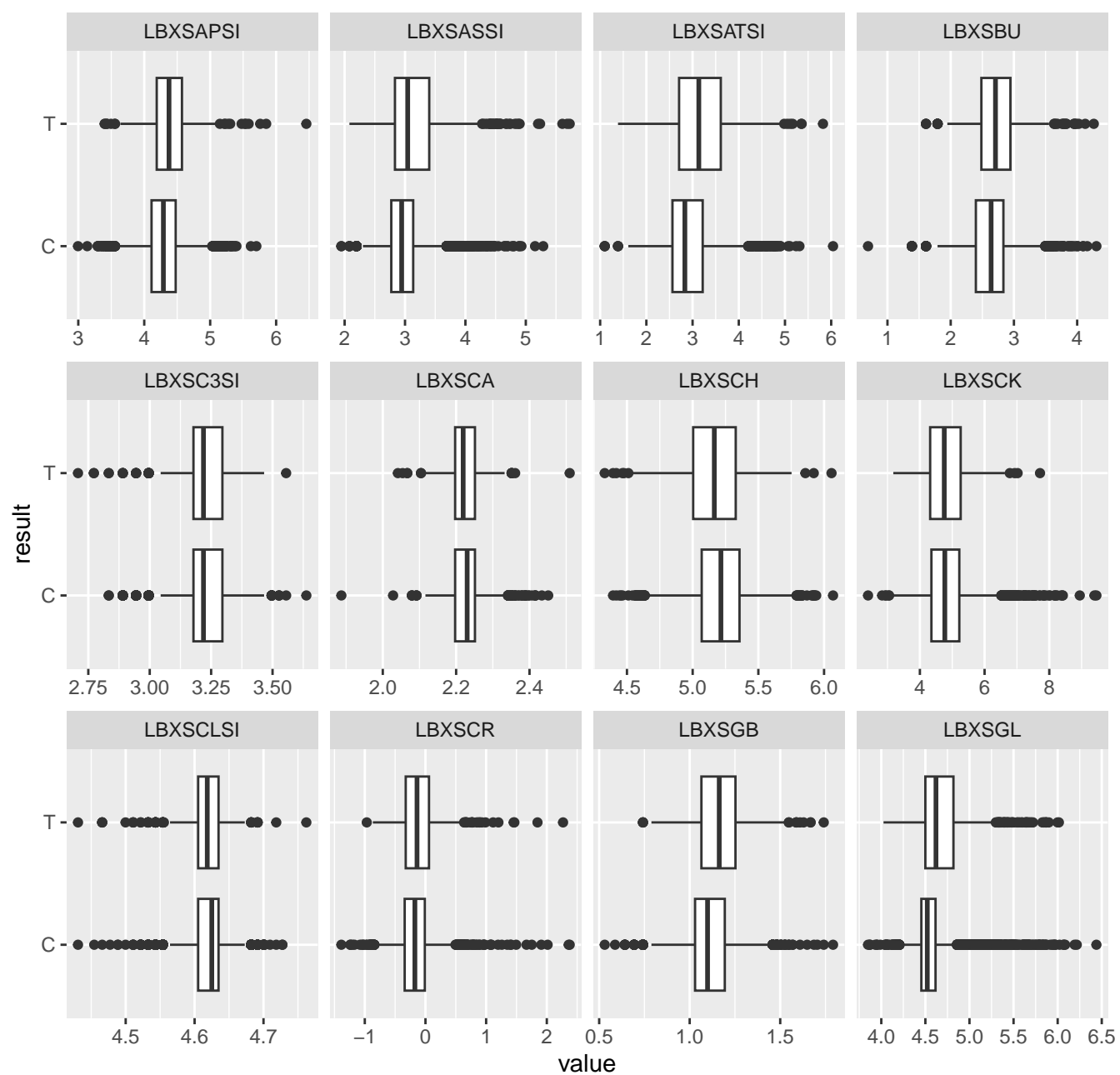

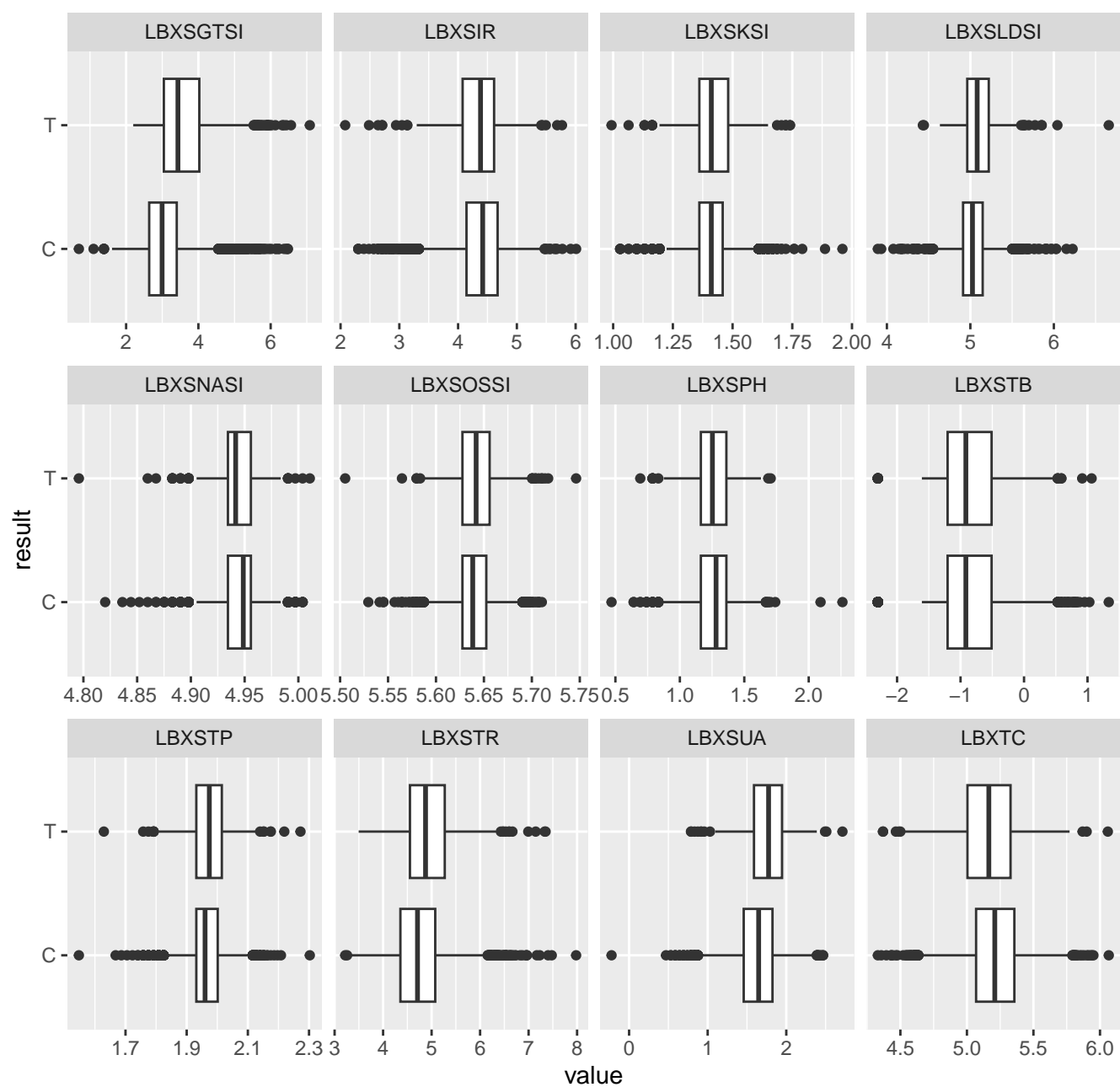

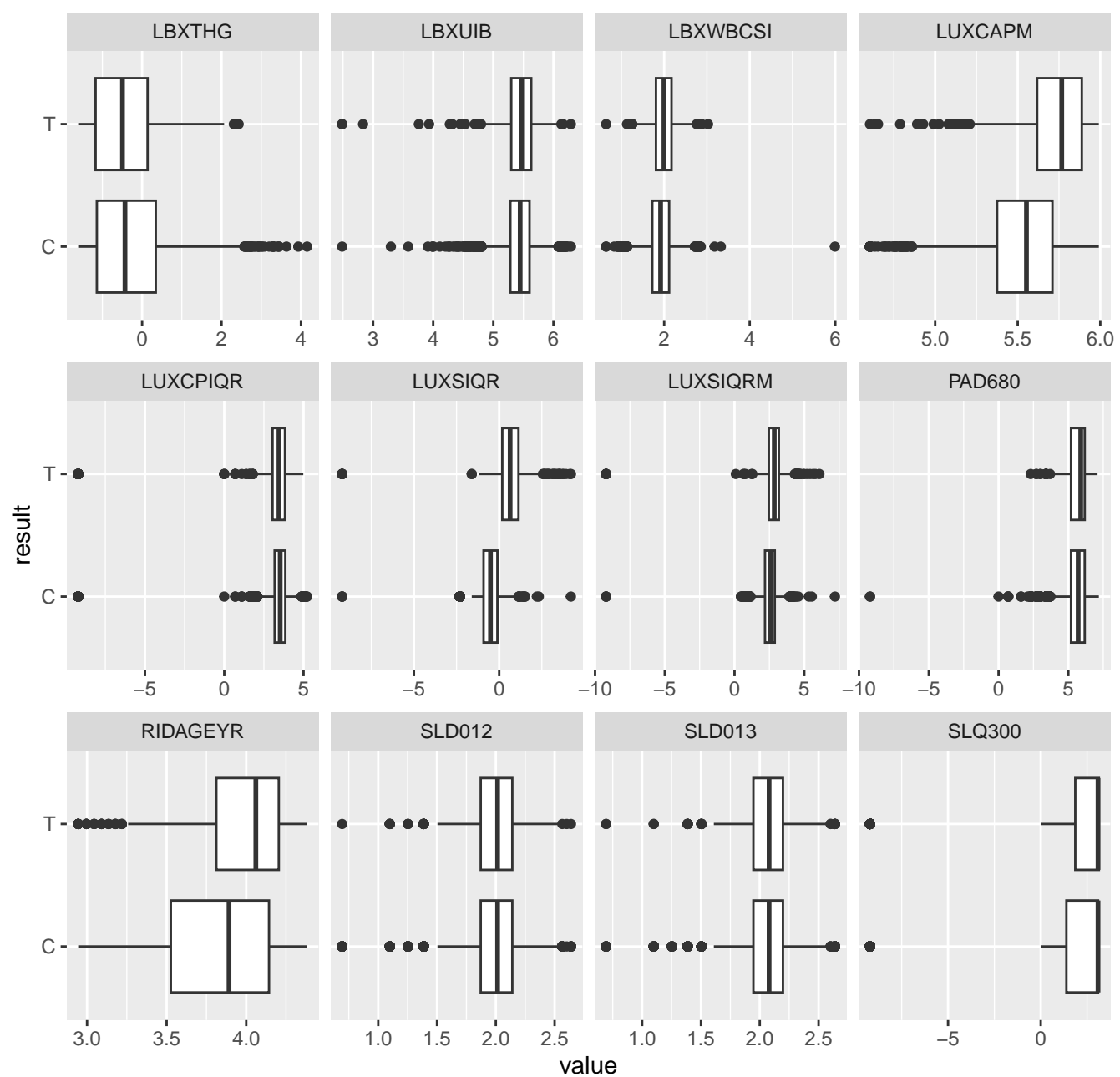

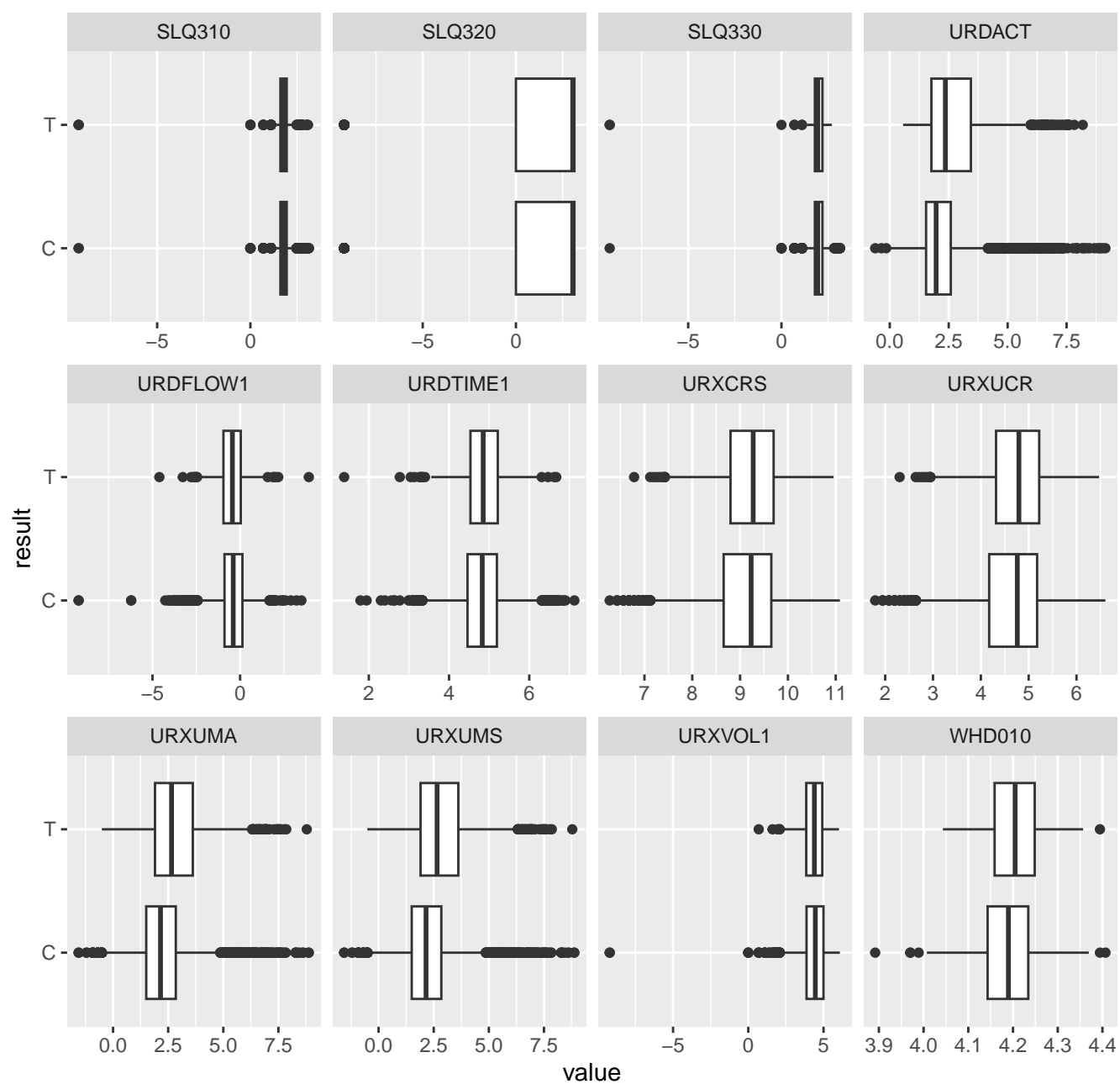

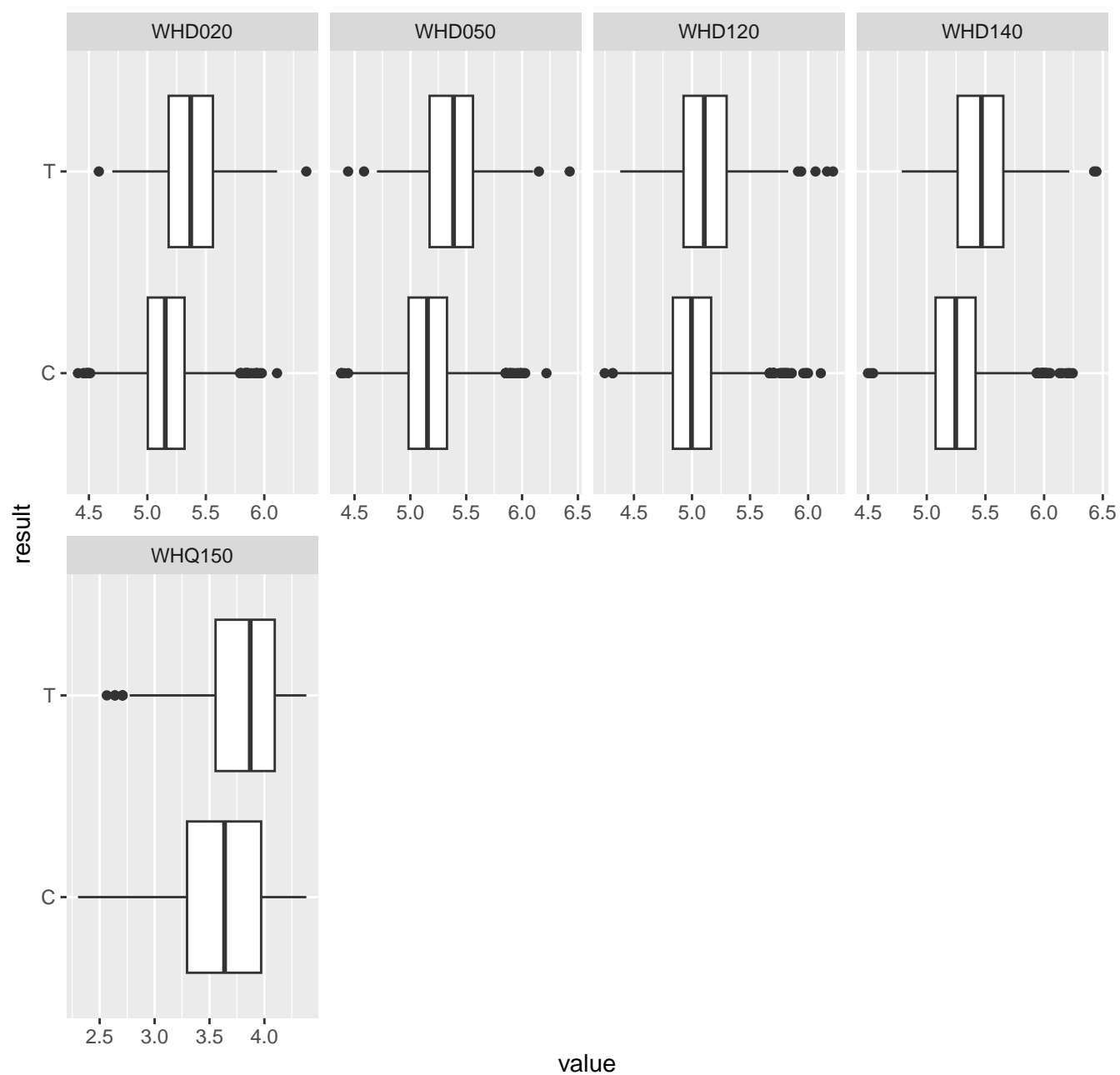

Supplement: Supplementary file 6 — This document presents a series of box plots derived from the logarithmic transformation results of the original continuous variables in “Supplementary file 5”. Each subplot corresponds to one indicator, and by comparing the box plots of the control group (C) and the clinically significant liver fibrosis case group (T), it intuitively displays the median, quartiles, and distribution range of the transformed data. [file Data_Sheet_6.pdf]

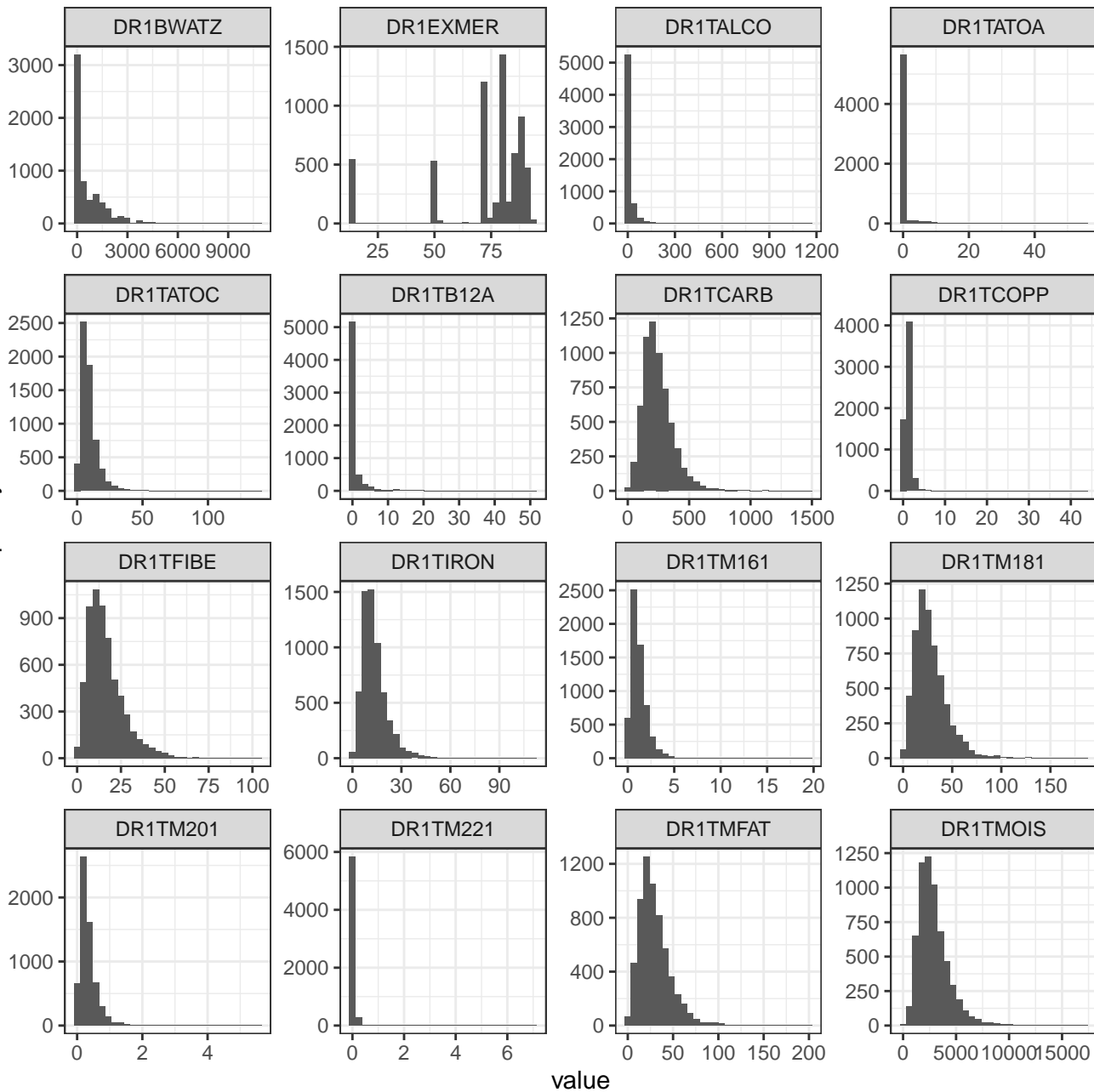

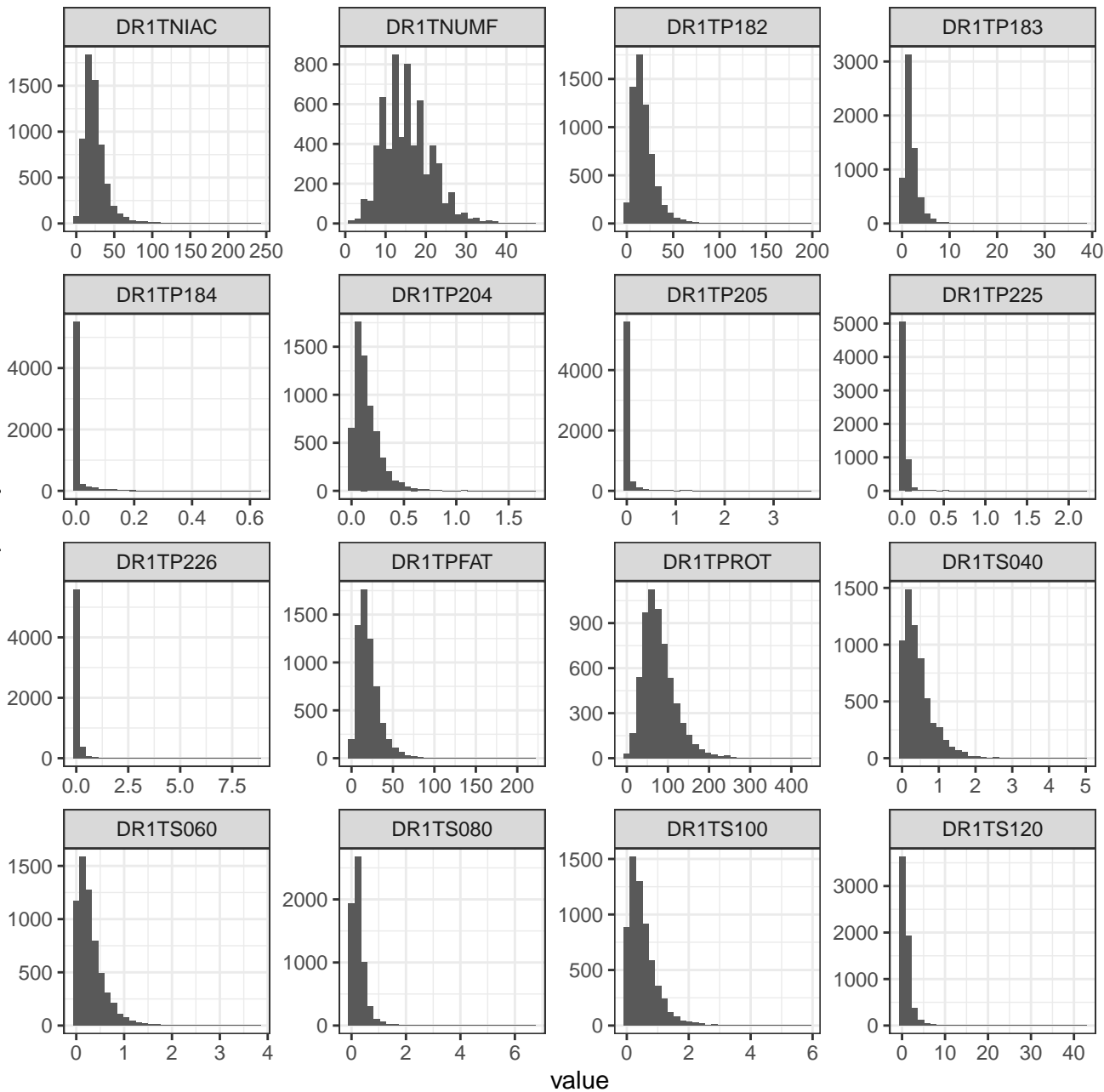

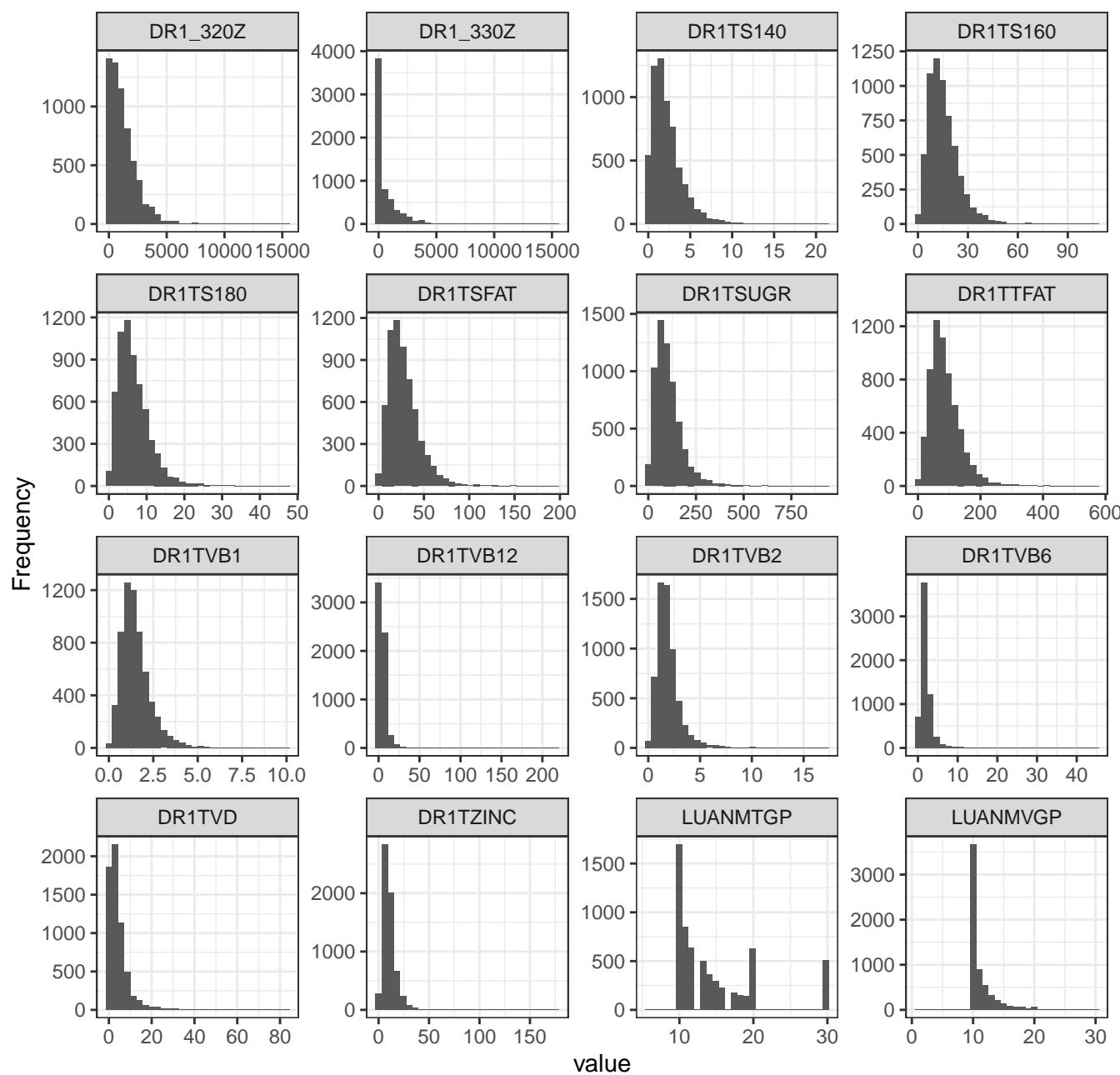

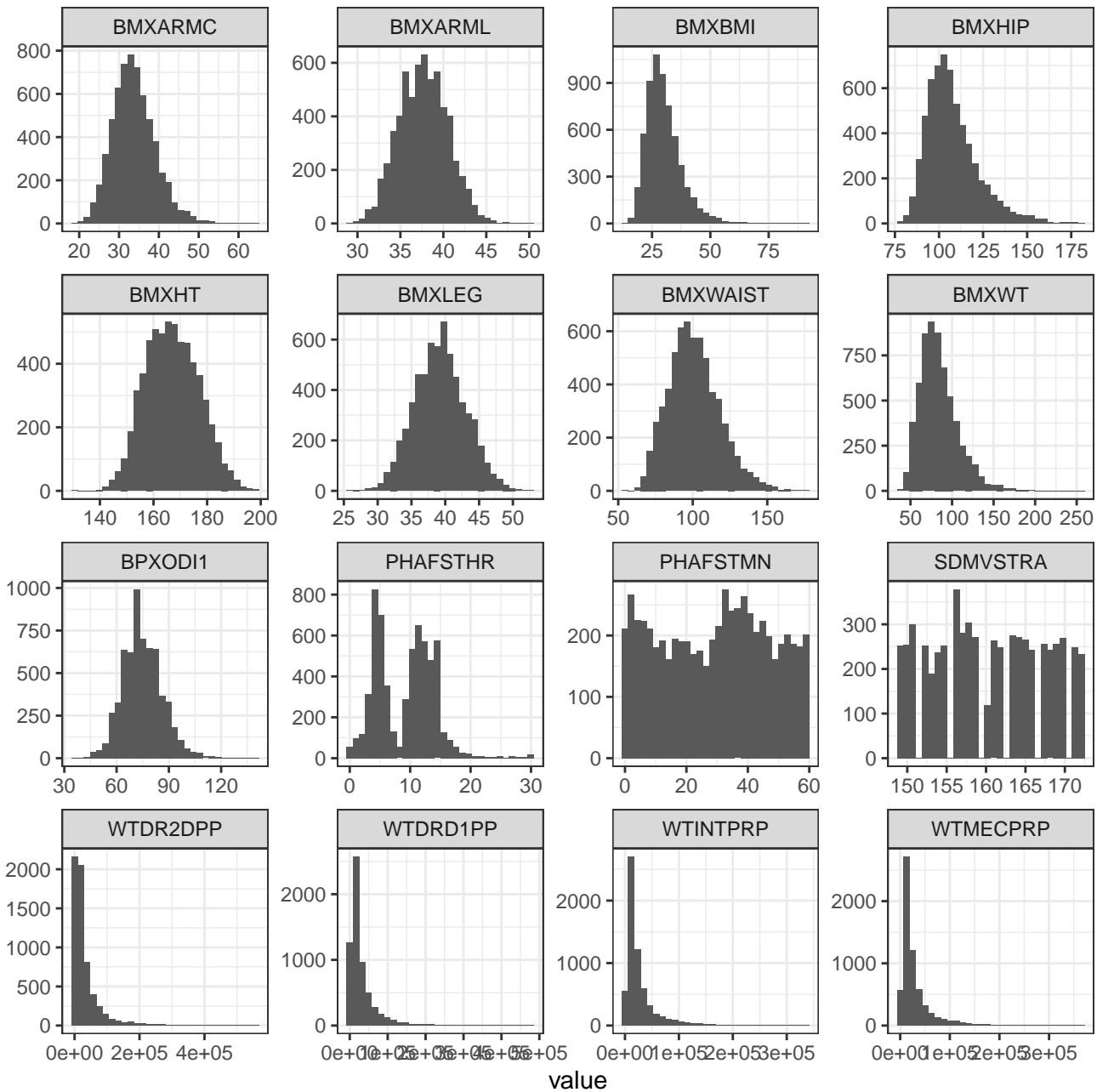

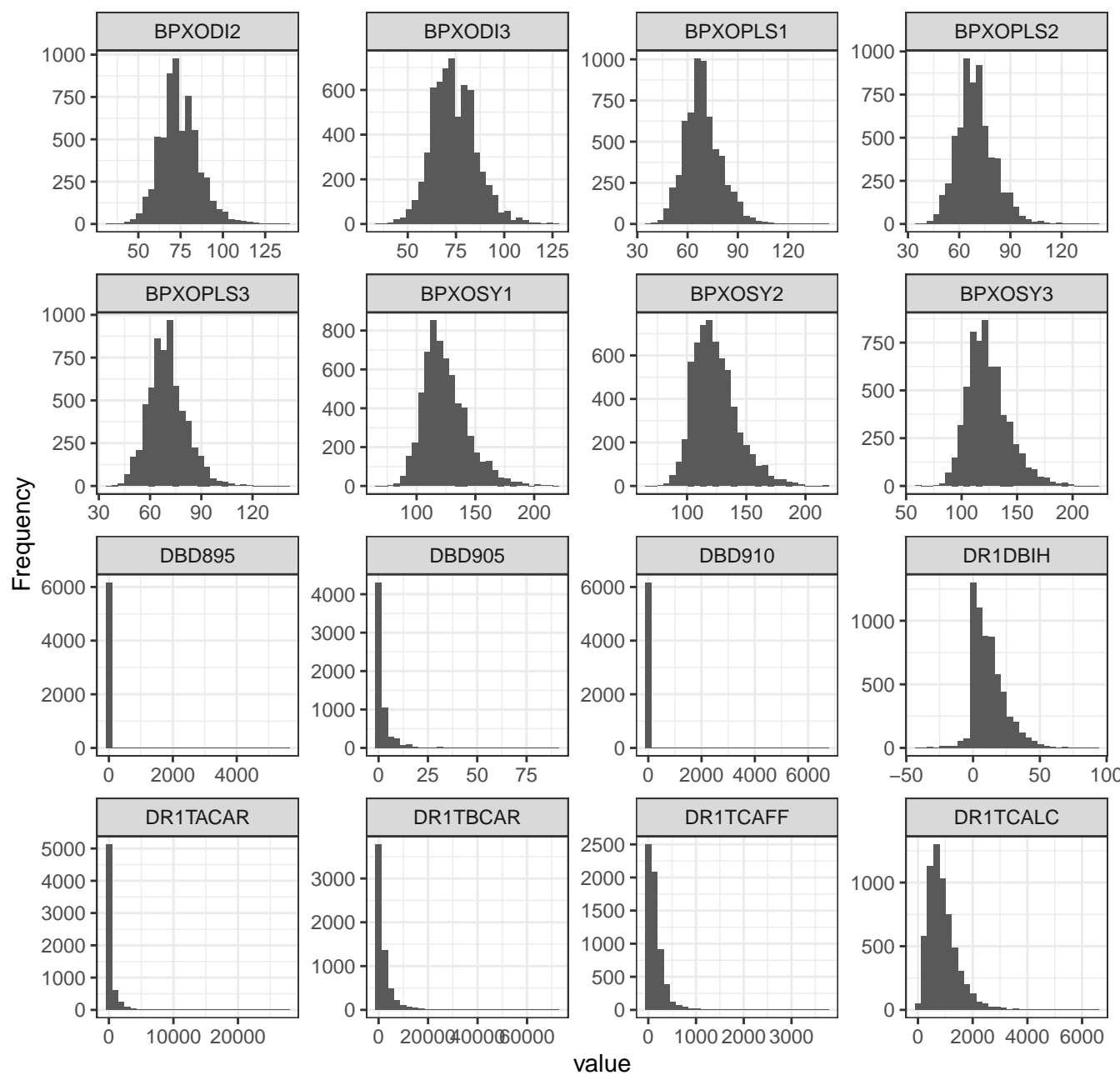

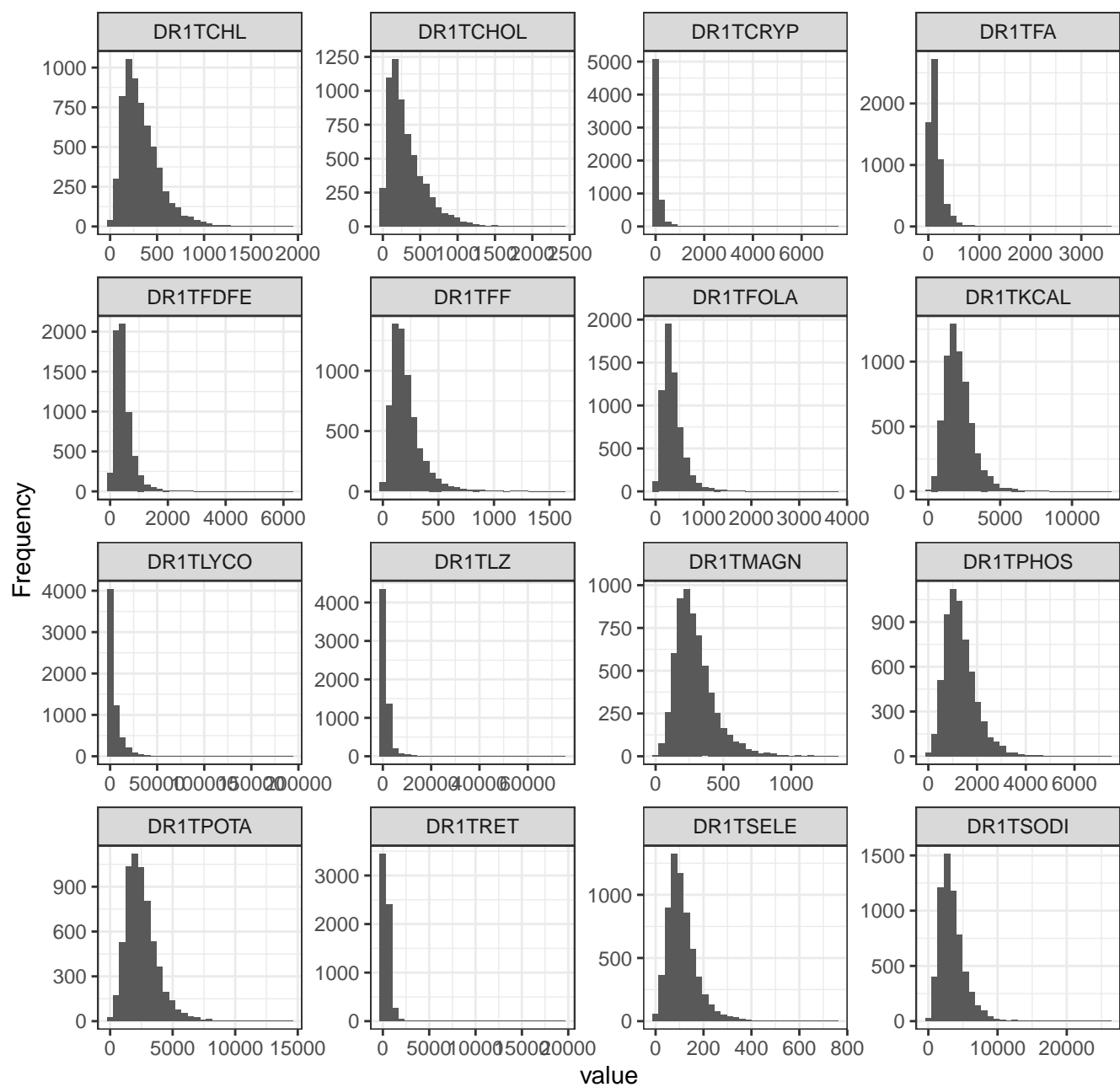

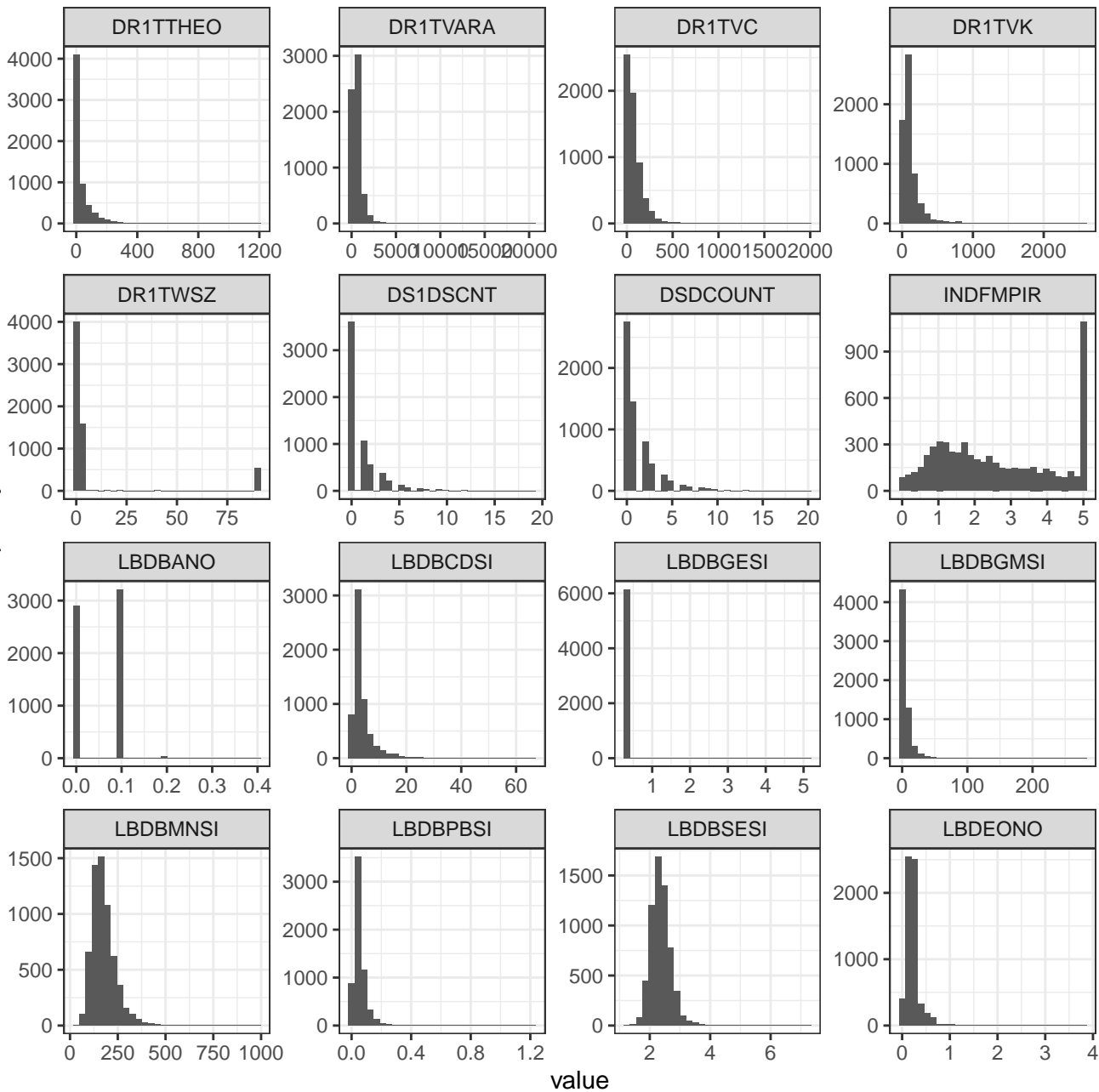

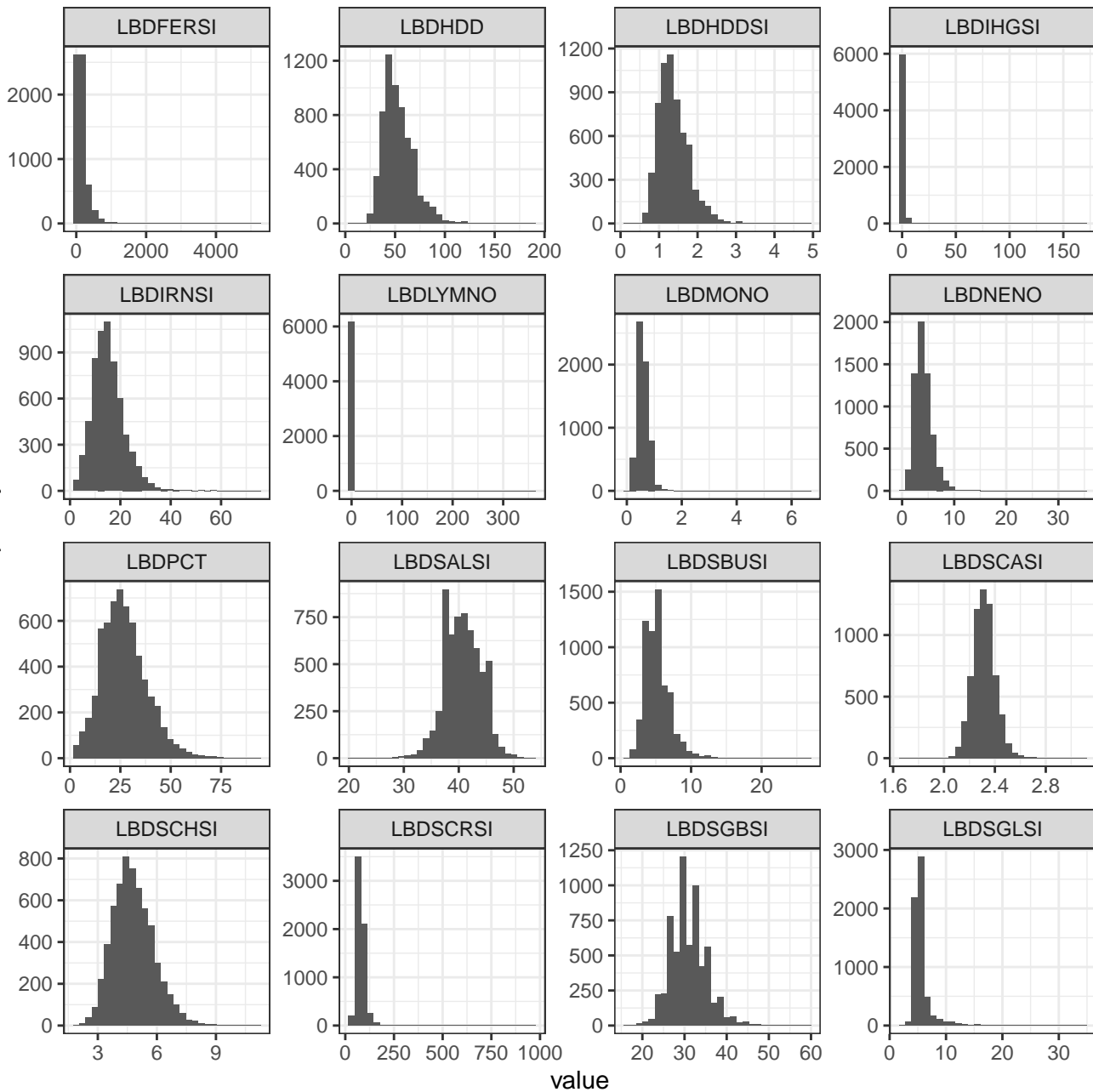

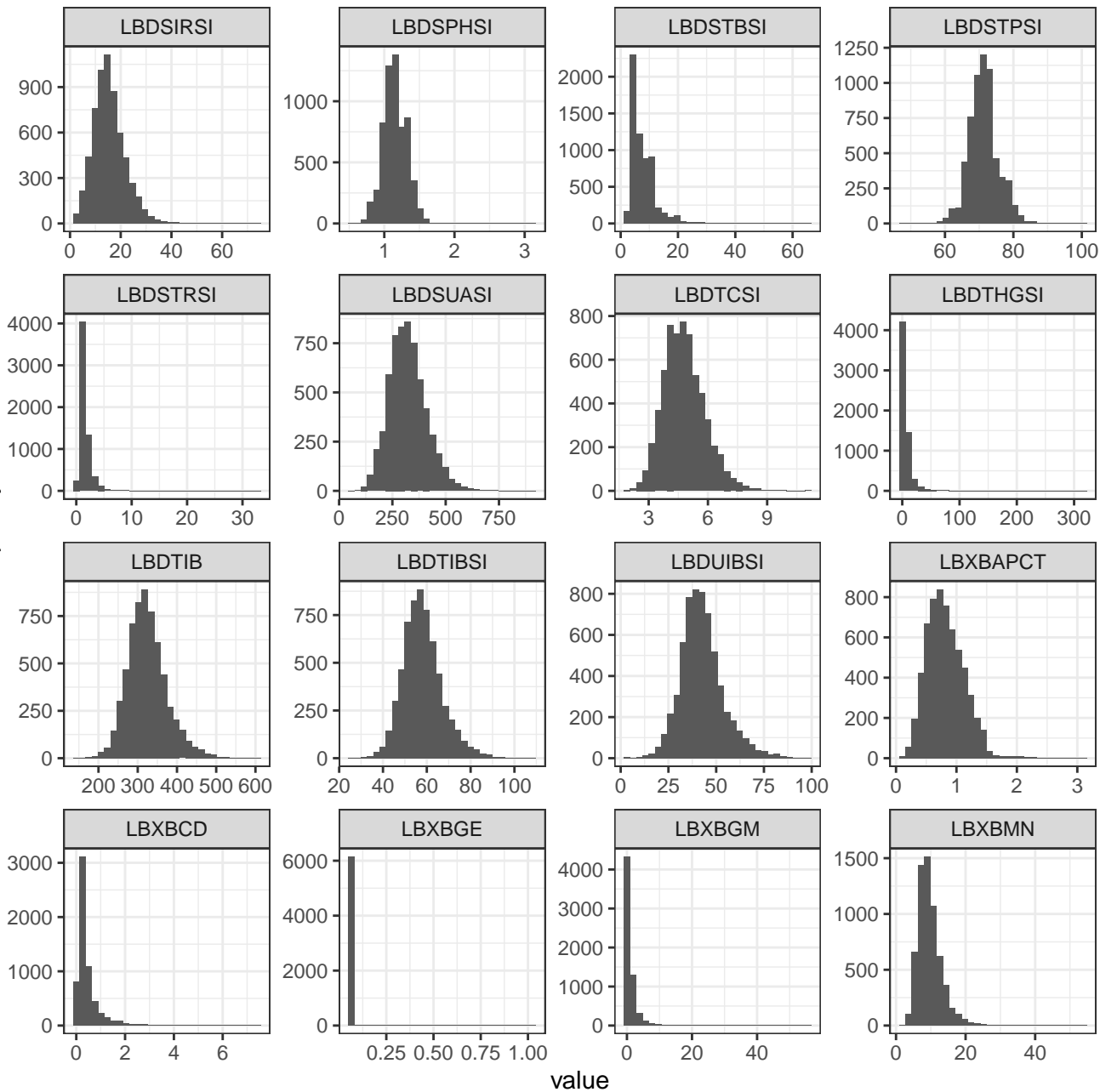

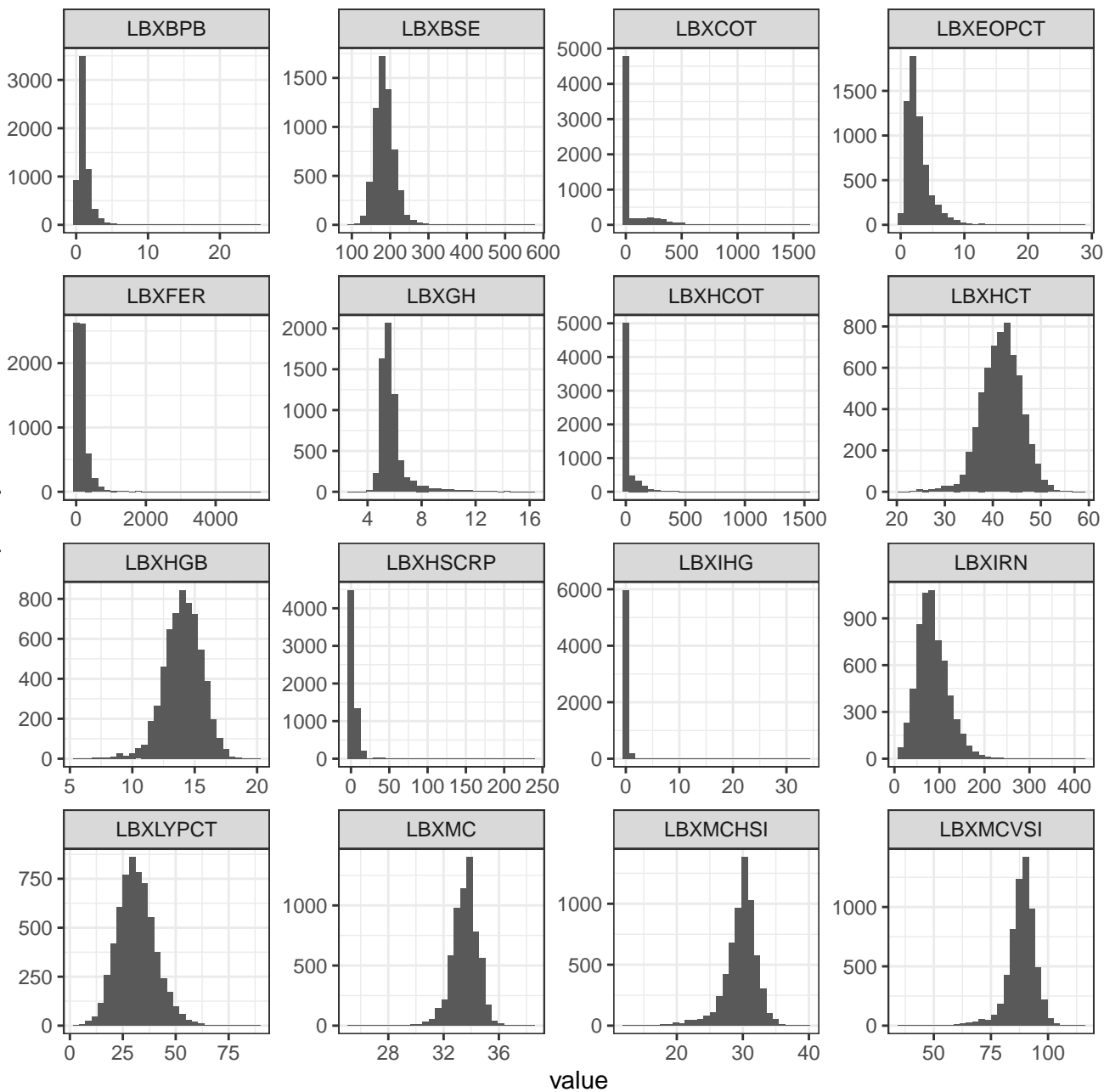

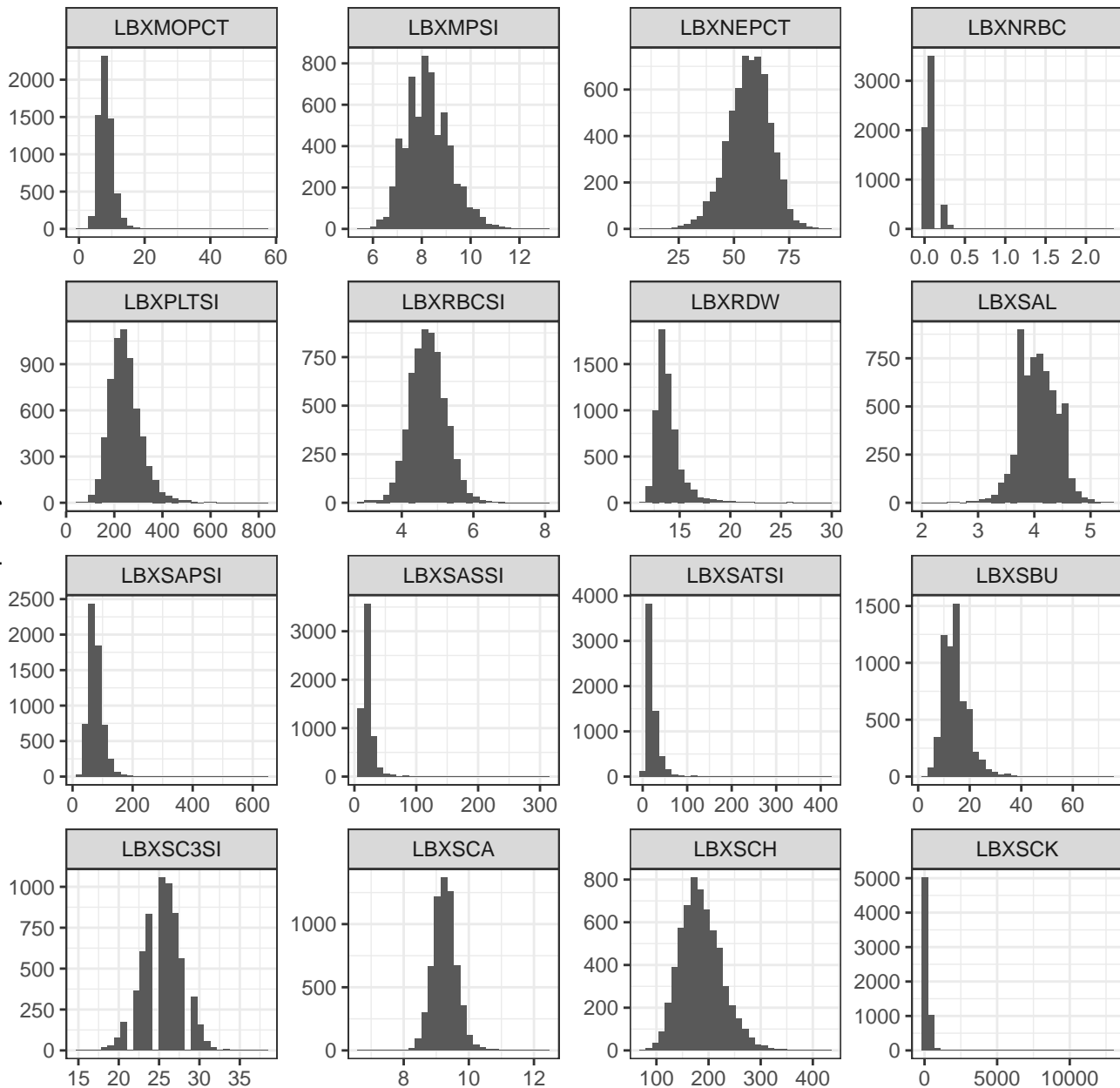

Frequency

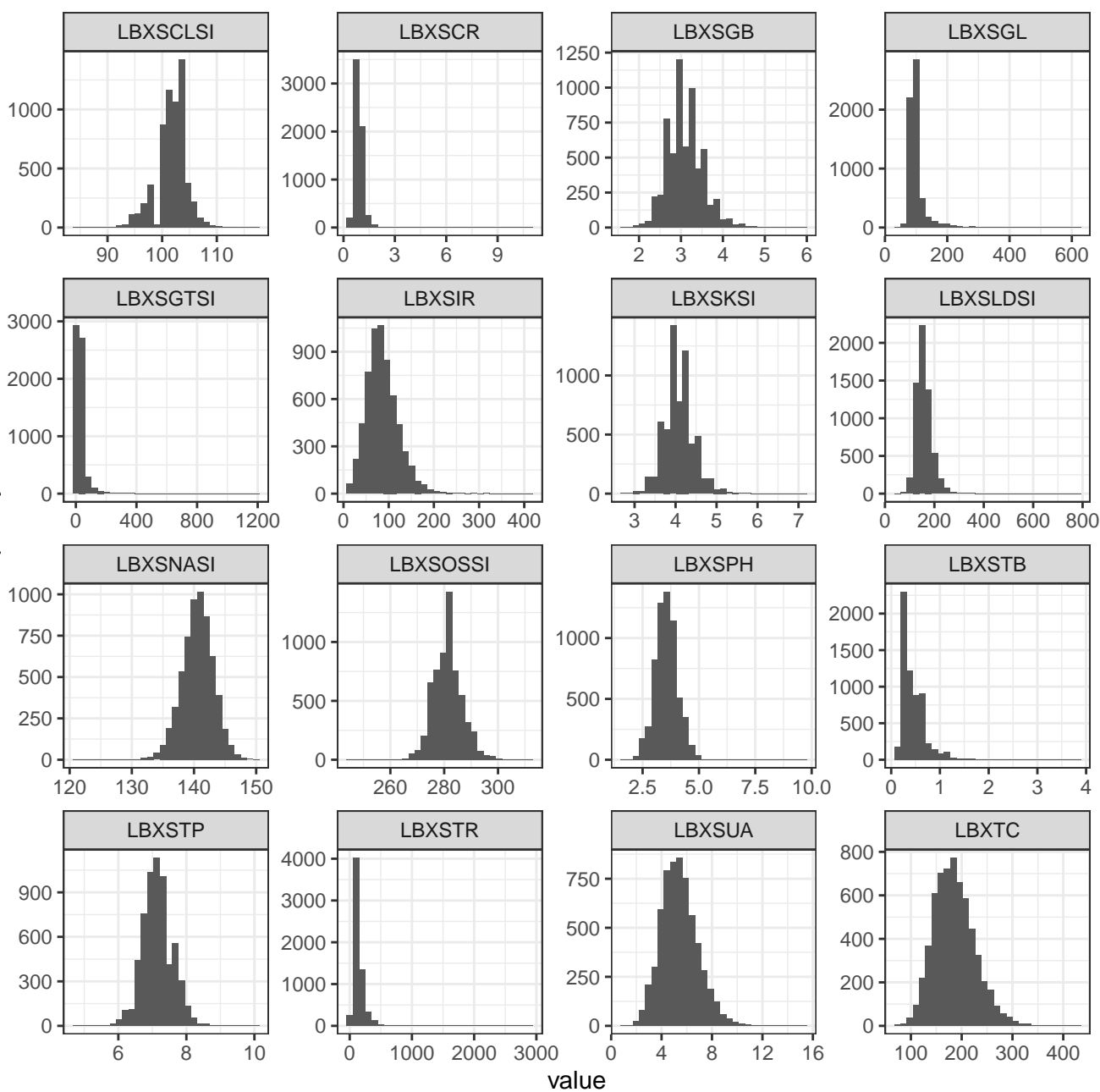

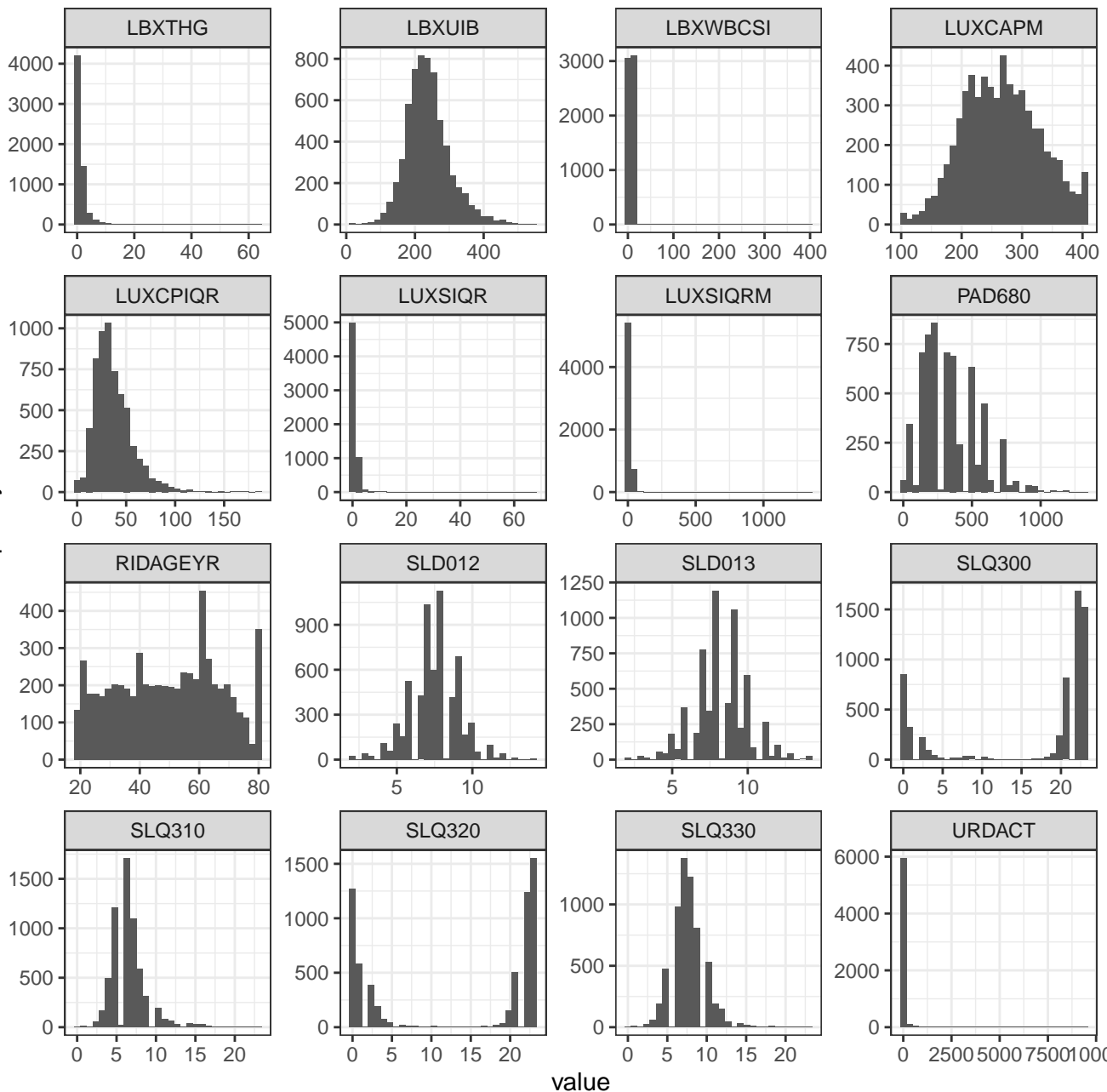

Frequency

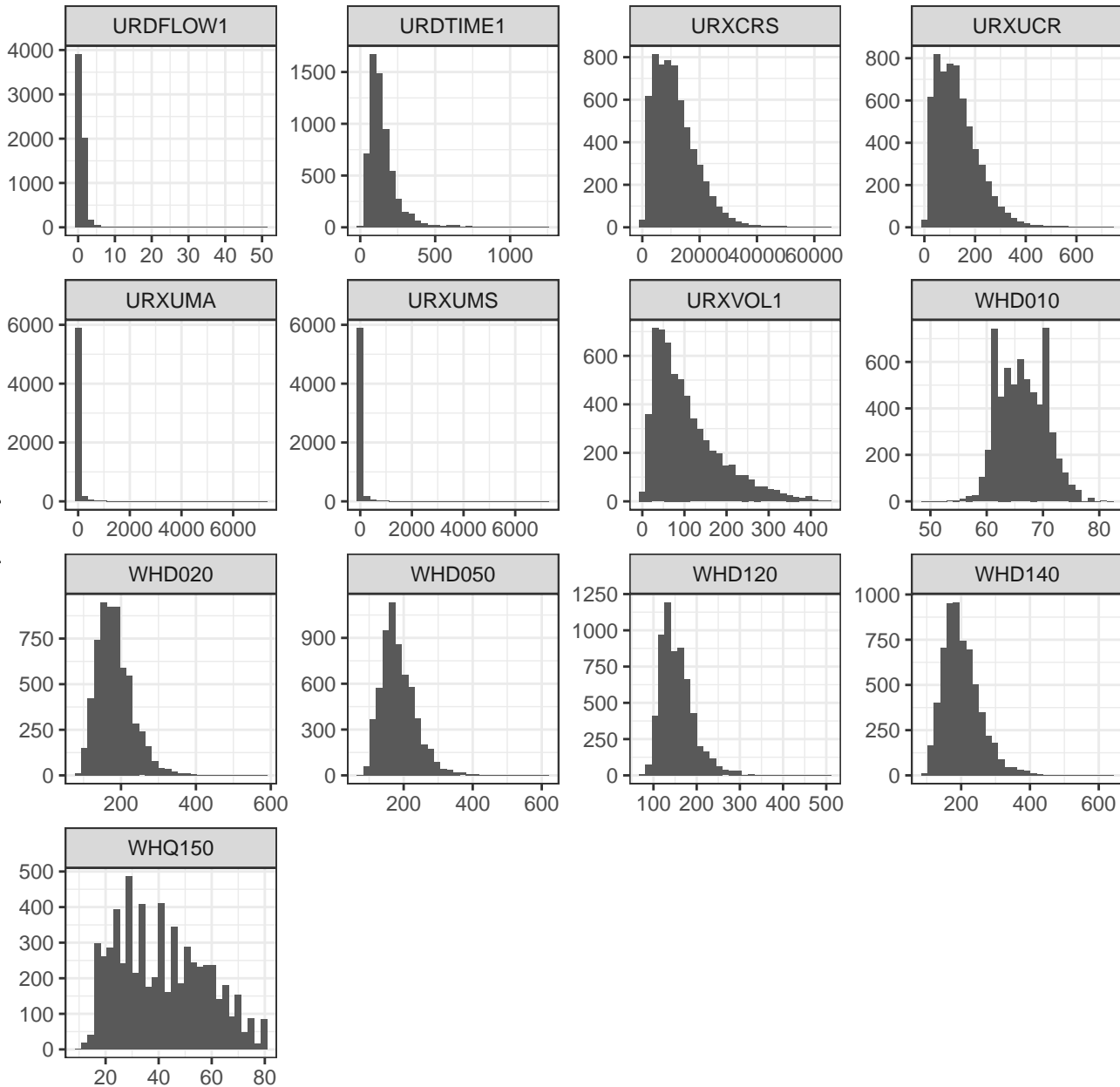

value

Supplement: Supplementary file 7 — This figure presents the distribution characteristics of the original values of relevant continuous variables in the clinically significant liver fibrosis study through a series of histograms. Each subplot corresponds to one indicator, where the horizontal axis represents the original measured values of the variable, and the vertical axis shows the frequency of occurrence of the values. These histograms intuitively display the laws of data distribution, facilitating a quick judgment on the degree of variable skewness and providing a basis for subsequent data analysis— the more significant the skewness of a variable, the more necessary it is to perform data transformation to meet the model assumptions. [file Data_Sheet_7.pdf]

Frequency

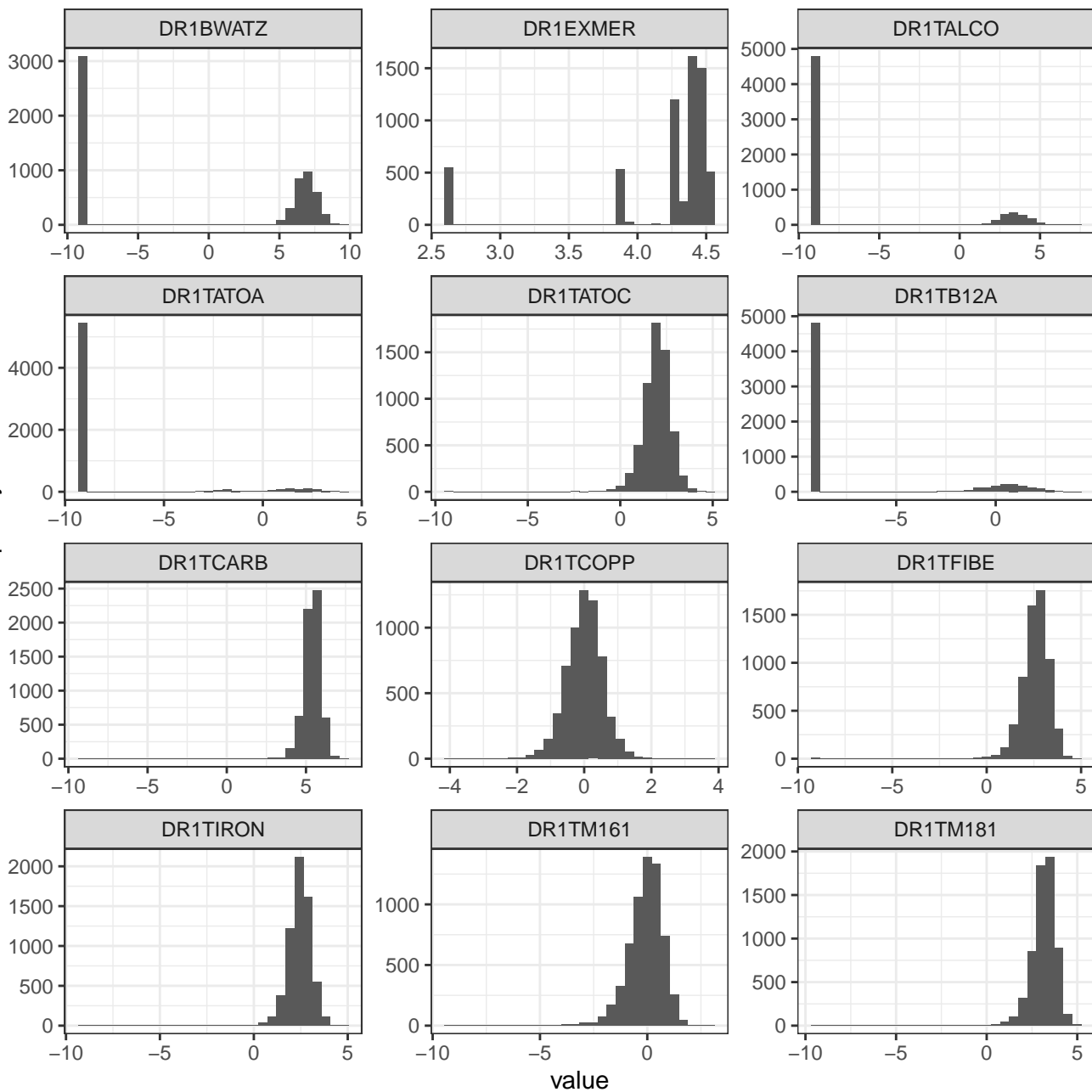

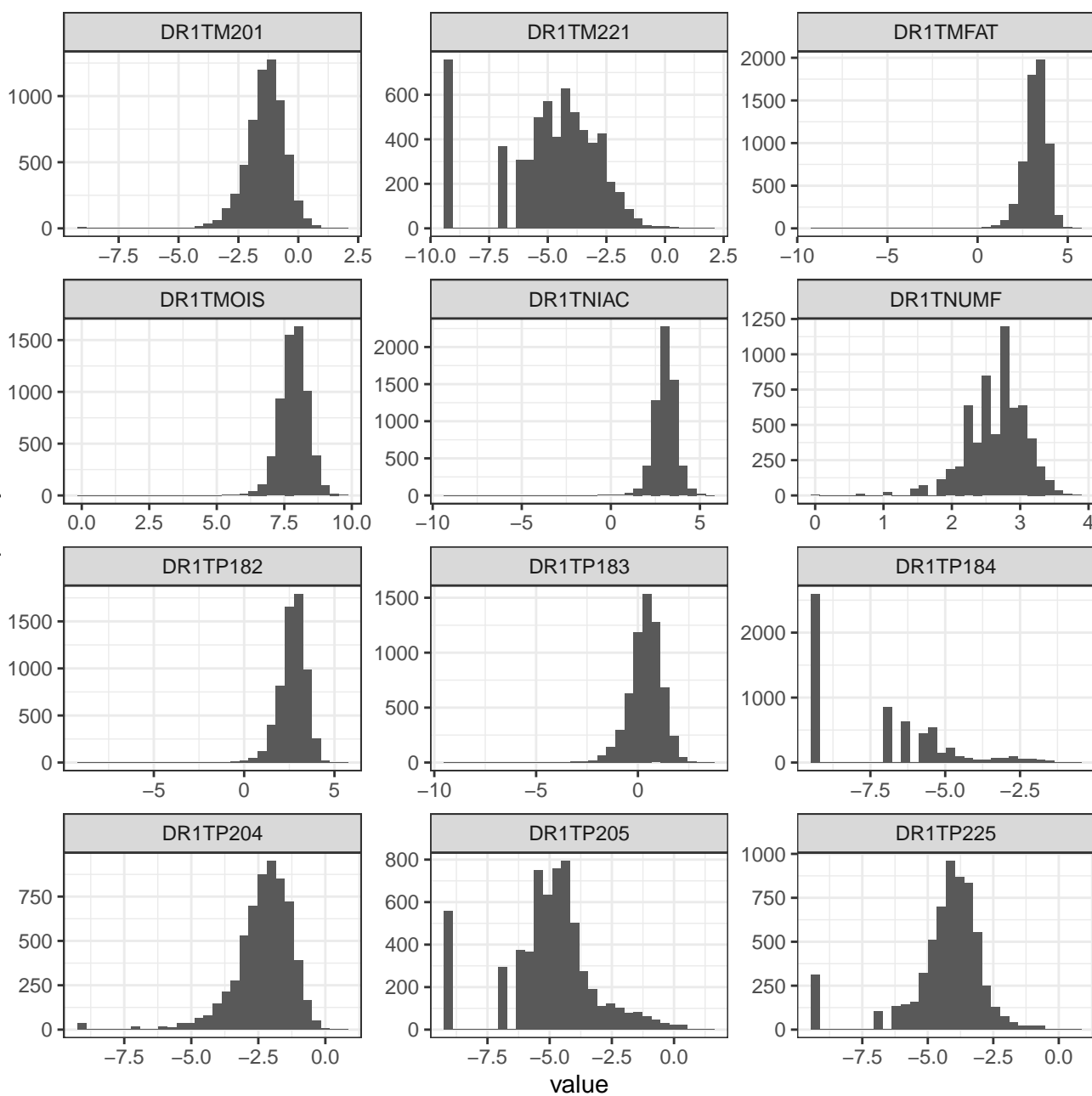

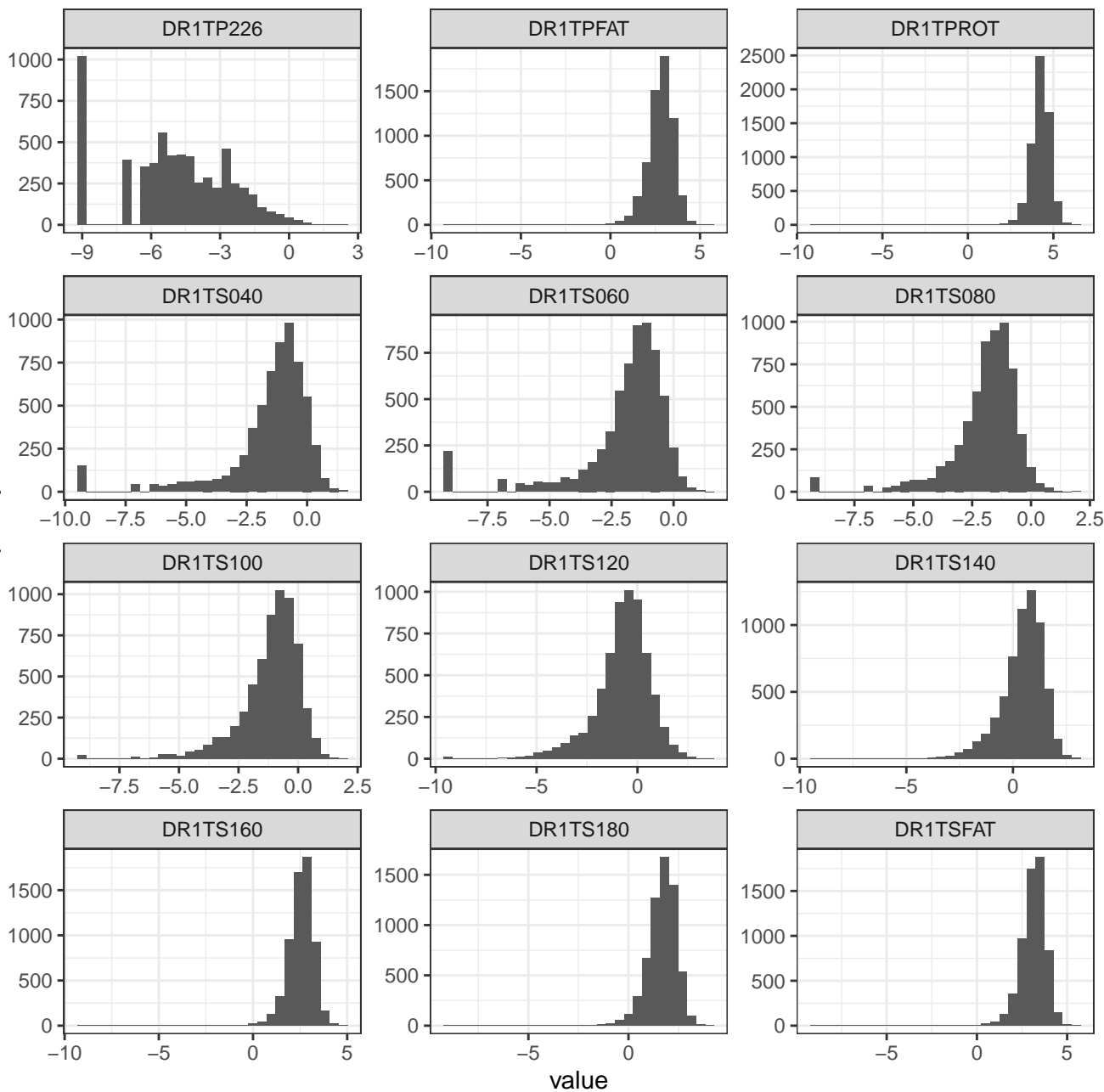

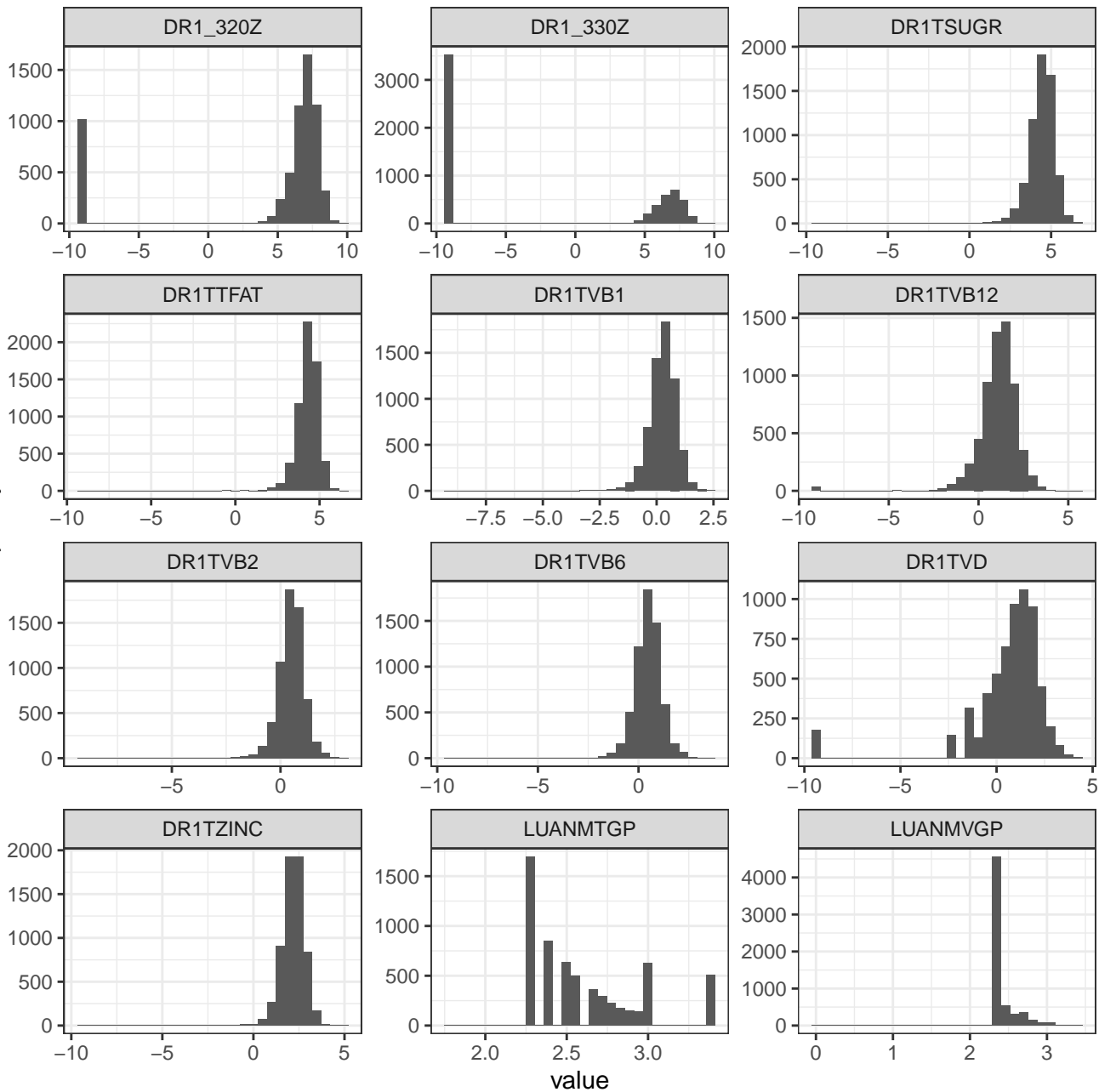

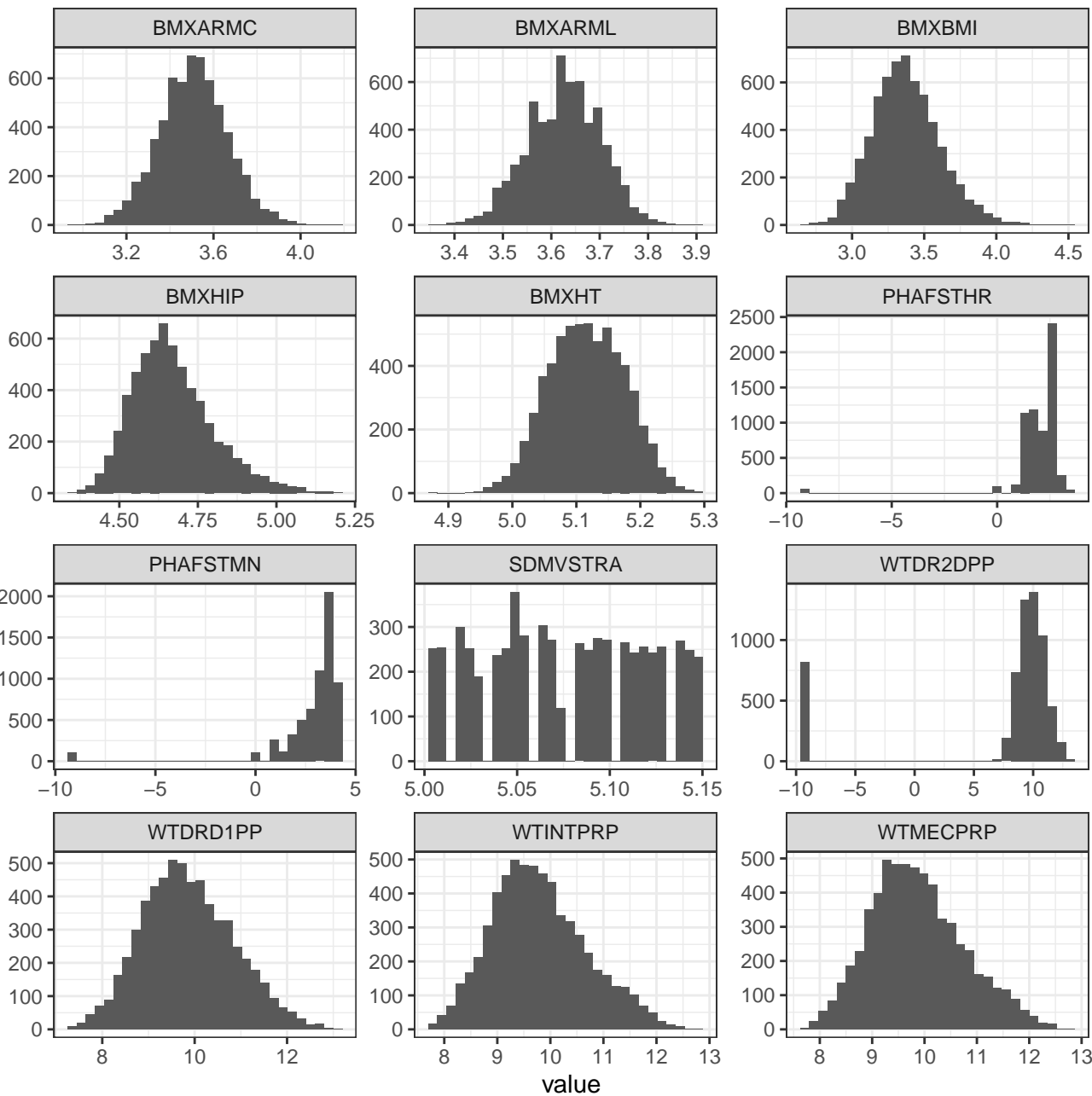

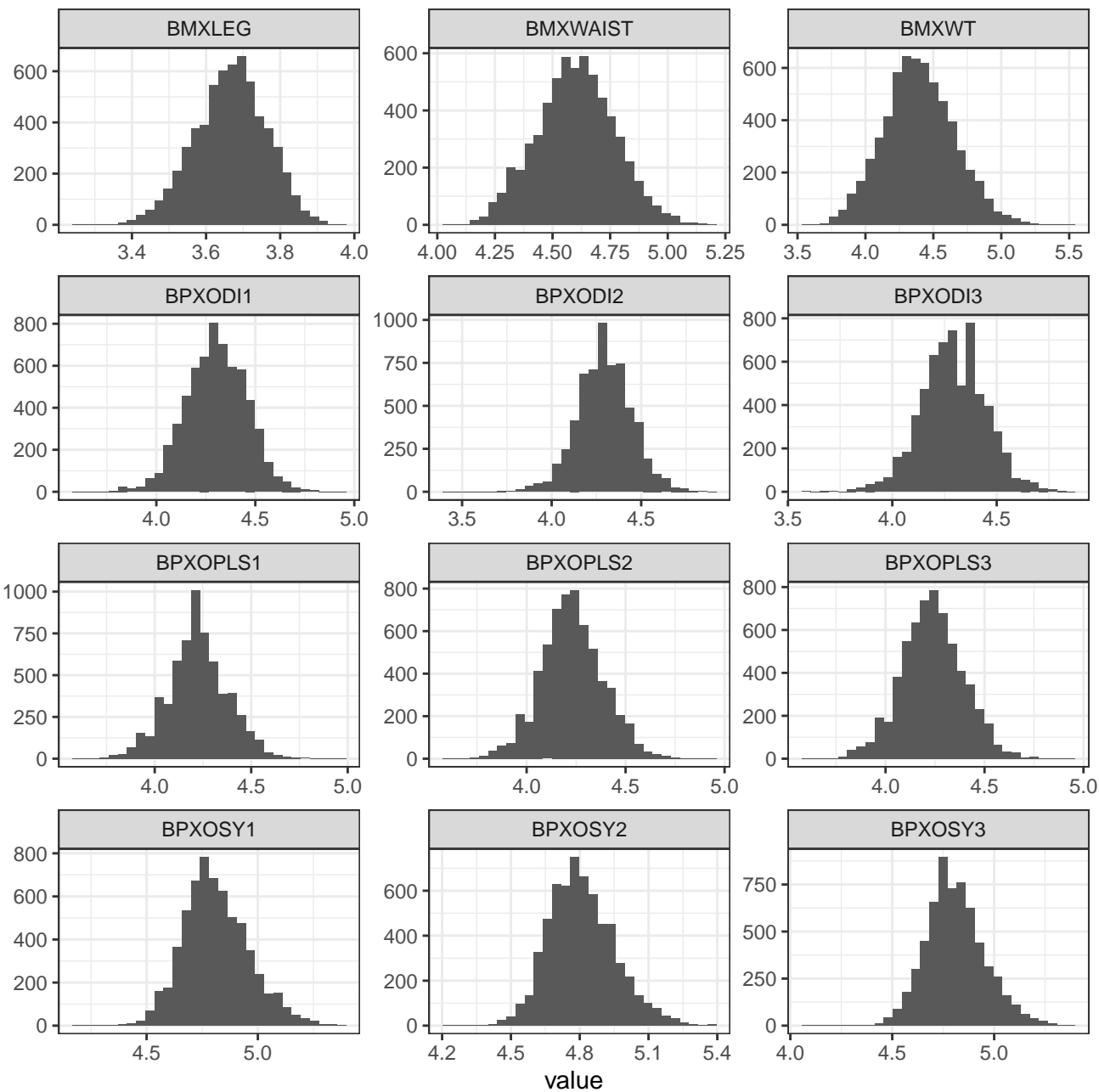

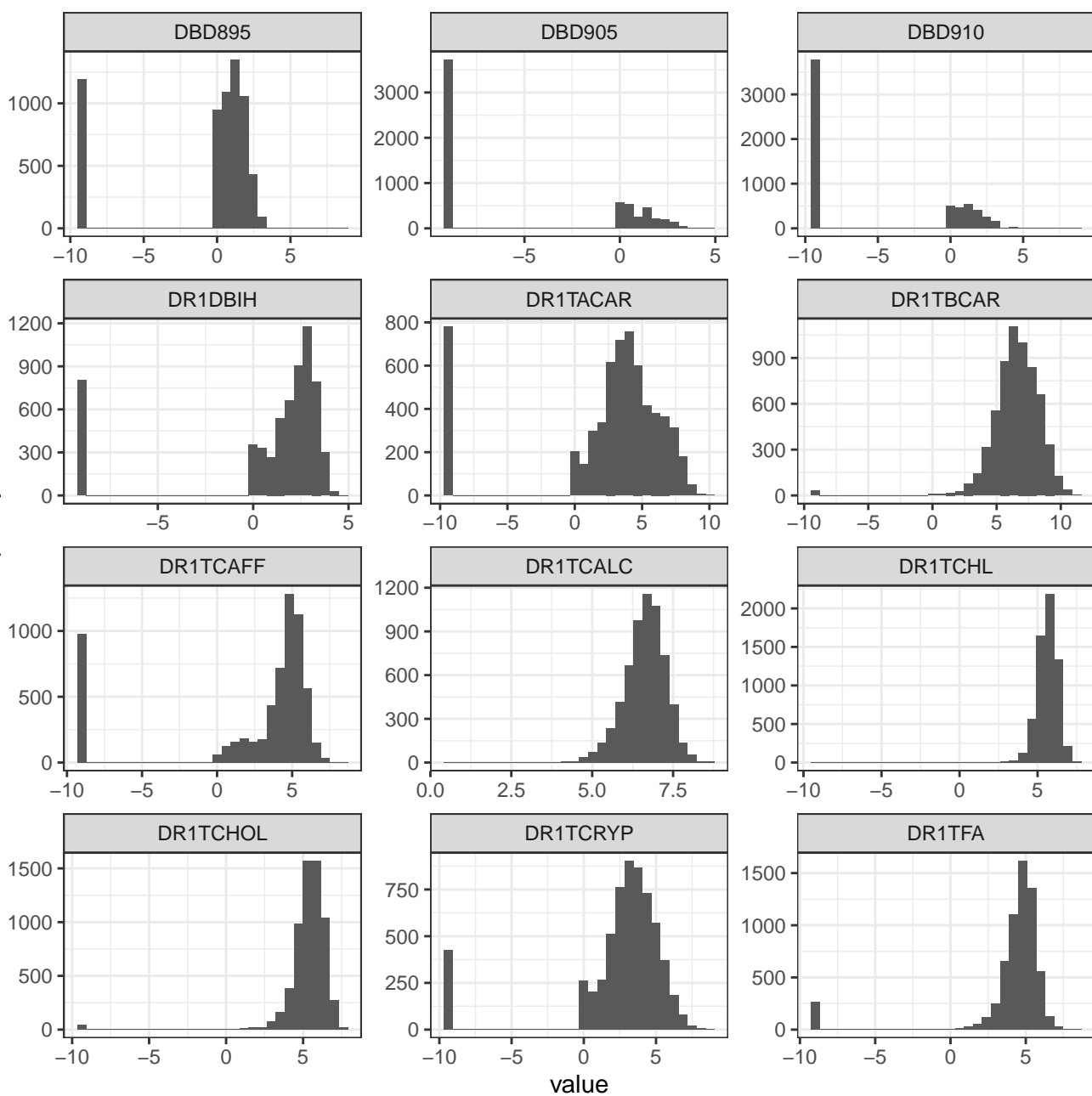

Frequency

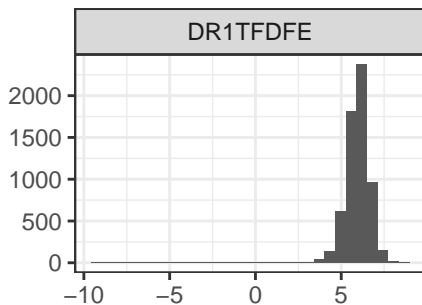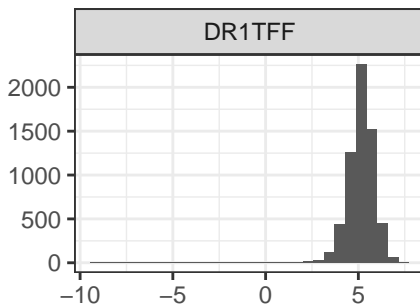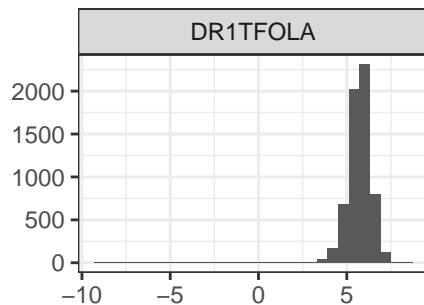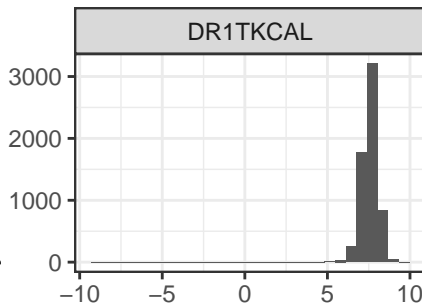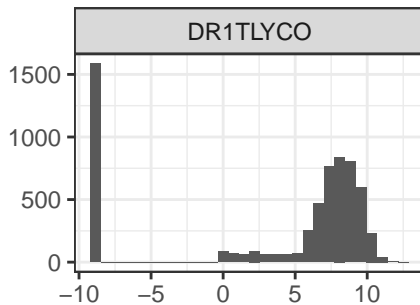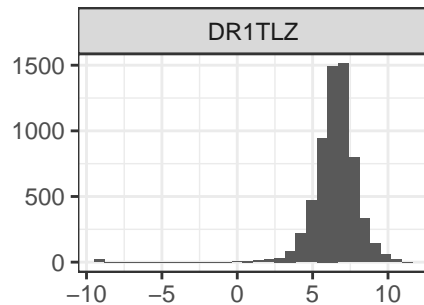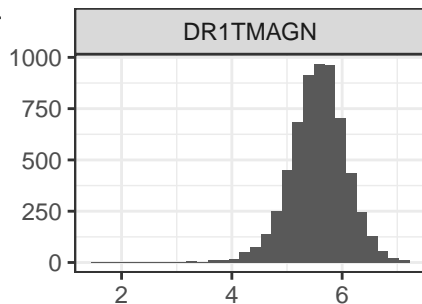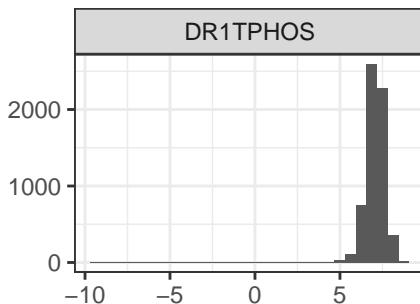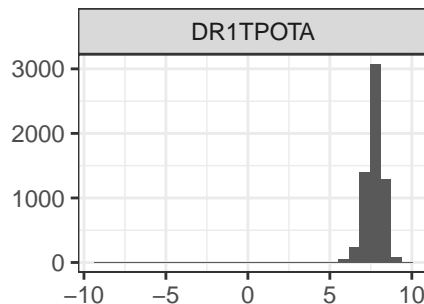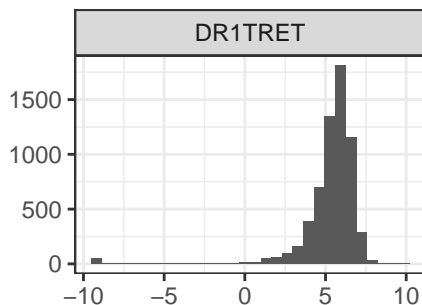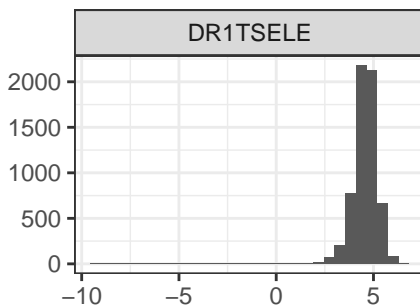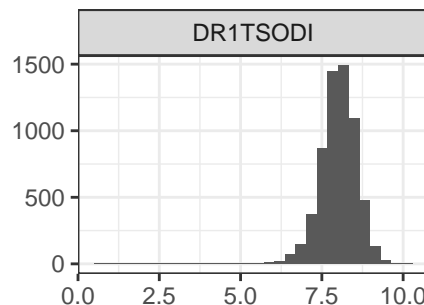

value

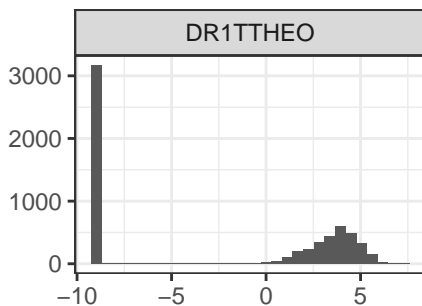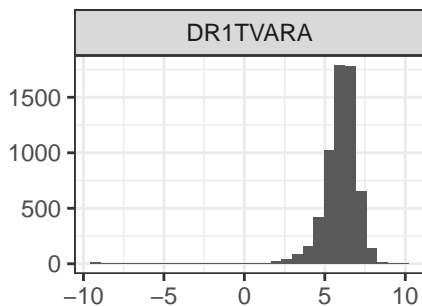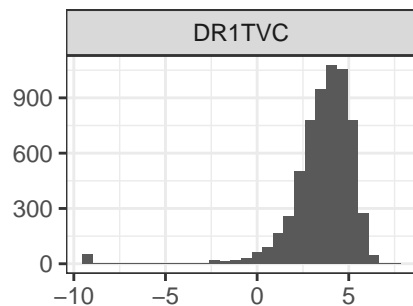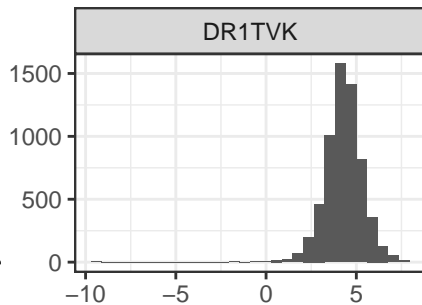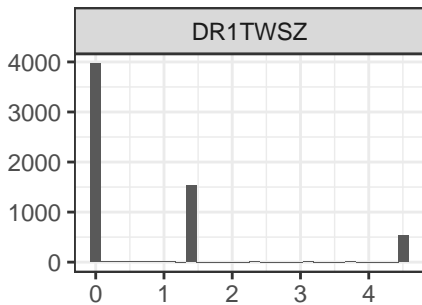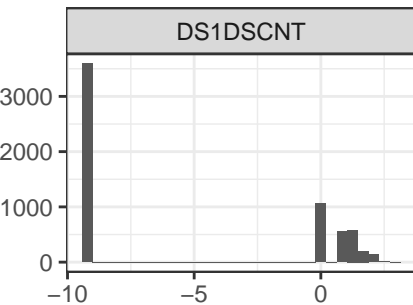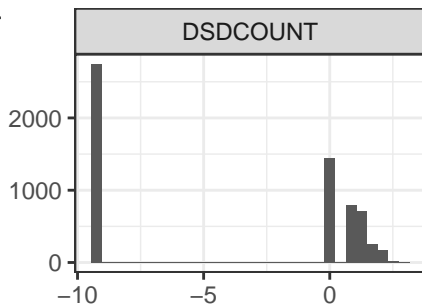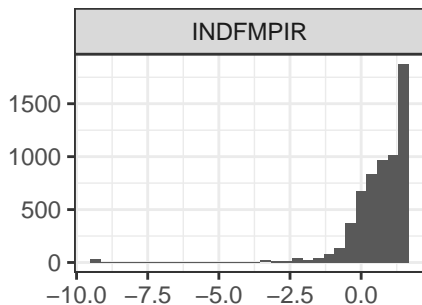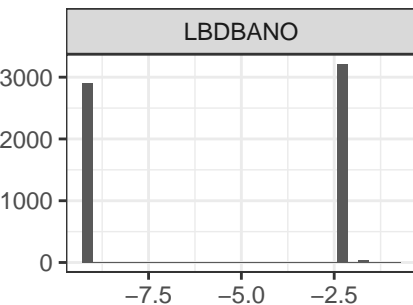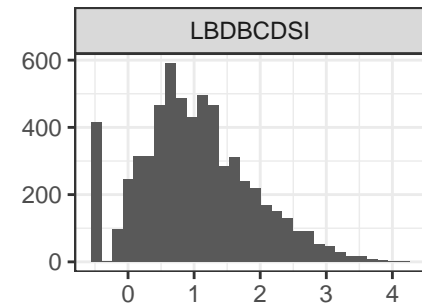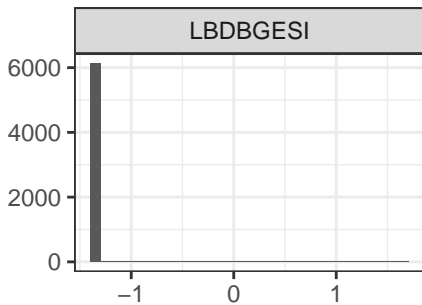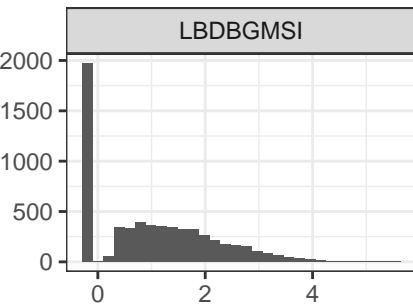

value

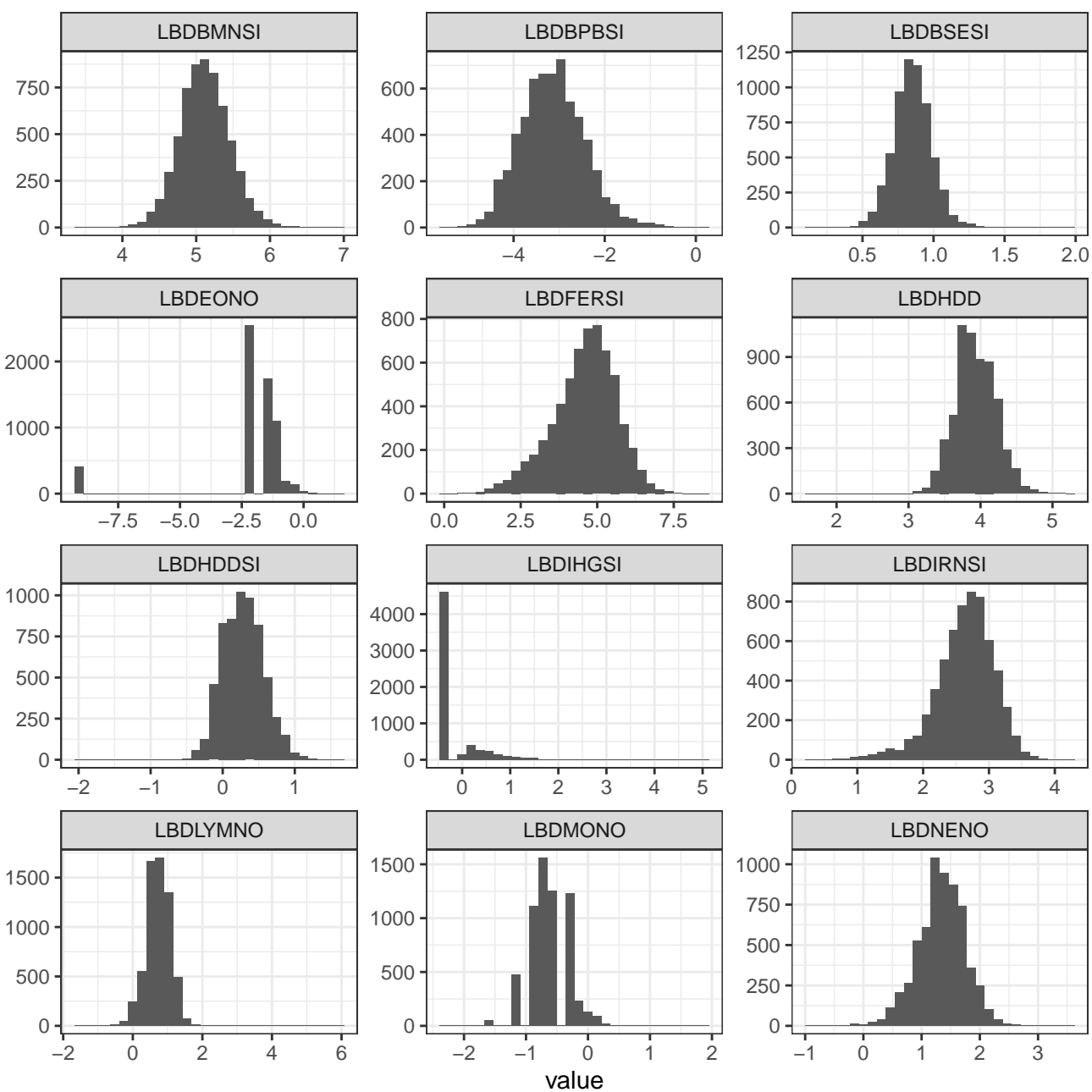

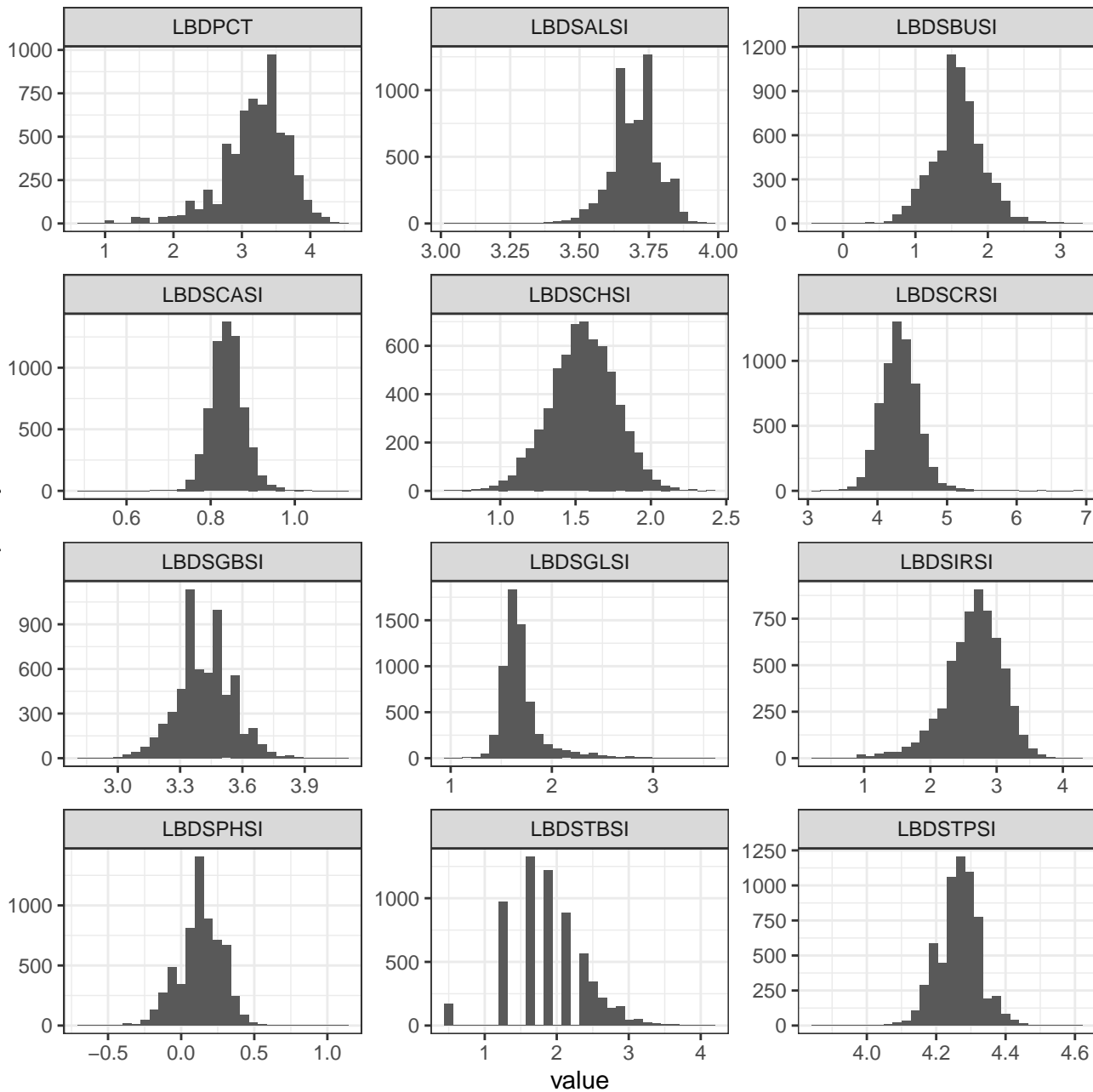

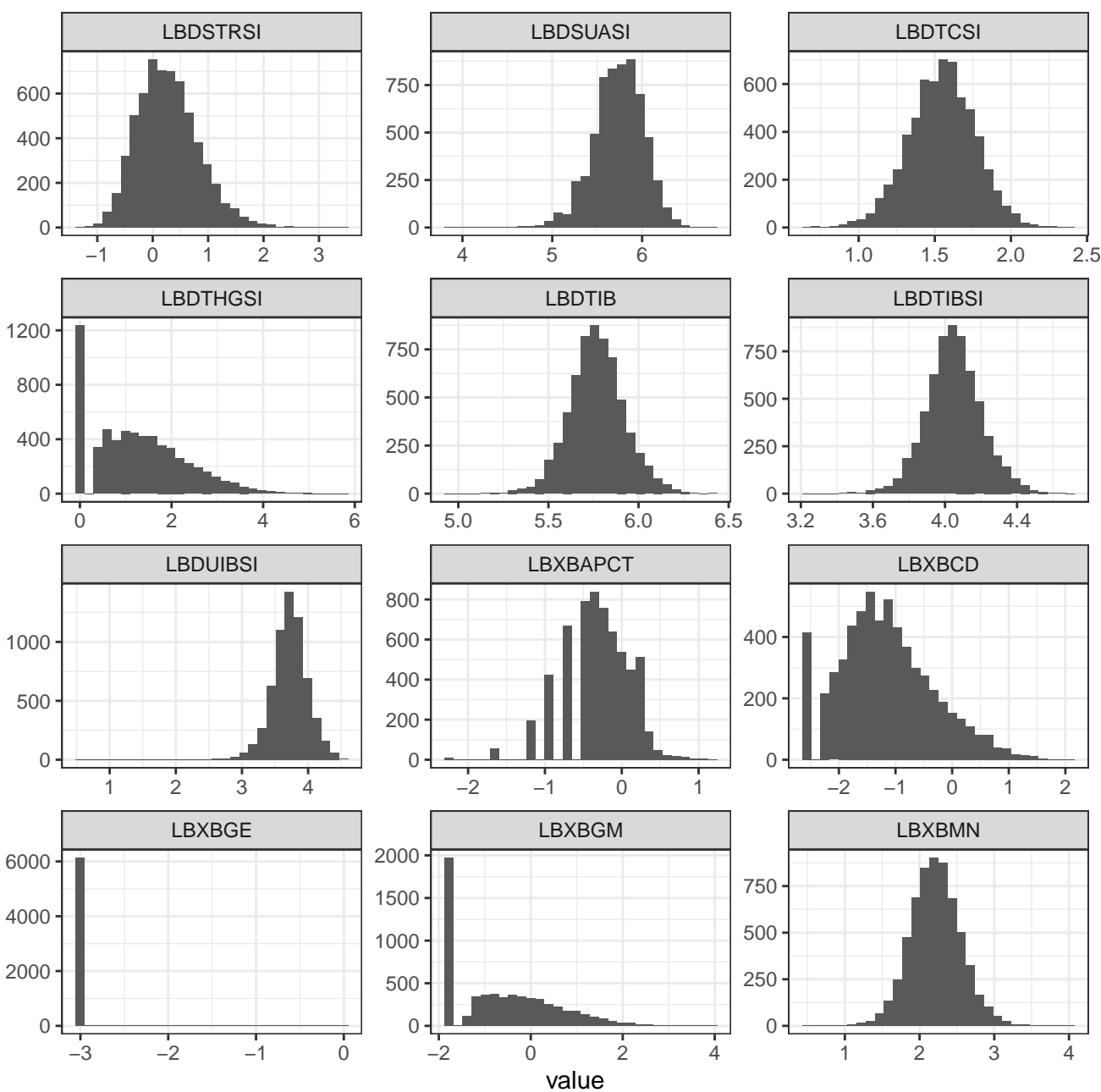

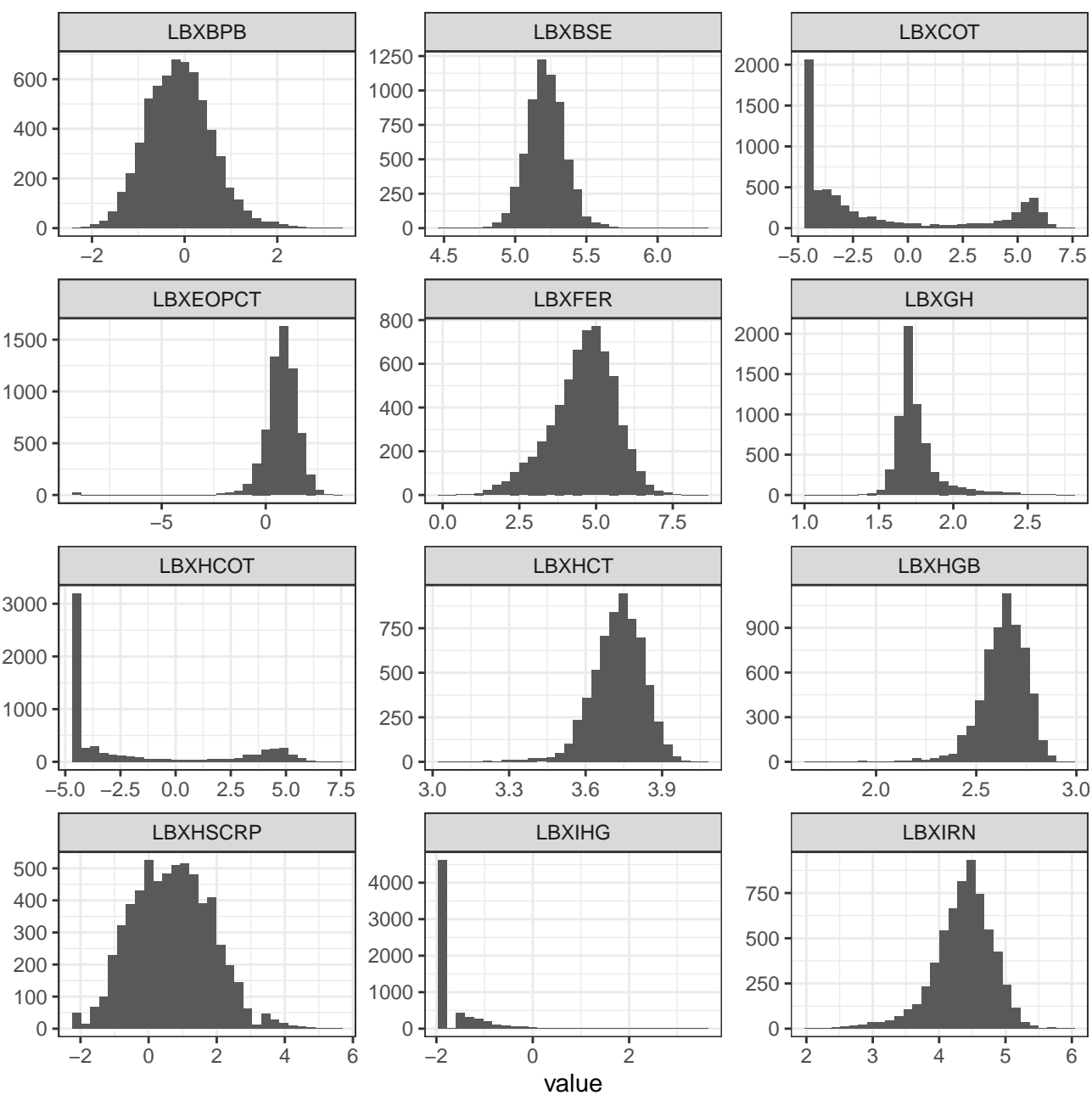

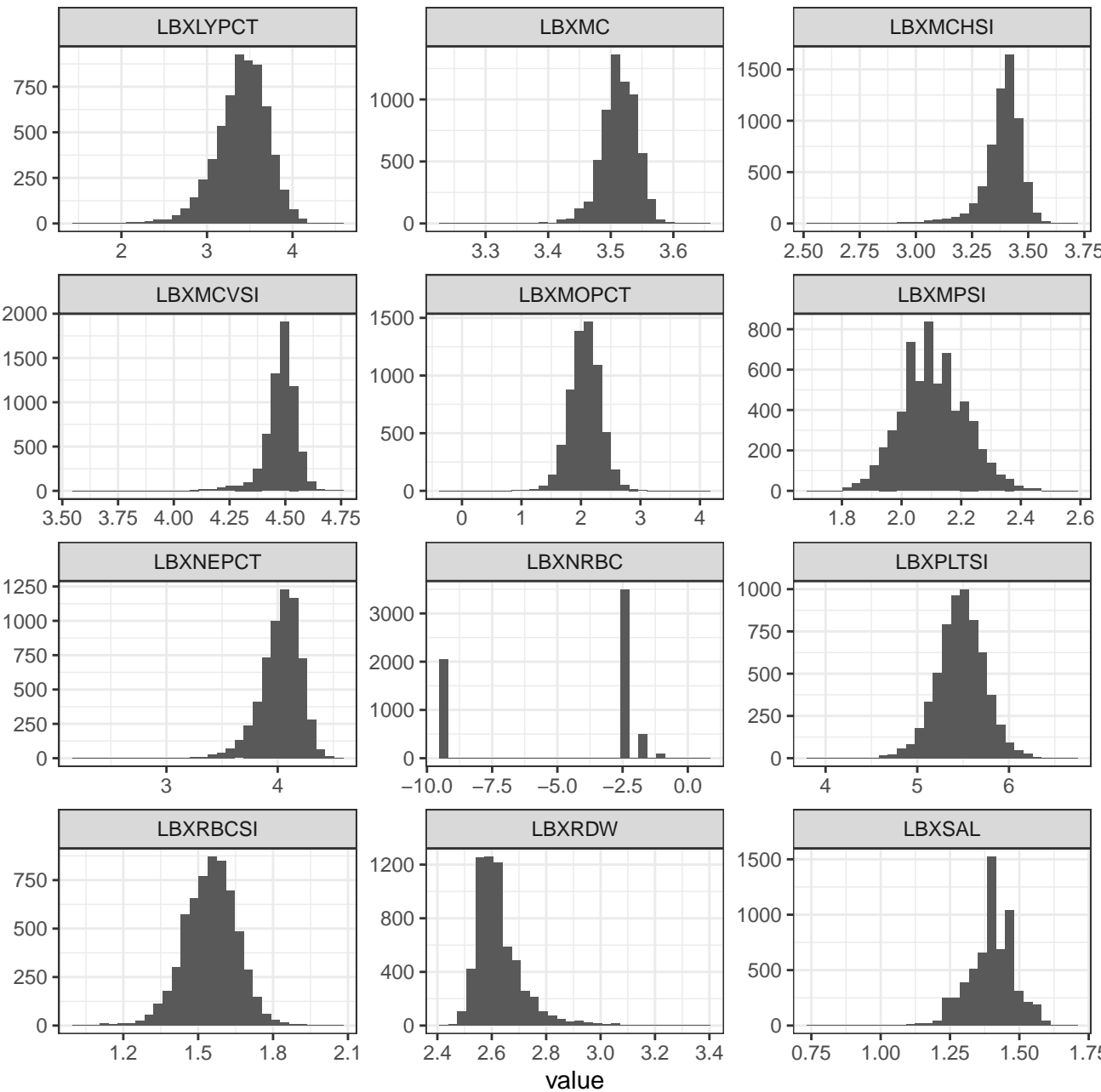

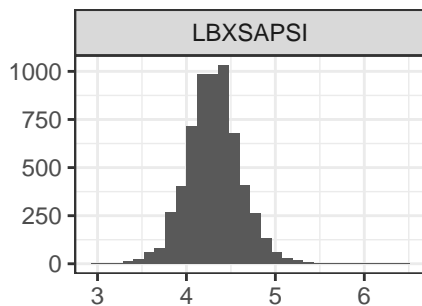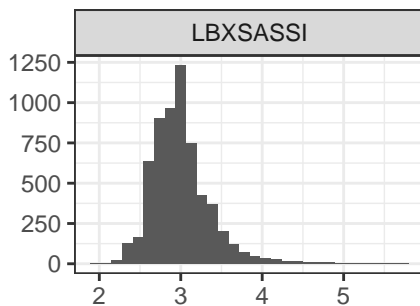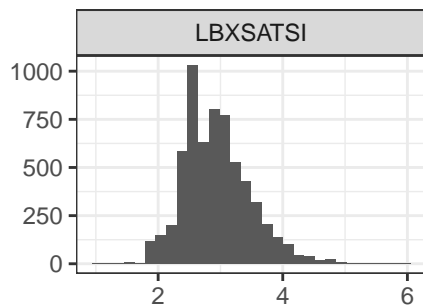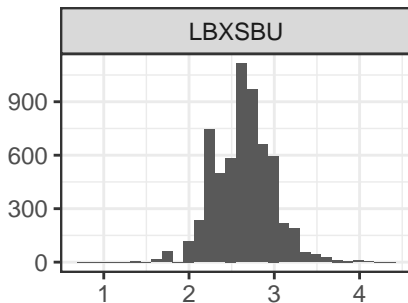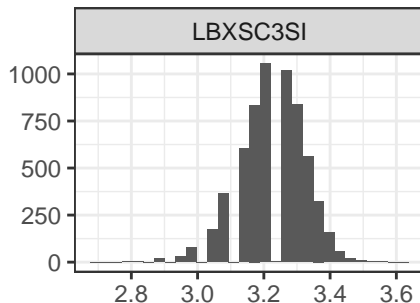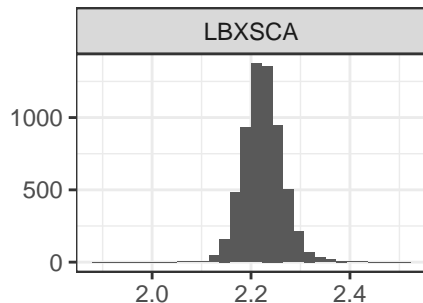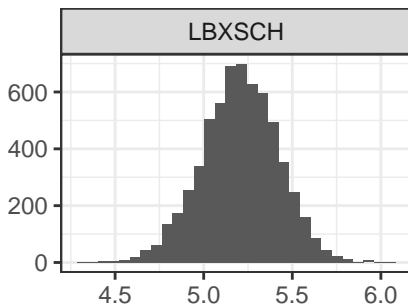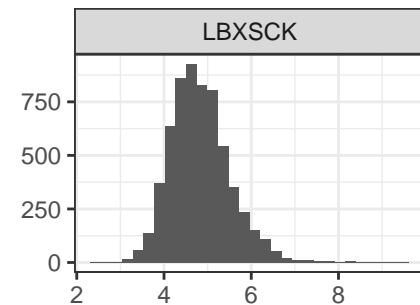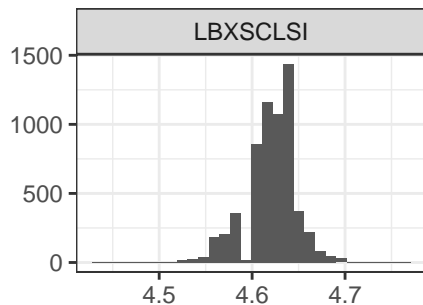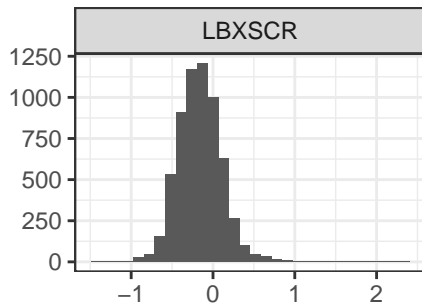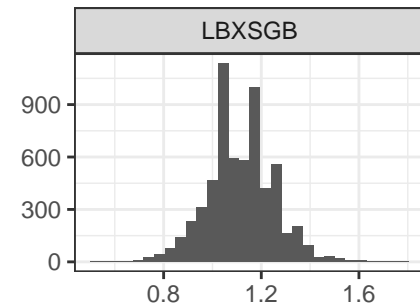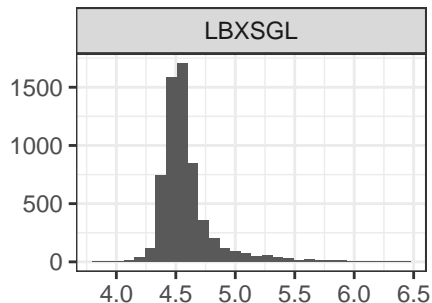

value

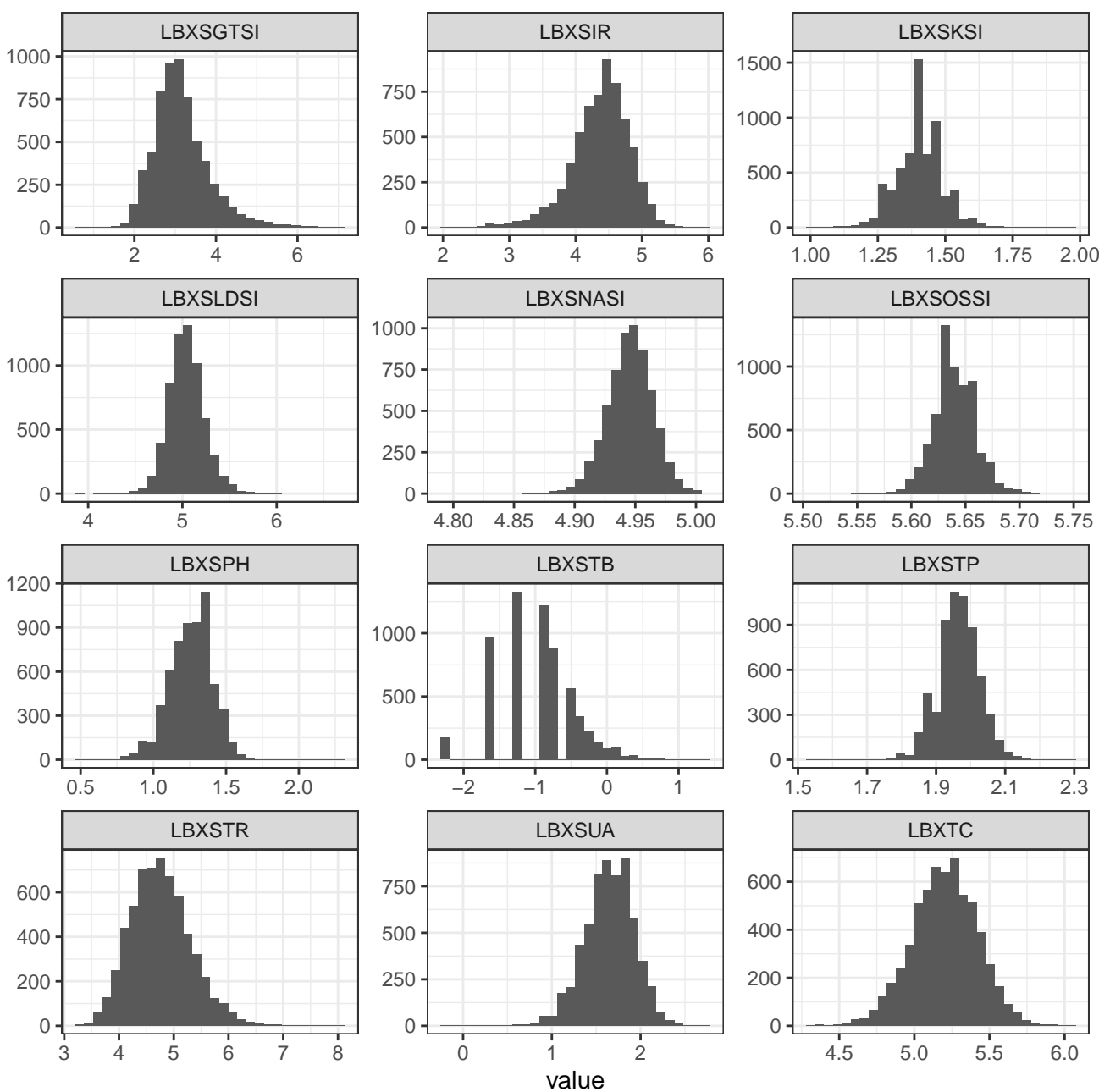

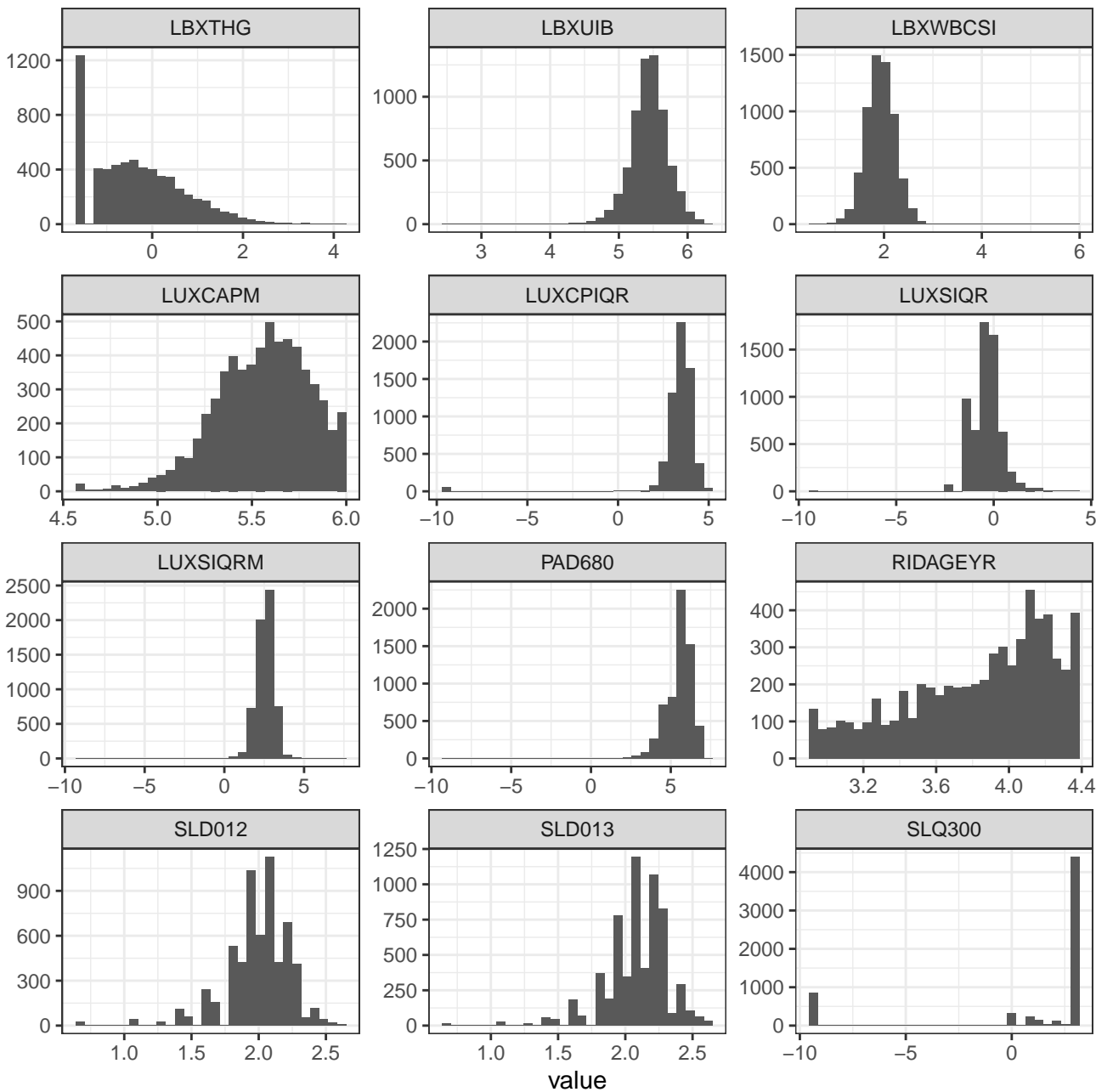

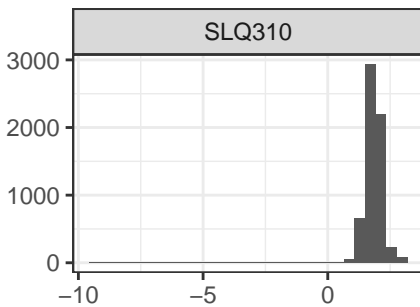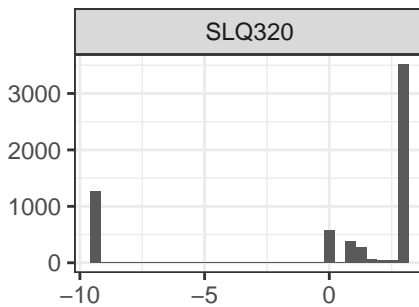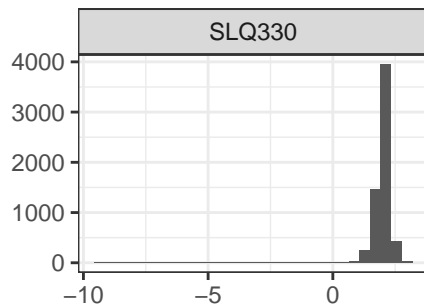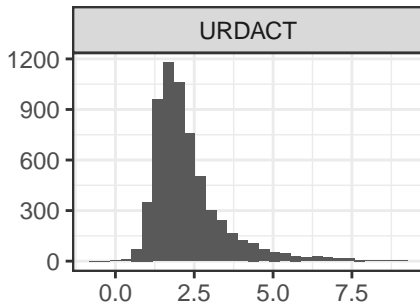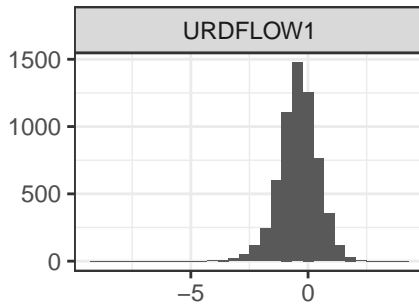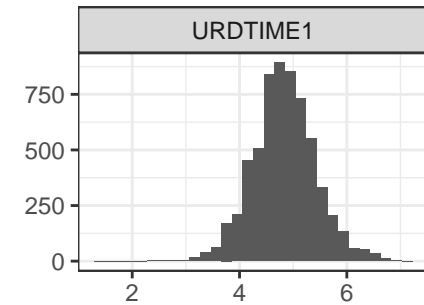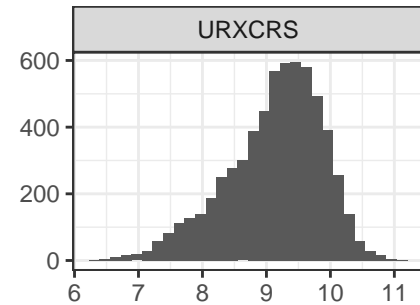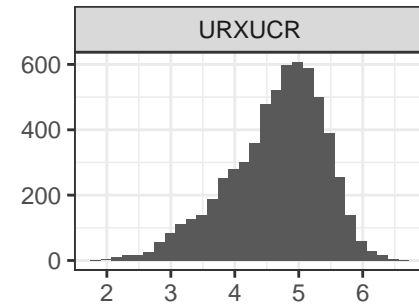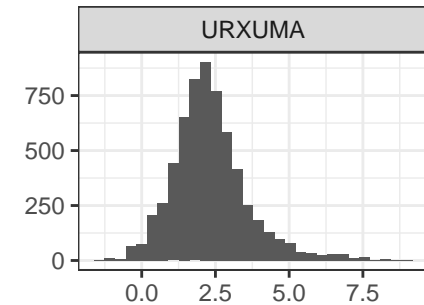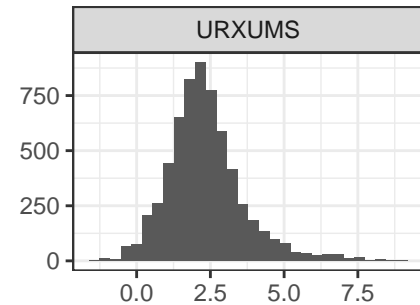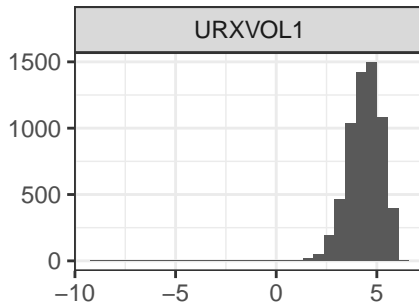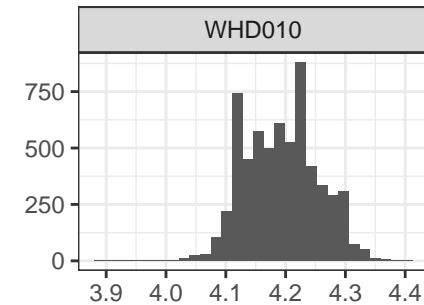

value

Frequency

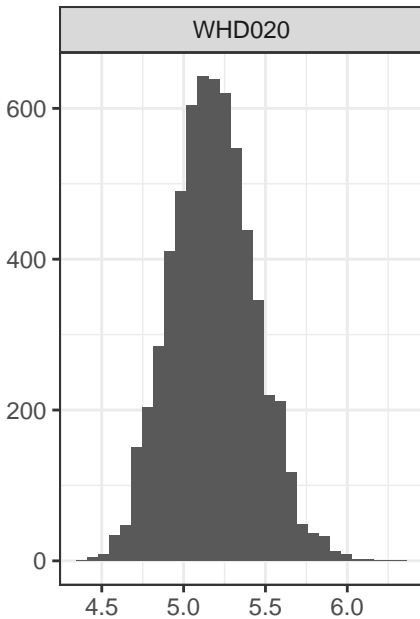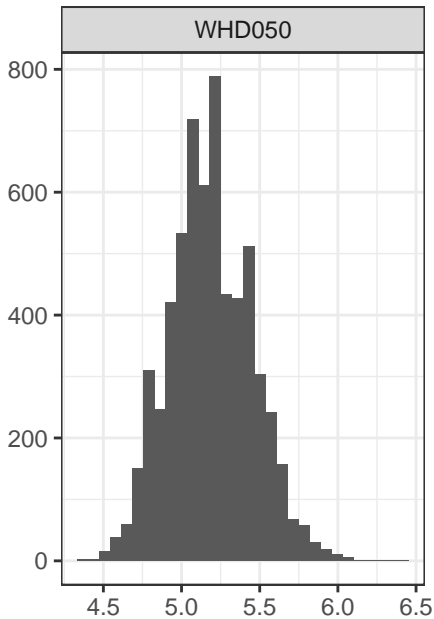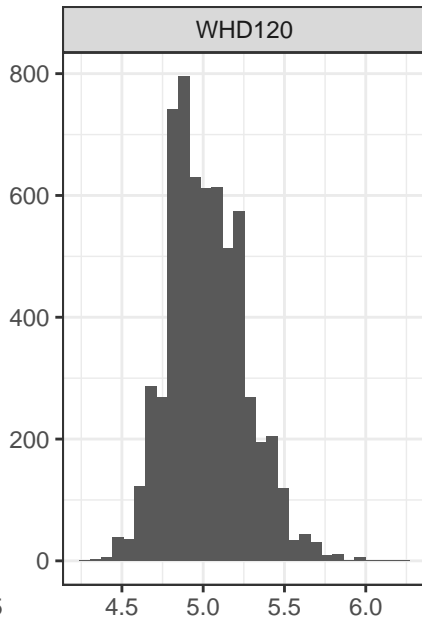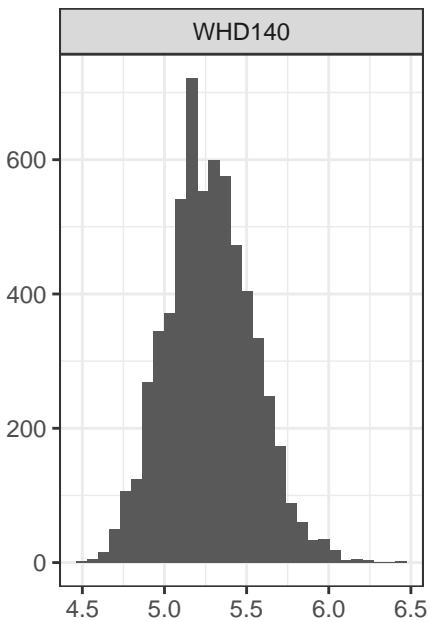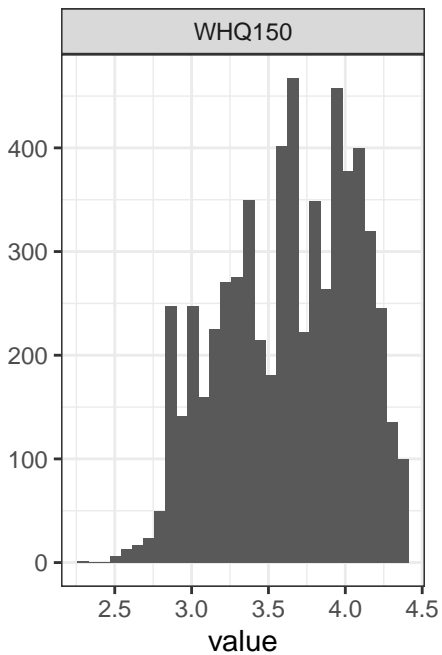

Supplement: Supplementary file 8 — This figure presents the numerical distribution characteristics of relevant continuous variables in the clinically significant liver fibrosis study after logarithmic transformation through a series of histograms. Each subplot corresponds to one indicator, intuitively showing the frequency distribution patterns of the transformed data, and providing an optimized data distribution reference for machine learning models in feature processing and analyzing the correlation between variables and clinically significant liver fibrosis. [file Data_Sheet_8.pdf]

Gbm

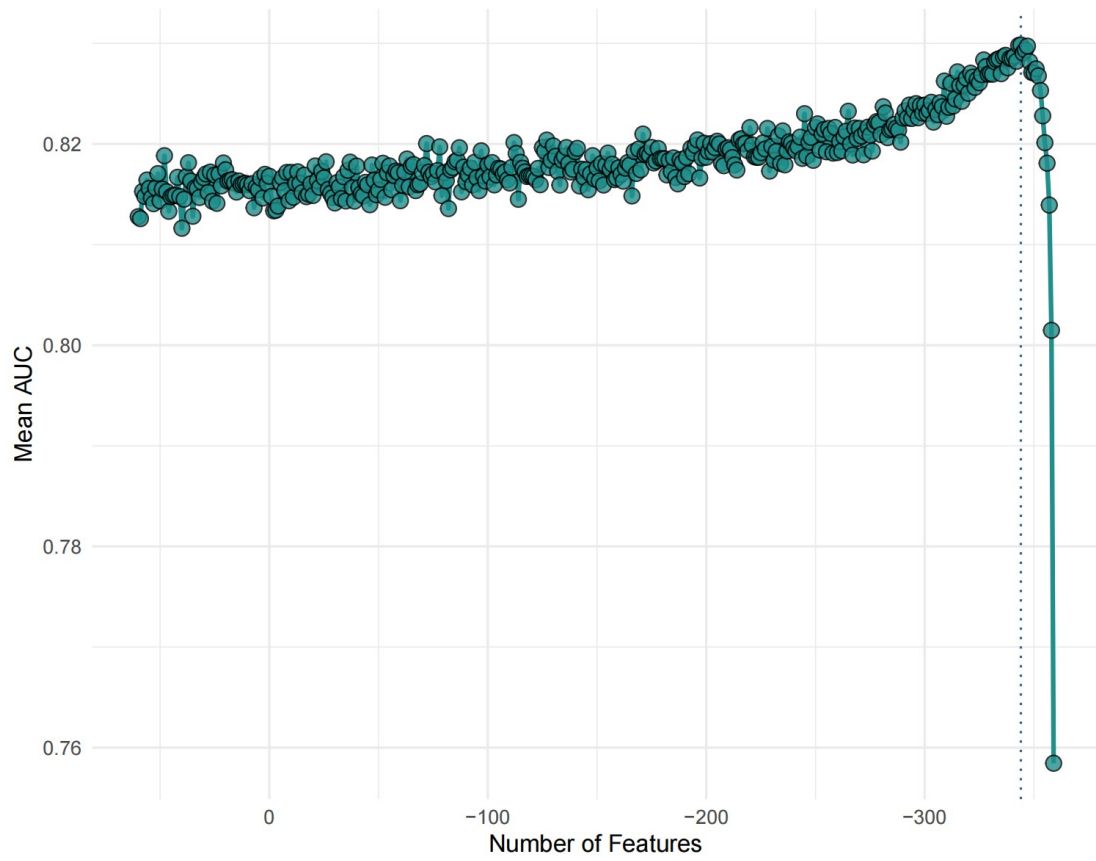

Lightgbm

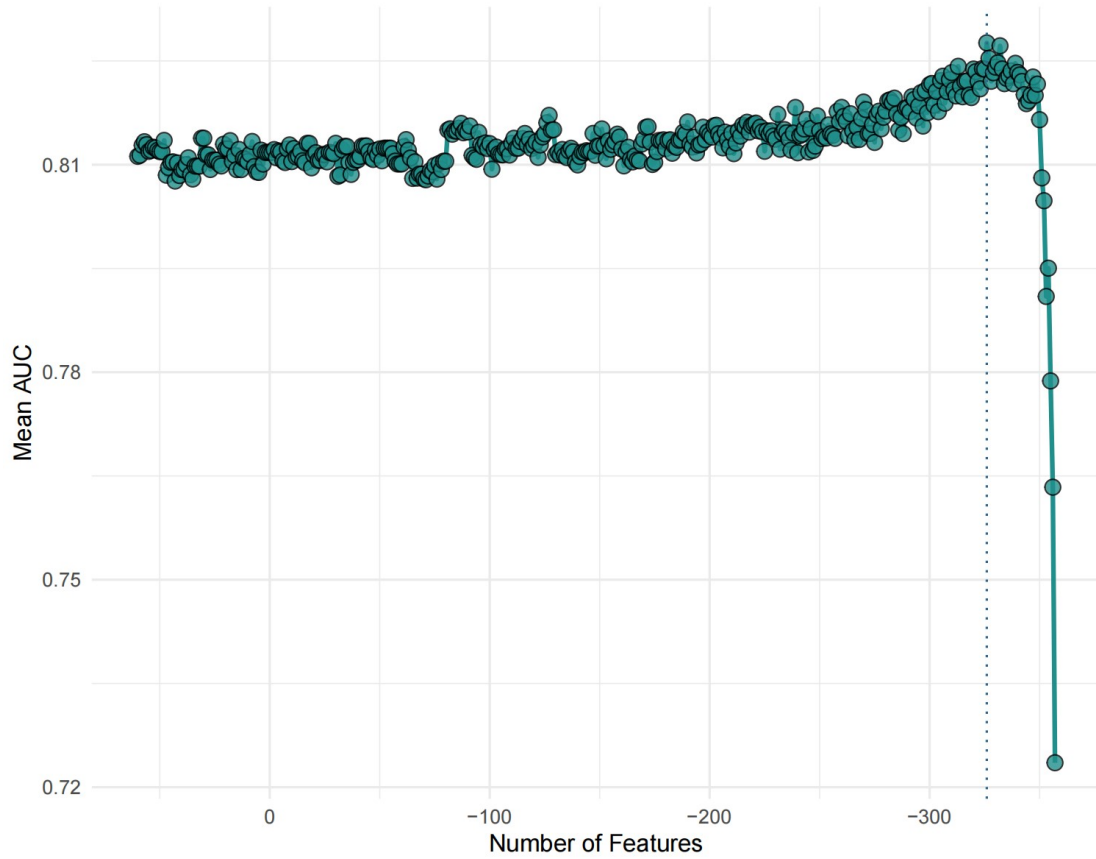

Ranger

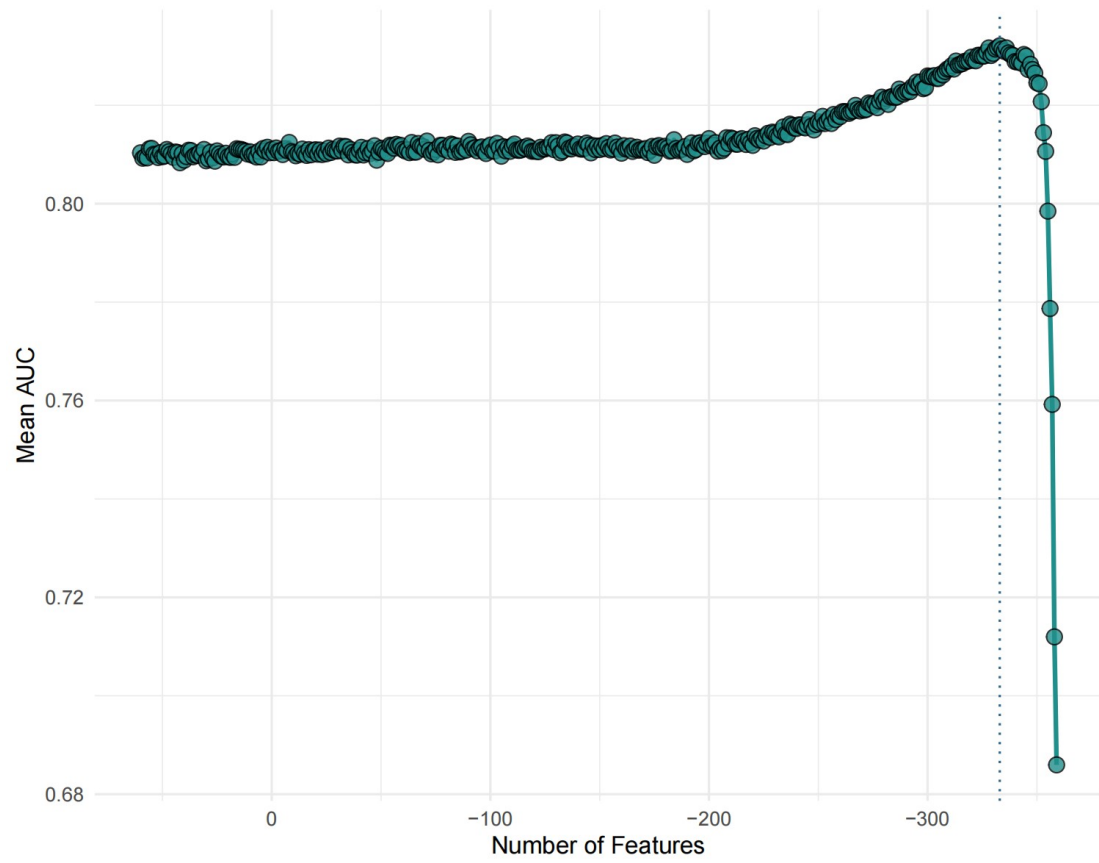

Rpart

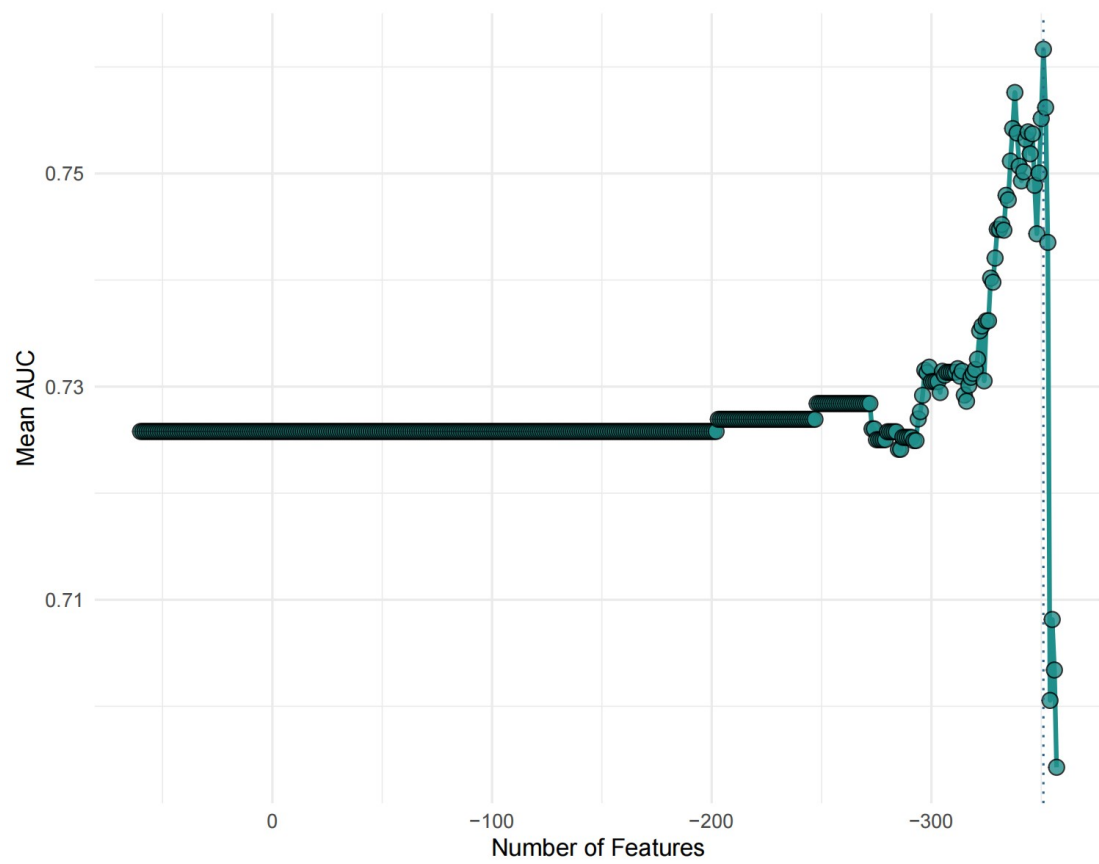

Svm

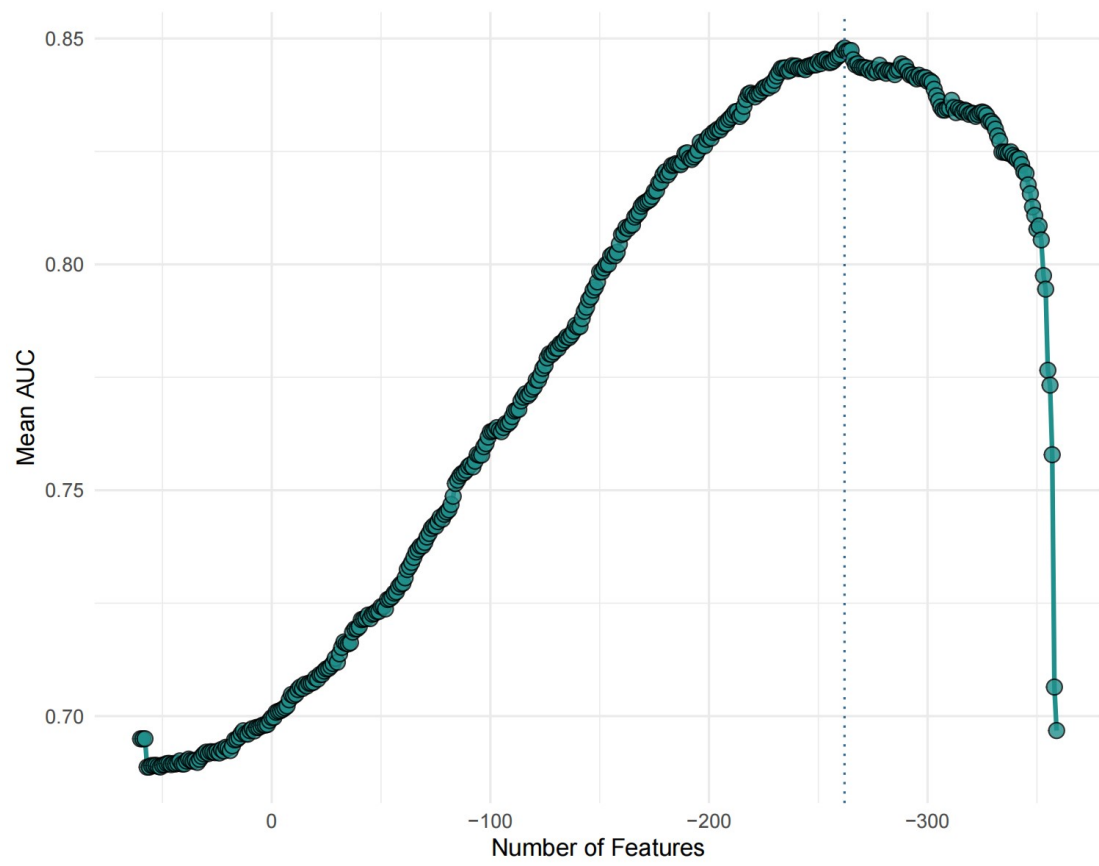

Xgboost

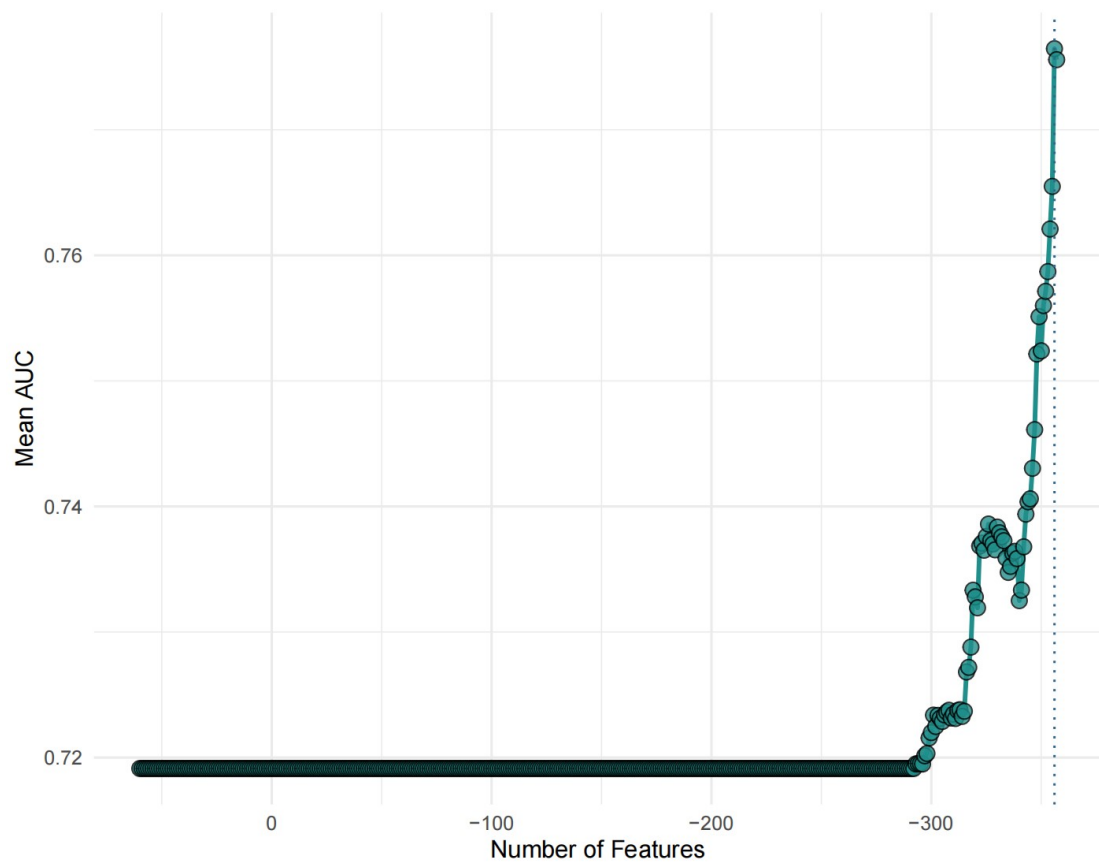

Catboost

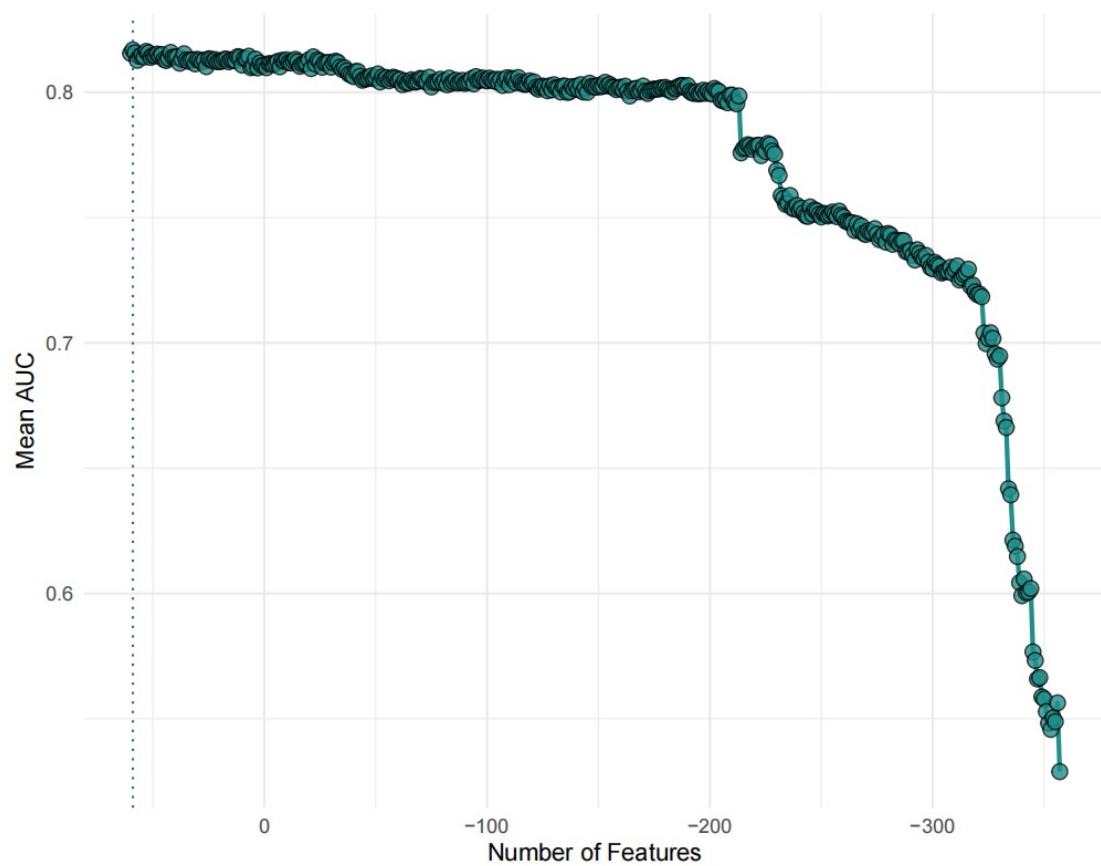

Supplement: Supplementary file 11 — We adopted the Recursive Feature Elimination (RFE) method for feature selection, with the goal of maximizing the AUC value, and used five-fold cross-validation to evaluate the performance and effectiveness of feature selection. The table shows the best features (in the “best” column) and classification error rates (in the “ce” column) obtained after feature selection by different RFE models. [file Data_Sheet_11.pdf]

**Testing Set DCA (Undersampled)**

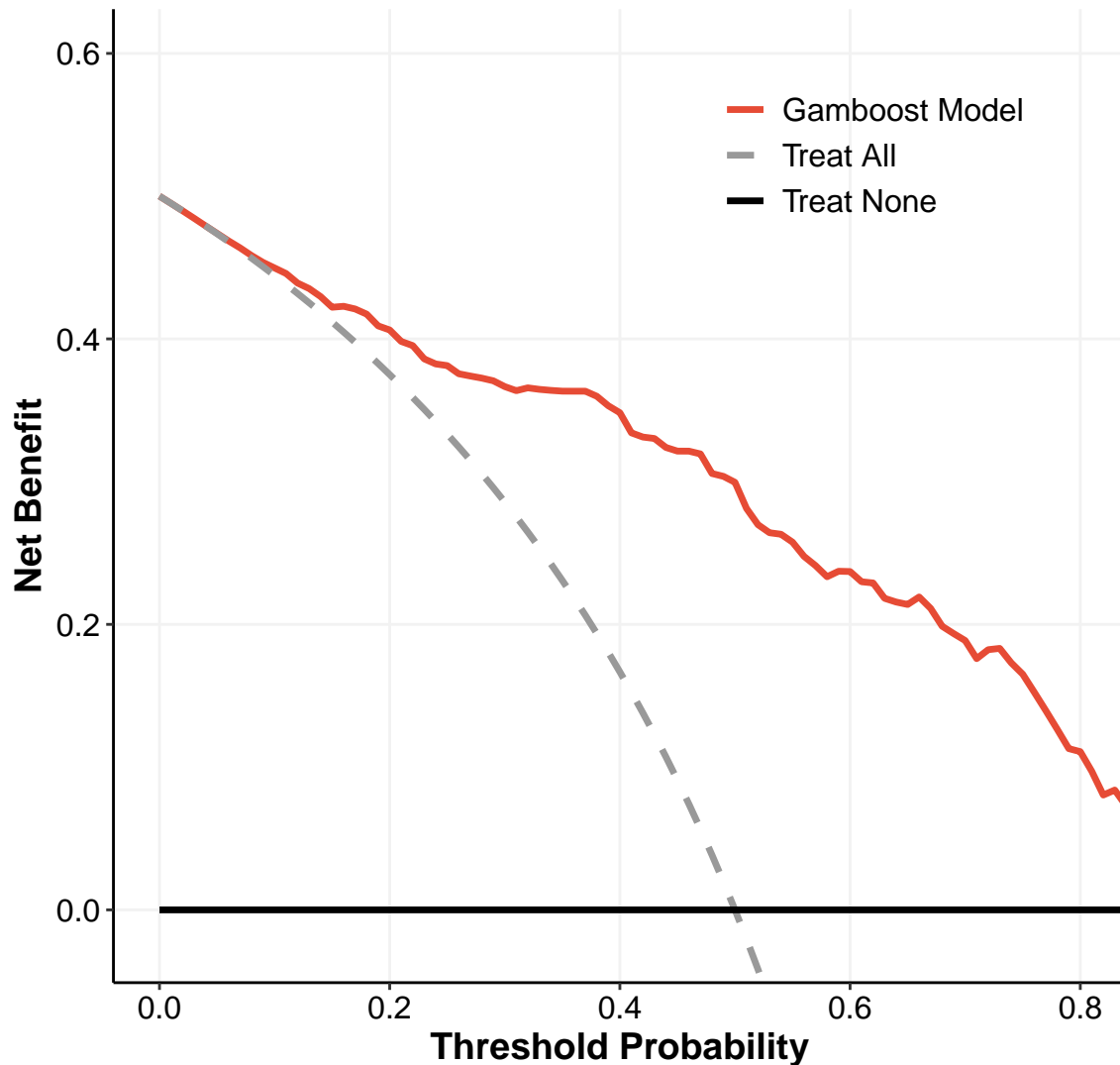

**External Validation DCA (Prevalence Calibrated)**

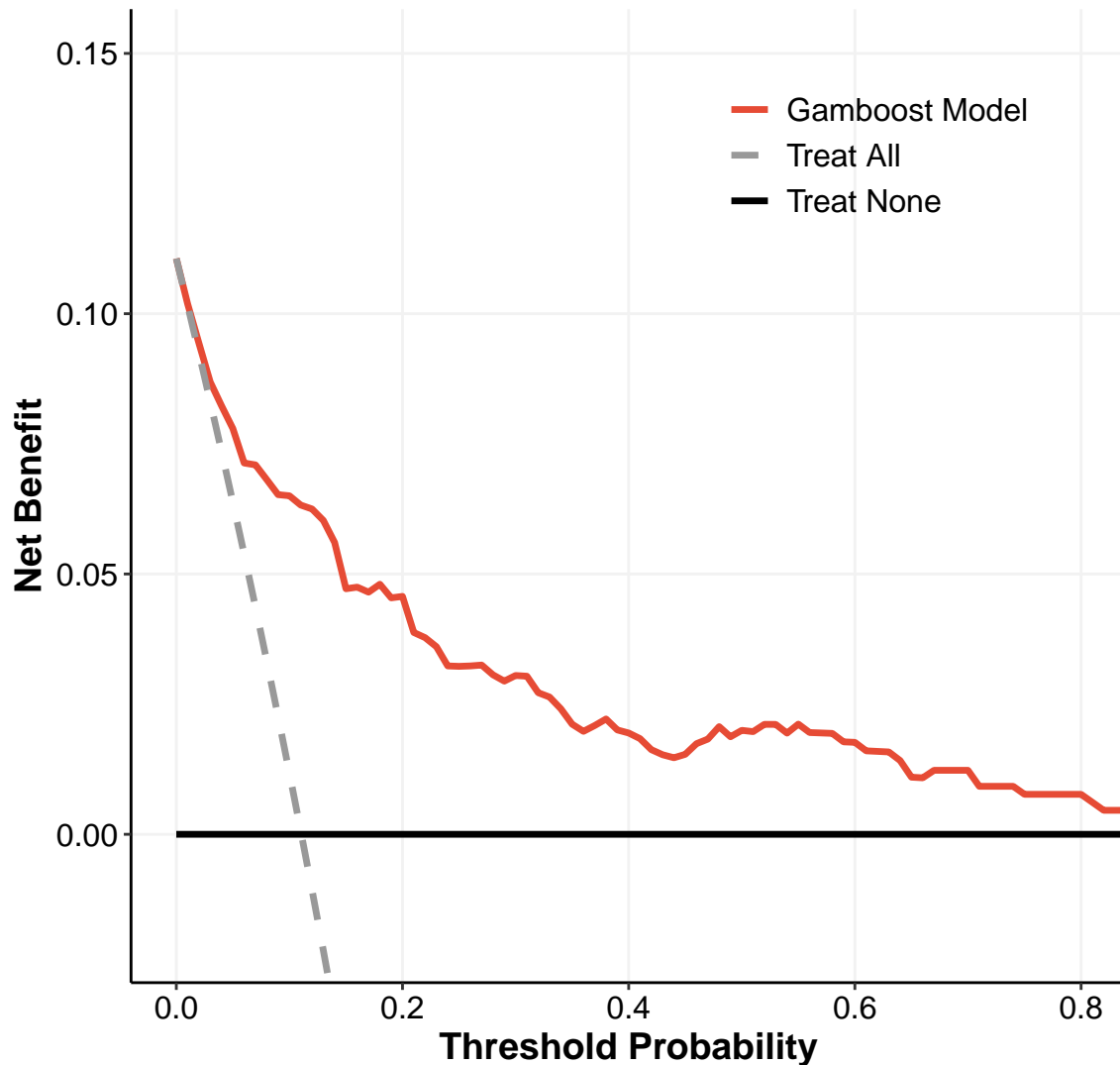

Supplement: Supplementary file 14 — Decision curve analysis (DCA) evaluating the clinical utility of the Gamboost model. The graphs depict the net clinical benefit of utilizing the Gamboost predictive framework (red solid line) to guide interventions, compared against the default strategies of treating all patients (dashed grey line) or treating no patients (solid black line) across a continuum of threshold probabilities. (A) Internal Testing Set. In the 1:1 undersampled internal cohort, the Gamboost model demonstrated a substantial net clinical benefit over default strategies across the entire spectrum of risk thresholds. (B) External Validation Cohort. DCA was conducted after applying a Bayesian prior-correction (prevalence calibration) to strictly reflect the real-world incidence rate of advanced liver fibrosis in the hospital setting. The recalibrated Gamboost model consistently yielded a superior net clinical benefit across clinically relevant threshold probabilities. This confirms that implementing the Gamboost model for non-invasive risk stratification provides tangible, real-world value for clinical decision-making. [file Data_Sheet_14.pdf]

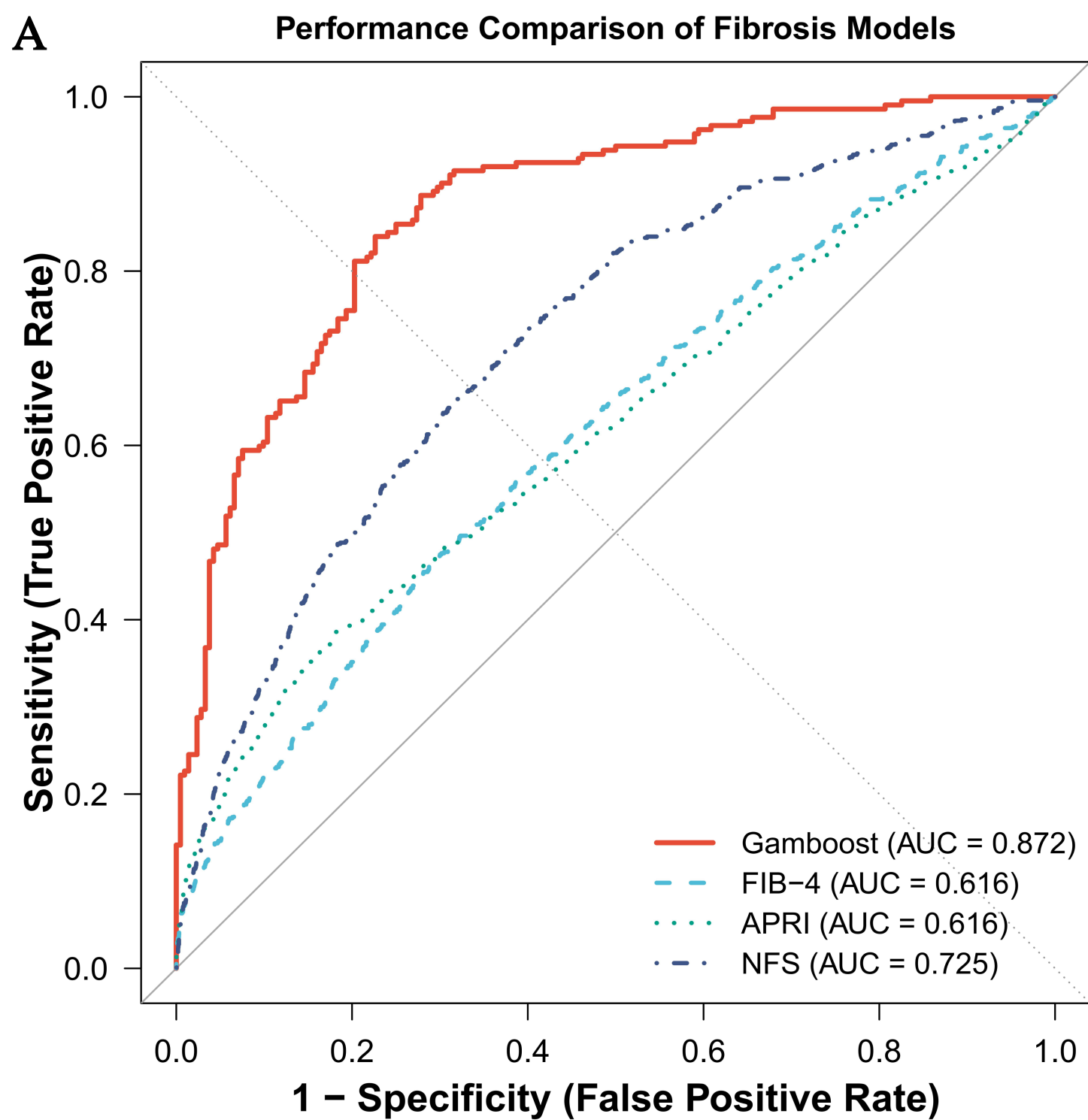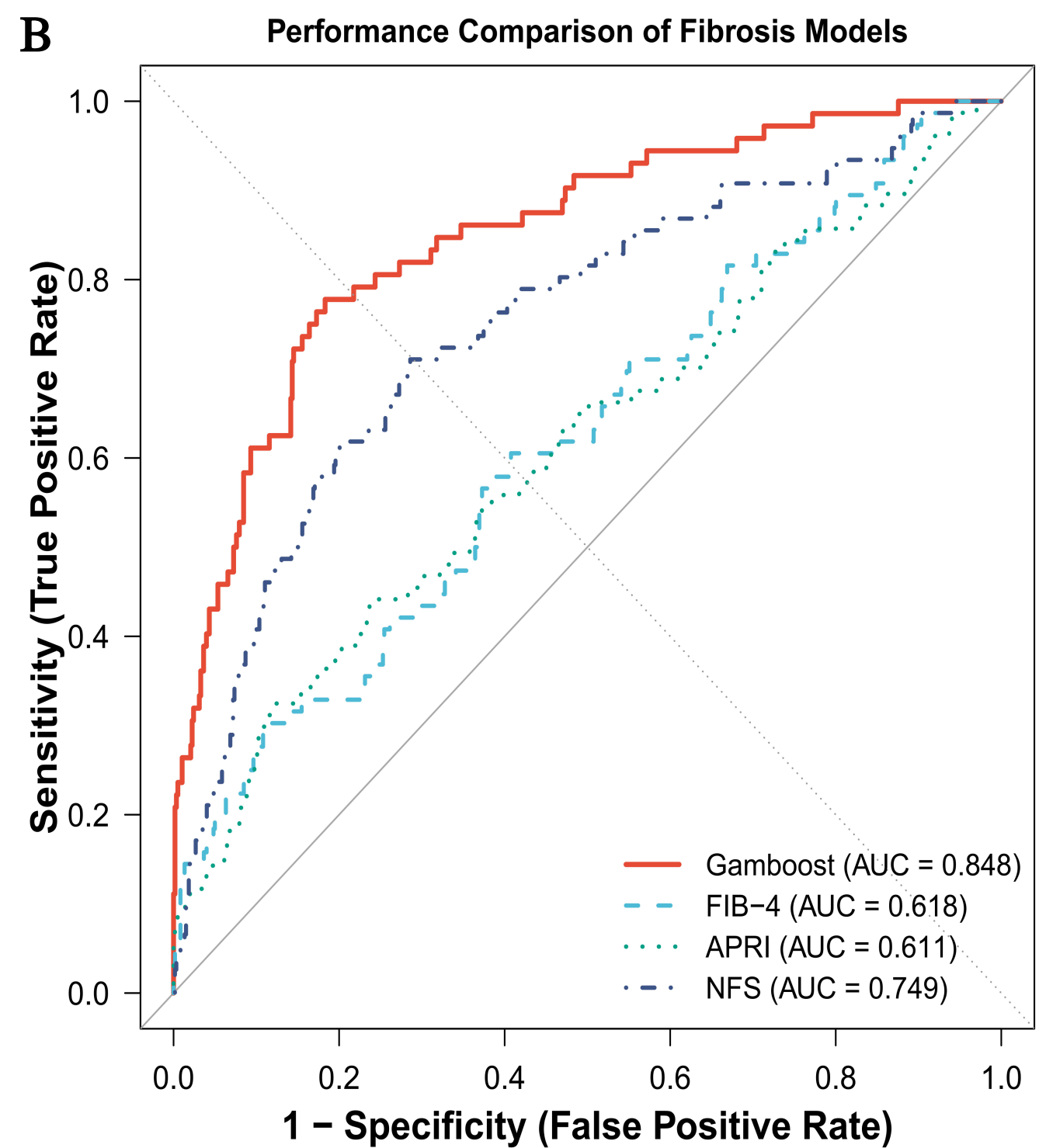

Supplement: Supplementary file 15 — Head-to-head comparison of discriminative performance between the Gamboost framework and traditional non-invasive fibrosis scores. Receiver operating characteristic curves illustrate the predictive accuracy of the Gamboost model compared against FIB-4, APRI, and the NAFLD Fibrosis Score (NFS). (A) In the internal testing set, Gamboost (AUC = 0.872) significantly outperformed all traditional indices (FIB-4: 0.616; APRI: 0.616; NFS: 0.725). (B) In the independent external validation cohort, the Gamboost model maintained robust predictive superiority (AUC = 0.848) over FIB-4 (0.618), APRI (0.611), and NFS (0.749), confirming its exceptional cross-cohort generalizability and clinical utility. [file Data_Sheet_15.pdf]
