# Supplementary material for: Integrative and interpretable machine learning framework for early non-invasive detection of clinically significant liver fibrosis
Source: Front Med (Lausanne). 2026 Jun 23;13:1736295. doi: 10.3389/fmed.2026.1736295 (PMC13337473; doi:10.3389/fmed.2026.1736295)
Supplement: Supplementary file 2 — Contemporary etiological distribution of the external validation cohort based on the 2023 Steatotic Liver Disease (SLD) criteria. Patients with clinically significant liver fibrosis in the real-world hospital cohort were retrospectively re-evaluated using the latest AASLD/EASL Delphi consensus. The disease spectrum was predominantly driven by Pure MASLD (55.1%, n = 43) and Alcohol-Associated Liver Disease (ALD; 15.4%, n = 12), with the newly defined MetALD accounting for 1.3% (n = 1). Owing to the inherent limitations of retrospective electronic medical records—specifically the absence of precise quantitative alcohol consumption logs required for strict contemporary subtyping-−28.2% (n = 22) of the participants were categorized as other/unclassified. [file Data_Sheet_2.pdf]

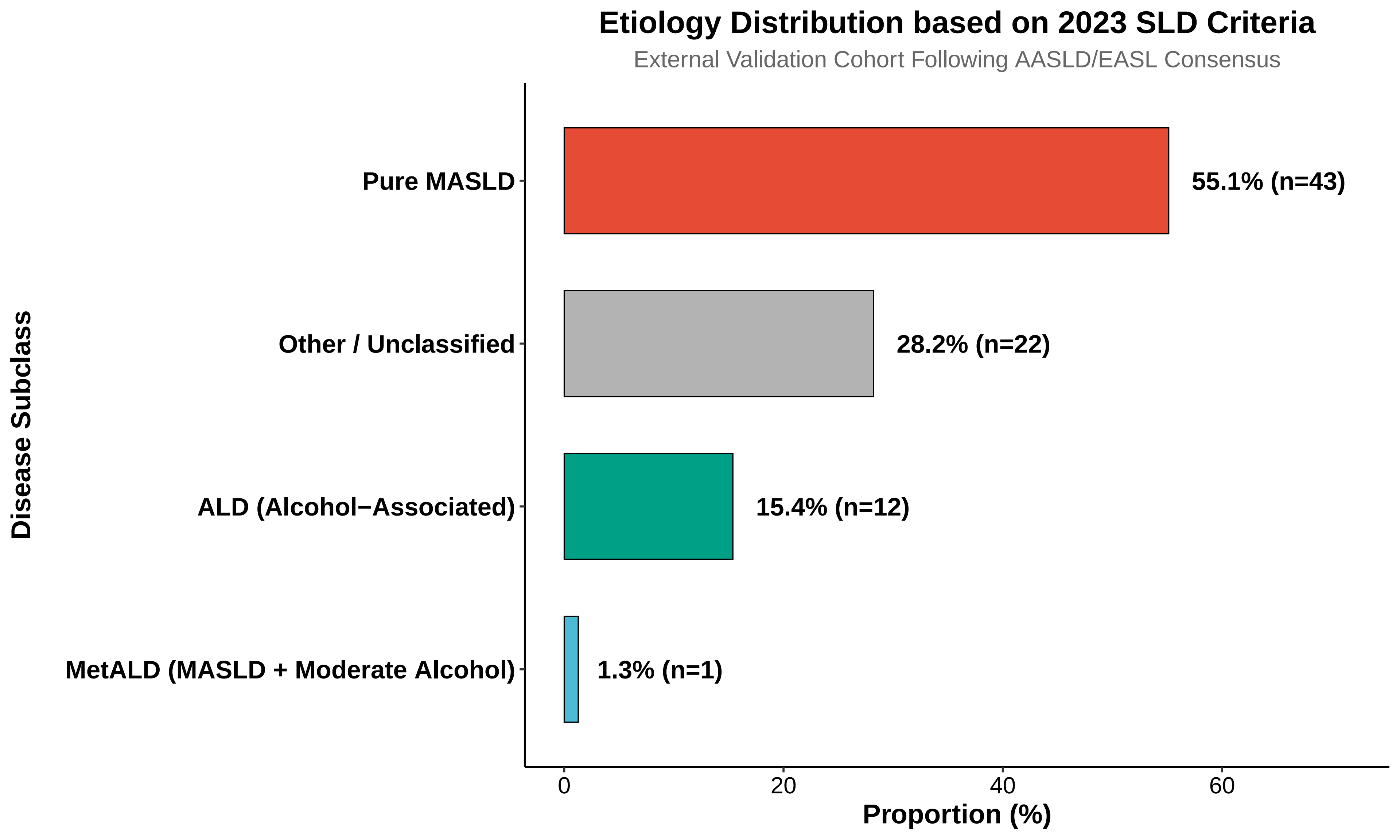

Etiology Distribution based on 2023 SLD Criteria
External Validation Cohort Following AASLD/EASL Consensus
Disease Subclass
Pure MASLD
55.1% (n=43)
Other / Unclassified
28.2% (n=22)
ALD (Alcohol−Associated)
15.4% (n=12)
MetALD (MASLD + Moderate Alcohol)
1.3% (n=1)
0
20
40
60
Proportion (%)
