# Supplementary material for: Integrative and interpretable machine learning framework for early non-invasive detection of clinically significant liver fibrosis
Source: Front Med (Lausanne). 2026 Jun 23;13:1736295. doi: 10.3389/fmed.2026.1736295 (PMC13337473; doi:10.3389/fmed.2026.1736295)
Supplement: Supplementary file 3 — The figure shows the percentage distribution of clinically significant liver fibrosis cases and control cases across different categories of each categorical variable. [file Data_Sheet_3.pdf]

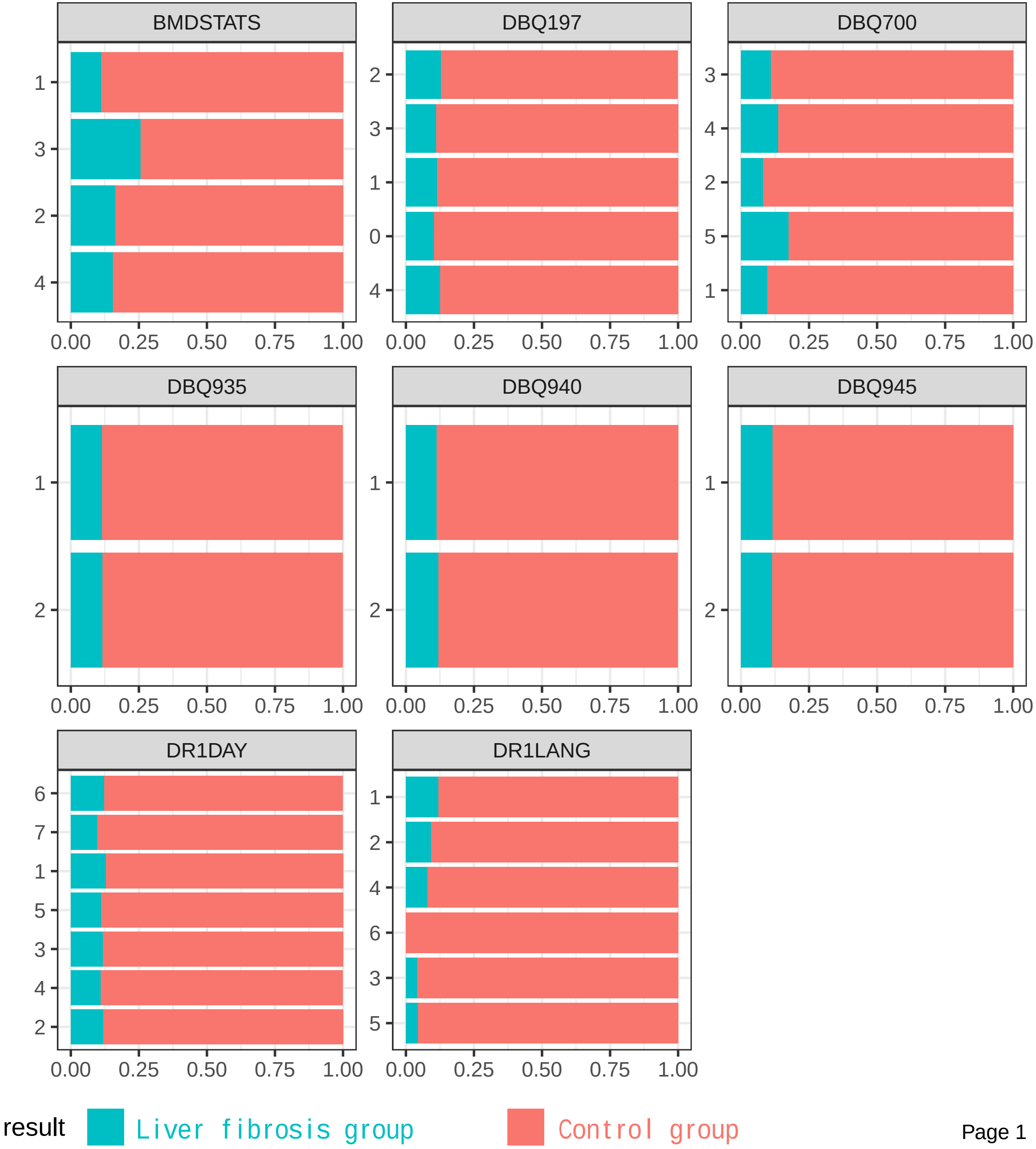
BMDSTATS
DBQ197
DBQ700
DBQ935
DBQ940
DBQ945
DR1DAY
DR1LANG
0.00 0.25 0.50 0.75 1.00
result
Liver fibrosis group
Control group

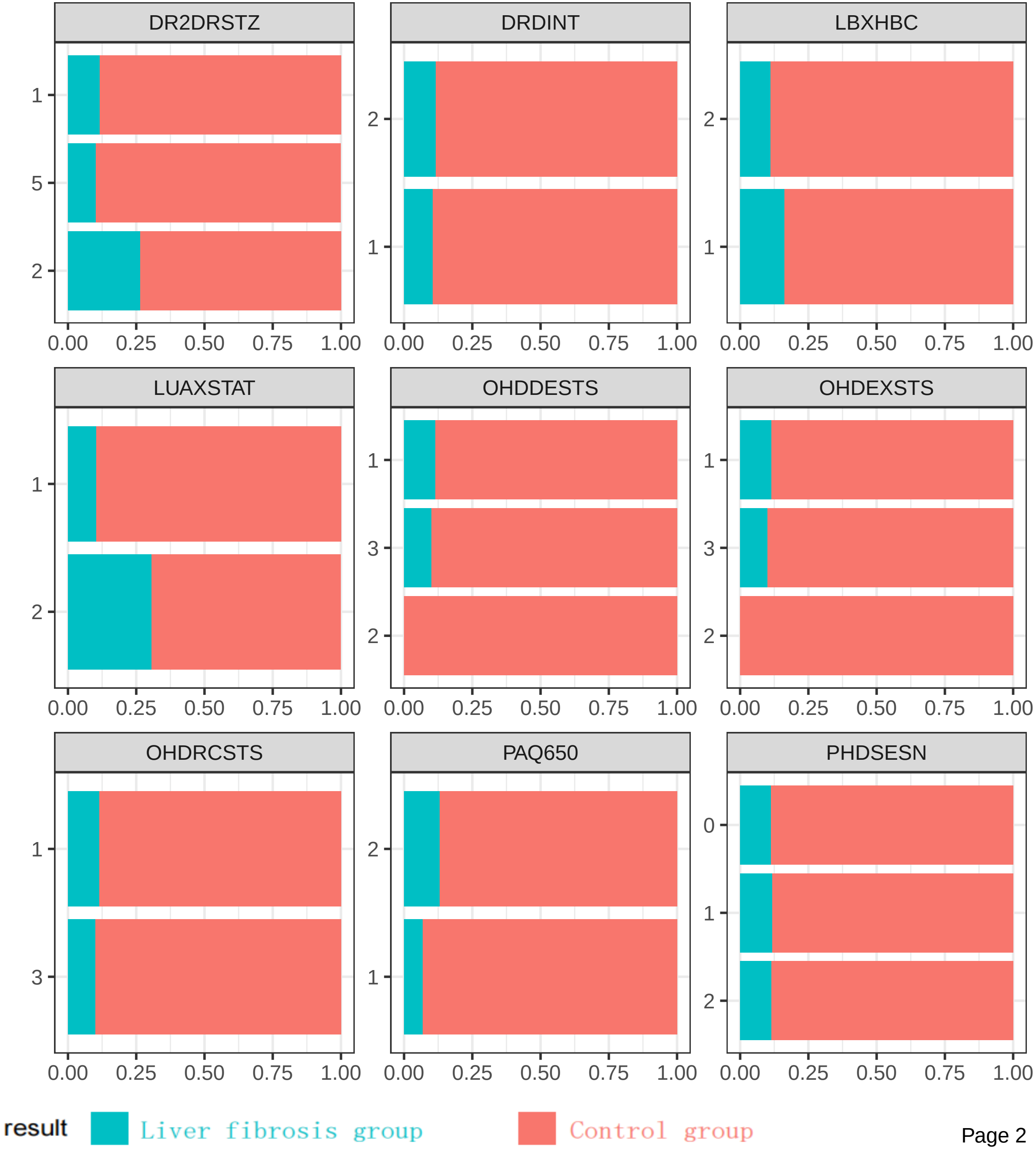
DR2DRSTZ
DRDINT
LBXHBC
LUAXSTAT
OHDDESTS
OHDEXSTS
OHDRCSTS
PAQ650
PHDSESN
0.00 0.25 0.50 0.75 1.00
result
Liver fibrosis group
Control group

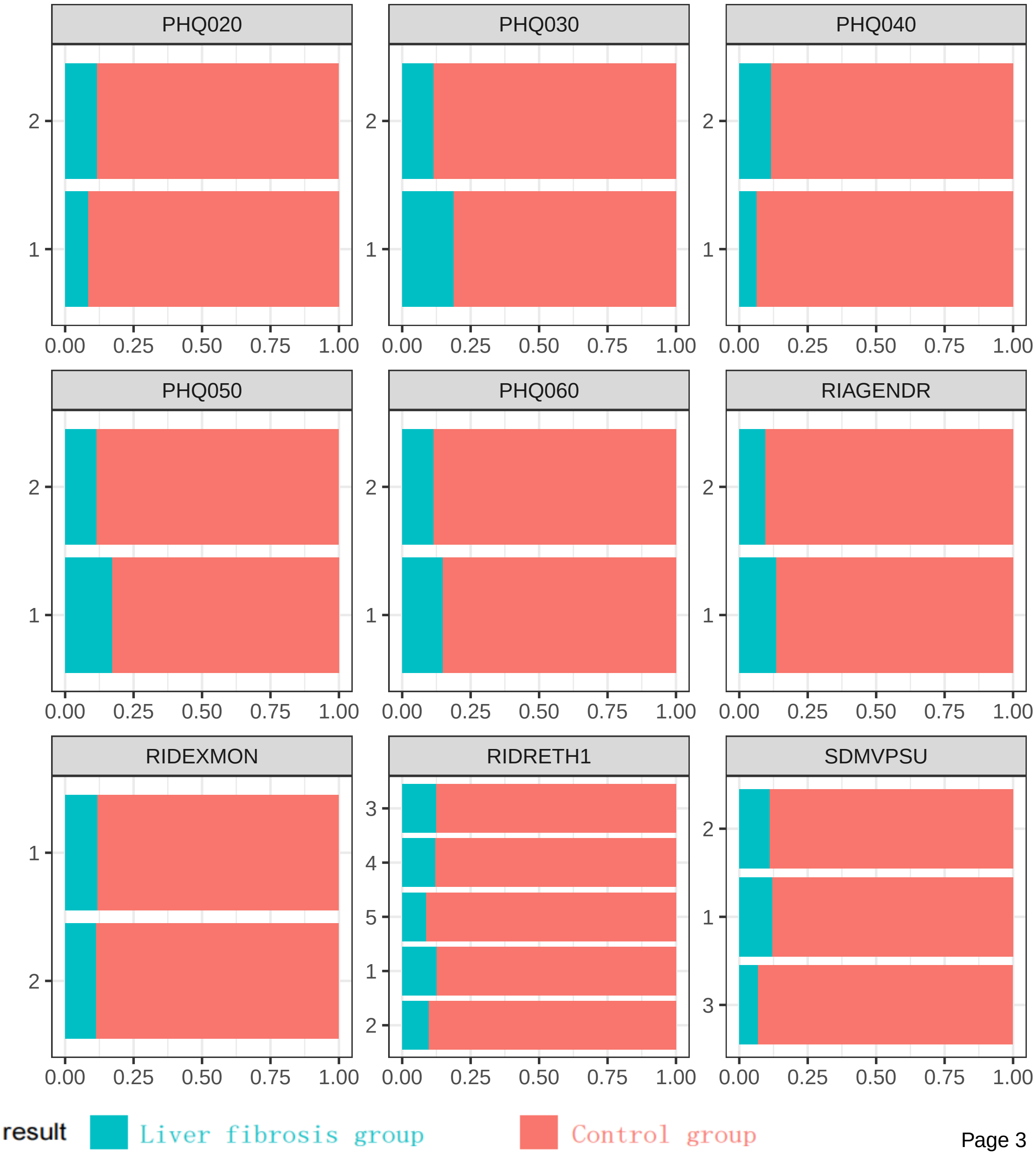
PHQ020
PHQ030
PHQ040
PHQ050
PHQ060
RIAGENDR
RIDEXMON
RIDRETH1
SDMVPSU
0.00
0.25
0.50
0.75
1.00
result
Liver fibrosis group
Control group

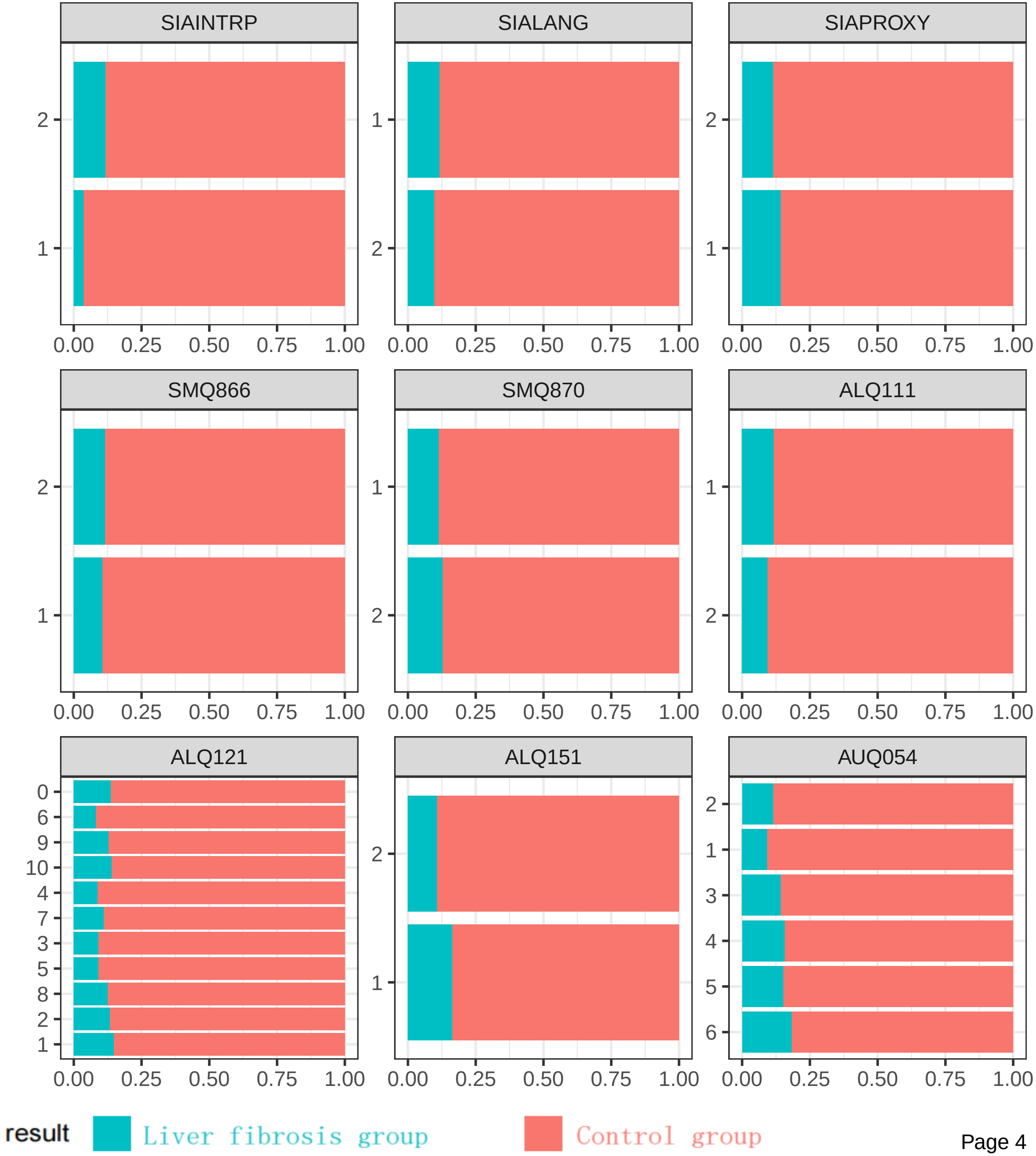
SIAINTRP
SIALANG
SIAPROXY
SMQ866
SMQ870
ALQ111
ALQ121
ALQ151
AUQ054
0.00
0.25
0.50
0.75
1.00
result
Liver fibrosis group
Control group

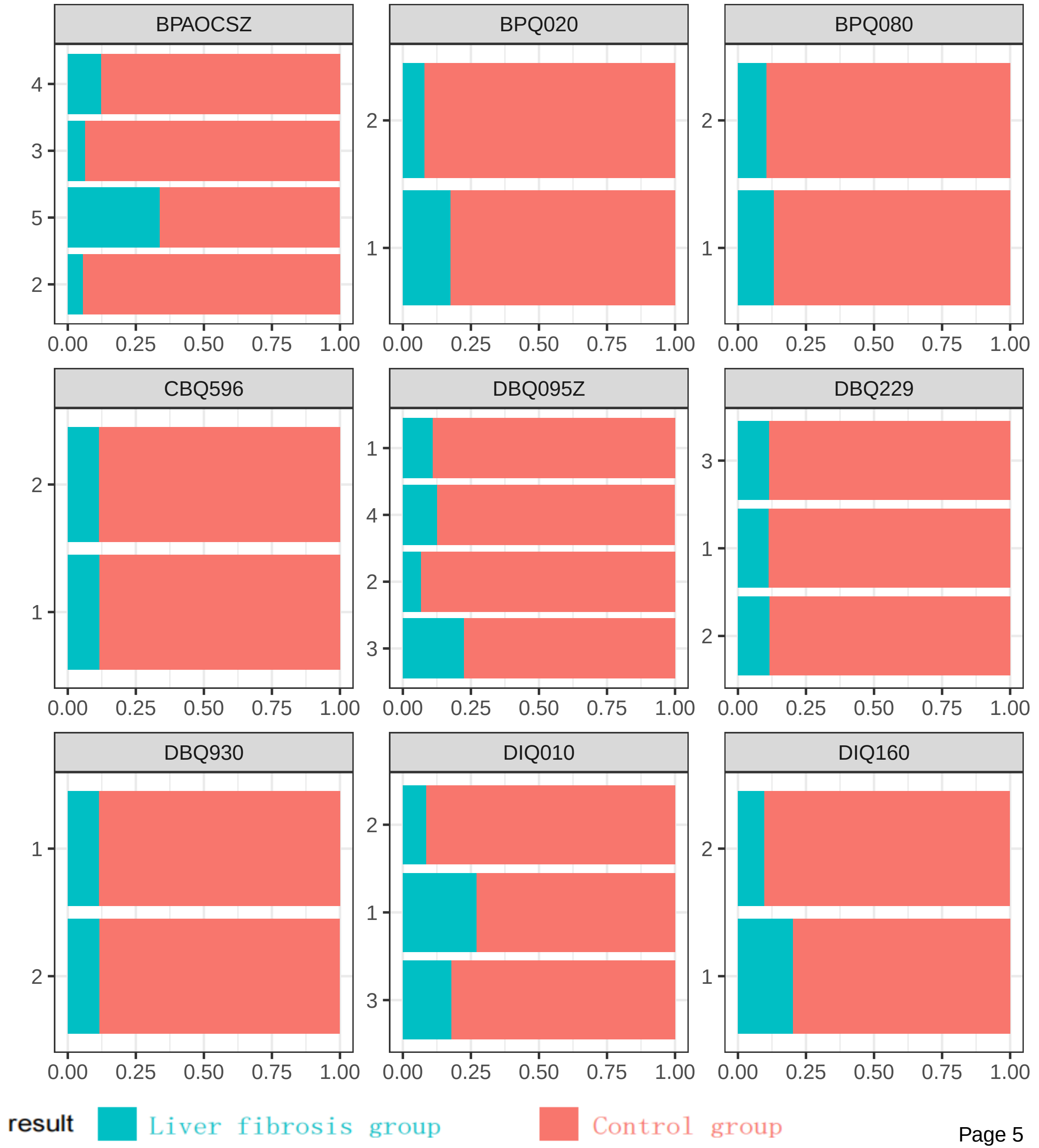
BPAOCSZ
BPQ020
BPQ080
CBQ596
DBQ095Z
DBQ229
DBQ930
DIQ010
DIQ160
0.00
0.25
0.50
0.75
1.00
result
Liver fibrosis group
Control group

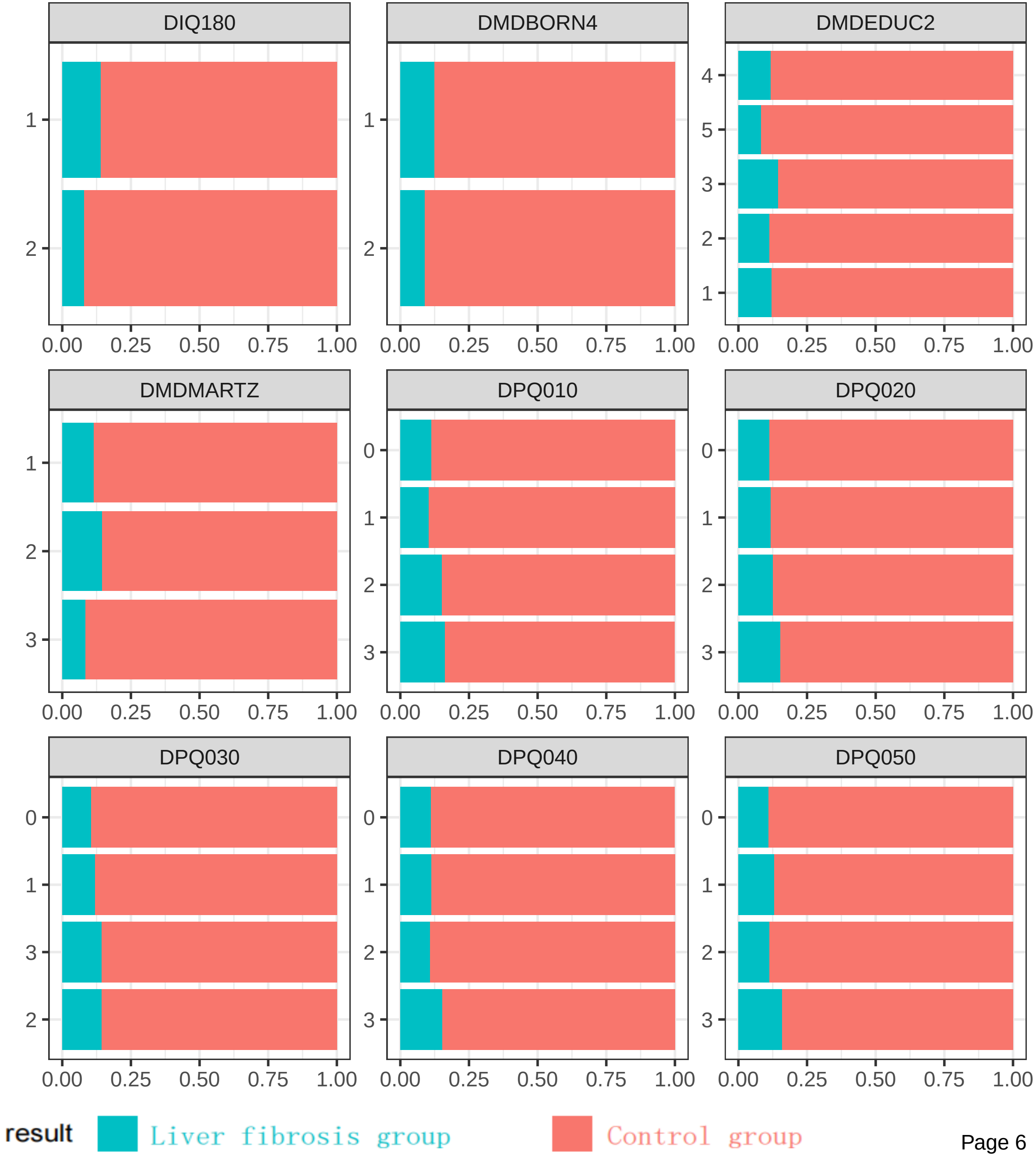
DIQ180
DMDBORN4
DMDEDUC2
DMDMARTZ
DPQ010
DPQ020
DPQ030
DPQ040
DPQ050
0.00
0.25
0.50
0.75
1.00
result
Liver fibrosis group
Control group

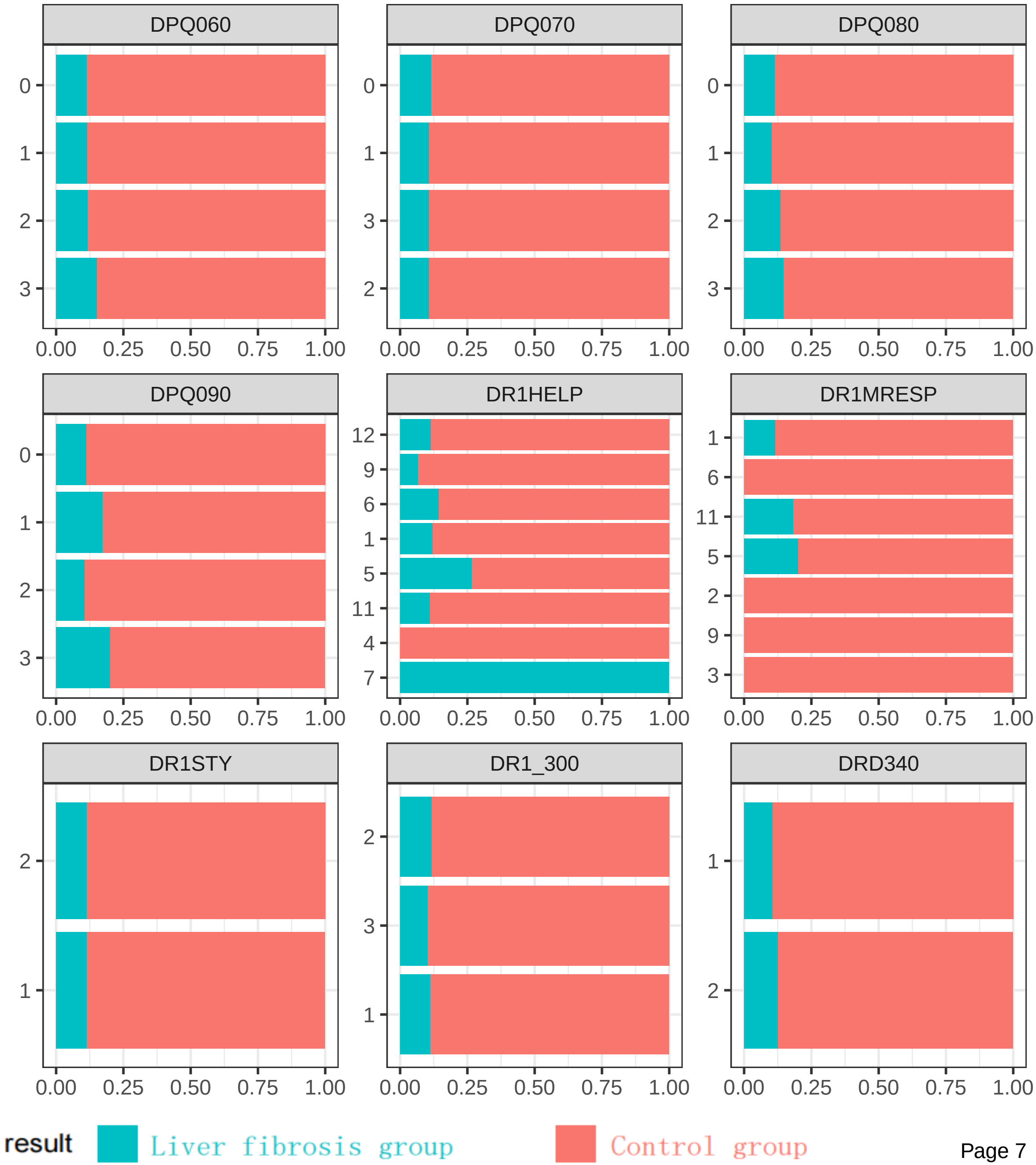
DPQ060
DPQ070
DPQ080
DPQ090
DR1HELP
DR1MRESP
DR1STY
DR1_300
DRD340
0.00 0.25 0.50 0.75 1.00
result
Liver fibrosis group
Control group

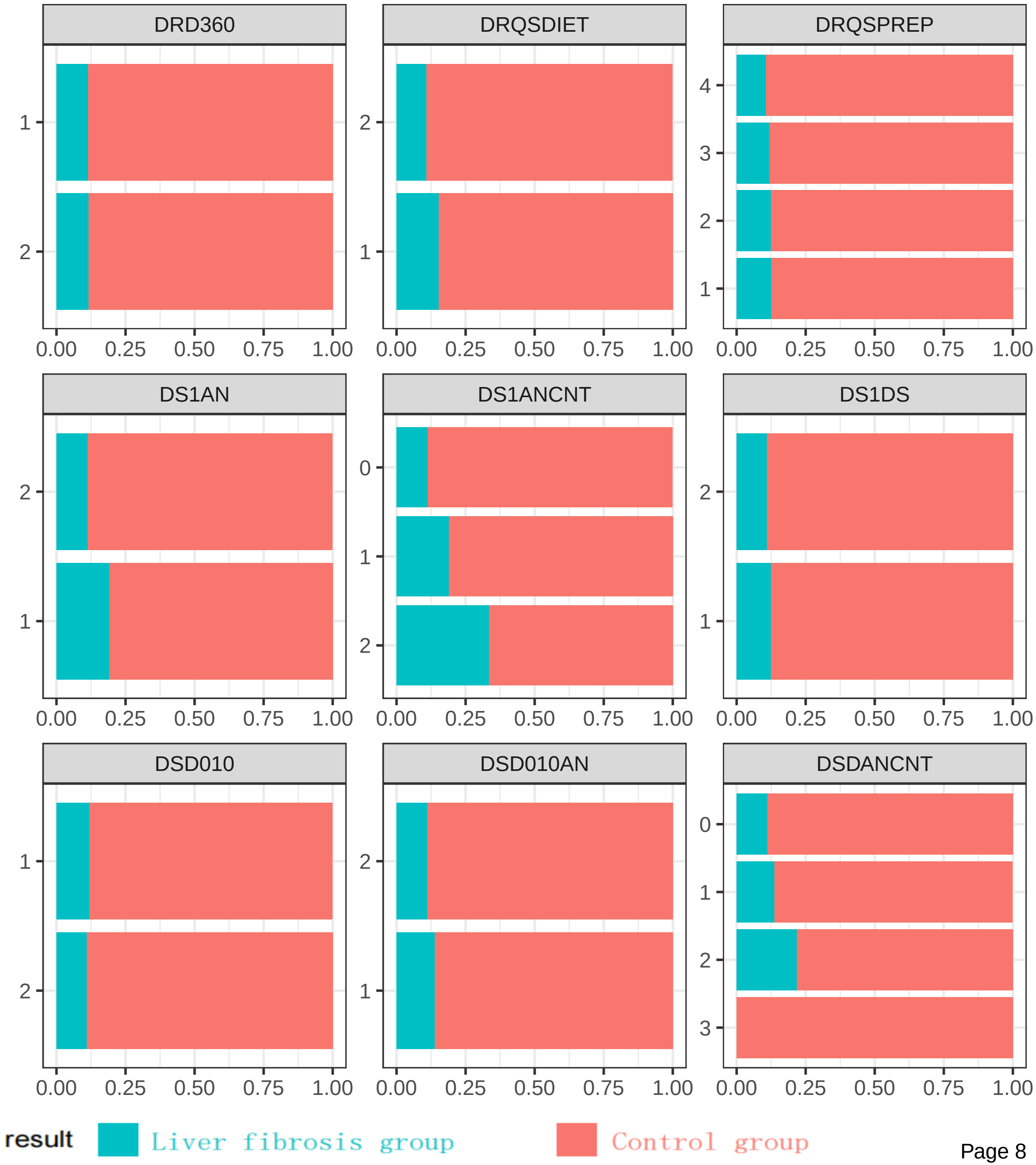
DRD360
DRQSDIET
DRQSPREP
DS1AN
DS1ANCNT
DS1DS
DSD010
DSD010AN
DSDANCNT
0.00 0.25 0.50 0.75 1.00
result
Liver fibrosis group
Control group

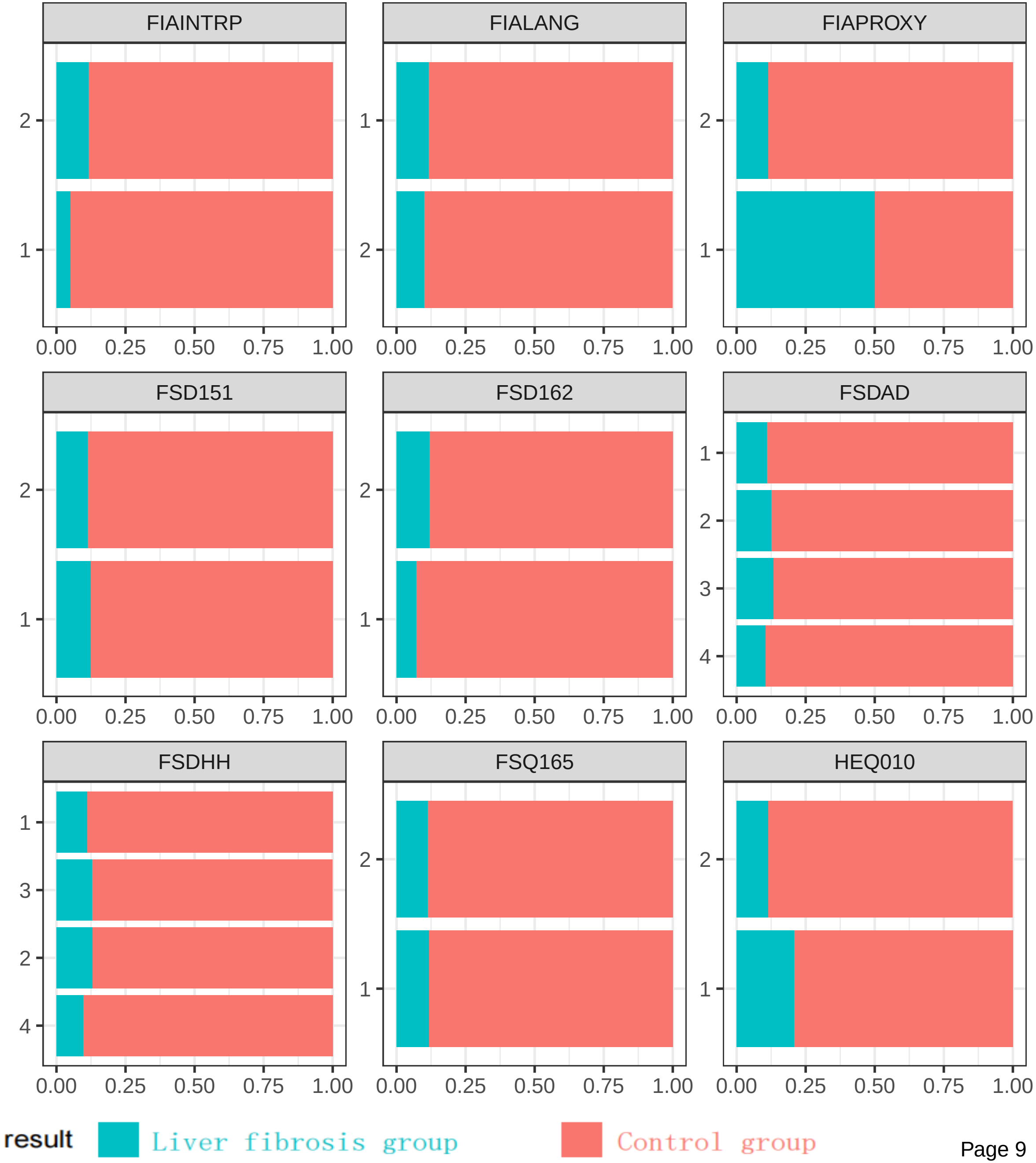
FIAINTRP
FIALANG
FIAPROXY
FSD151
FSD162
FSDAD
FSDHH
FSQ165
HEQ010
0.00
0.25
0.50
0.75
1.00
result
Liver fibrosis group
Control group

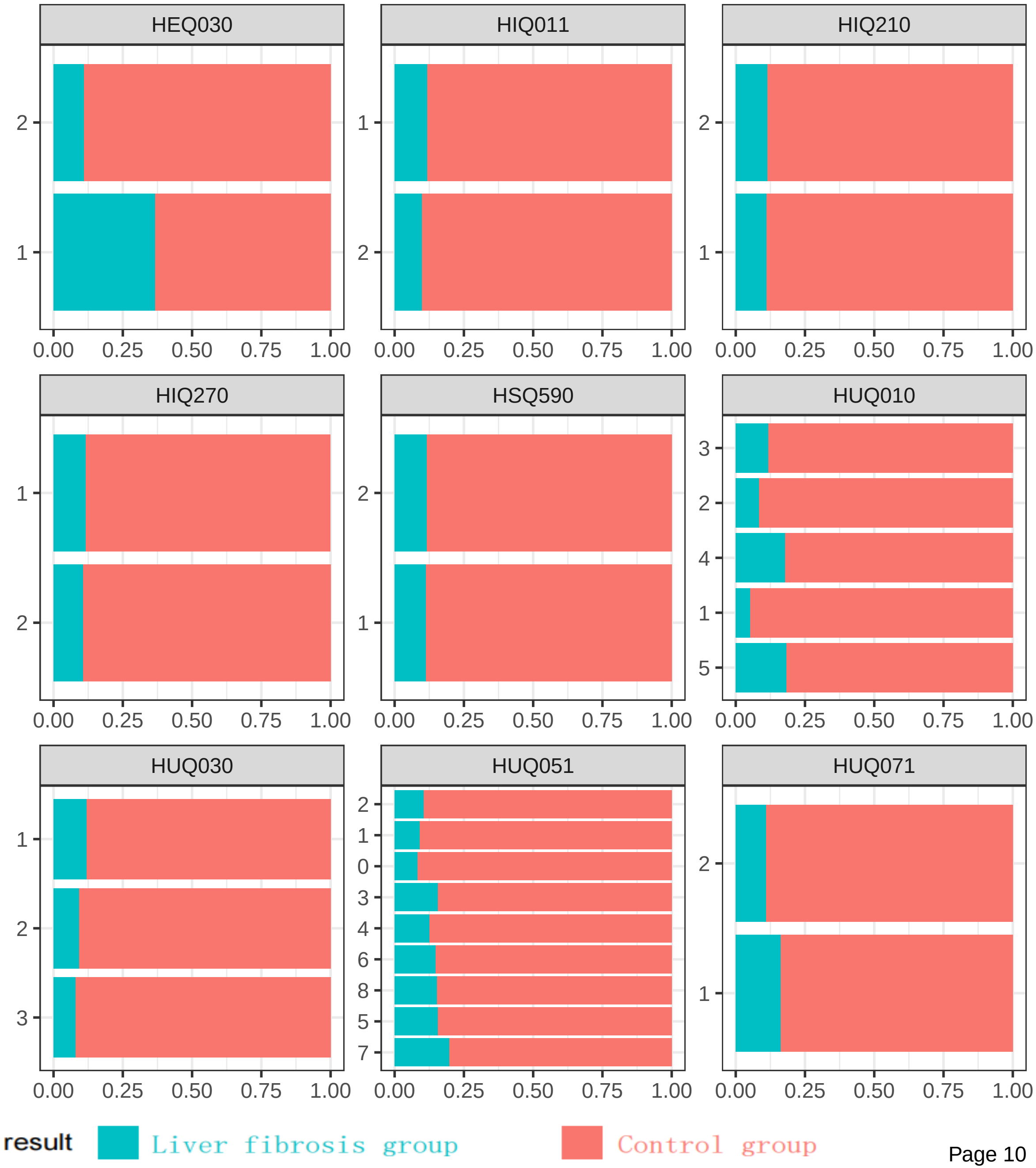
HEQ030
HIQ011
HIQ210
HIQ270
HSQ590
HUQ010
HUQ030
HUQ051
HUQ071
0.00
0.25
0.50
0.75
1.00
result
Liver fibrosis group
Control group

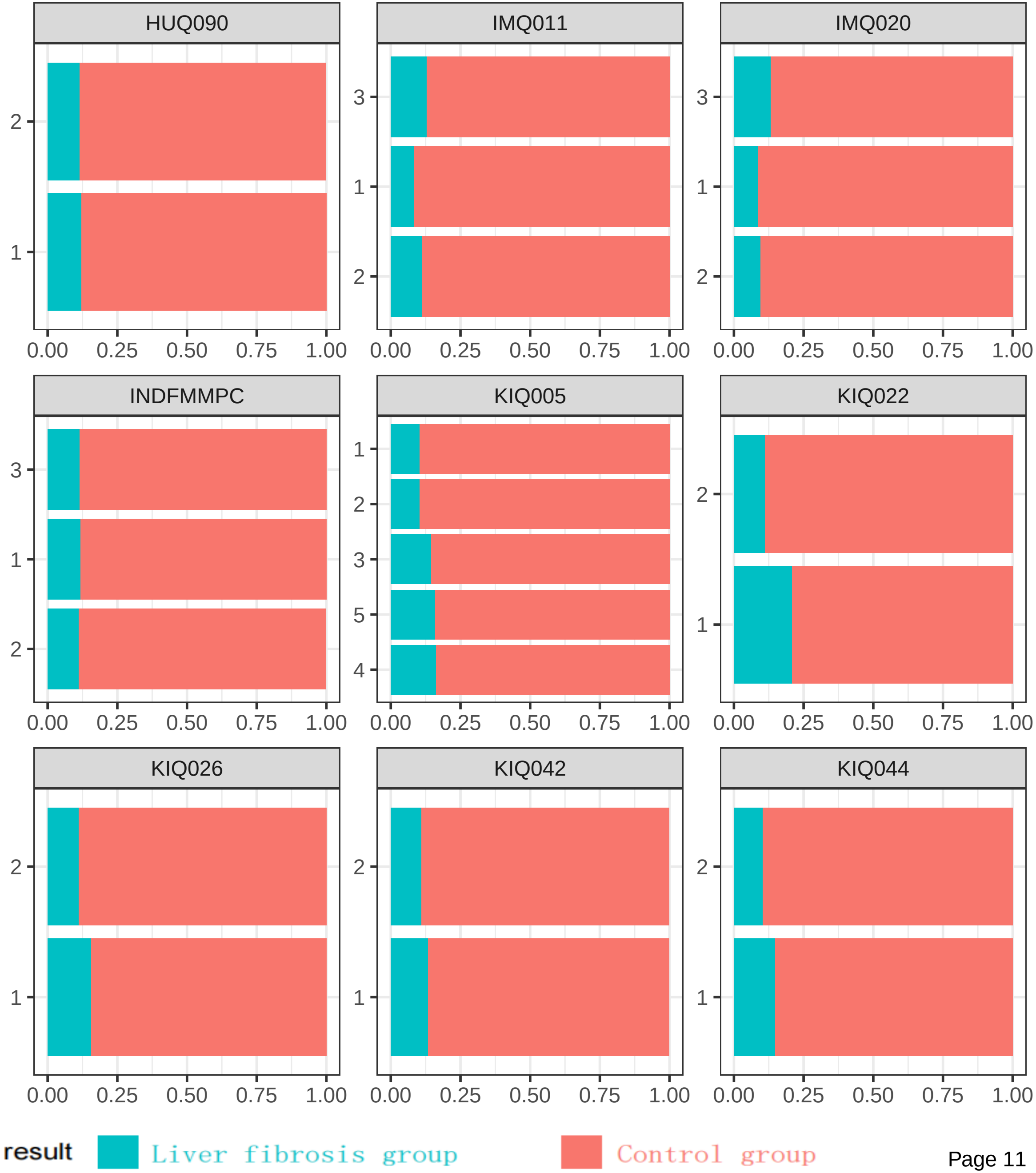
HUQ090
IMQ011
IMQ020
INDFMMPC
KIQ005
KIQ022
KIQ026
KIQ042
KIQ044
0.00
0.25
0.50
0.75
1.00
result
Liver fibrosis group
Control group

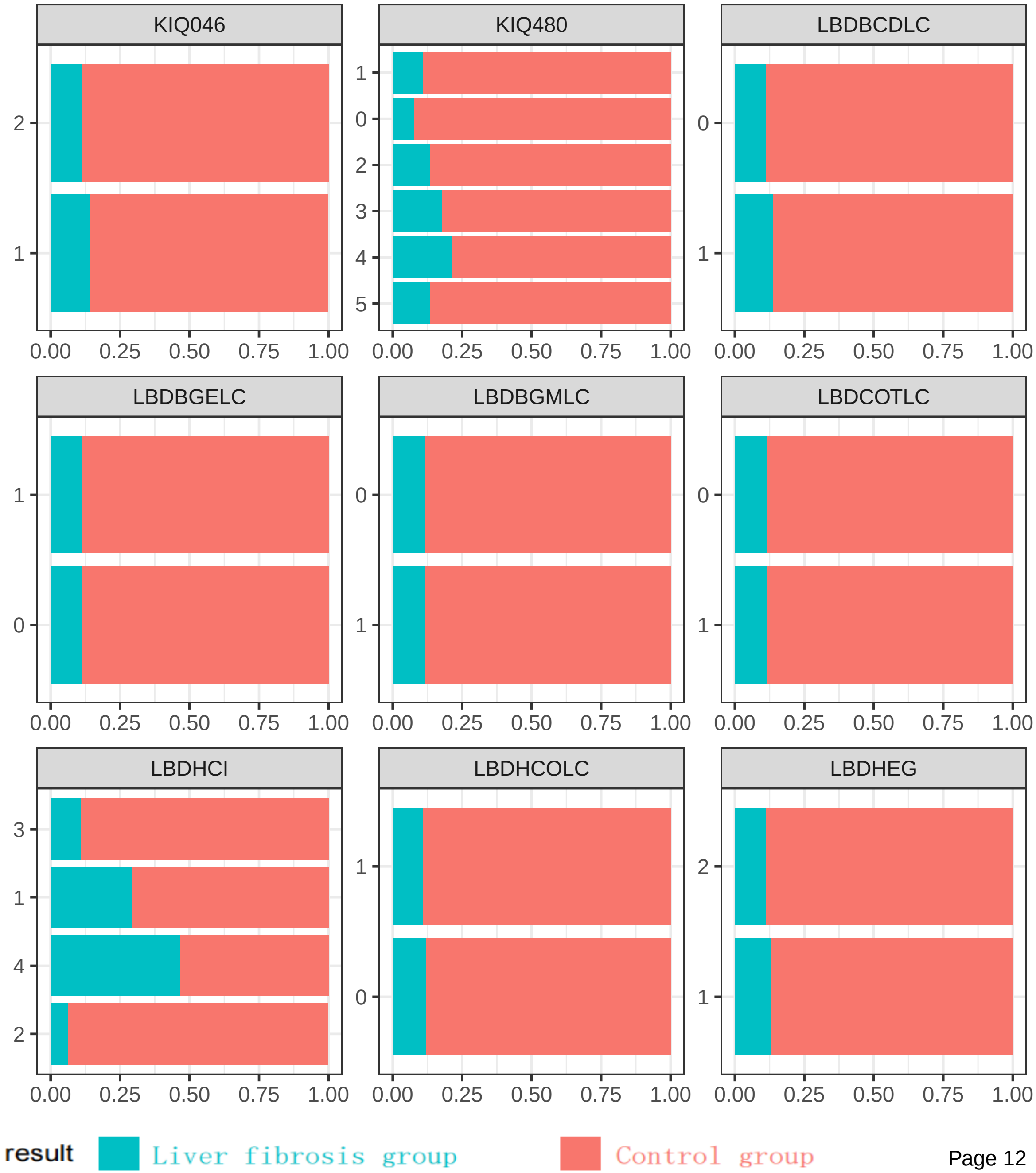

KIQ046
2
1
0.00 0.25 0.50 0.75 1.00
KIQ480
1
0
2
3
4
5
0.00 0.25 0.50 0.75 1.00
LBDBCDLC
0
1
0.00 0.25 0.50 0.75 1.00
LBDBGELC
1
0
0.00 0.25 0.50 0.75 1.00
LBDBGMLC
0
1
0.00 0.25 0.50 0.75 1.00
LBDCOTLC
0
1
0.00 0.25 0.50 0.75 1.00
LBDHCI
3
1
4
2
0.00 0.25 0.50 0.75 1.00
LBDHCOLC
1
0
0.00 0.25 0.50 0.75 1.00
LBDHEG
2
1
0.00 0.25 0.50 0.75 1.00
result
Liver fibrosis group
Control group

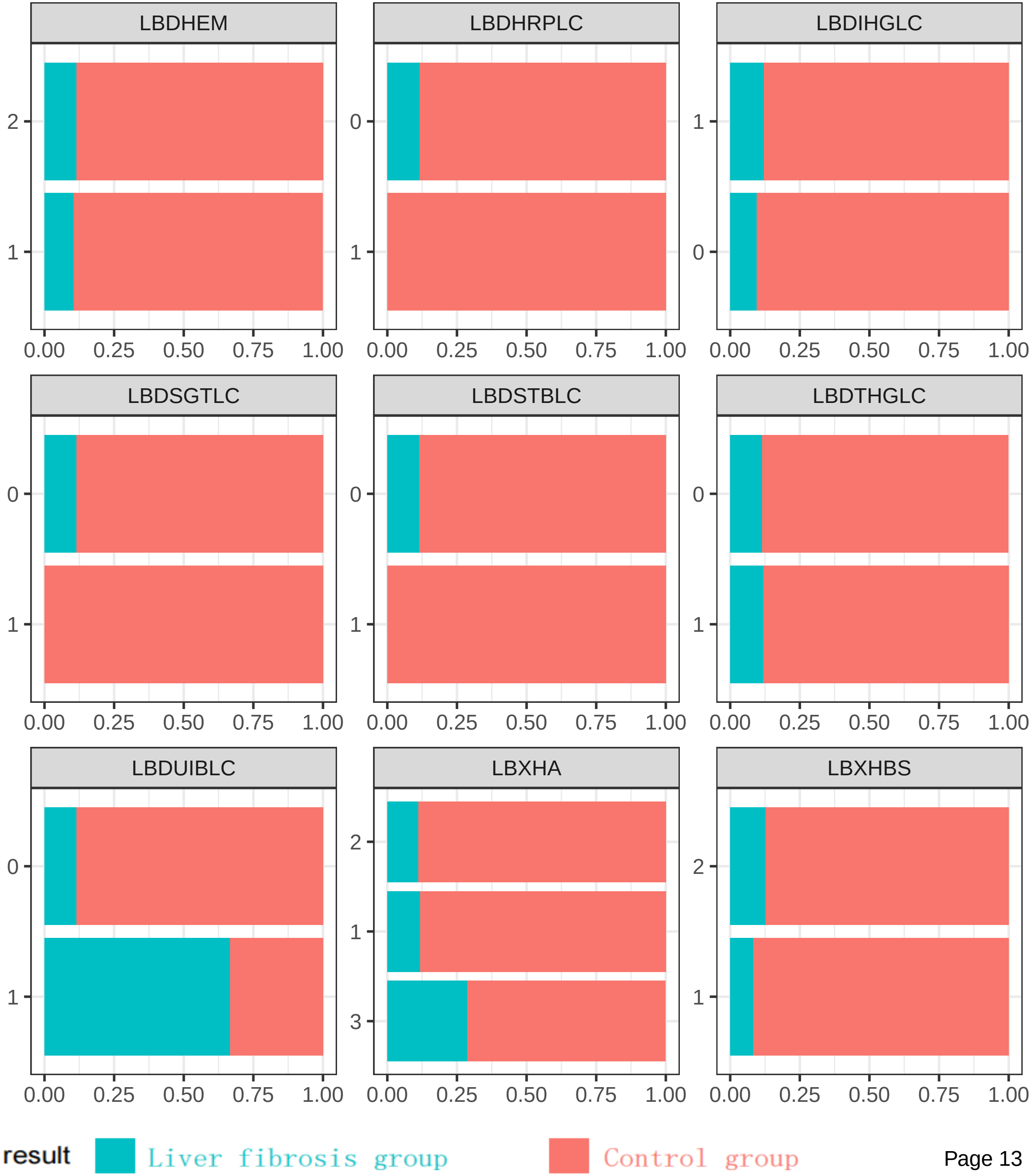
LBDHEM
LBDHRPLC
LBDIHGLC
LBDSGTLC
LBDSTBLC
LBDTHGLC
LBDUIBLC
LBXHA
LBXHBS
0.00
0.25
0.50
0.75
1.00
result
Liver fibrosis group
Control group

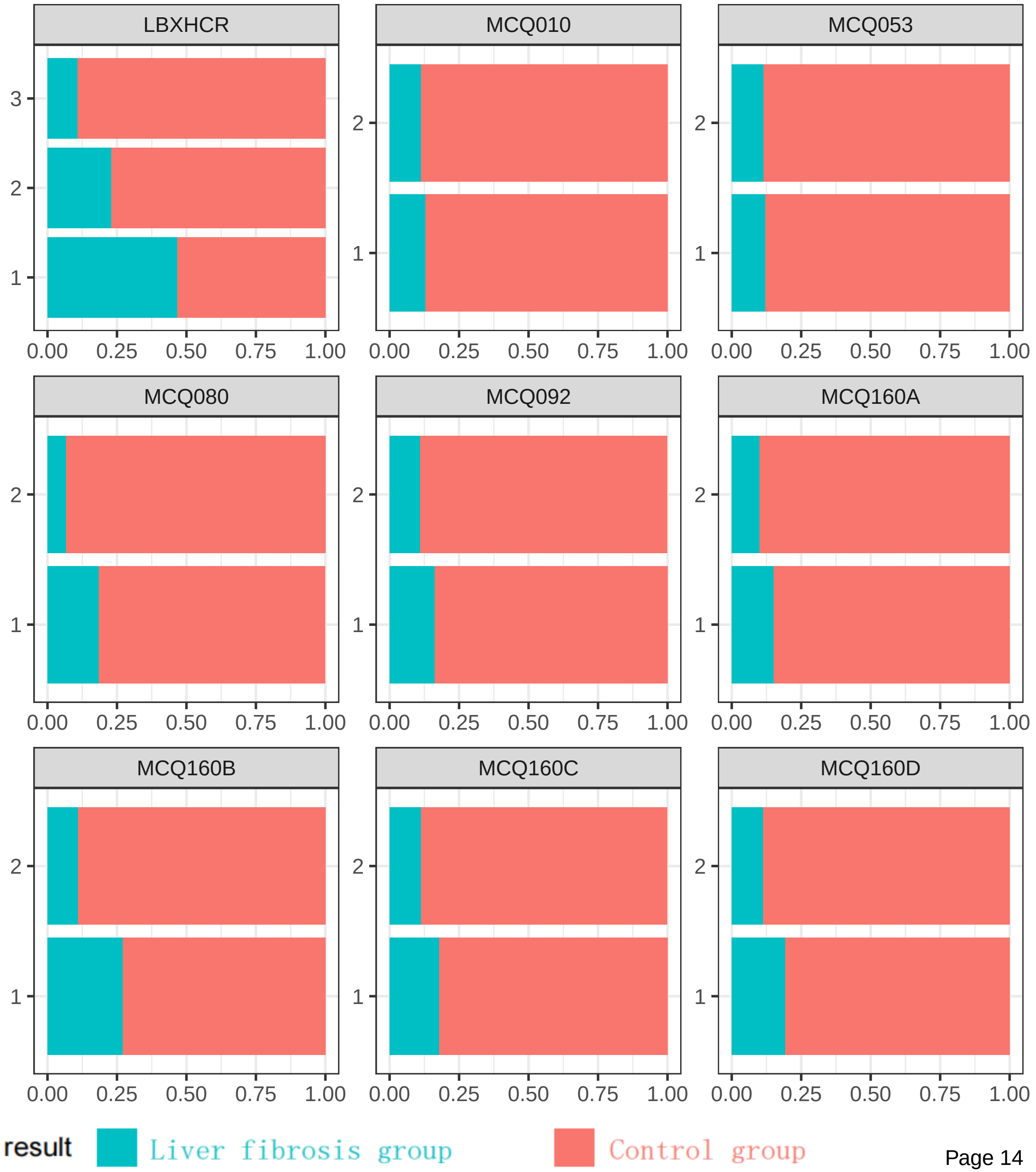
LBXHCR
MCQ010
MCQ053
MCQ080
MCQ092
MCQ160A
MCQ160B
MCQ160C
MCQ160D
0.00 0.25 0.50 0.75 1.00
result
Liver fibrosis group
Control group

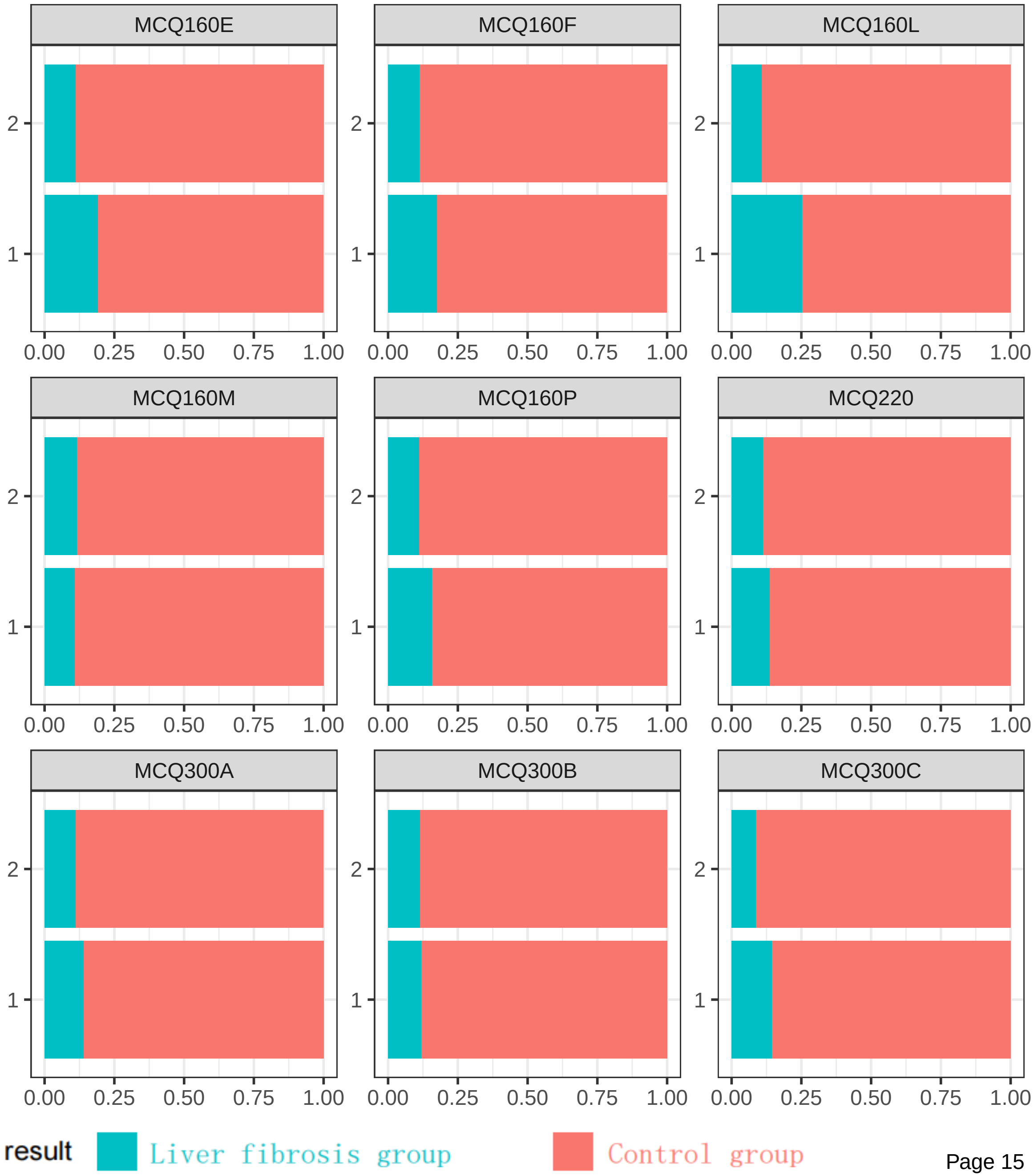
MCQ160E
MCQ160F
MCQ160L
MCQ160M
MCQ160P
MCQ220
MCQ300A
MCQ300B
MCQ300C
2
1
0.00
0.25
0.50
0.75
1.00
result
Liver fibrosis group
Control group

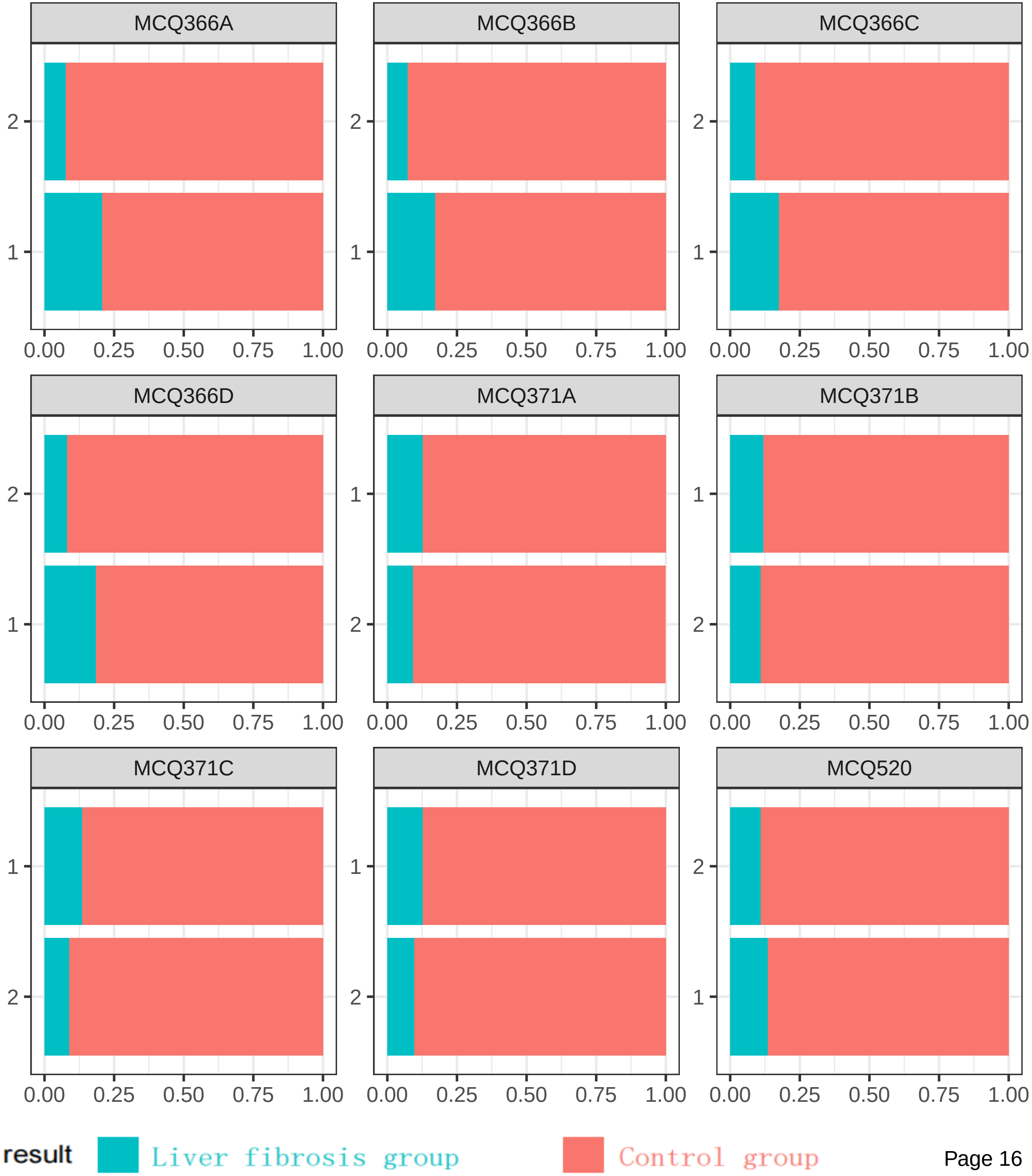
MCQ366A
MCQ366B
MCQ366C
MCQ366D
MCQ371A
MCQ371B
MCQ371C
MCQ371D
MCQ520
0.00
0.25
0.50
0.75
1.00
1
2
result
Liver fibrosis group
Control group

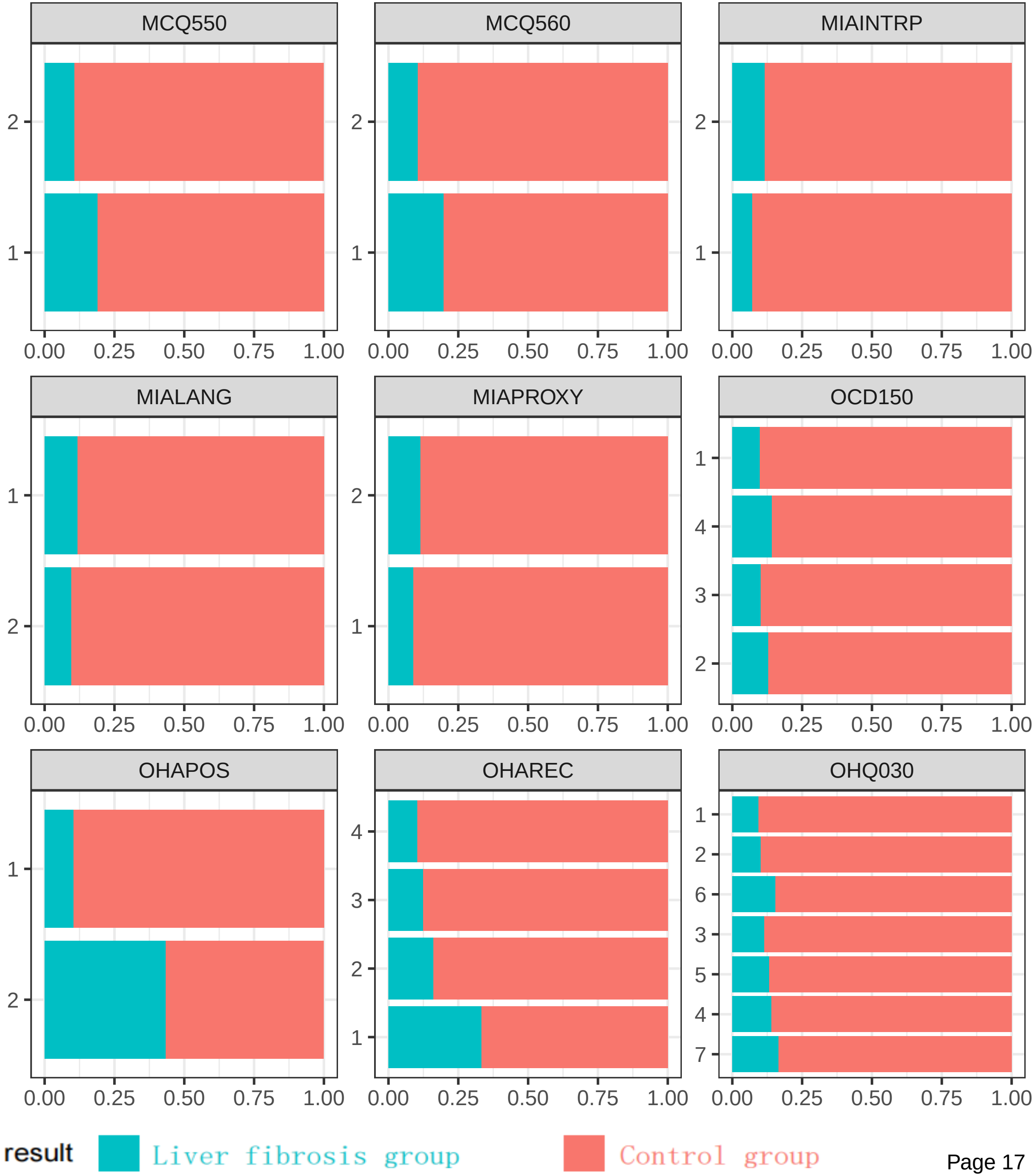
MCQ550
MCQ560
MIAINTRP
MIALANG
MIAPROXY
OCD150
OHAPOS
OHAREC
OHQ030
0.00
0.25
0.50
0.75
1.00
result
Liver fibrosis group
Control group

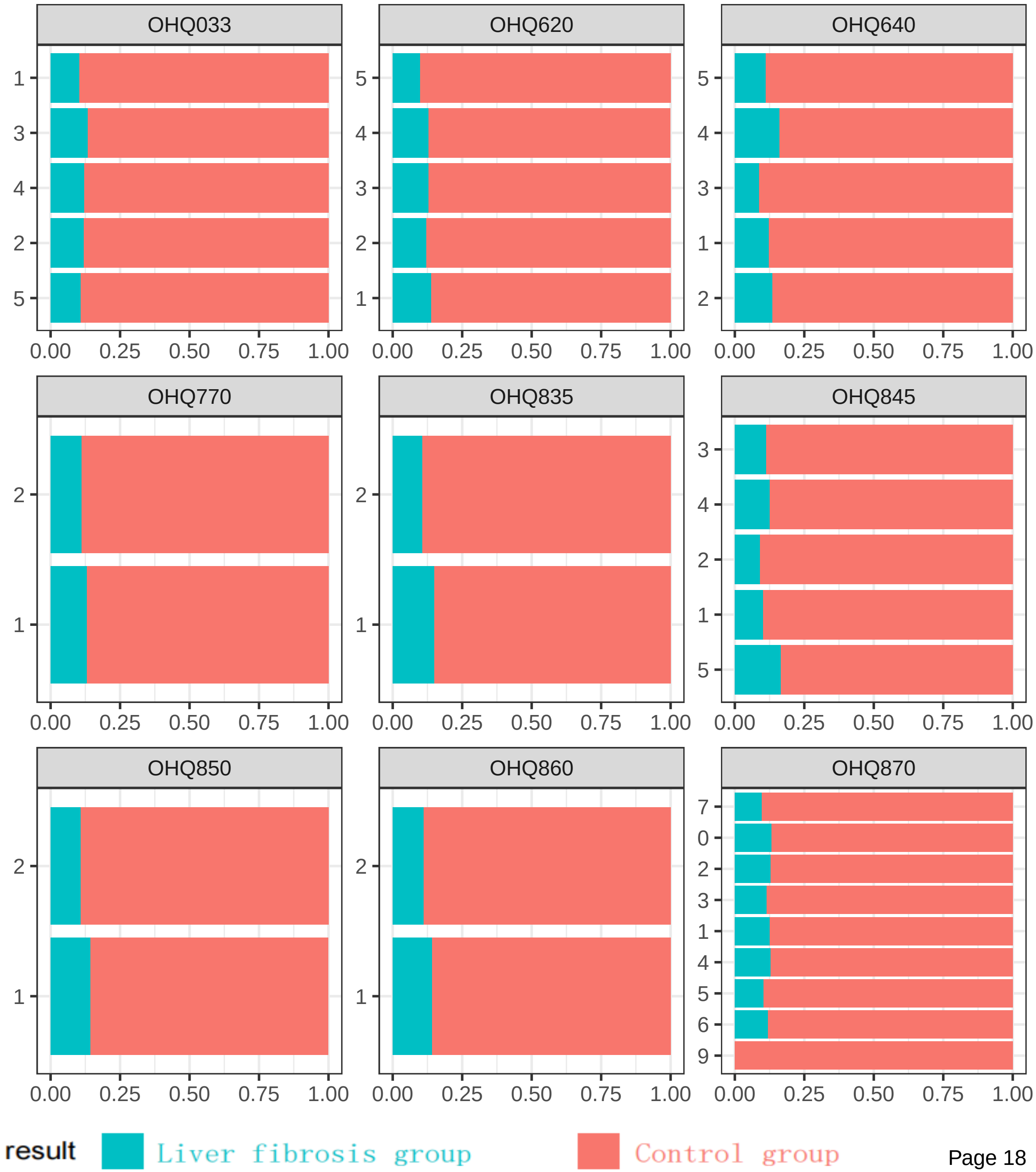
OHQ033
OHQ620
OHQ640
OHQ770
OHQ835
OHQ845
OHQ850
OHQ860
OHQ870
0.00 0.25 0.50 0.75 1.00
result
Liver fibrosis group
Control group

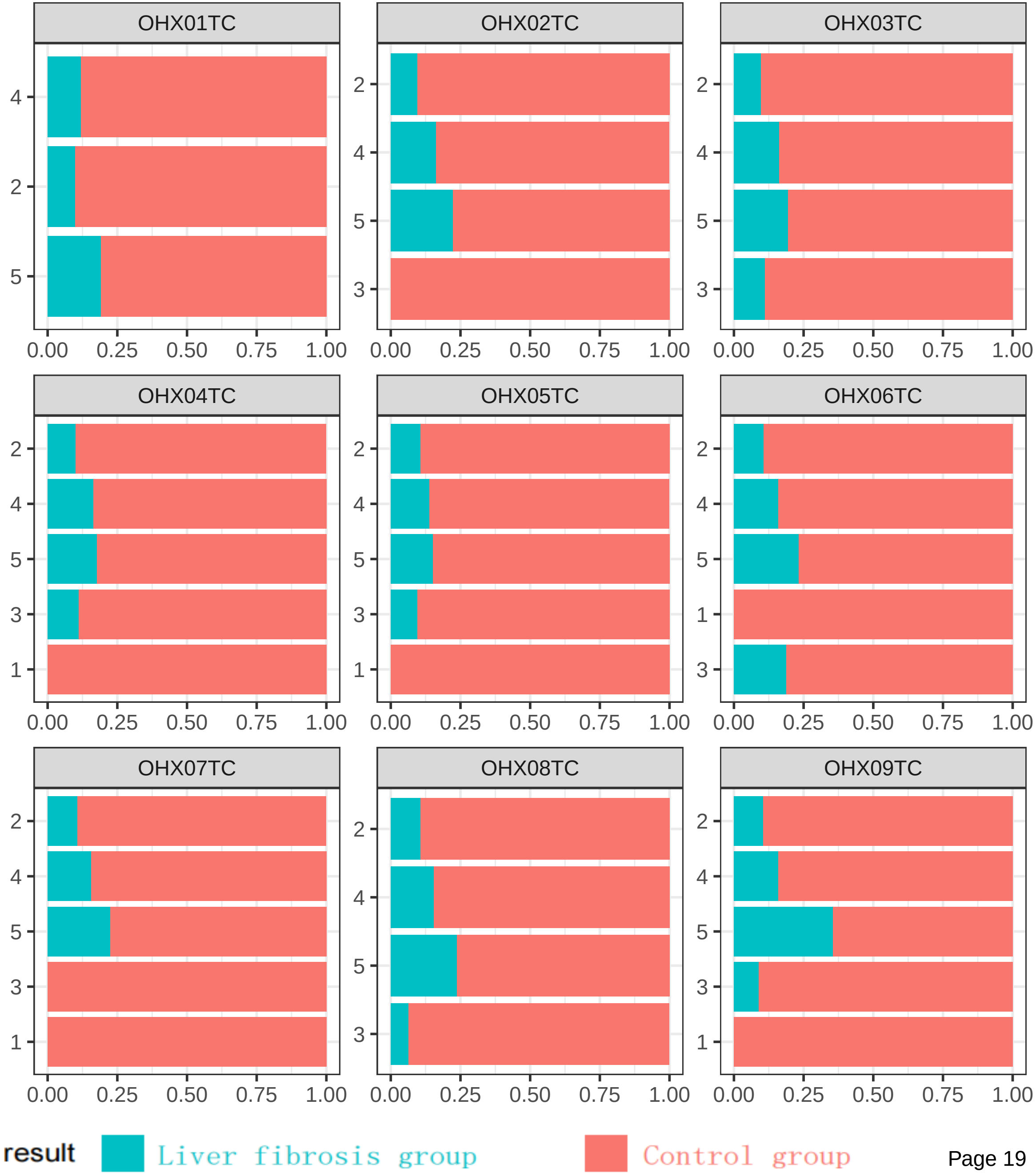

OHX01TC
OHX02TC
OHX03TC
OHX04TC
OHX05TC
OHX06TC
OHX07TC
OHX08TC
OHX09TC
0.00
0.25
0.50
0.75
1.00
result
Liver fibrosis group
Control group

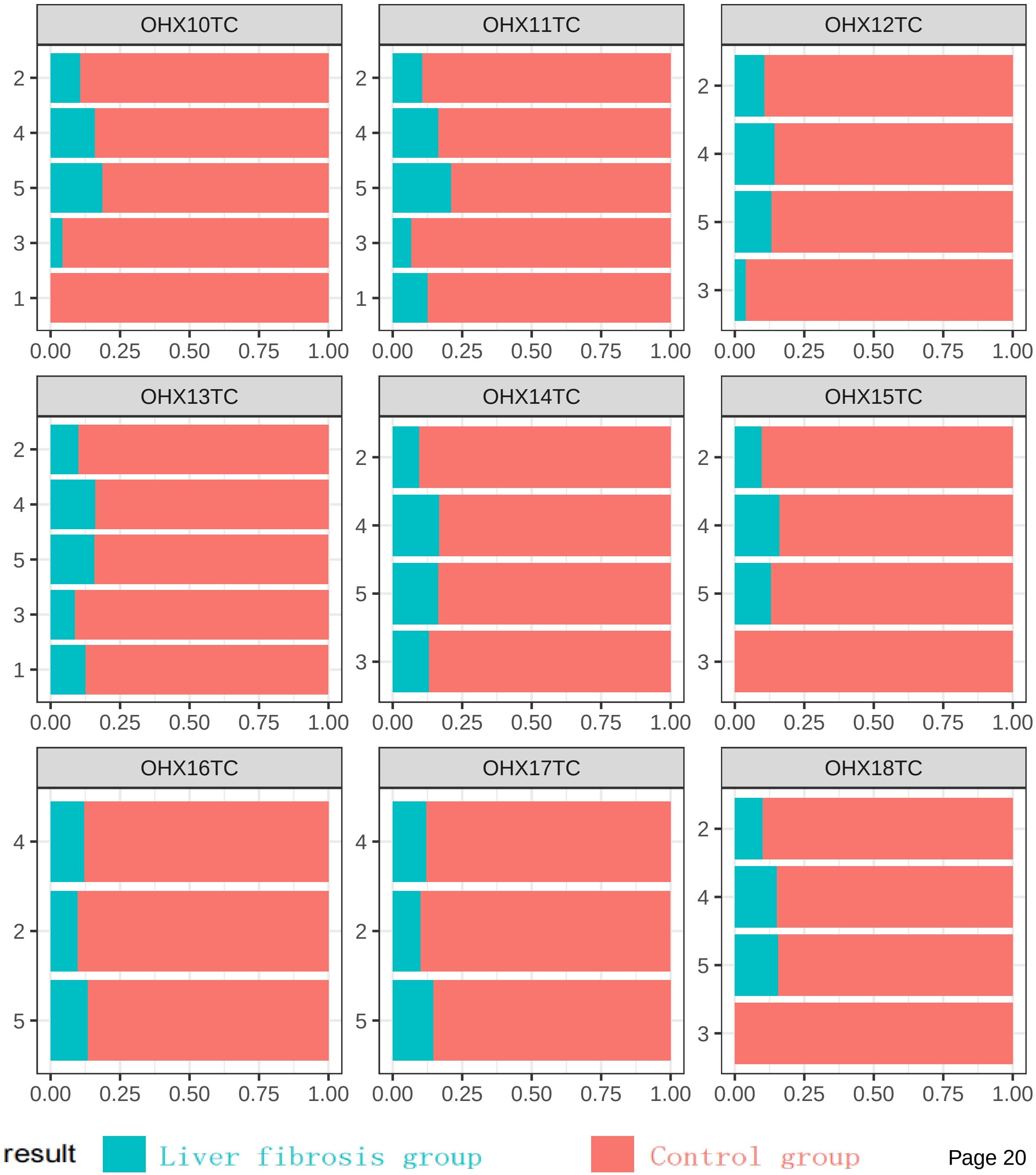

OHX10TC
OHX11TC
OHX12TC
OHX13TC
OHX14TC
OHX15TC
OHX16TC
OHX17TC
OHX18TC
0.00 0.25 0.50 0.75 1.00
result
Liver fibrosis group
Control group

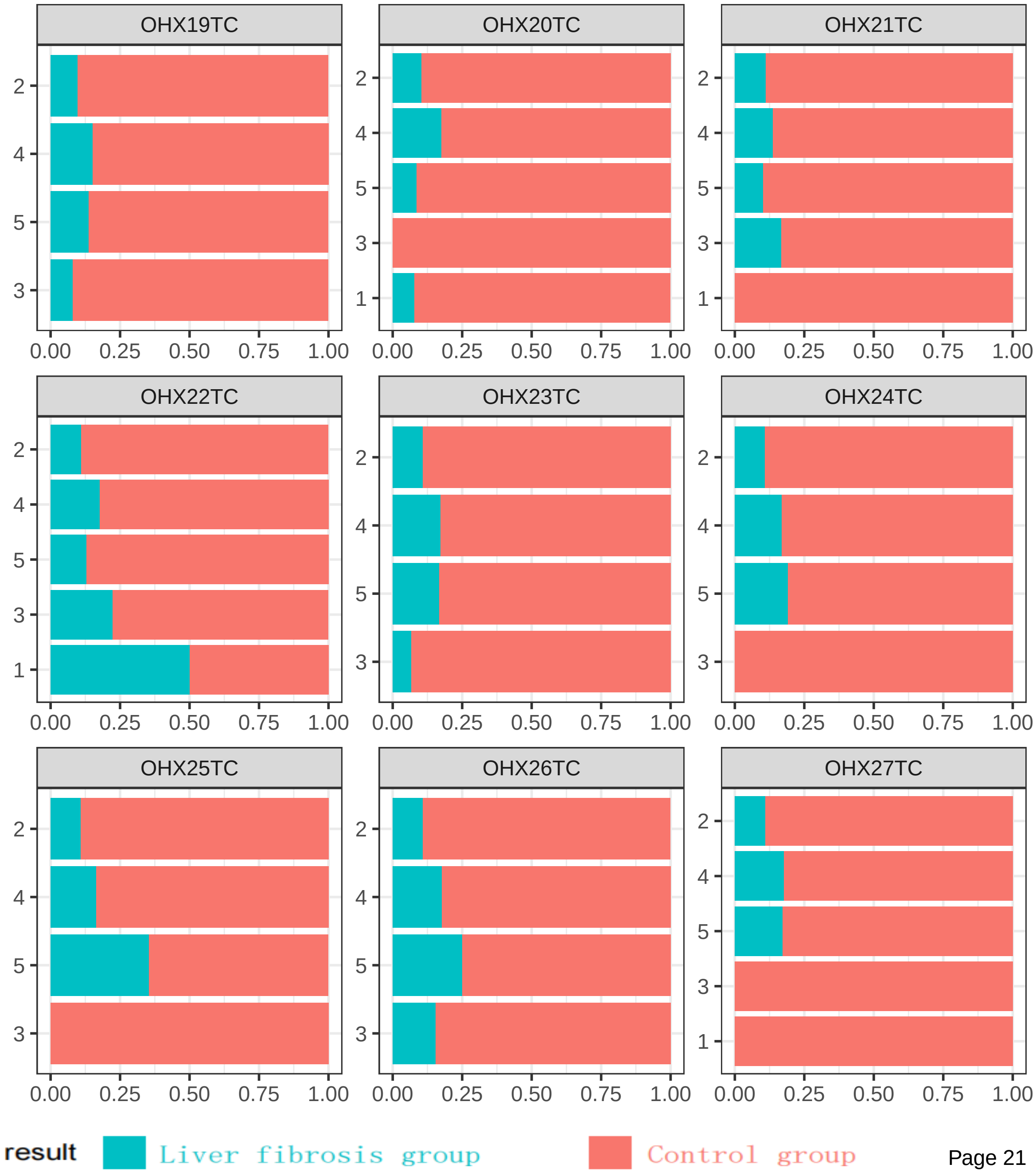
OHX19TC
OHX20TC
OHX21TC
OHX22TC
OHX23TC
OHX24TC
OHX25TC
OHX26TC
OHX27TC
0.00 0.25 0.50 0.75 1.00
result
Liver fibrosis group
Control group

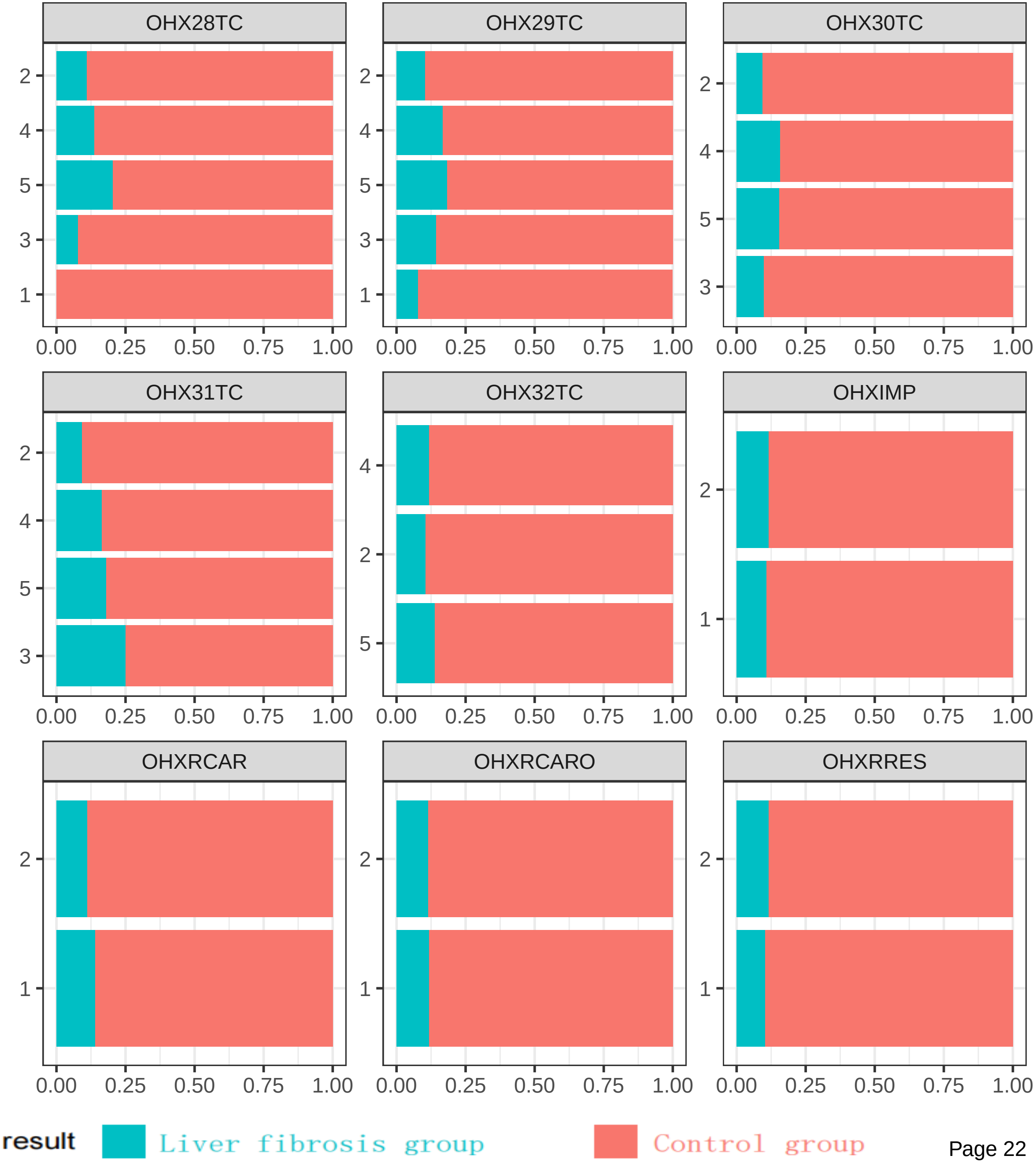
OHX28TC
OHX29TC
OHX30TC
OHX31TC
OHX32TC
OHXIMP
OHXRCAR
OHXRCARO
OHXRRES
0.00
0.25
0.50
0.75
1.00
result
Liver fibrosis group
Control group

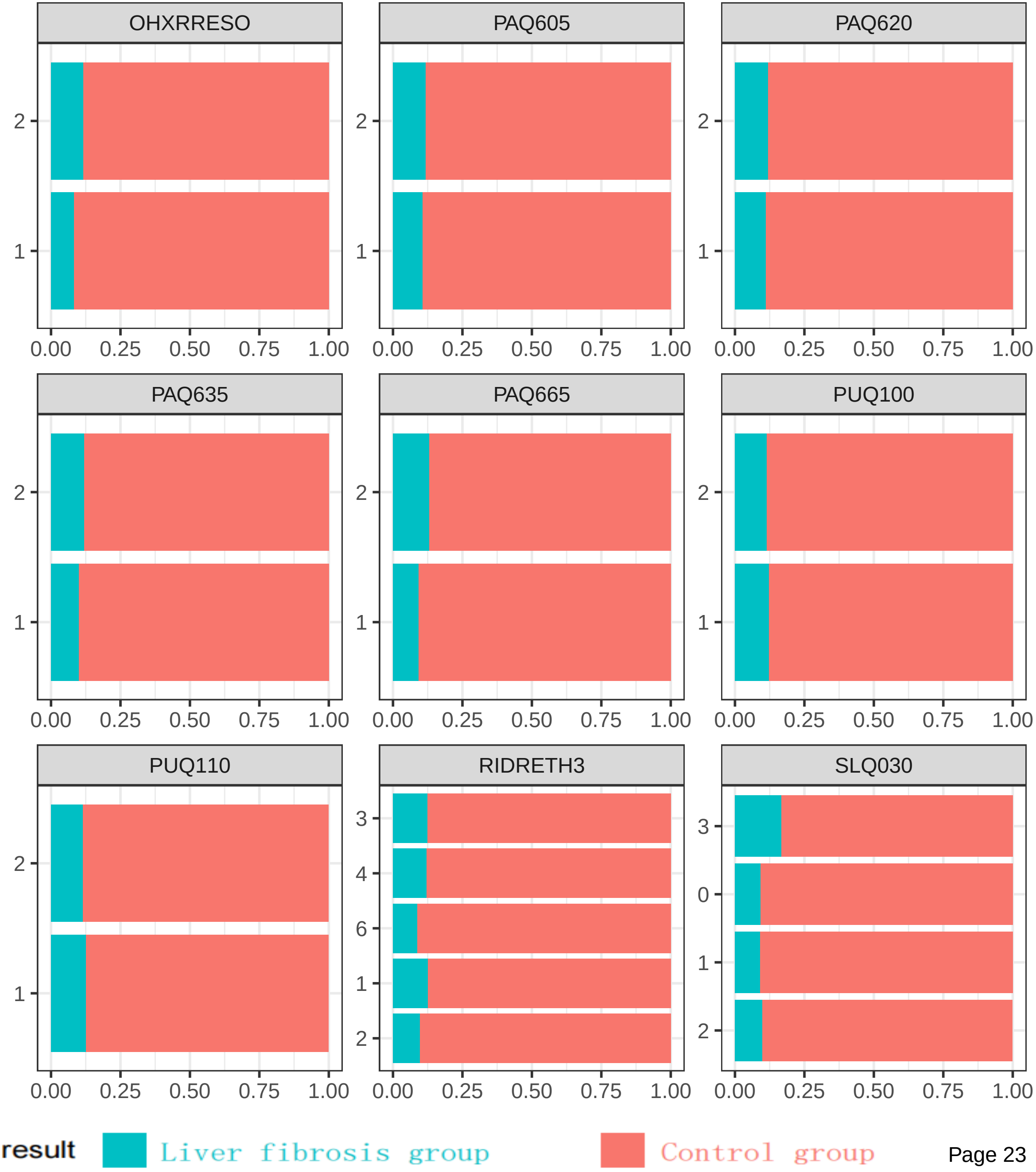
OHXRRESO
PAQ605
PAQ620
PAQ635
PAQ665
PUQ100
PUQ110
RIDRETH3
SLQ030
0.00
0.25
0.50
0.75
1.00
result
Liver fibrosis group
Control group

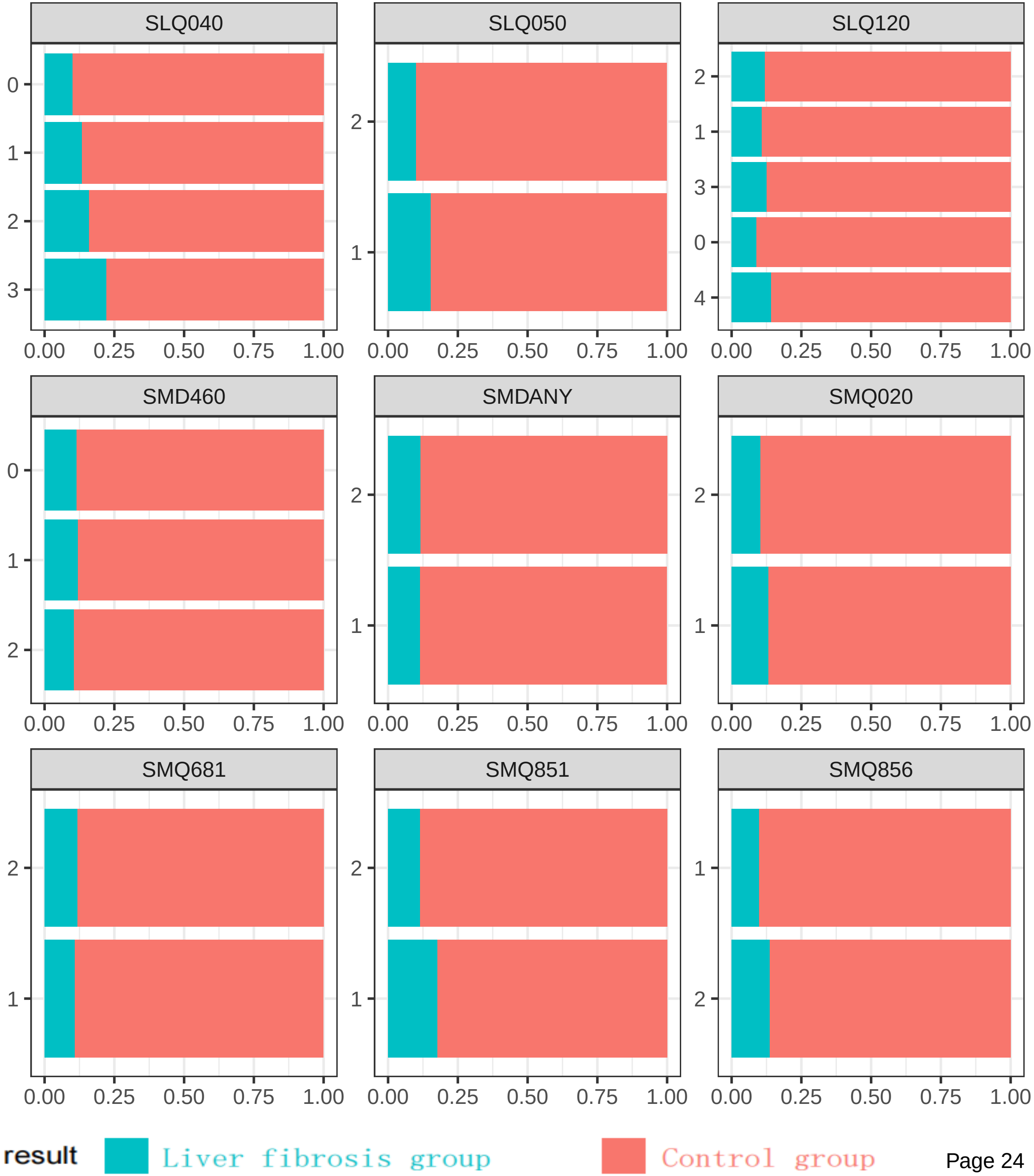
SLQ040
SLQ050
SLQ120
SMD460
SMDANY
SMQ020
SMQ681
SMQ851
SMQ856
0.00
0.25
0.50
0.75
1.00
result
Liver fibrosis group
Control group

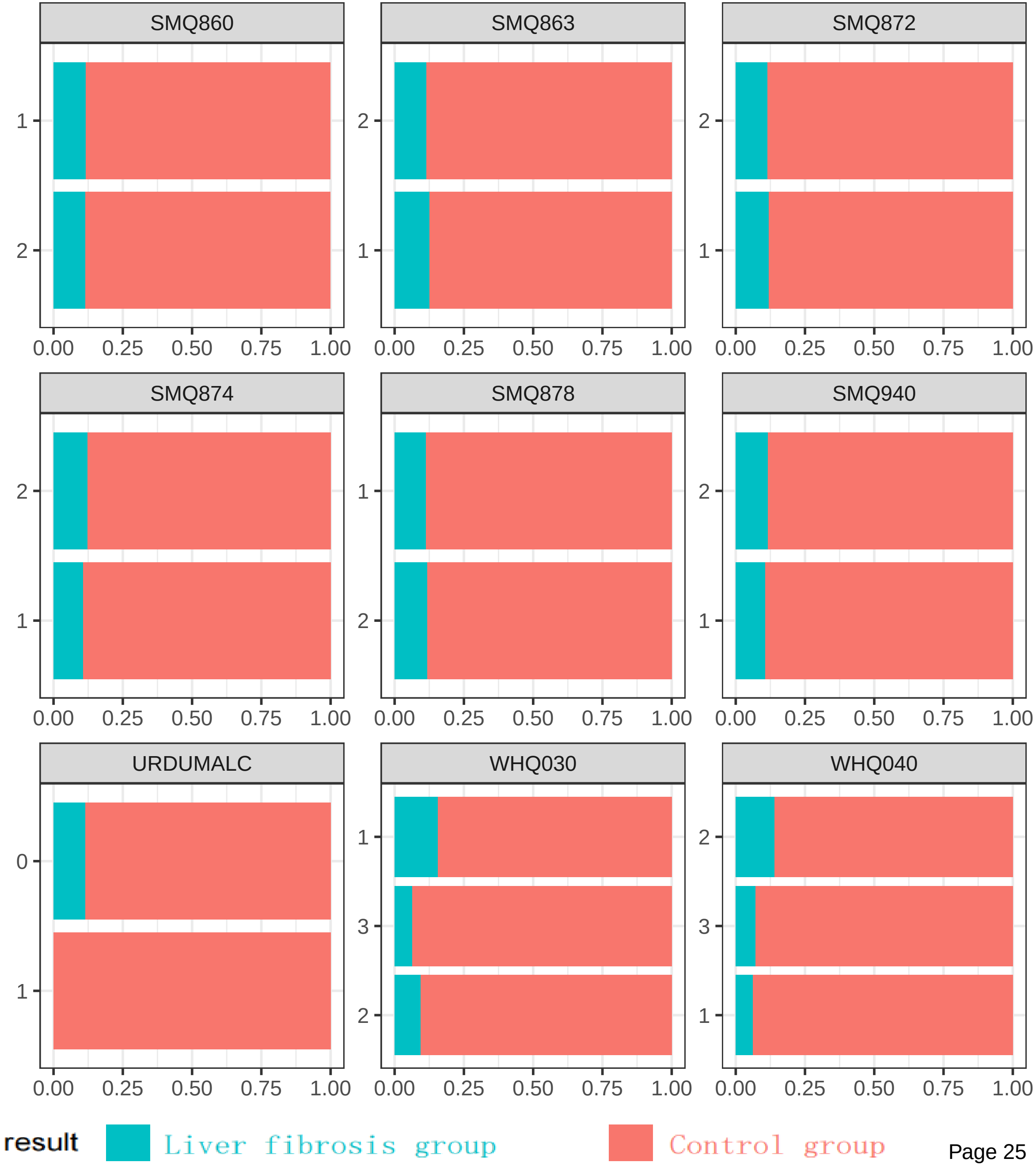

SMQ860
SMQ863
SMQ872
SMQ874
SMQ878
SMQ940
URDUMALC
WHQ030
WHQ040
0.00
0.25
0.50
0.75
1.00
result
Liver fibrosis group
Control group

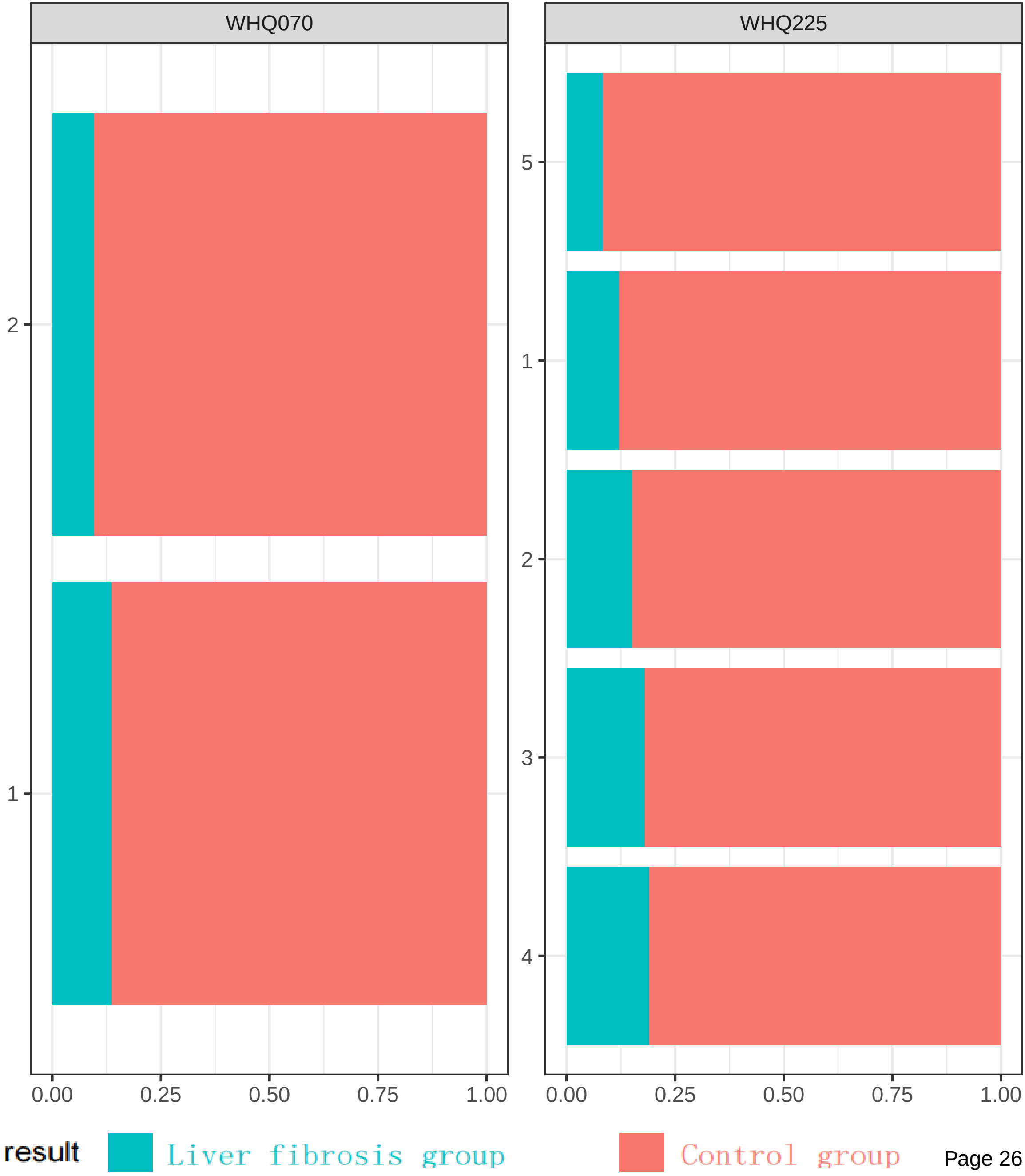
WHQ070
WHQ225
2
1
5
1
2
3
4
0.00
0.25
0.50
0.75
1.00
result
Liver fibrosis group
Control group
