# Supplementary material for: Integrative and interpretable machine learning framework for early non-invasive detection of clinically significant liver fibrosis
Source: Front Med (Lausanne). 2026 Jun 23;13:1736295. doi: 10.3389/fmed.2026.1736295 (PMC13337473; doi:10.3389/fmed.2026.1736295)
Supplement: Supplementary file 4 — The figure shows the frequency distribution of different categories for each categorical variable included in this study, and intuitively presents the number of samples corresponding to different categories of each categorical variable. [file Data_Sheet_4.pdf]

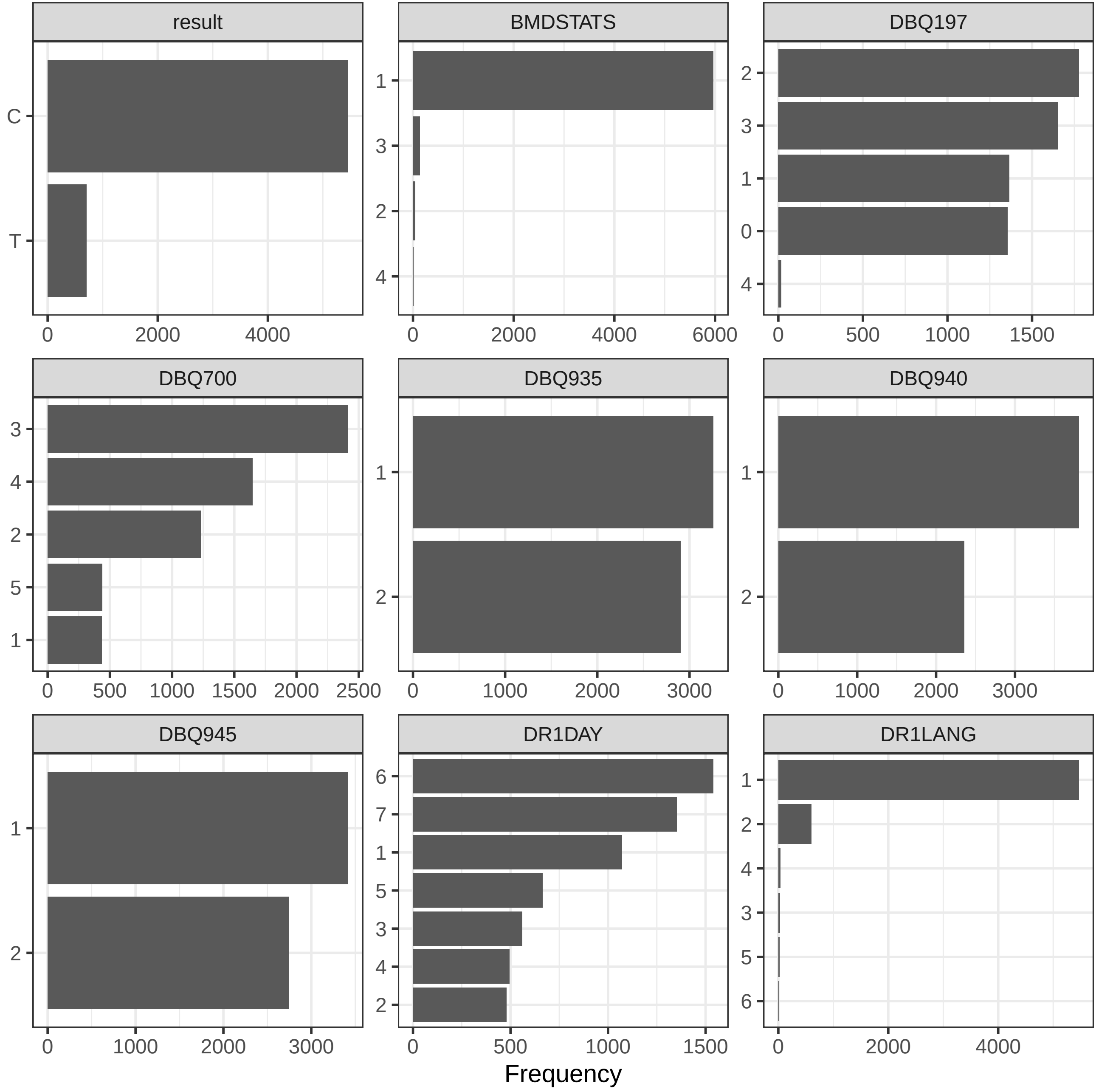
result
C
T
0
2000
4000
BMDSTATS
1
3
2
4
0
2000
4000
6000
DBQ197
2
3
1
0
4
0
500
1000
1500
DBQ700
3
4
2
5
1
0
500
1000
1500
2000
2500
DBQ935
1
2
0
1000
2000
3000
DBQ940
1
2
0
1000
2000
3000
DBQ945
1
2
0
1000
2000
3000
DR1DAY
6
7
1
5
3
4
2
0
500
1000
1500
DR1LANG
1
2
4
3
5
6
0
2000
4000
Frequency

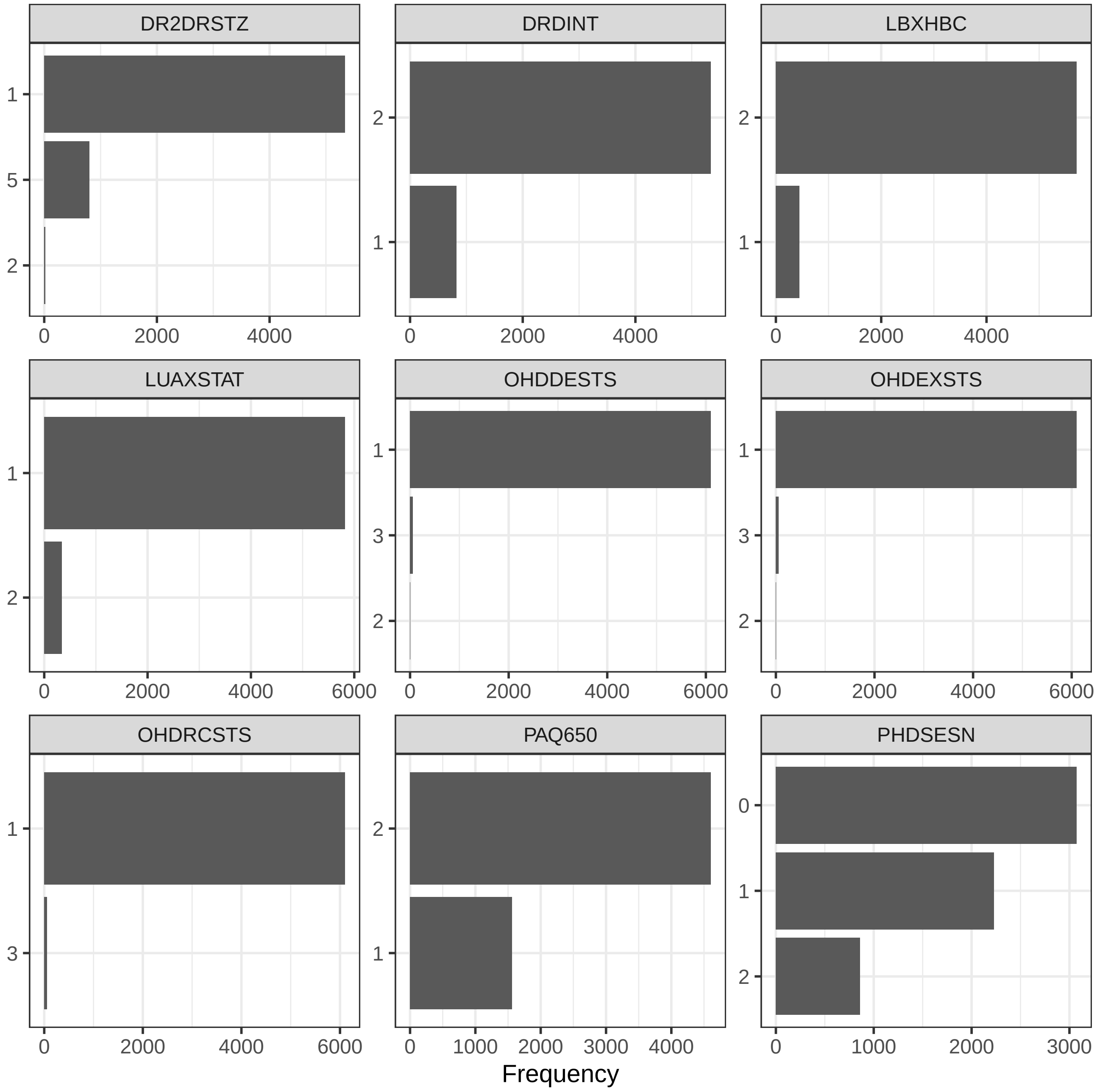
DR2DRSTZ
1
5
2
0
2000
4000
DRDINT
2
1
0
2000
4000
LBXHBC
2
1
0
2000
4000
LUAXSTAT
1
2
0
2000
4000
6000
OHDDESTS
1
3
2
0
2000
4000
6000
OHDEXSTS
1
3
2
0
2000
4000
6000
OHDRCSTS
1
3
0
2000
4000
6000
PAQ650
2
1
0
1000
2000
3000
4000
PHDSESN
0
1
2
0
1000
2000
3000
Frequency

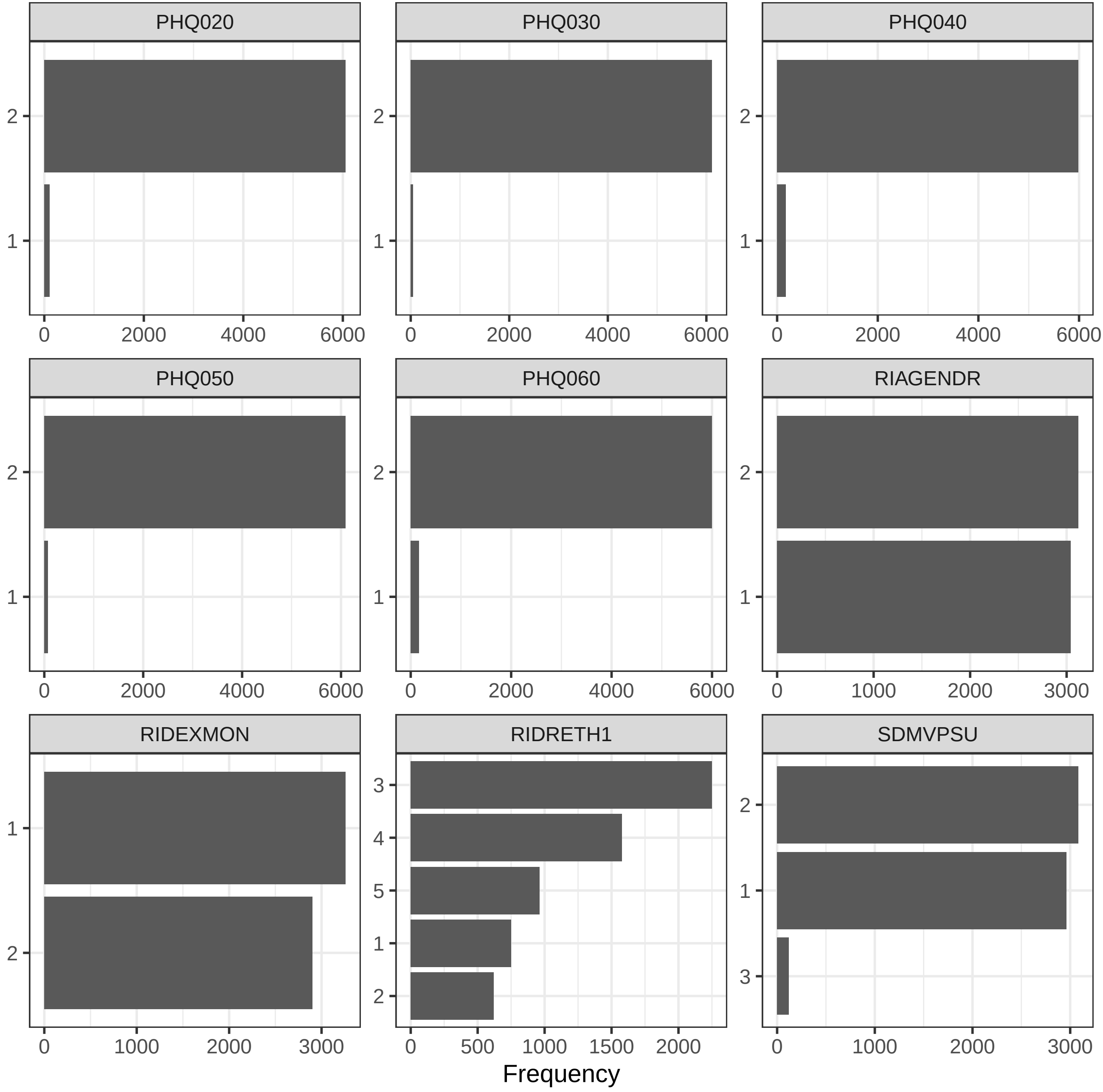
PHQ020
PHQ030
PHQ040
PHQ050
PHQ060
RIAGENDR
RIDEXMON
RIDRETH1
SDMVPSU
Frequency

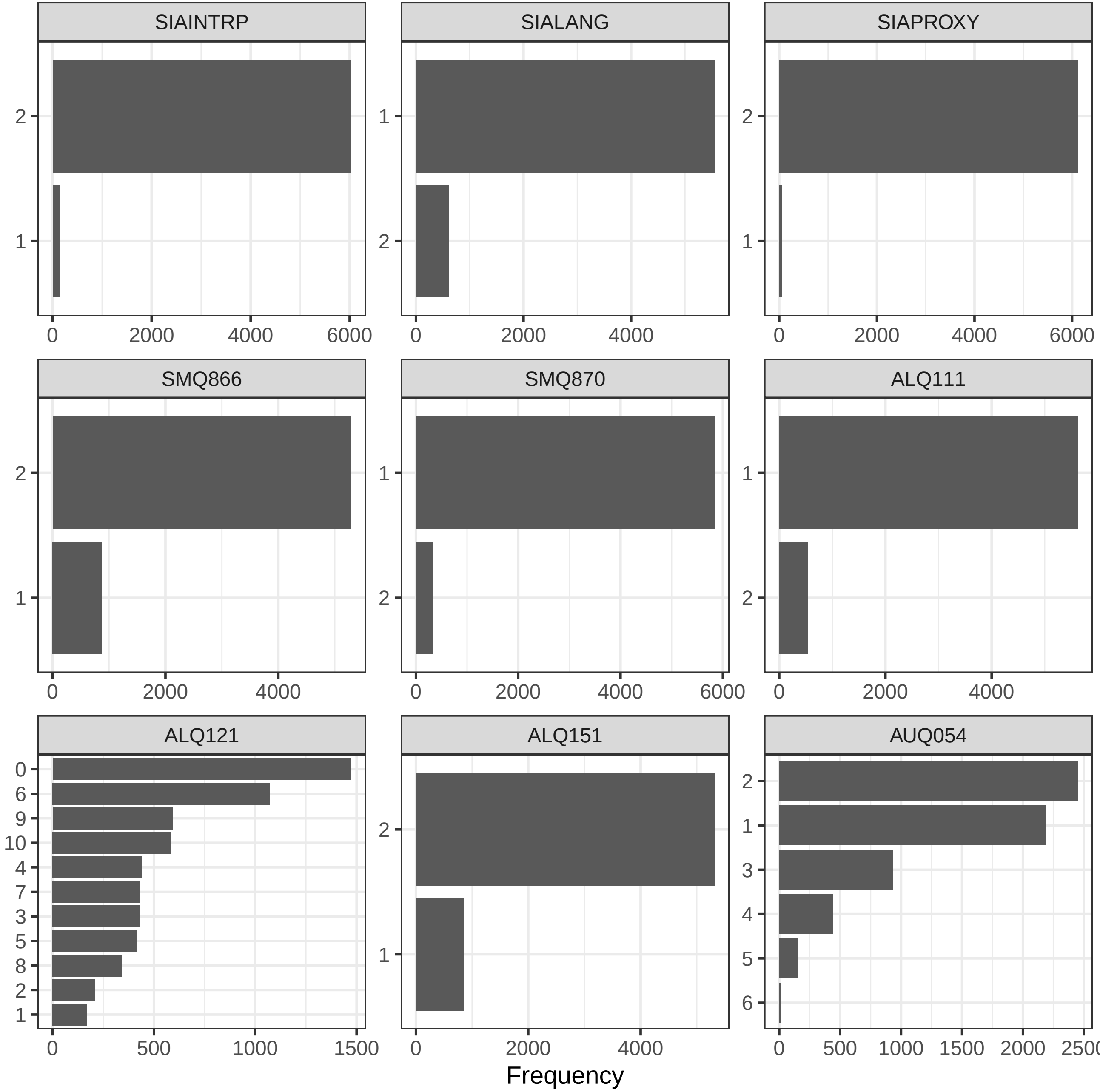
SIAINTRP
SIALANG
SIAPROXY
SMQ866
SMQ870
ALQ111
ALQ121
ALQ151
AUQ054
Frequency

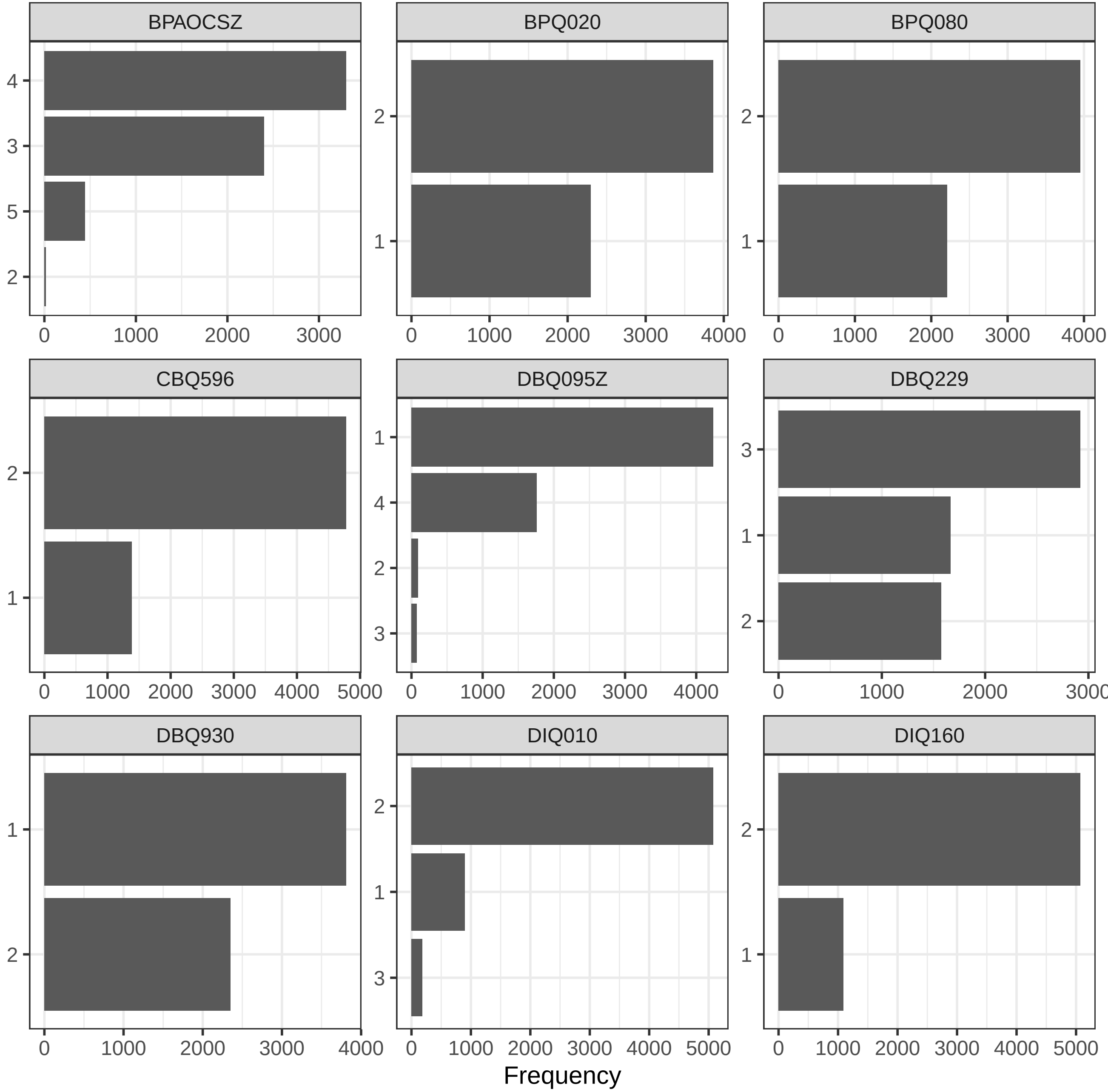
BPAOCSZ
4
3
5
2
0
1000
2000
3000
BPQ020
2
1
0
1000
2000
3000
4000
BPQ080
2
1
0
1000
2000
3000
4000
CBQ596
2
1
0
1000
2000
3000
4000
5000
DBQ095Z
1
4
2
3
0
1000
2000
3000
4000
DBQ229
3
1
2
0
1000
2000
3000
DBQ930
1
2
0
1000
2000
3000
4000
DIQ010
2
1
3
0
1000
2000
3000
4000
5000
DIQ160
2
1
0
1000
2000
3000
4000
5000
Frequency

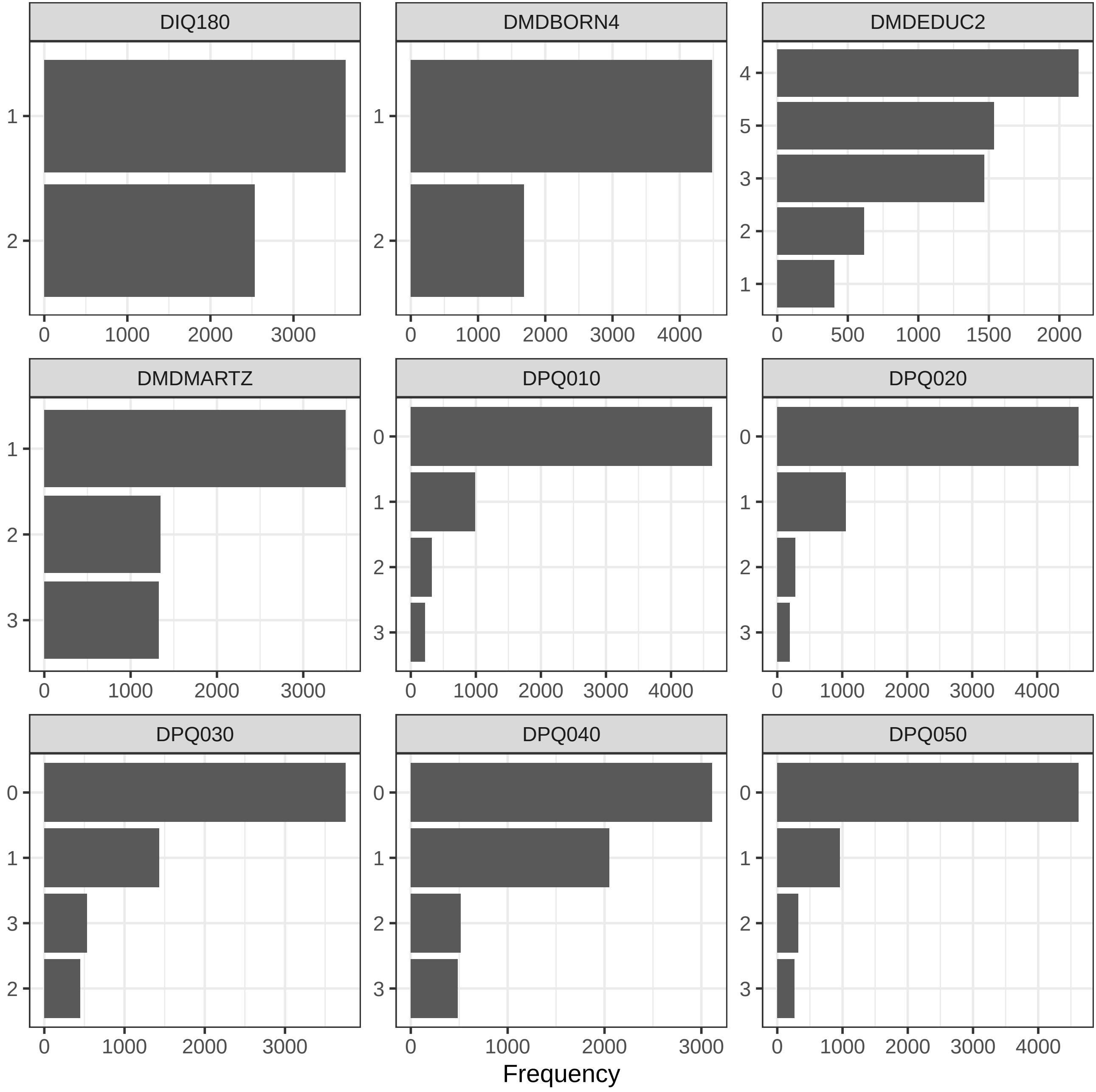
DIQ180
DMDBORN4
DMDEDUC2
DMDMARTZ
DPQ010
DPQ020
DPQ030
DPQ040
DPQ050
Frequency

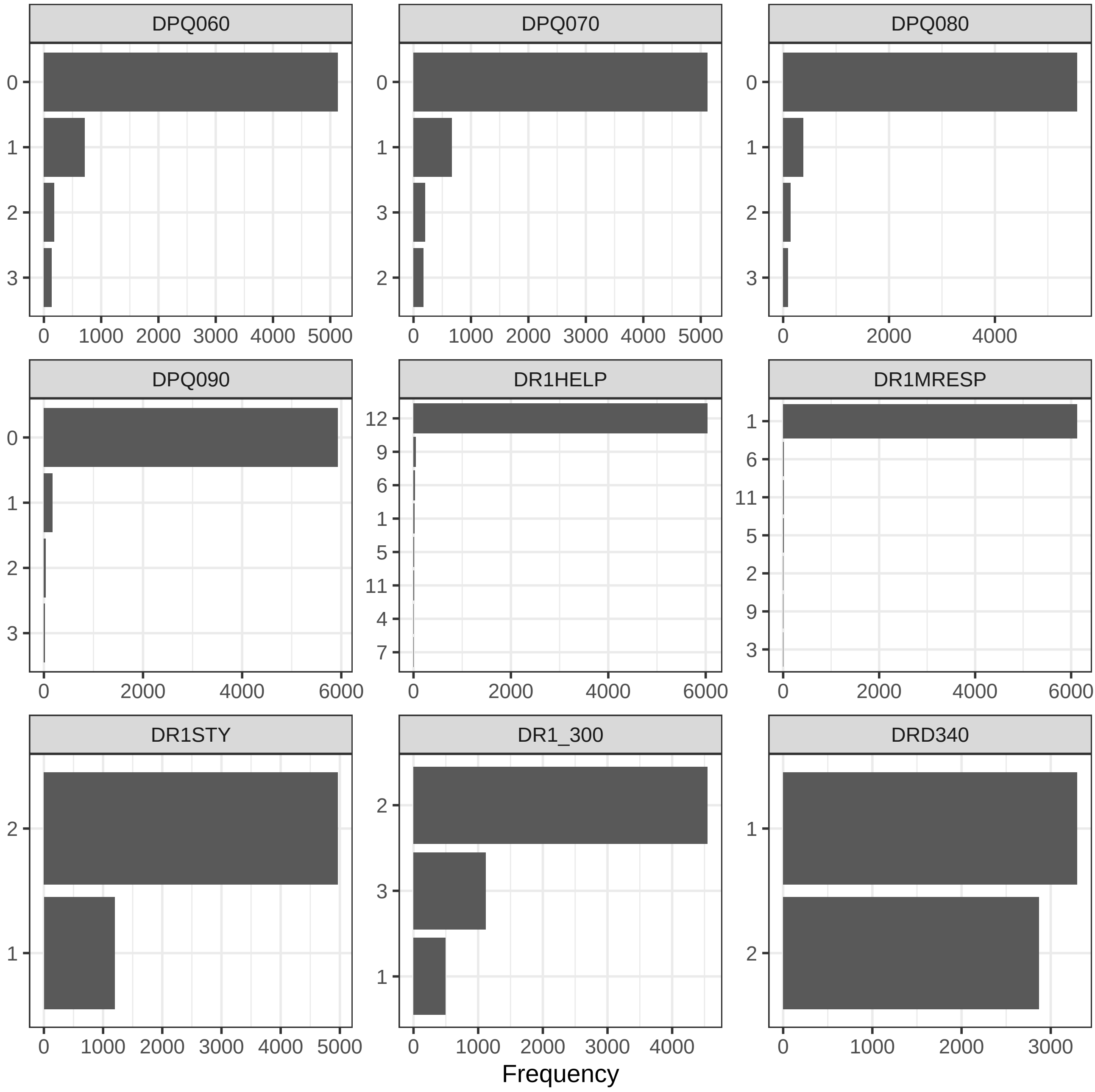
DPQ060
DPQ070
DPQ080
DPQ090
DR1HELP
DR1MRESP
DR1STY
DR1_300
DRD340
0
1
2
3
12
9
6
5
11
4
7
1000
2000
3000
4000
5000
6000
Frequency

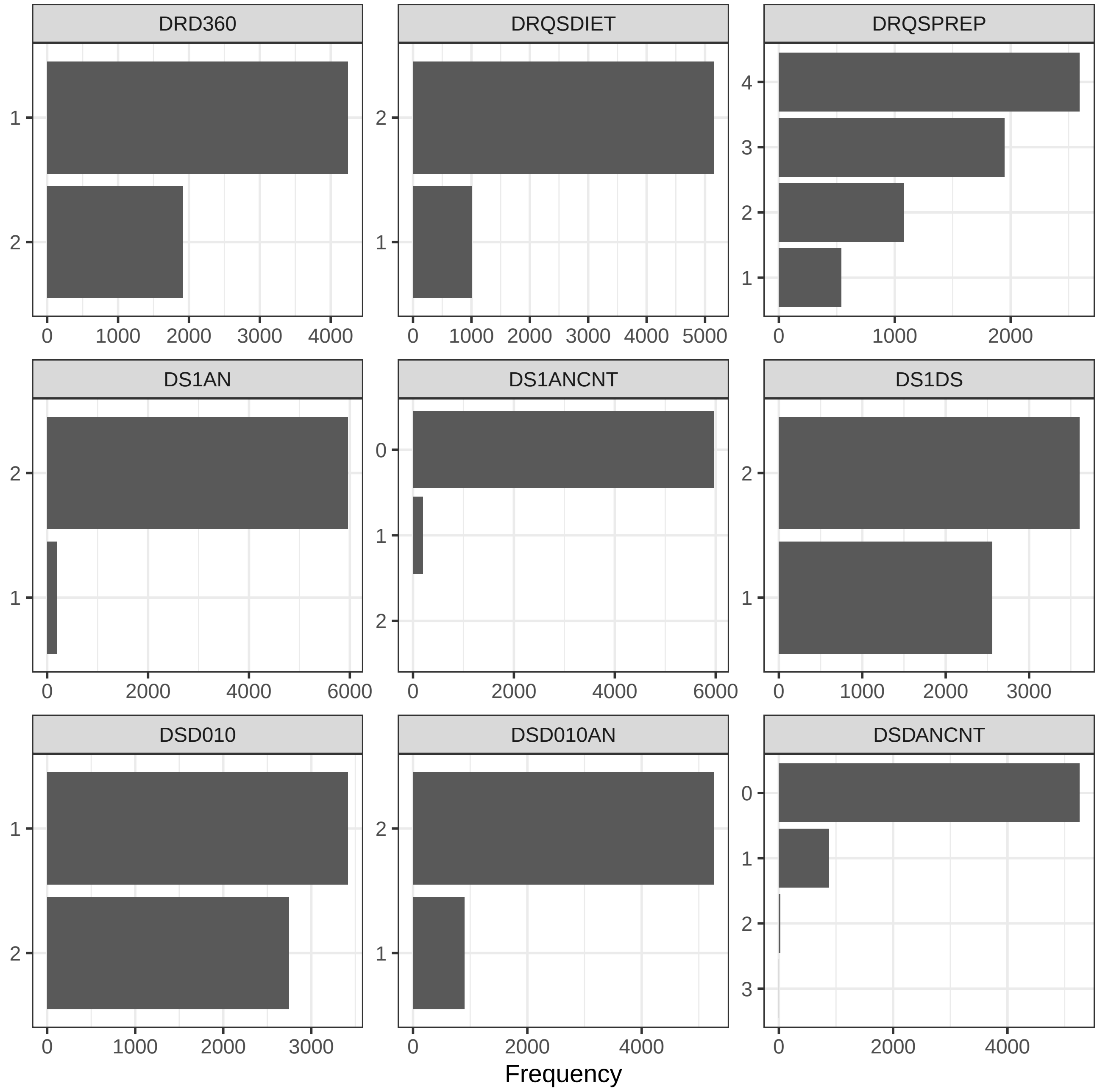
DRD360
DRQSDIET
DRQSPREP
DS1AN
DS1ANCNT
DS1DS
DSD010
DSD010AN
DSDANCNT
Frequency

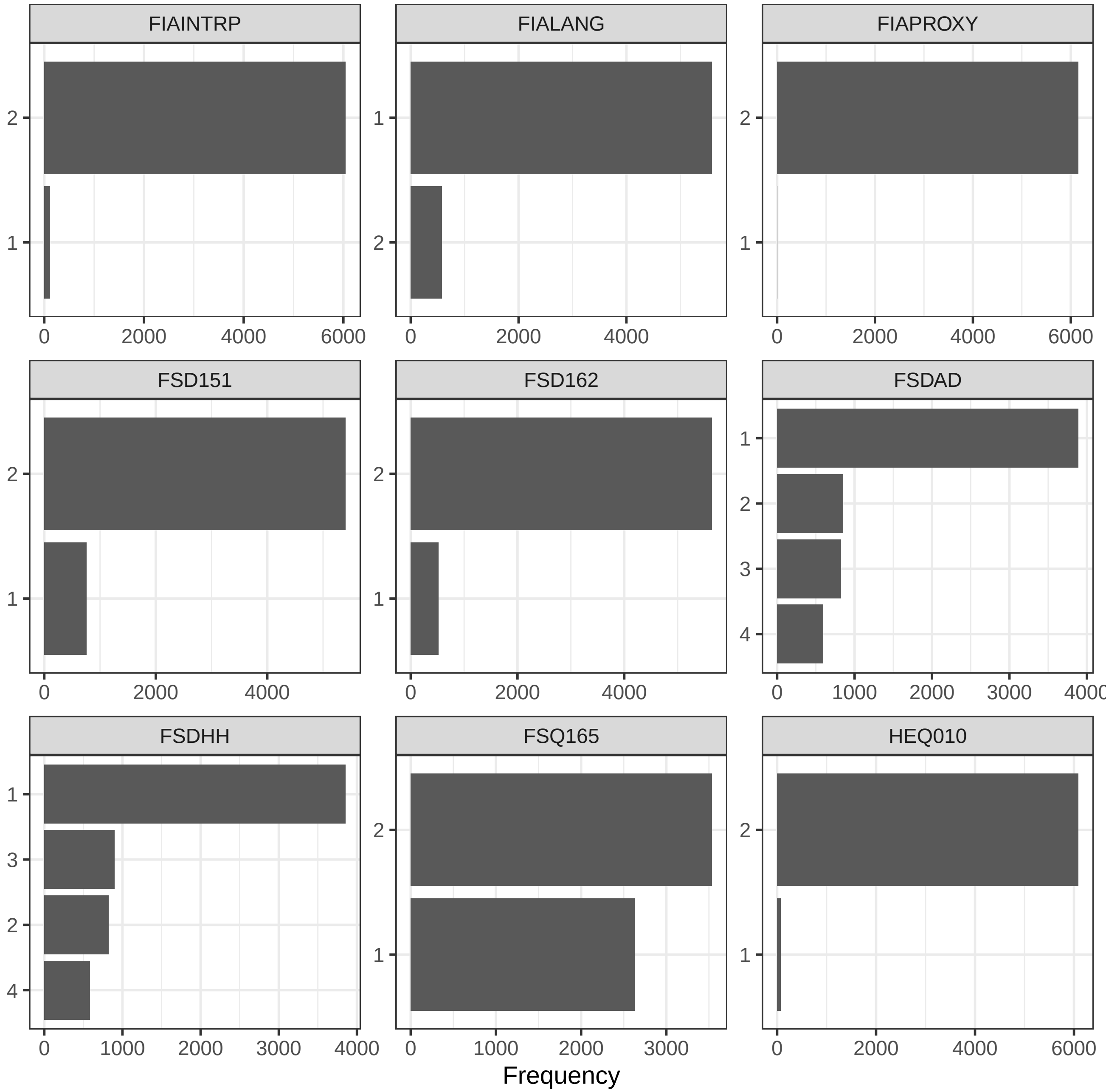
FIAINTRP
FIALANG
FIAPROXY
FSD151
FSD162
FSDAD
FSDHH
FSQ165
HEQ010
Frequency

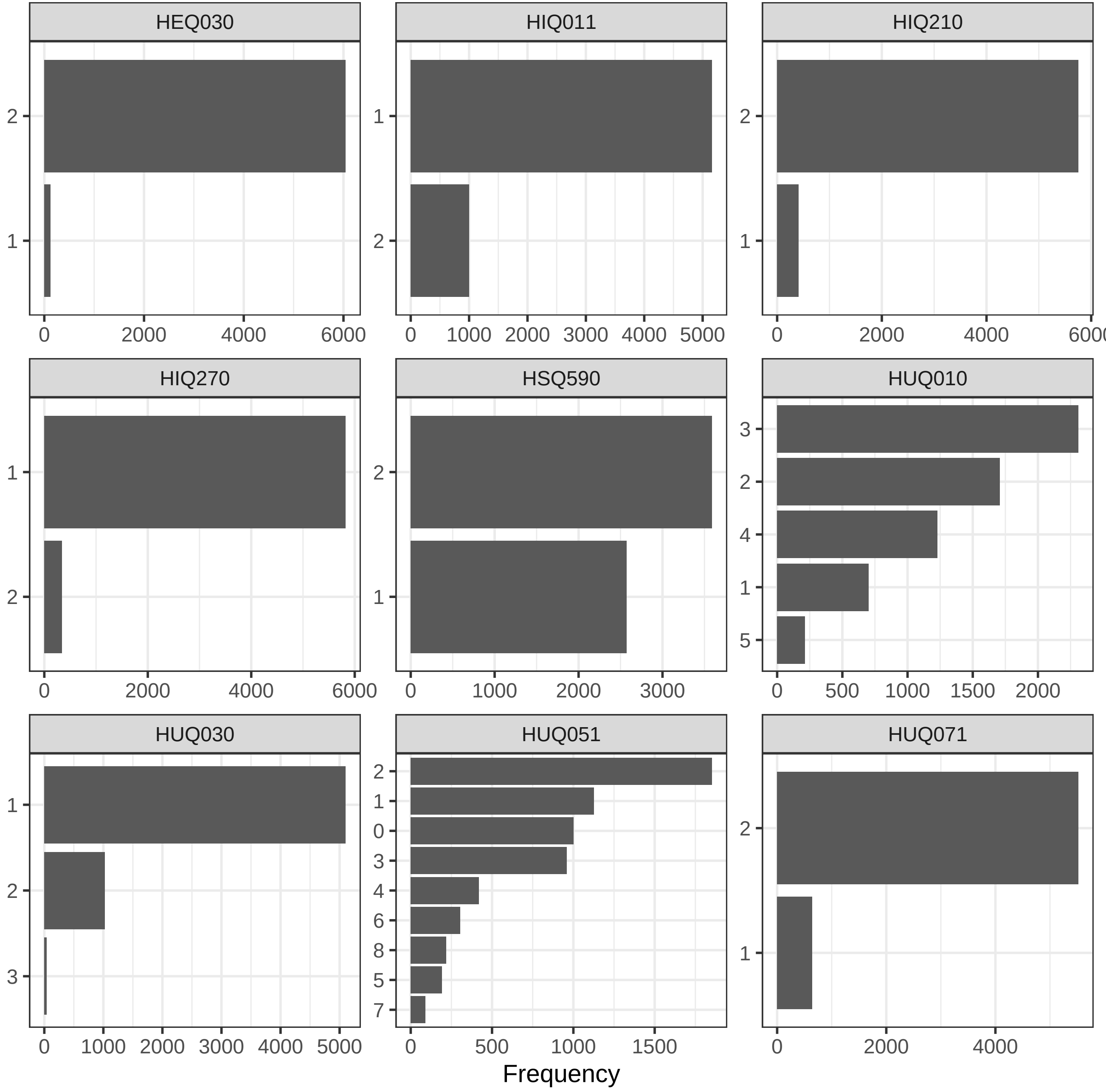
HEQ030
2
1
0
2000
4000
6000
HIQ011
1
2
0
1000
2000
3000
4000
5000
HIQ210
2
1
0
2000
4000
6000
HIQ270
1
2
0
2000
4000
6000
HSQ590
2
1
0
1000
2000
3000
HUQ010
3
2
4
1
5
0
500
1000
1500
2000
HUQ030
1
2
3
0
1000
2000
3000
4000
5000
HUQ051
2
1
0
3
4
6
8
5
7
0
500
1000
1500
Frequency
HUQ071
2
1
0
2000
4000

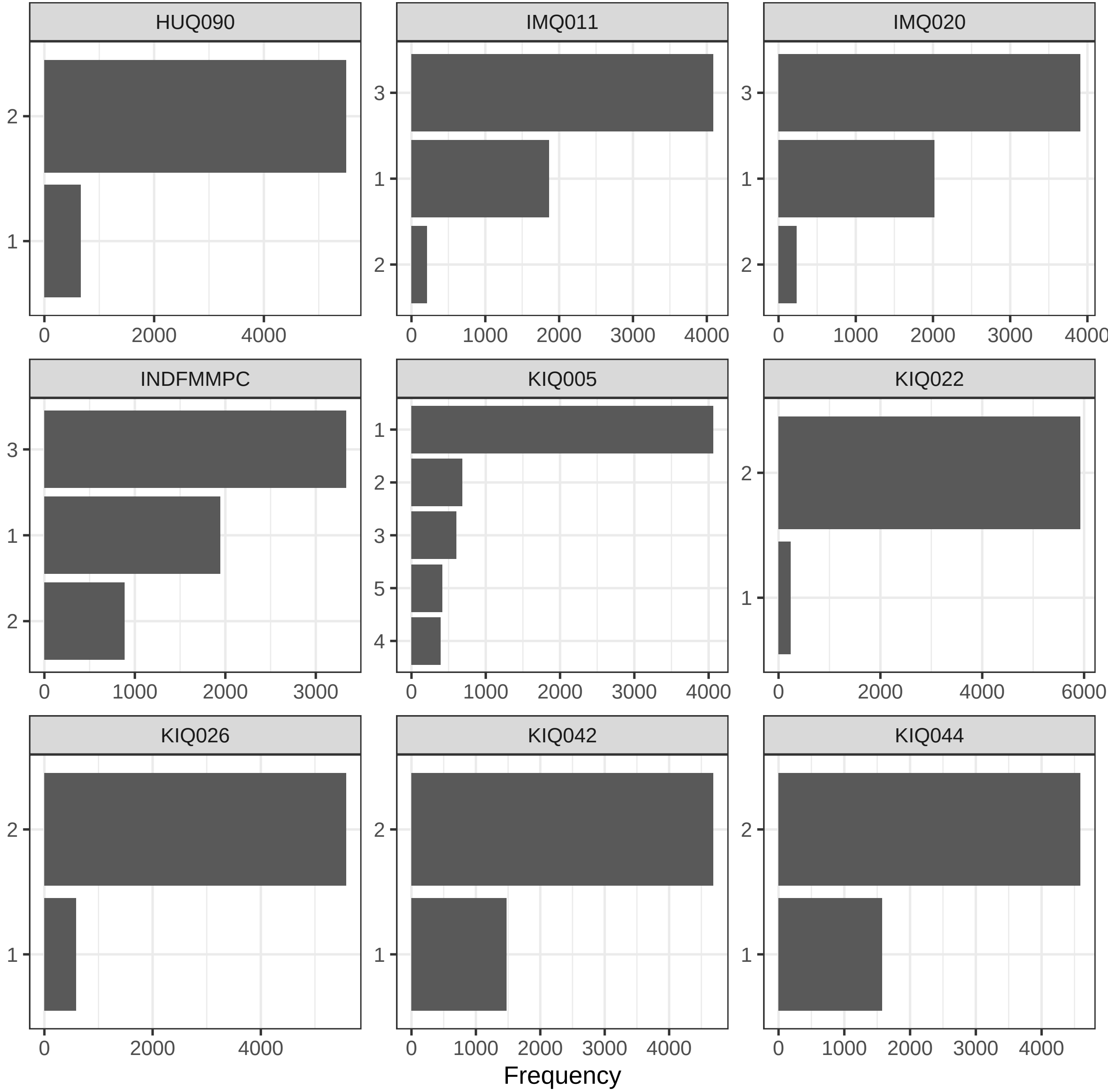

HUQ090
IMQ011
IMQ020
INDFMMPC
KIQ005
KIQ022
KIQ026
KIQ042
KIQ044
Frequency

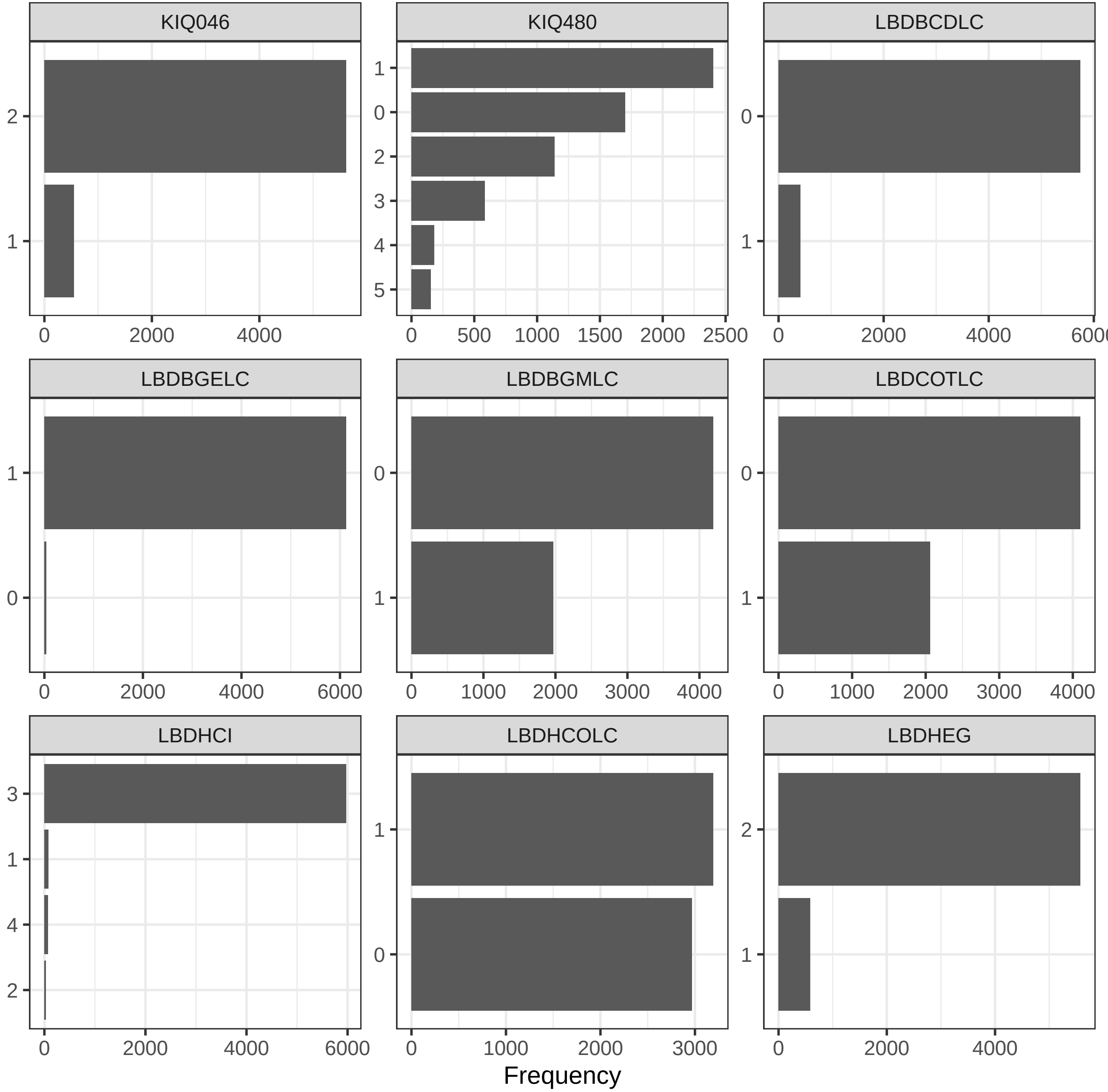
KIQ046
KIQ480
LBDBCDLC
LBDBGELC
LBDBGMLC
LBDCOTLC
LBDHCI
LBDHCOLC
LBDHEG
Frequency

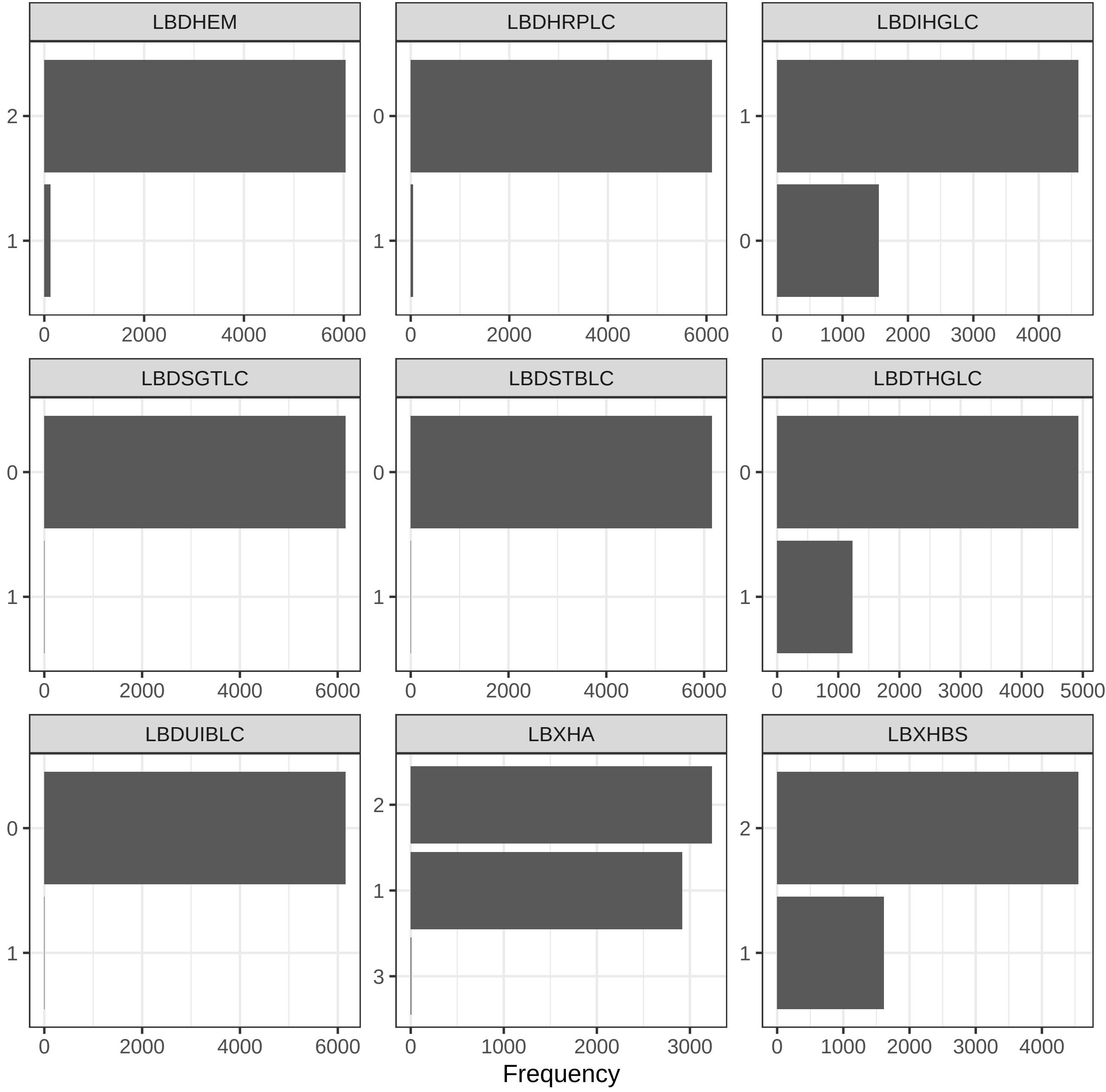

LBDHEM
LBDHRPLC
LBDIHGLC
LBDSGTLC
LBDSTBLC
LBDTHGLC
LBDUIBLC
LBXHA
LBXHBS
Frequency

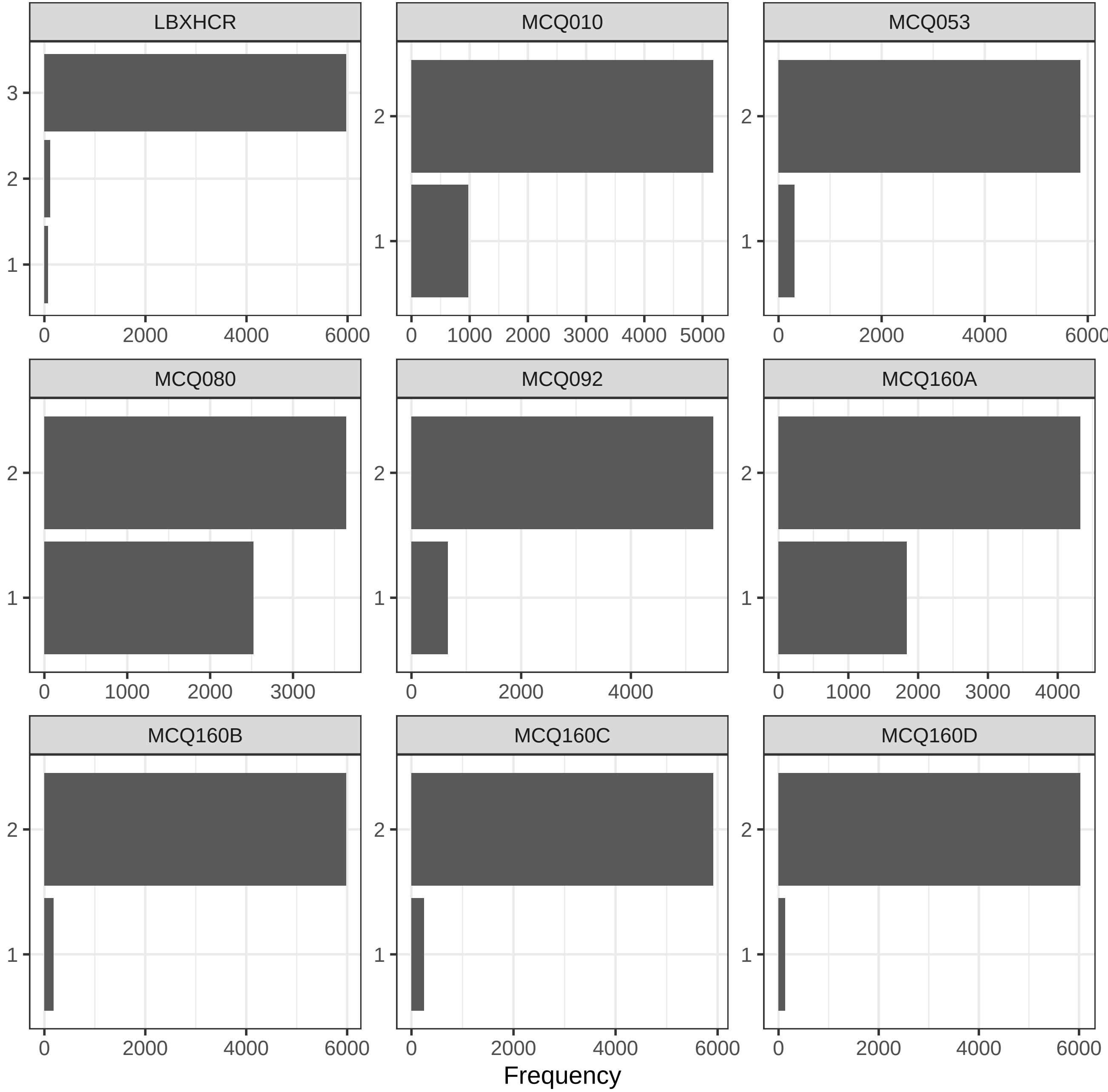
LBXHCR
MCQ010
MCQ053
MCQ080
MCQ092
MCQ160A
MCQ160B
MCQ160C
MCQ160D
Frequency

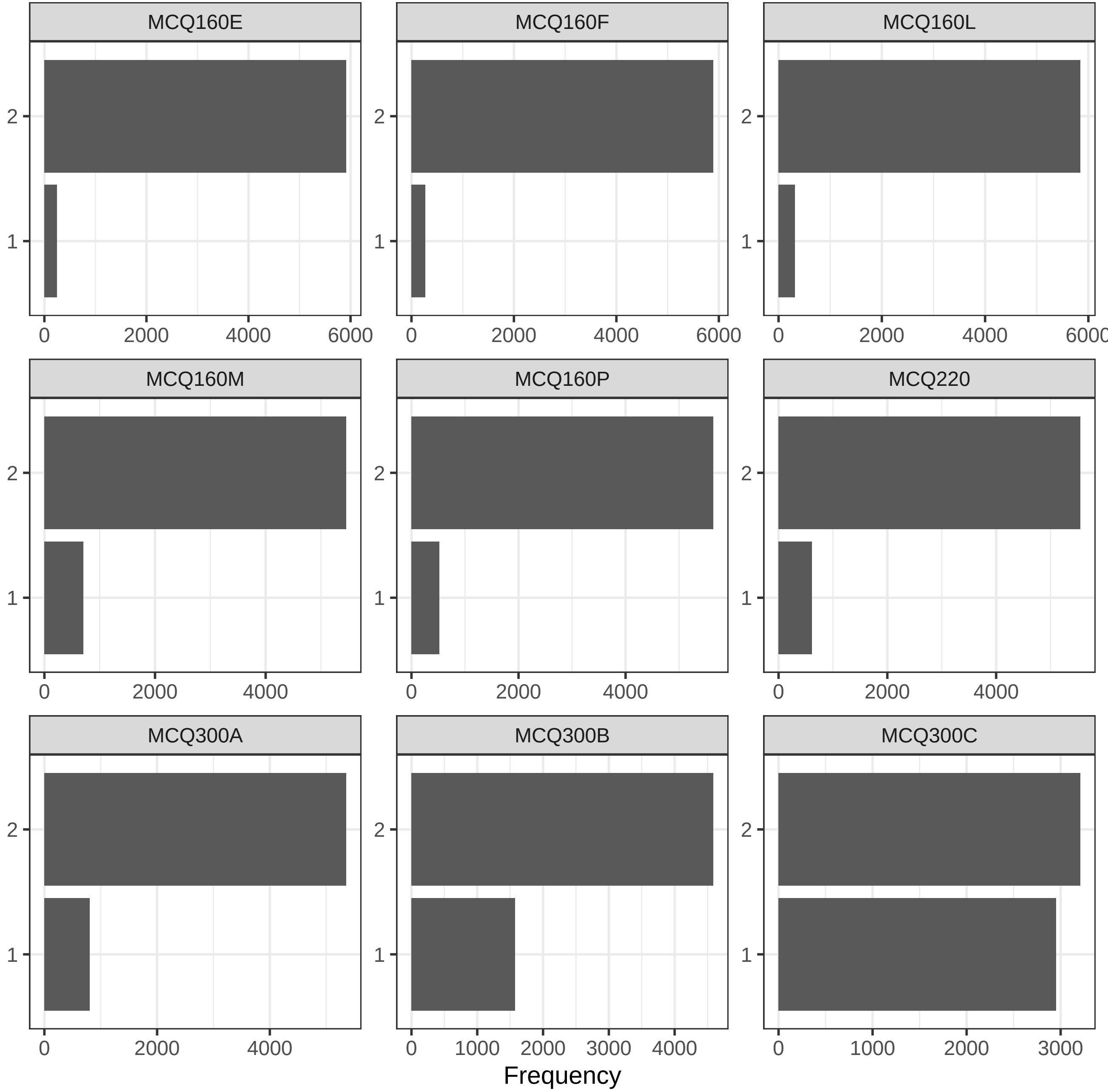
MCQ160E
MCQ160F
MCQ160L
MCQ160M
MCQ160P
MCQ220
MCQ300A
MCQ300B
MCQ300C
2
1
0
1000
2000
3000
4000
6000
Frequency

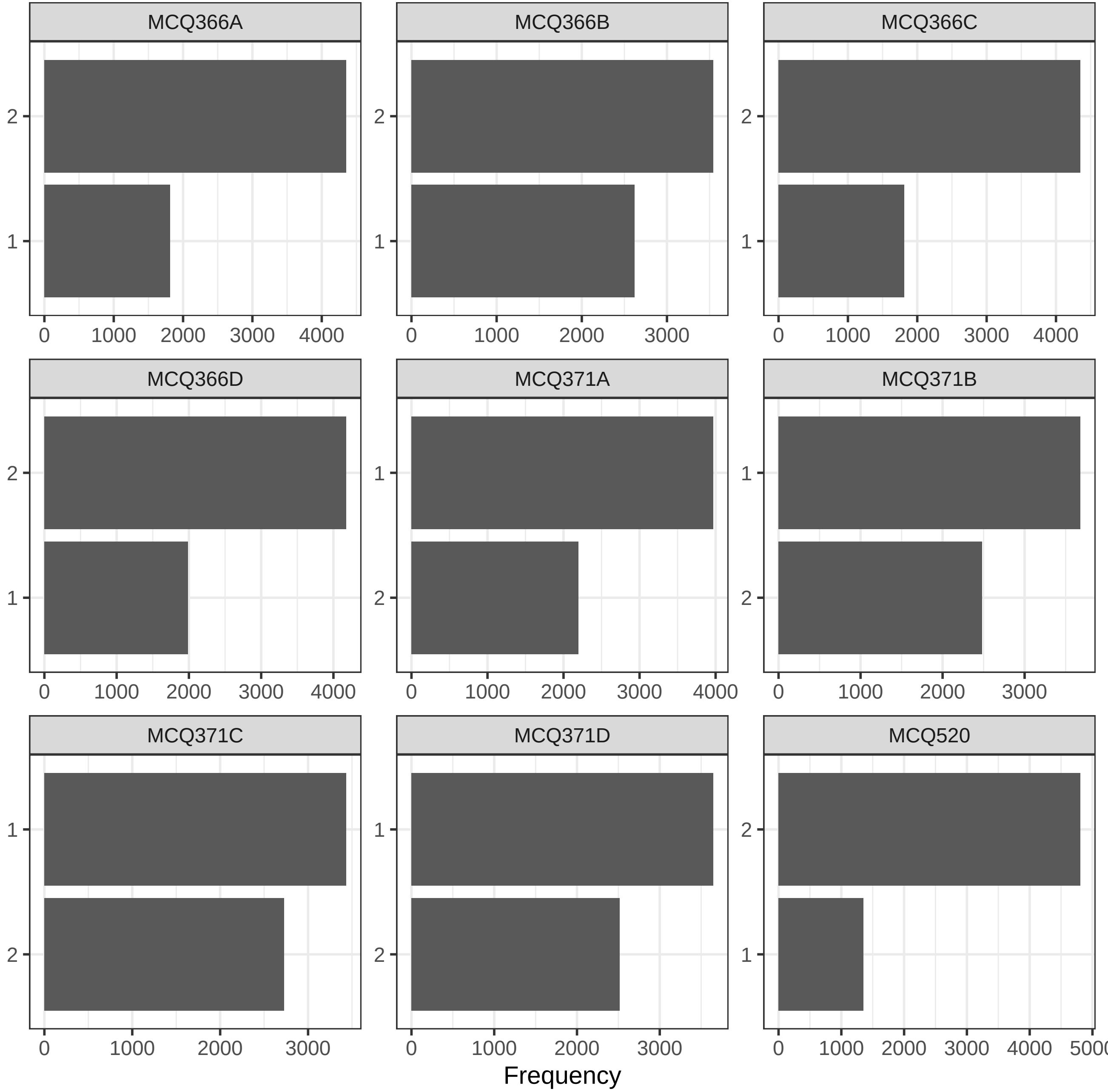
MCQ366A
MCQ366B
MCQ366C
MCQ366D
MCQ371A
MCQ371B
MCQ371C
MCQ371D
MCQ520
Frequency

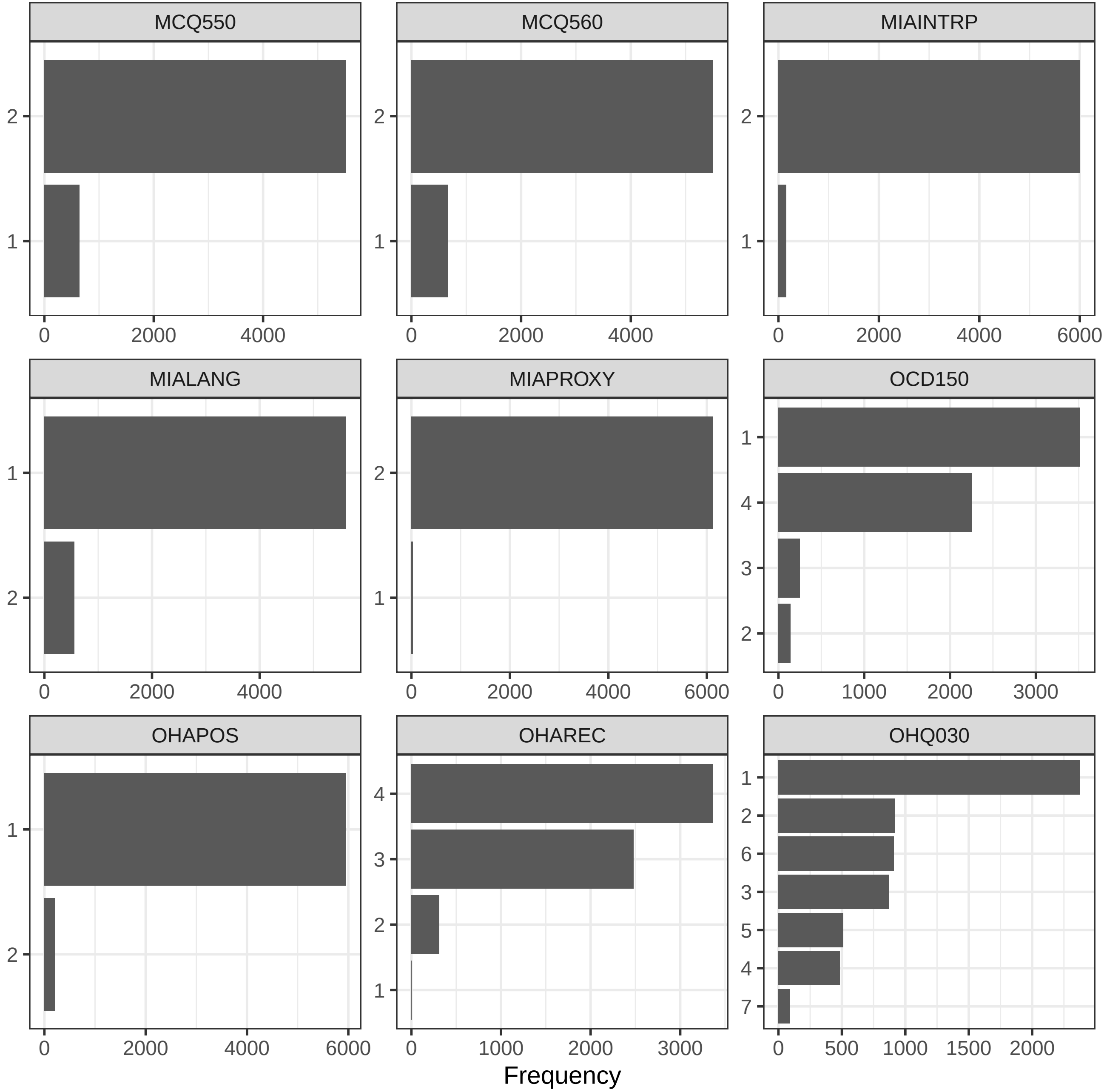
MCQ550
MCQ560
MIAINTRP
MIALANG
MIAPROXY
OCD150
OHAPOS
OHAREC
OHQ030
Frequency

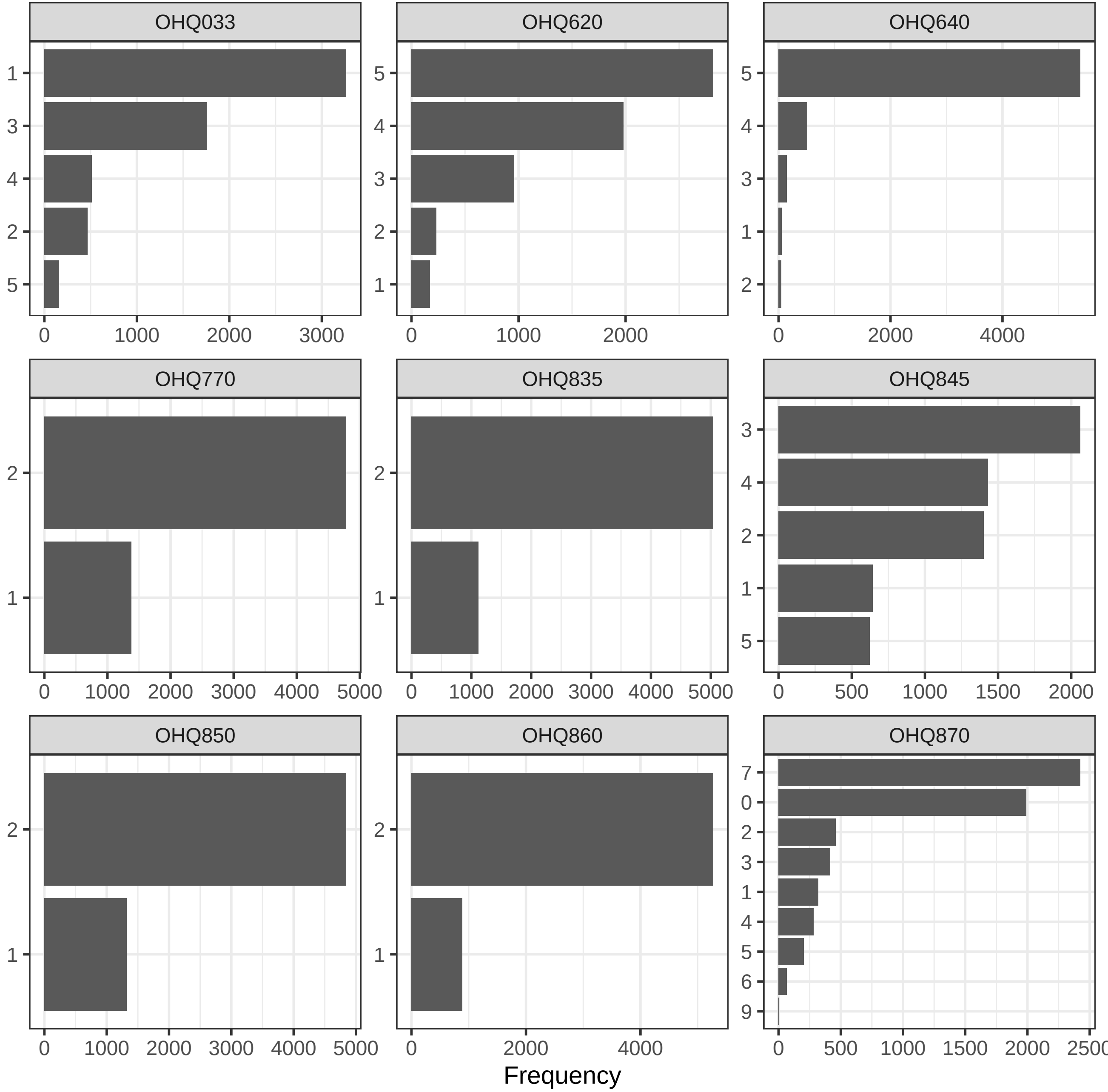
OHQ033
1
3
4
2
5
0
1000
2000
3000
OHQ620
5
4
3
2
1
0
1000
2000
OHQ640
5
4
3
1
2
0
2000
4000
OHQ770
2
1
0
1000
2000
3000
4000
5000
OHQ835
2
1
0
1000
2000
3000
4000
5000
OHQ845
3
4
2
1
5
0
500
1000
1500
2000
OHQ850
2
1
0
1000
2000
3000
4000
5000
OHQ860
2
1
0
2000
4000
OHQ870
7
0
2
3
1
4
5
6
9
0
500
1000
1500
2000
2500
Frequency

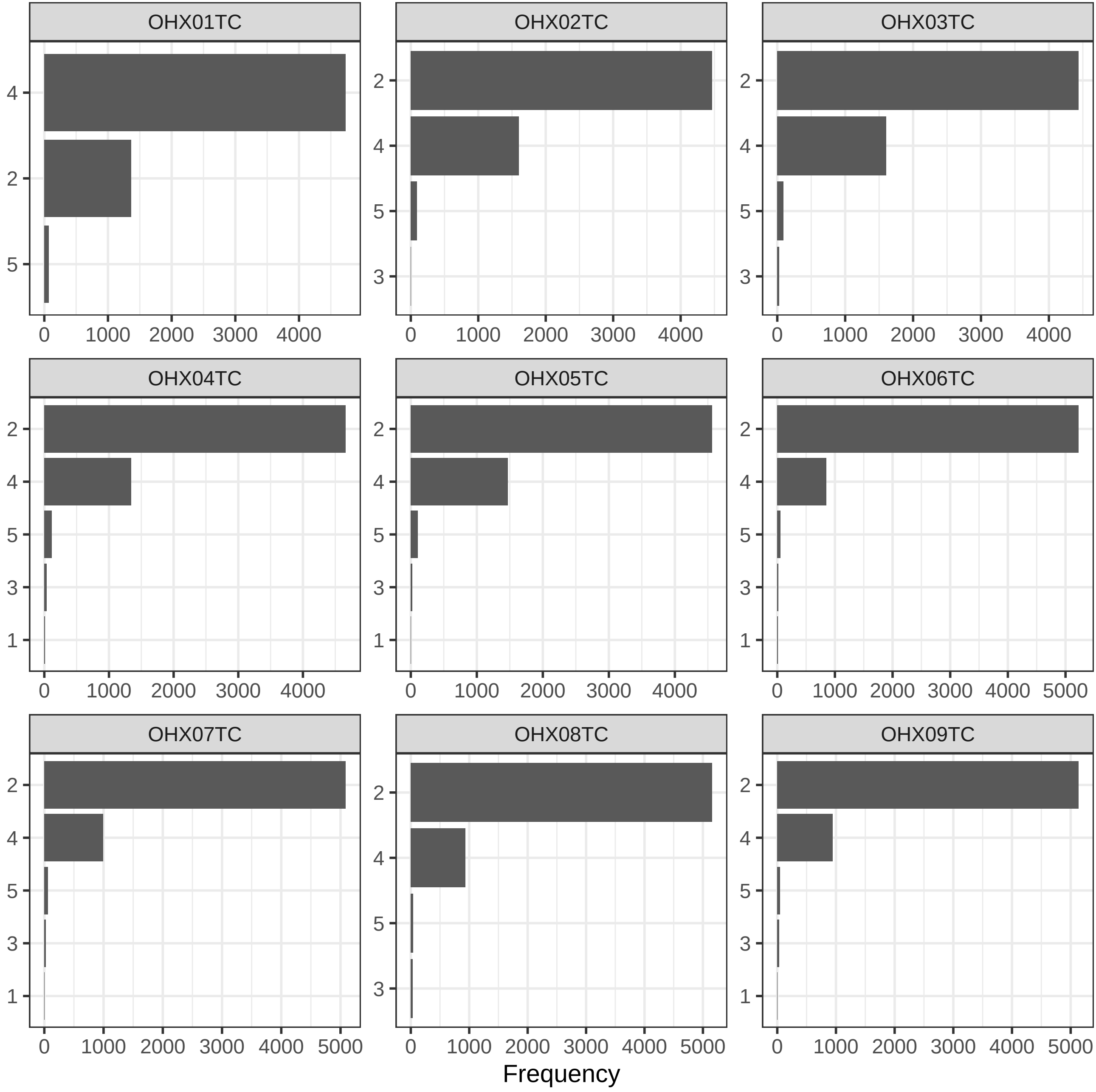
OHX01TC
4
2
5
0
1000
2000
3000
4000
OHX02TC
2
4
5
3
0
1000
2000
3000
4000
OHX03TC
2
4
5
3
0
1000
2000
3000
4000
OHX04TC
2
4
5
3
1
0
1000
2000
3000
4000
OHX05TC
2
4
5
3
1
0
1000
2000
3000
4000
OHX06TC
2
4
5
3
1
0
1000
2000
3000
4000
5000
OHX07TC
2
4
5
3
1
0
1000
2000
3000
4000
5000
OHX08TC
2
4
5
3
0
1000
2000
3000
4000
5000
OHX09TC
2
4
5
3
1
0
1000
2000
3000
4000
5000
Frequency

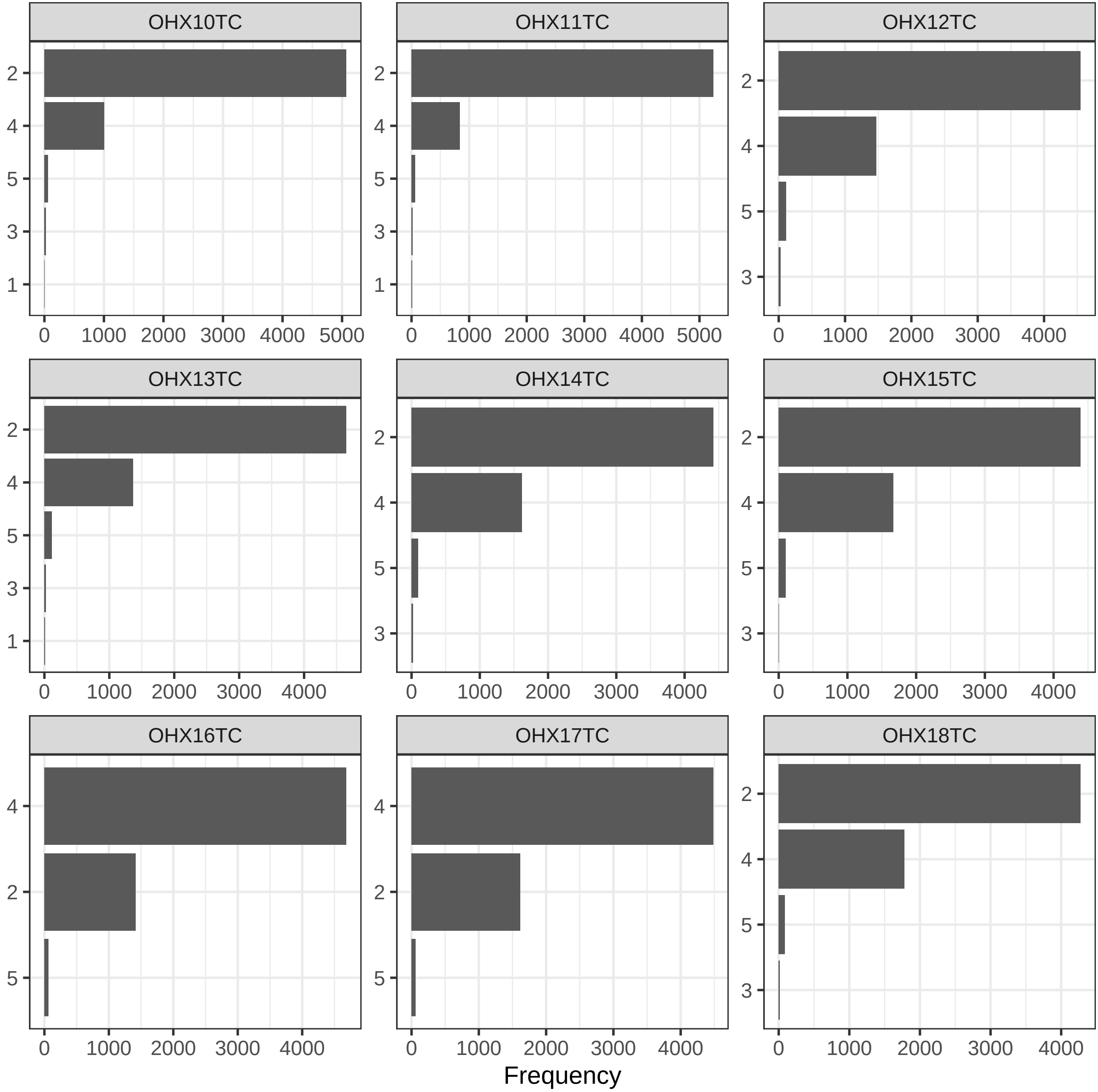

OHX10TC
OHX11TC
OHX12TC
OHX13TC
OHX14TC
OHX15TC
OHX16TC
OHX17TC
OHX18TC
Frequency

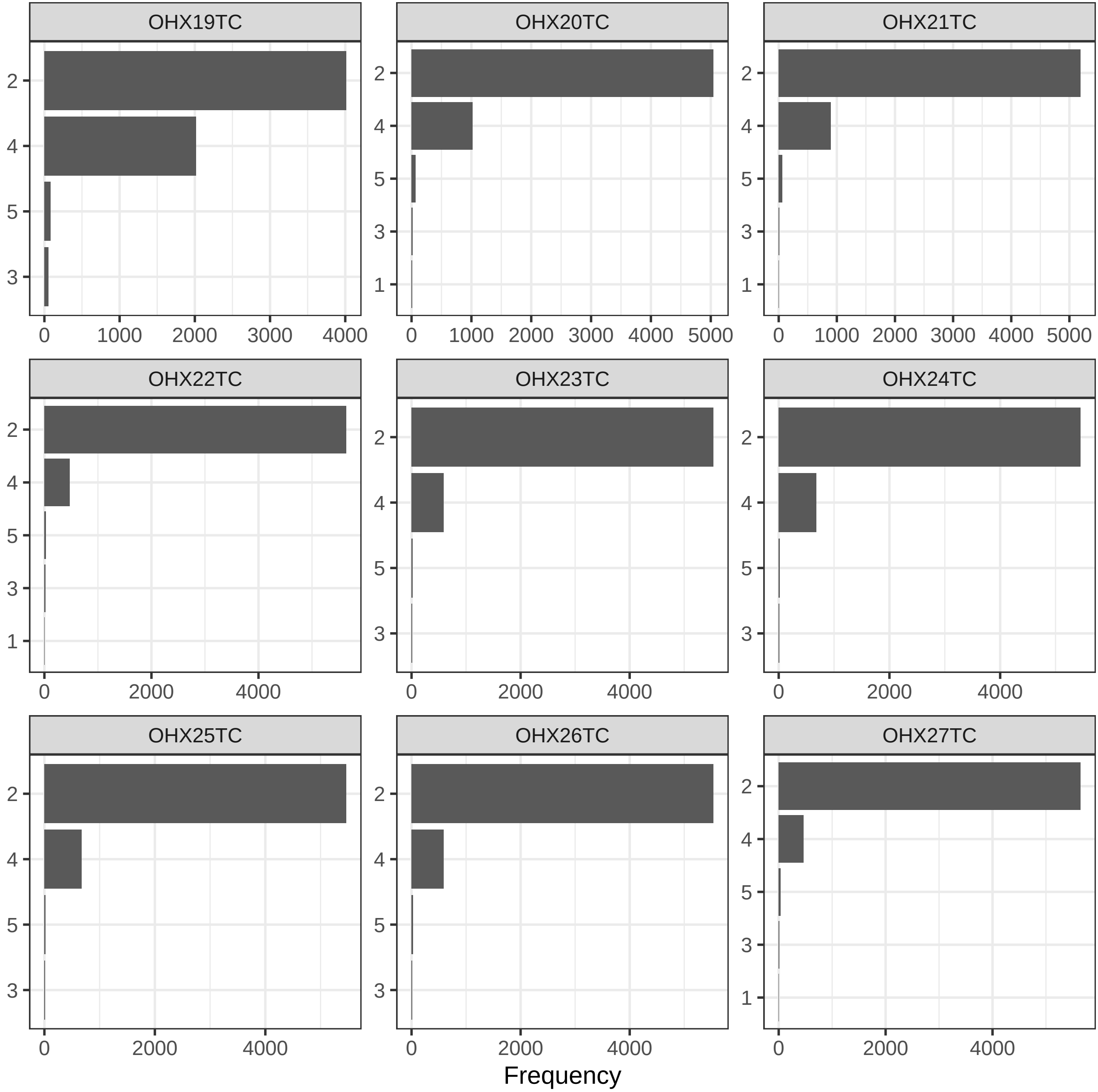
OHX19TC
OHX20TC
OHX21TC
OHX22TC
OHX23TC
OHX24TC
OHX25TC
OHX26TC
OHX27TC
Frequency

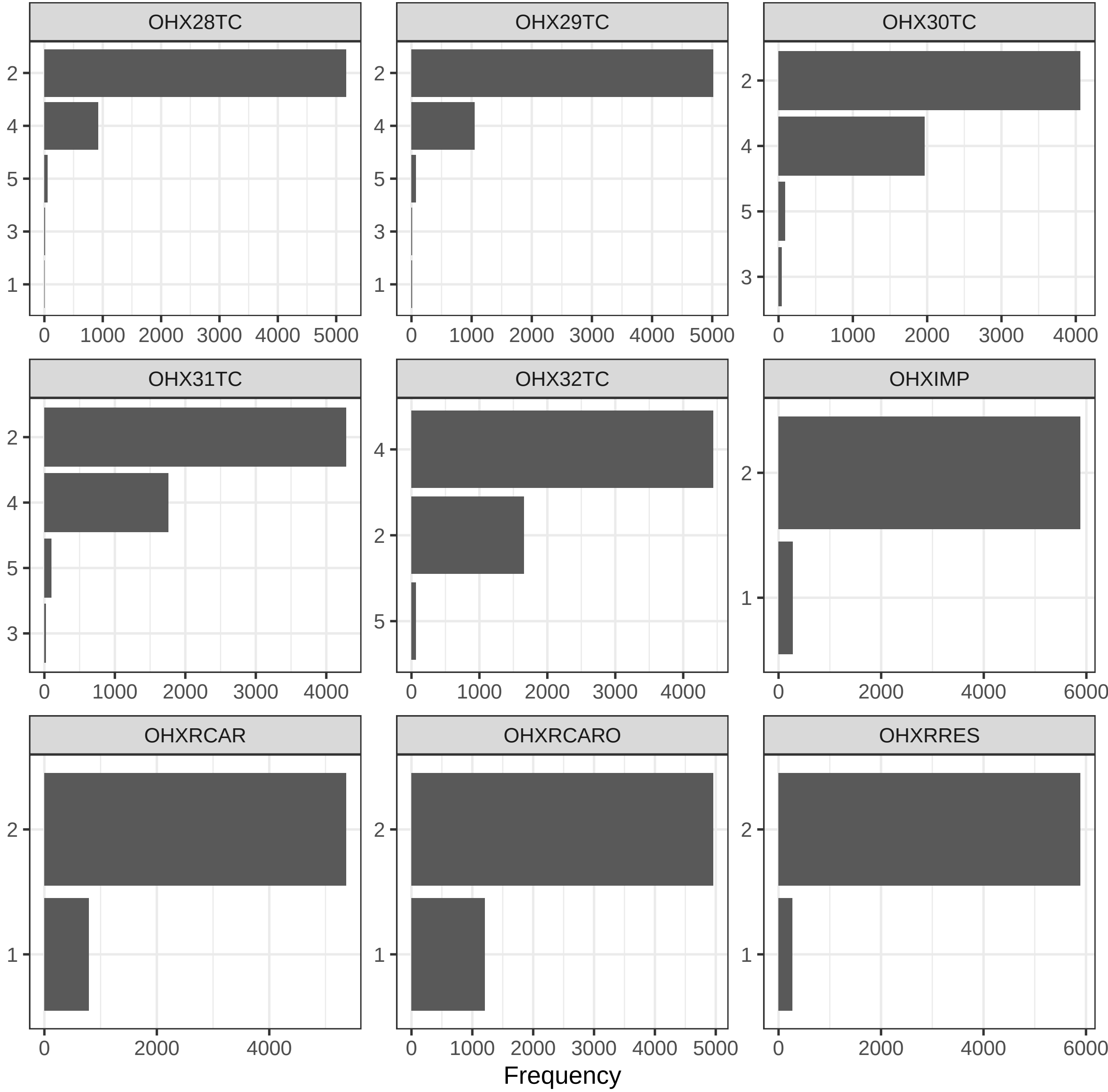

OHX28TC
OHX29TC
OHX30TC
OHX31TC
OHX32TC
OHXIMP
OHXRCAR
OHXRCARO
OHXRRES
Frequency

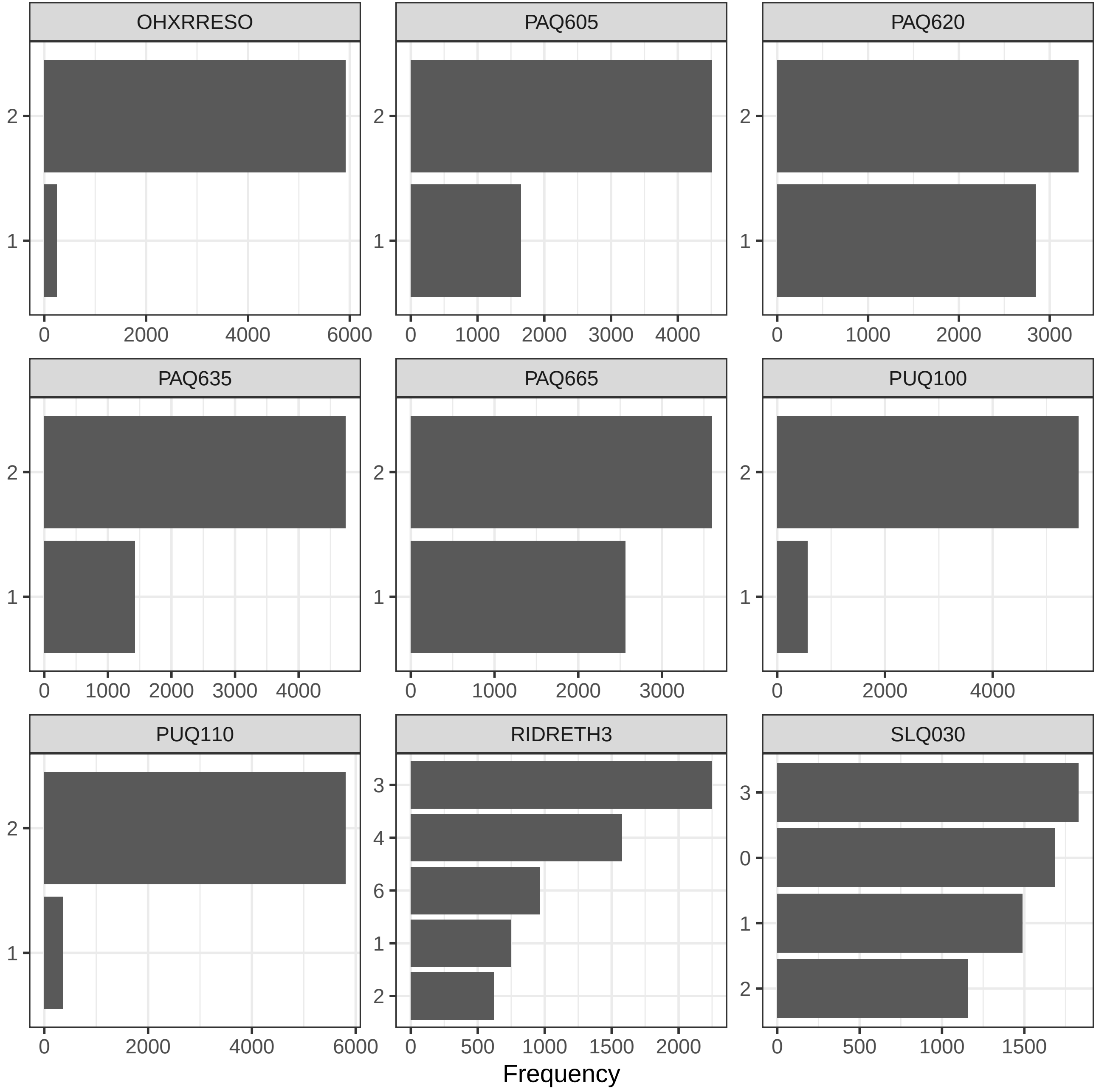

OHXRRESO
PAQ605
PAQ620
PAQ635
PAQ665
PUQ100
PUQ110
RIDRETH3
SLQ030
Frequency

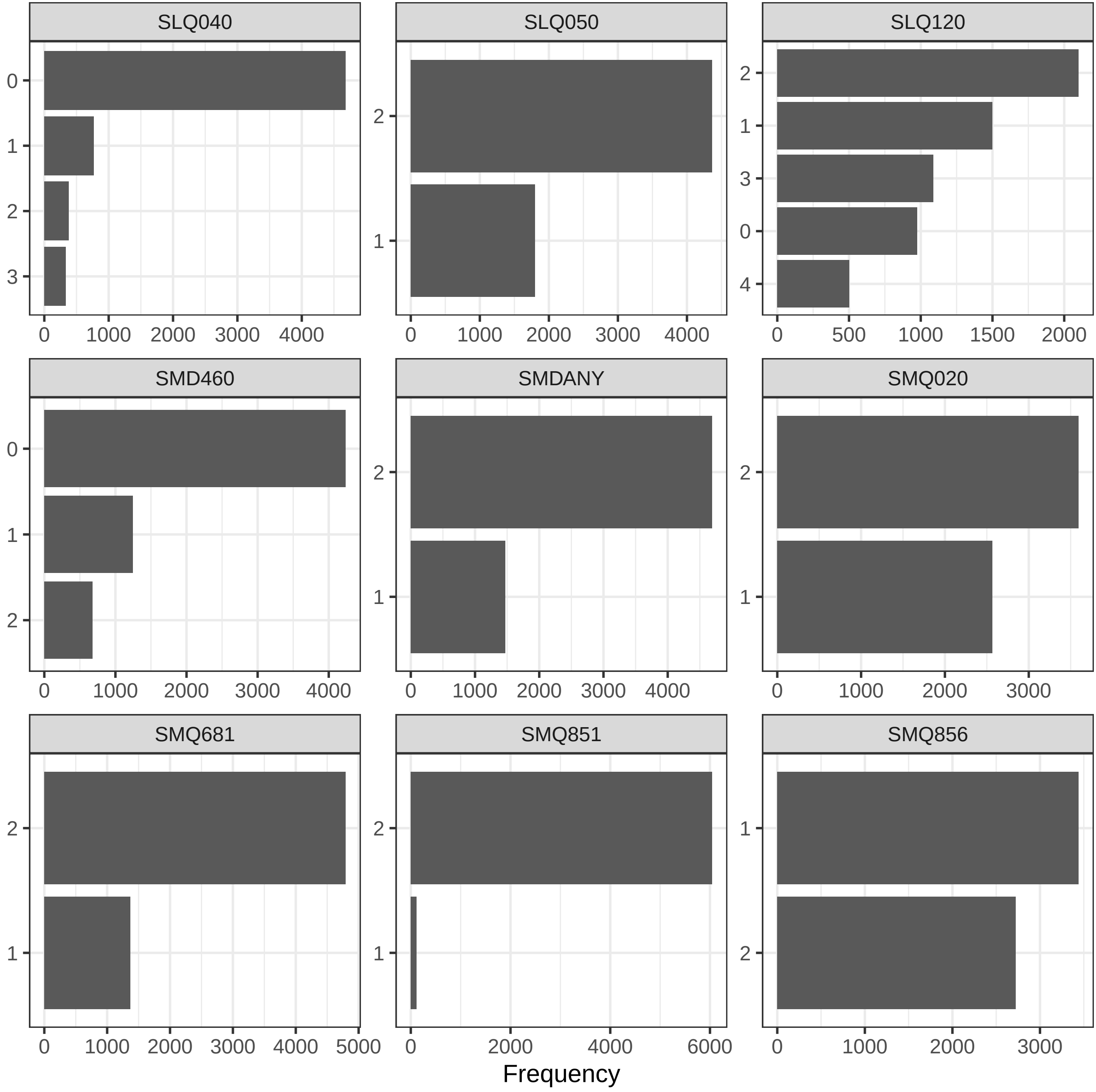
SLQ040
0
1
2
3
0 1000 2000 3000 4000
SLQ050
2
1
0 1000 2000 3000 4000
SLQ120
2
1
3
0
4
0 500 1000 1500 2000
SMD460
0
1
2
0 1000 2000 3000 4000
SMDANY
2
1
0 1000 2000 3000 4000
SMQ020
2
1
0 1000 2000 3000
SMQ681
2
1
0 1000 2000 3000 4000 5000
SMQ851
2
1
0 2000 4000 6000
SMQ856
1
2
0 1000 2000 3000
Frequency

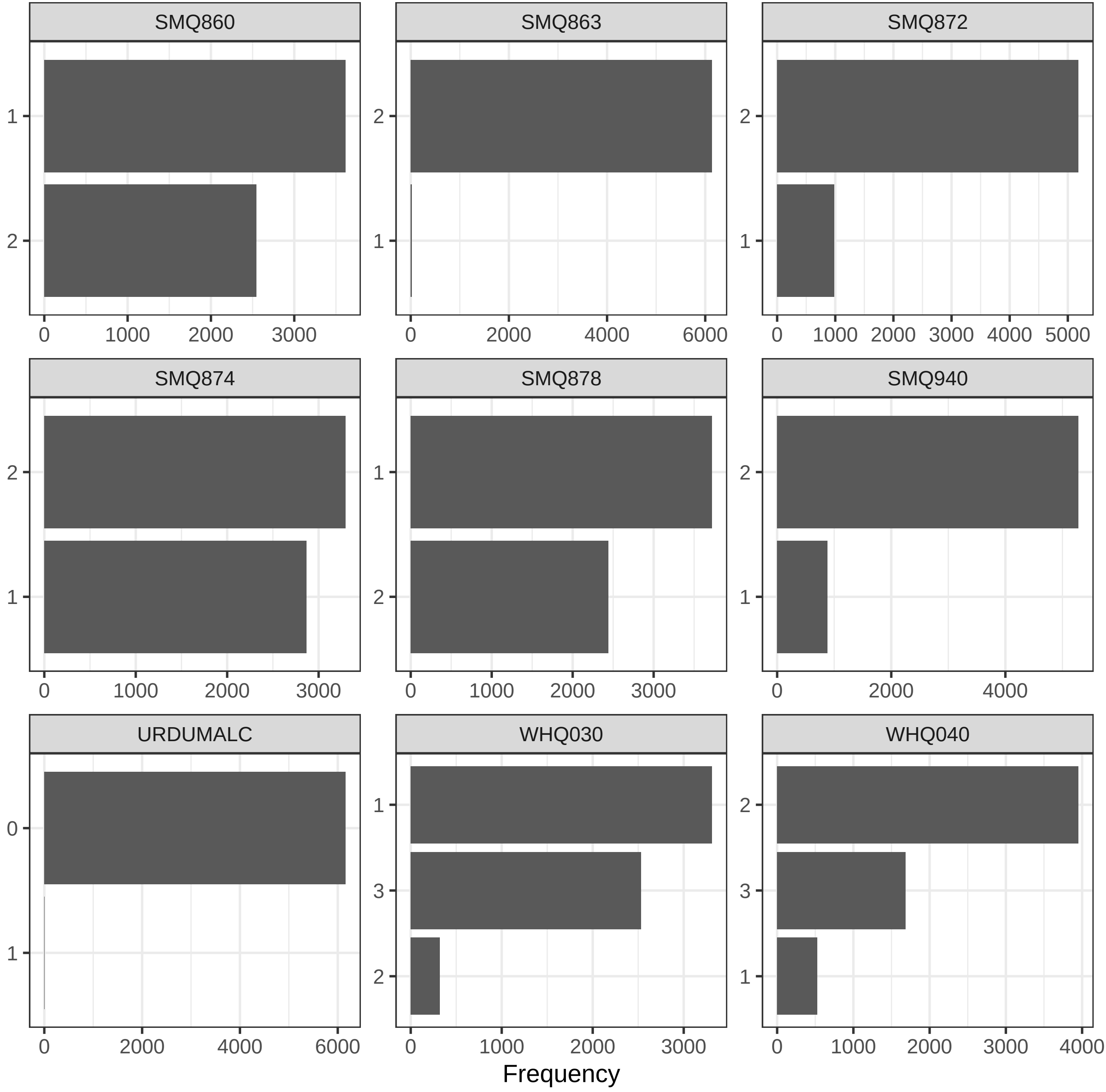
SMQ860
SMQ863
SMQ872
SMQ874
SMQ878
SMQ940
URDUMALC
WHQ030
WHQ040
Frequency

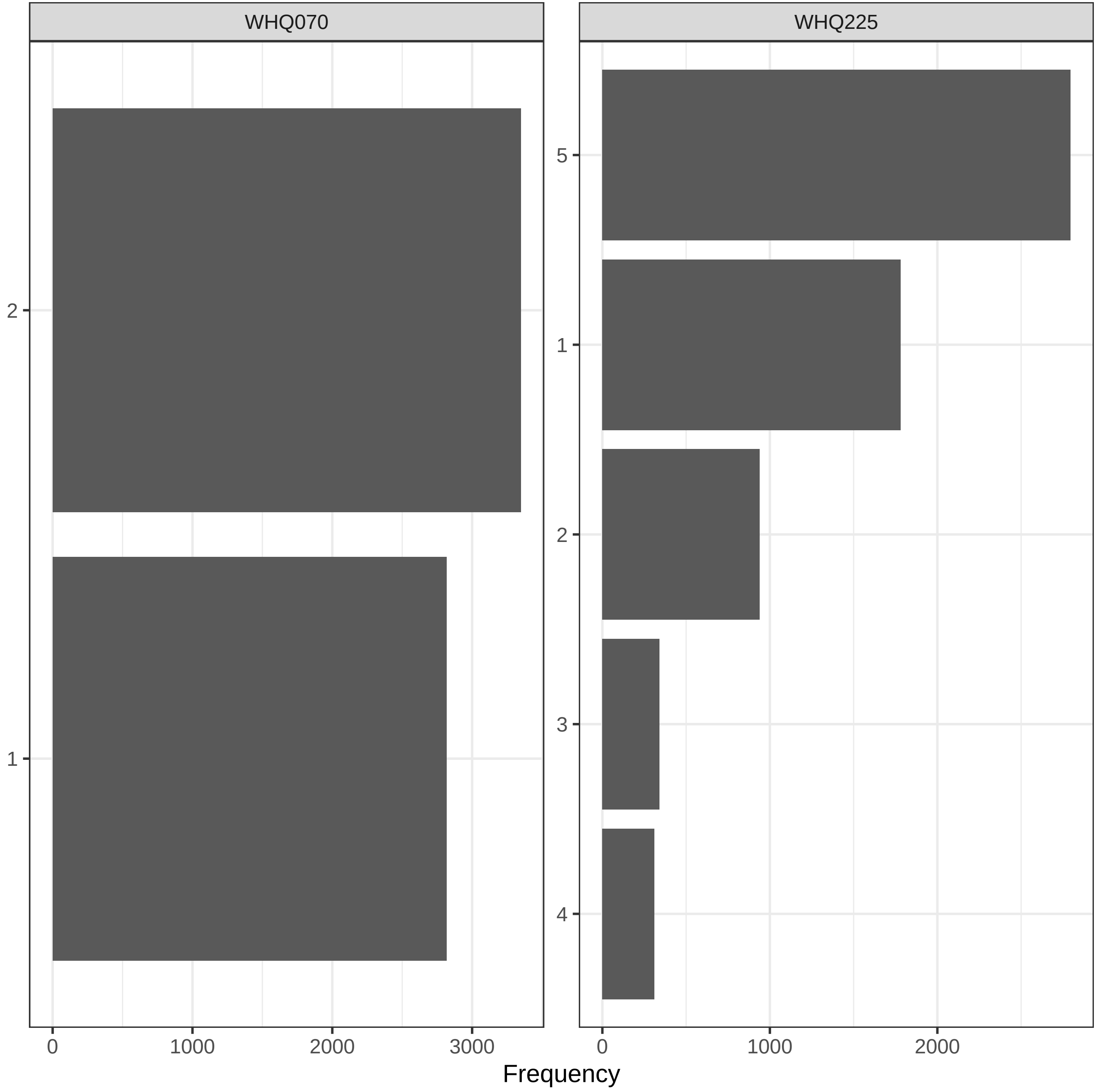
WHQ070
WHQ225
2
1
5
1
2
3
4
0
1000
2000
3000
0
1000
2000
Frequency
