# Supplementary material for: Integrative and interpretable machine learning framework for early non-invasive detection of clinically significant liver fibrosis
Source: Front Med (Lausanne). 2026 Jun 23;13:1736295. doi: 10.3389/fmed.2026.1736295 (PMC13337473; doi:10.3389/fmed.2026.1736295)
Supplement: Supplementary file 9 — This table provides detailed baseline demographic, anthropometric, and clinical laboratory characteristics for the entire NHANES 2017-2020 cohort (N = 6164) after data cleaning, Data are stratified according to clinically significant liver fibrosis status (Control vs. Case). Categorical variables are presented as n (%), and continuous variables are presented as mean ± standard deviation (SD) or median (interquartile range, IQR), as appropriate. Between-group differences were evaluated using Pearson's Chi-squared test or Fisher's exact test for categorical variables, and the Wilcoxon rank sum test for continuous variables. [file Data_Sheet_9.pdf]

Supplementary Table 1. Baseline Characteristics of the NHANES Training Cohort

Characteristic	Overall<br>N = 6,164[1]	Control<br>N = 5,458[1]	Case<br>N = 706[1]	p-value[2]
**RIAGENDR**				<0.001
1	3,041 (49%)	2,630 (48%)	411 (58%)	
2	3,123 (51%)	2,828 (52%)	295 (42%)	
**RIDRETH1**				0.016
1	751 (12%)	657 (12%)	94 (13%)	
2	619 (10%)	559 (10%)	60 (8.5%)	
3	2,251 (37%)	1,973 (36%)	278 (39%)	
4	1,579 (26%)	1,389 (25%)	190 (27%)	
5	964 (16%)	880 (16%)	84 (12%)	
**RIDRETH3**				0.008
1	751 (13%)	657 (13%)	94 (14%)	
2	619 (11%)	559 (11%)	60 (8.9%)	
3	2,251 (38%)	1,973 (38%)	278 (41%)	
4	1,579 (27%)	1,389 (27%)	190 (28%)	
6	667 (11%)	615 (12%)	52 (7.7%)	
**RIDEXMON**				0.5
1	3,261 (53%)	2,880 (53%)	381 (54%)	
2	2,903 (47%)	2,578 (47%)	325 (46%)	
**DMDBORN4**				<0.001
1	4,480 (73%)	3,923 (72%)	557 (79%)	
2	1,679 (27%)	1,530 (28%)	149 (21%)	
**DMDEDUC2**				<0.001
1	401 (6.7%)	352 (6.6%)	49 (7.0%)	
2	616 (10%)	547 (10%)	69 (9.9%)	
3	1,469 (24%)	1,258 (24%)	211 (30%)	
4	2,020 (34%)	1,777 (33%)	243 (35%)	
5	1,517 (25%)	1,391 (26%)	126 (18%)	
**DMDMARTZ**				<0.001

Characteristic	Overall<br>N = 6,164[1]	Control<br>N = 5,458[1]	Case<br>N = 706[1]	p-value[2]
1	3,487 (58%)	3,086 (58%)	401 (57%)	
2	1,343 (22%)	1,150 (22%)	193 (28%)	
3	1,194 (20%)	1,090 (20%)	104 (15%)	
**SIALANG**				0.2
1	5,547 (90%)	4,901 (90%)	646 (92%)	
2	617 (10%)	557 (10%)	60 (8.5%)	
**SIAPROXY**				0.5
1	49 (0.8%)	42 (0.8%)	7 (1.0%)	
2	6,115 (99%)	5,416 (99%)	699 (99%)	
**SIAINTRP**				0.004
1	135 (2.2%)	130 (2.4%)	5 (0.7%)	
2	6,029 (98%)	5,328 (98%)	701 (99%)	
**FIALANG**				0.3
1	5,312 (91%)	4,690 (91%)	622 (92%)	
2	534 (9.1%)	479 (9.3%)	55 (8.1%)	
**FIAPROXY**				0.024
1	6 (0.1%)	3 (<0.1%)	3 (0.4%)	
2	5,840 (100%)	5,166 (100%)	674 (100%)	
**FIAINTRP**				0.033
1	114 (2.0%)	108 (2.1%)	6 (0.9%)	
2	5,732 (98%)	5,061 (98%)	671 (99%)	
**MIALANG**				0.2
1	5,522 (91%)	4,881 (91%)	641 (92%)	
2	547 (9.0%)	494 (9.2%)	53 (7.6%)	
**MIAPROXY**				0.8
1	34 (0.6%)	31 (0.6%)	3 (0.4%)	
2	6,035 (99%)	5,344 (99%)	691 (100%)	
**MIAINTRP**				0.11
1	140 (2.3%)	130 (2.4%)	10 (1.4%)	

Characteristic	Overall N = 6,164[1]	Control N = 5,458[1]	Case N = 706[1]	p-value[2]
2	5,929 (98%)	5,245 (98%)	684 (99%)	
**SDMVPSU**				0.14
1	2,960 (48%)	2,604 (48%)	356 (50%)	
2	3,085 (50%)	2,743 (50%)	342 (48%)	
3	119 (1.9%)	111 (2.0%)	8 (1.1%)	
**URDUMALC**				>0.9
0	6,119 (100%)	5,422 (100%)	697 (100%)	
1	2 (<0.1%)	2 (<0.1%)	0 (0%)	
**ALQ111**				0.12
1	5,520 (91%)	4,877 (91%)	643 (93%)	
2	542 (8.9%)	491 (9.1%)	51 (7.3%)	
**ALQ151**				<0.001
1	850 (15%)	711 (15%)	139 (22%)	
2	4,662 (85%)	4,158 (85%)	504 (78%)	
**LBDSGTLC**				>0.9
0	6,150 (100%)	5,445 (100%)	705 (100%)	
1	1 (<0.1%)	1 (<0.1%)	0 (0%)	
**LBDSTBLC**				>0.9
0	6,152 (100%)	5,447 (100%)	705 (100%)	
1	1 (<0.1%)	1 (<0.1%)	0 (0%)	
**BMDSTATS**				<0.001
1	5,967 (97%)	5,306 (97%)	661 (94%)	
2	43 (0.7%)	36 (0.7%)	7 (1.0%)	
3	141 (2.3%)	105 (1.9%)	36 (5.1%)	
4	13 (0.2%)	11 (0.2%)	2 (0.3%)	
**BPQ020**				<0.001
1	2,295 (37%)	1,895 (35%)	400 (57%)	
2	3,861 (63%)	3,556 (65%)	305 (43%)	
**BPQ080**				0.001

Characteristic	Overall<br>N = 6,164[1]	Control<br>N = 5,458[1]	Case<br>N = 706[1]	p-value[2]
1	2,191 (36%)	1,904 (35%)	287 (41%)	
2	3,933 (64%)	3,524 (65%)	409 (59%)	
**BPAOCSZ**				<0.001
2	18 (0.3%)	17 (0.3%)	1 (0.1%)	
3	2,271 (39%)	2,133 (41%)	138 (21%)	
4	3,140 (54%)	2,751 (53%)	389 (58%)	
5	427 (7.3%)	283 (5.5%)	144 (21%)	
**LBDCOTLC**				0.6
0	4,103 (67%)	3,639 (67%)	464 (66%)	
1	2,060 (33%)	1,819 (33%)	241 (34%)	
**LBDHCOLC**				0.2
0	2,968 (48%)	2,612 (48%)	356 (50%)	
1	3,195 (52%)	2,846 (52%)	349 (50%)	
**DBQ700**				<0.001
1	434 (7.0%)	392 (7.2%)	42 (5.9%)	
2	1,231 (20%)	1,132 (21%)	99 (14%)	
3	2,414 (39%)	2,151 (39%)	263 (37%)	
4	1,645 (27%)	1,420 (26%)	225 (32%)	
5	440 (7.1%)	363 (6.7%)	77 (11%)	
**DBQ197**				0.2
0	1,354 (22%)	1,215 (22%)	139 (20%)	
1	1,366 (22%)	1,210 (22%)	156 (22%)	
2	1,776 (29%)	1,549 (28%)	227 (32%)	
3	1,652 (27%)	1,470 (27%)	182 (26%)	
4	16 (0.3%)	14 (0.3%)	2 (0.3%)	
**DBQ229**				>0.9
1	1,627 (27%)	1,442 (27%)	185 (27%)	
2	1,556 (26%)	1,374 (26%)	182 (26%)	
3	2,844 (47%)	2,513 (47%)	331 (47%)	

Characteristic	Overall<br>N = 6,164[1]	Control<br>N = 5,458[1]	Case<br>N = 706[1]	p-value[2]
**CBQ596**				0.9
1	1,382 (22%)	1,222 (22%)	160 (23%)	
2	4,768 (78%)	4,223 (78%)	545 (77%)	
**DBQ930**				>0.9
1	3,813 (62%)	3,378 (62%)	435 (62%)	
2	2,350 (38%)	2,080 (38%)	270 (38%)	
**DBQ935**				0.9
1	3,259 (53%)	2,888 (53%)	371 (53%)	
2	2,905 (47%)	2,570 (47%)	335 (47%)	
**DBQ940**				0.5
1	3,808 (62%)	3,381 (62%)	427 (60%)	
2	2,356 (38%)	2,077 (38%)	279 (40%)	
**DBQ945**				0.8
1	3,418 (55%)	3,023 (55%)	395 (56%)	
2	2,746 (45%)	2,435 (45%)	311 (44%)	
**DIQ010**				<0.001
1	898 (15%)	655 (12%)	243 (34%)	
2	5,079 (82%)	4,649 (85%)	430 (61%)	
3	186 (3.0%)	153 (2.8%)	33 (4.7%)	
**DIQ160**				<0.001
1	637 (13%)	557 (12%)	80 (19%)	
2	4,433 (87%)	4,085 (88%)	348 (81%)	
**DIQ180**				0.008
1	2,687 (53%)	2,425 (52%)	262 (59%)	
2	2,407 (47%)	2,223 (48%)	184 (41%)	
**DPQ010**				0.014
0	4,496 (75%)	3,991 (75%)	505 (73%)	
1	989 (16%)	888 (17%)	101 (15%)	
2	325 (5.4%)	276 (5.2%)	49 (7.1%)	

Characteristic	Overall<br>N = 6,164[1]	Control<br>N = 5,458[1]	Case<br>N = 706[1]	p-value[2]
3	217 (3.6%)	182 (3.4%)	35 (5.1%)	
**DPQ020**				0.3
0	4,503 (75%)	4,000 (75%)	503 (73%)	
1	1,057 (18%)	933 (17%)	124 (18%)	
2	278 (4.6%)	243 (4.6%)	35 (5.1%)	
3	190 (3.2%)	161 (3.0%)	29 (4.2%)	
**DPQ030**				0.011
0	3,658 (61%)	3,272 (61%)	386 (56%)	
1	1,392 (23%)	1,227 (23%)	165 (24%)	
2	446 (7.4%)	382 (7.2%)	64 (9.3%)	
3	531 (8.8%)	455 (8.5%)	76 (11%)	
**DPQ040**				0.054
0	3,016 (50%)	2,680 (50%)	336 (49%)	
1	2,010 (33%)	1,785 (33%)	225 (33%)	
2	517 (8.6%)	461 (8.6%)	56 (8.1%)	
3	484 (8.0%)	410 (7.7%)	74 (11%)	
**DPQ050**				0.031
0	4,483 (74%)	3,995 (75%)	488 (71%)	
1	958 (16%)	833 (16%)	125 (18%)	
2	321 (5.3%)	285 (5.3%)	36 (5.2%)	
3	265 (4.4%)	223 (4.2%)	42 (6.1%)	
**DPQ060**				0.6
0	4,998 (83%)	4,431 (83%)	567 (82%)	
1	712 (12%)	630 (12%)	82 (12%)	
2	179 (3.0%)	158 (3.0%)	21 (3.0%)	
3	139 (2.3%)	118 (2.2%)	21 (3.0%)	
**DPQ070**				0.9
0	4,983 (83%)	4,404 (83%)	579 (84%)	
1	665 (11%)	594 (11%)	71 (10%)	

Characteristic	Overall<br>N = 6,164[1]	Control<br>N = 5,458[1]	Case<br>N = 706[1]	p-value[2]
2	176 (2.9%)	157 (2.9%)	19 (2.7%)	
3	204 (3.4%)	182 (3.4%)	22 (3.2%)	
**DPQ080**				0.6
0	5,419 (90%)	4,799 (90%)	620 (90%)	
1	380 (6.3%)	341 (6.4%)	39 (5.6%)	
2	140 (2.3%)	121 (2.3%)	19 (2.7%)	
3	89 (1.5%)	76 (1.4%)	13 (1.9%)	
**DPQ090**				0.050
0	5,797 (96%)	5,144 (96%)	653 (95%)	
1	173 (2.9%)	143 (2.7%)	30 (4.3%)	
2	37 (0.6%)	33 (0.6%)	4 (0.6%)	
3	20 (0.3%)	16 (0.3%)	4 (0.6%)	
**DRDINT**				0.3
1	823 (13%)	737 (14%)	86 (12%)	
2	5,341 (87%)	4,721 (86%)	620 (88%)	
**DR1LANG**				0.2
1	5,465 (89%)	4,819 (88%)	646 (92%)	
2	598 (9.7%)	543 (9.9%)	55 (7.8%)	
3	25 (0.4%)	24 (0.4%)	1 (0.1%)	
4	38 (0.6%)	35 (0.6%)	3 (0.4%)	
5	23 (0.4%)	22 (0.4%)	1 (0.1%)	
6	15 (0.2%)	15 (0.3%)	0 (0%)	
**DBQ095Z**				0.003
1	4,196 (69%)	3,738 (69%)	458 (66%)	
2	90 (1.5%)	84 (1.6%)	6 (0.9%)	
3	76 (1.2%)	59 (1.1%)	17 (2.4%)	
4	1,752 (29%)	1,534 (28%)	218 (31%)	
**DRQSPREP**				0.3
1	541 (8.9%)	473 (8.8%)	68 (9.8%)	

Characteristic	Overall<br>N = 6,164[1]	Control<br>N = 5,458[1]	Case<br>N = 706[1]	p-value[2]
2	1,080 (18%)	946 (18%)	134 (19%)	
3	1,917 (32%)	1,691 (31%)	226 (32%)	
4	2,542 (42%)	2,273 (42%)	269 (39%)	
**DR1STY**				>0.9
1	1,196 (19%)	1,059 (19%)	137 (19%)	
2	4,960 (81%)	4,391 (81%)	569 (81%)	
**DRQSDIET**				<0.001
1	1,012 (16%)	858 (16%)	154 (22%)	
2	5,148 (84%)	4,596 (84%)	552 (78%)	
**DR1_300**				0.3
1	496 (8.1%)	440 (8.1%)	56 (7.9%)	
2	4,527 (74%)	3,992 (73%)	535 (76%)	
3	1,120 (18%)	1,005 (18%)	115 (16%)	
**DRD340**				0.020
1	3,272 (53%)	2,925 (54%)	347 (49%)	
2	2,856 (47%)	2,499 (46%)	357 (51%)	
**DRD360**				0.8
1	4,220 (69%)	3,737 (69%)	483 (69%)	
2	1,910 (31%)	1,688 (31%)	222 (31%)	
**DR2DRSTZ**				0.057
1	5,341 (87%)	4,721 (86%)	620 (88%)	
2	19 (0.3%)	14 (0.3%)	5 (0.7%)	
5	804 (13%)	723 (13%)	81 (11%)	
**DS1DS**				0.085
1	2,550 (42%)	2,236 (41%)	314 (45%)	
2	3,590 (58%)	3,199 (59%)	391 (55%)	
**DS1ANCNT**				0.003
0	5,962 (97%)	5,294 (97%)	668 (95%)	
1	196 (3.2%)	159 (2.9%)	37 (5.2%)	

Characteristic	Overall<br>N = 6,164[1]	Control<br>N = 5,458[1]	Case<br>N = 706[1]	p-value[2]
2	3 (<0.1%)	2 (<0.1%)	1 (0.1%)	
**DS1AN**				<0.001
1	199 (3.2%)	161 (3.0%)	38 (5.4%)	
2	5,962 (97%)	5,294 (97%)	668 (95%)	
**DSDANCNT**				0.042
0	5,261 (85%)	4,680 (86%)	581 (82%)	
1	876 (14%)	757 (14%)	119 (17%)	
2	23 (0.4%)	18 (0.3%)	5 (0.7%)	
3	1 (<0.1%)	1 (<0.1%)	0 (0%)	
**DSD010**				0.3
1	3,415 (55%)	3,011 (55%)	404 (57%)	
2	2,744 (45%)	2,443 (45%)	301 (43%)	
**DSD010AN**				0.017
1	900 (15%)	776 (14%)	124 (18%)	
2	5,261 (85%)	4,680 (86%)	581 (82%)	
**PHQ020**				0.3
1	107 (1.7%)	98 (1.8%)	9 (1.3%)	
2	6,057 (98%)	5,360 (98%)	697 (99%)	
**PHQ030**				0.11
1	48 (0.8%)	39 (0.7%)	9 (1.3%)	
2	6,116 (99%)	5,419 (99%)	697 (99%)	
**PHQ040**				0.029
1	175 (2.8%)	164 (3.0%)	11 (1.6%)	
2	5,989 (97%)	5,294 (97%)	695 (98%)	
**PHQ050**				0.13
1	70 (1.1%)	58 (1.1%)	12 (1.7%)	
2	6,094 (99%)	5,400 (99%)	694 (98%)	
**PHQ060**				0.2
1	163 (2.6%)	139 (2.5%)	24 (3.4%)	

Characteristic	Overall<br>N = 6,164[1]	Control<br>N = 5,458[1]	Case<br>N = 706[1]	p-value[2]
2	6,001 (97%)	5,319 (97%)	682 (97%)	
**PHDSESN**				0.9
0	3,075 (50%)	2,728 (50%)	347 (49%)	
1	2,229 (36%)	1,968 (36%)	261 (37%)	
2	860 (14%)	762 (14%)	98 (14%)	
**LBDUIBLC**				0.036
0	6,134 (100%)	5,433 (100%)	701 (100%)	
1	3 (<0.1%)	1 (<0.1%)	2 (0.3%)	
**FSDHH**				0.10
1	3,648 (62%)	3,242 (63%)	406 (60%)	
2	823 (14%)	716 (14%)	107 (16%)	
3	826 (14%)	716 (14%)	110 (16%)	
4	553 (9.5%)	498 (9.6%)	55 (8.1%)	
**FSDAD**				0.2
1	3,674 (63%)	3,264 (63%)	410 (60%)	
2	854 (15%)	747 (14%)	107 (16%)	
3	735 (13%)	635 (12%)	100 (15%)	
4	587 (10%)	526 (10%)	61 (9.0%)	
**FSD151**				0.5
1	756 (13%)	663 (13%)	93 (14%)	
2	5,095 (87%)	4,510 (87%)	585 (86%)	
**FSQ165**				0.4
1	2,507 (43%)	2,207 (43%)	300 (45%)	
2	3,315 (57%)	2,941 (57%)	374 (55%)	
**FSD162**				<0.001
1	511 (8.7%)	475 (9.2%)	36 (5.3%)	
2	5,333 (91%)	4,692 (91%)	641 (95%)	
**LBXHA**				0.2
1	2,878 (47%)	2,541 (47%)	337 (48%)	

Characteristic	Overall<br>N = 6,164[1]	Control<br>N = 5,458[1]	Case<br>N = 706[1]	p-value[2]
2	3,210 (53%)	2,851 (53%)	359 (51%)	
3	7 (0.1%)	5 (<0.1%)	2 (0.3%)	
**LBXHBS**				<0.001
1	1,608 (26%)	1,476 (27%)	132 (19%)	
2	4,546 (74%)	3,974 (73%)	572 (81%)	
**LBXHBC**				0.002
1	450 (7.3%)	377 (6.9%)	73 (10%)	
2	5,714 (93%)	5,081 (93%)	633 (90%)	
3	0 (0%)	0 (0%)	0 (0%)	
**LBXHCR**				<0.001
1	70 (1.1%)	39 (0.7%)	31 (4.4%)	
2	107 (1.8%)	81 (1.5%)	26 (3.7%)	
3	5,929 (97%)	5,286 (98%)	643 (92%)	
**LBDHCI**				<0.001
1	70 (1.1%)	46 (0.9%)	24 (3.4%)	
2	29 (0.5%)	28 (0.5%)	1 (0.1%)	
3	5,929 (97%)	5,286 (98%)	643 (92%)	
4	70 (1.1%)	39 (0.7%)	31 (4.4%)	
**LBDHEG**				0.2
1	586 (9.5%)	509 (9.3%)	77 (11%)	
2	5,577 (90%)	4,948 (91%)	629 (89%)	
**LBDHEM**				0.7
1	125 (2.0%)	112 (2.1%)	13 (1.8%)	
2	6,036 (98%)	5,343 (98%)	693 (98%)	
**HEQ010**				0.012
1	72 (1.2%)	57 (1.0%)	15 (2.1%)	
2	6,071 (99%)	5,384 (99%)	687 (98%)	
**HEQ030**				<0.001
1	117 (1.9%)	74 (1.4%)	43 (6.1%)	

Characteristic	Overall N = 6,164[1]	Control N = 5,458[1]	Case N = 706[1]	p-value[2]
2	6,025 (98%)	5,368 (99%)	657 (94%)	
**HIQ011**				0.067
1	5,155 (84%)	4,549 (83%)	606 (86%)	
2	996 (16%)	899 (17%)	97 (14%)	
**HIQ270**				0.5
1	4,753 (93%)	4,190 (93%)	563 (94%)	
2	339 (6.7%)	303 (6.7%)	36 (6.0%)	
**HIQ210**				0.7
1	406 (7.9%)	361 (7.9%)	45 (7.4%)	
2	4,757 (92%)	4,195 (92%)	562 (93%)	
**LBDHRPLC**				0.011
0	6,091 (99%)	5,386 (99%)	705 (100%)	
1	50 (0.8%)	50 (0.9%)	0 (0%)	
**HSQ590**				0.5
1	2,479 (42%)	2,205 (42%)	274 (41%)	
2	3,413 (58%)	3,017 (58%)	396 (59%)	
**HUQ010**				<0.001
1	703 (11%)	667 (12%)	36 (5.1%)	
2	1,705 (28%)	1,561 (29%)	144 (20%)	
3	2,308 (37%)	2,040 (37%)	268 (38%)	
4	1,228 (20%)	1,011 (19%)	217 (31%)	
5	213 (3.5%)	174 (3.2%)	39 (5.5%)	
**HUQ030**				0.033
1	5,100 (83%)	4,491 (82%)	609 (86%)	
2	1,023 (17%)	929 (17%)	94 (13%)	
3	38 (0.6%)	35 (0.6%)	3 (0.4%)	
**HUQ071**				<0.001
1	641 (10%)	537 (9.8%)	104 (15%)	
2	5,522 (90%)	4,920 (90%)	602 (85%)	

Characteristic	Overall<br>N = 6,164[1]	Control<br>N = 5,458[1]	Case<br>N = 706[1]	p-value[2]
**HUQ090**				0.6
1	662 (11%)	582 (11%)	80 (11%)	
2	5,501 (89%)	4,875 (89%)	626 (89%)	
**LBDIHGLC**				0.004
0	1,549 (25%)	1,403 (26%)	146 (21%)	
1	4,608 (75%)	4,050 (74%)	558 (79%)	
**LBDBGELC**				0.8
0	35 (0.6%)	32 (0.6%)	3 (0.4%)	
1	6,122 (99%)	5,421 (99%)	701 (100%)	
**LBDBGMLC**				0.7
0	4,187 (68%)	3,712 (68%)	475 (67%)	
1	1,970 (32%)	1,741 (32%)	229 (33%)	
**IMQ011**				<0.001
1	1,438 (28%)	1,313 (29%)	125 (20%)	
2	212 (4.1%)	188 (4.2%)	24 (3.9%)	
3	3,501 (68%)	3,028 (67%)	473 (76%)	
**IMQ020**				<0.001
1	1,669 (31%)	1,519 (32%)	150 (24%)	
2	233 (4.3%)	211 (4.5%)	22 (3.5%)	
3	3,467 (65%)	3,002 (63%)	465 (73%)	
**INDFMMPC**				0.7
1	1,731 (31%)	1,521 (31%)	210 (32%)	
2	851 (15%)	758 (15%)	93 (14%)	
3	2,984 (54%)	2,637 (54%)	347 (53%)	
**KIQ022**				<0.001
1	235 (3.9%)	186 (3.5%)	49 (7.0%)	
2	5,785 (96%)	5,136 (97%)	649 (93%)	
**KIQ026**				0.001
1	585 (9.7%)	494 (9.3%)	91 (13%)	

Characteristic	Overall<br>N = 6,164[1]	Control<br>N = 5,458[1]	Case<br>N = 706[1]	p-value[2]
2	5,433 (90%)	4,828 (91%)	605 (87%)	
**KIQ005**				<0.001
1	3,821 (65%)	3,424 (65%)	397 (58%)	
2	687 (12%)	616 (12%)	71 (10%)	
3	602 (10%)	515 (9.8%)	87 (13%)	
4	394 (6.7%)	330 (6.3%)	64 (9.3%)	
5	417 (7.0%)	351 (6.7%)	66 (9.6%)	
**KIQ042**				0.020
1	1,462 (25%)	1,268 (24%)	194 (28%)	
2	4,459 (75%)	3,967 (76%)	492 (72%)	
**KIQ044**				<0.001
1	1,551 (26%)	1,322 (25%)	229 (33%)	
2	4,370 (74%)	3,914 (75%)	456 (67%)	
**KIQ046**				0.039
1	546 (9.2%)	468 (8.9%)	78 (11%)	
2	5,372 (91%)	4,764 (91%)	608 (89%)	
**KIQ480**				<0.001
0	1,608 (27%)	1,482 (28%)	126 (18%)	
1	2,250 (38%)	2,005 (38%)	245 (36%)	
2	1,140 (19%)	989 (19%)	151 (22%)	
3	584 (9.9%)	480 (9.2%)	104 (15%)	
4	180 (3.0%)	142 (2.7%)	38 (5.5%)	
5	155 (2.6%)	134 (2.6%)	21 (3.1%)	
**LUAXSTAT**				<0.001
1	5,823 (94%)	5,221 (96%)	602 (85%)	
2	341 (5.5%)	237 (4.3%)	104 (15%)	
**MCQ010**				0.13
1	977 (16%)	851 (16%)	126 (18%)	
2	5,182 (84%)	4,602 (84%)	580 (82%)	

Characteristic	Overall<br>N = 6,164[1]	Control<br>N = 5,458[1]	Case<br>N = 706[1]	p-value[2]
**MCQ053**				0.7
1	306 (5.0%)	269 (4.9%)	37 (5.2%)	
2	5,849 (95%)	5,180 (95%)	669 (95%)	
**MCQ080**				<0.001
1	2,521 (41%)	2,057 (38%)	464 (66%)	
2	3,636 (59%)	3,395 (62%)	241 (34%)	
**MCQ092**				<0.001
1	660 (11%)	553 (10%)	107 (15%)	
2	5,430 (89%)	4,844 (90%)	586 (85%)	
**MCQ160A**				<0.001
1	1,833 (30%)	1,557 (29%)	276 (40%)	
2	4,186 (70%)	3,765 (71%)	421 (60%)	
**MCQ160B**				<0.001
1	179 (3.0%)	130 (2.4%)	49 (7.1%)	
2	5,838 (97%)	5,192 (98%)	646 (93%)	
**MCQ160C**				0.003
1	245 (4.1%)	202 (3.8%)	43 (6.2%)	
2	5,764 (96%)	5,114 (96%)	650 (94%)	
**MCQ160D**				0.004
1	135 (2.2%)	109 (2.1%)	26 (3.8%)	
2	5,871 (98%)	5,207 (98%)	664 (96%)	
**MCQ160E**				<0.001
1	245 (4.1%)	199 (3.7%)	46 (6.6%)	
2	5,775 (96%)	5,124 (96%)	651 (93%)	
**MCQ160F**				0.002
1	270 (4.5%)	223 (4.2%)	47 (6.8%)	
2	5,749 (96%)	5,100 (96%)	649 (93%)	
**MCQ160M**				0.5
1	706 (12%)	630 (12%)	76 (11%)	

Characteristic	Overall<br>N = 6,164[1]	Control<br>N = 5,458[1]	Case<br>N = 706[1]	p-value[2]
2	5,312 (88%)	4,693 (88%)	619 (89%)	
**MCQ160P**				0.002
1	523 (8.7%)	441 (8.3%)	82 (12%)	
2	5,498 (91%)	4,883 (92%)	615 (88%)	
**MCQ160L**				<0.001
1	315 (5.2%)	236 (4.4%)	79 (11%)	
2	5,701 (95%)	5,086 (96%)	615 (89%)	
**MCQ520**				0.017
1	1,351 (22%)	1,170 (22%)	181 (26%)	
2	4,674 (78%)	4,158 (78%)	516 (74%)	
**MCQ550**				<0.001
1	639 (11%)	517 (9.7%)	122 (17%)	
2	5,384 (89%)	4,808 (90%)	576 (83%)	
**MCQ560**				<0.001
1	659 (11%)	529 (9.9%)	130 (19%)	
2	5,368 (89%)	4,800 (90%)	568 (81%)	
**MCQ220**				0.081
1	614 (10%)	530 (9.9%)	84 (12%)	
2	5,412 (90%)	4,800 (90%)	612 (88%)	
**MCQ300B**				0.6
1	1,566 (26%)	1,381 (26%)	185 (27%)	
2	4,474 (74%)	3,966 (74%)	508 (73%)	
**MCQ300C**				<0.001
1	2,914 (49%)	2,500 (47%)	414 (60%)	
2	3,037 (51%)	2,765 (53%)	272 (40%)	
**MCQ300A**				0.018
1	807 (14%)	694 (13%)	113 (17%)	
2	5,086 (86%)	4,519 (87%)	567 (83%)	
**MCQ366A**				<0.001

Characteristic	Overall<br>N = 6,164[1]	Control<br>N = 5,458[1]	Case<br>N = 706[1]	p-value[2]
1	1,809 (29%)	1,434 (26%)	375 (53%)	
2	4,353 (71%)	4,022 (74%)	331 (47%)	
**MCQ366B**				<0.001
1	2,617 (42%)	2,170 (40%)	447 (63%)	
2	3,544 (58%)	3,287 (60%)	257 (37%)	
**MCQ366C**				<0.001
1	1,810 (29%)	1,494 (27%)	316 (45%)	
2	4,350 (71%)	3,961 (73%)	389 (55%)	
**MCQ366D**				<0.001
1	1,985 (32%)	1,618 (30%)	367 (52%)	
2	4,178 (68%)	3,839 (70%)	339 (48%)	
**MCQ371A**				<0.001
1	3,969 (64%)	3,463 (63%)	506 (72%)	
2	2,192 (36%)	1,992 (37%)	200 (28%)	
**MCQ371B**				0.3
1	3,679 (60%)	3,244 (59%)	435 (62%)	
2	2,483 (40%)	2,212 (41%)	271 (38%)	
**MCQ371C**				<0.001
1	3,435 (56%)	2,972 (54%)	463 (66%)	
2	2,728 (44%)	2,485 (46%)	243 (34%)	
**MCQ371D**				<0.001
1	3,647 (59%)	3,185 (58%)	462 (66%)	
2	2,515 (41%)	2,272 (42%)	243 (34%)	
**OCD150**				<0.001
1	3,518 (57%)	3,173 (58%)	345 (49%)	
2	140 (2.3%)	122 (2.2%)	18 (2.5%)	
3	248 (4.0%)	223 (4.1%)	25 (3.5%)	
4	2,256 (37%)	1,938 (36%)	318 (45%)	
**OHQ033**				0.015

Characteristic	Overall<br>N = 6,164[1]	Control<br>N = 5,458[1]	Case<br>N = 706[1]	p-value[2]
1	3,193 (53%)	2,869 (53%)	324 (47%)	
2	469 (7.7%)	413 (7.7%)	56 (8.1%)	
3	1,723 (28%)	1,492 (28%)	231 (33%)	
4	515 (8.5%)	453 (8.4%)	62 (9.0%)	
5	159 (2.6%)	142 (2.6%)	17 (2.5%)	
**OHQ770**				0.033
1	1,362 (22%)	1,185 (22%)	177 (26%)	
2	4,708 (78%)	4,194 (78%)	514 (74%)	
**OHQ620**				0.4
1	173 (3.4%)	149 (3.3%)	24 (3.7%)	
2	233 (4.5%)	205 (4.6%)	28 (4.3%)	
3	958 (19%)	835 (19%)	123 (19%)	
4	1,714 (33%)	1,476 (33%)	238 (36%)	
5	2,056 (40%)	1,813 (40%)	243 (37%)	
**OHQ640**				0.12
1	57 (1.1%)	50 (1.1%)	7 (1.1%)	
2	52 (1.0%)	45 (1.0%)	7 (1.1%)	
3	150 (2.9%)	137 (3.1%)	13 (2.0%)	
4	512 (10.0%)	430 (9.6%)	82 (13%)	
5	4,363 (85%)	3,816 (85%)	547 (83%)	
**OHQ835**				0.003
1	1,036 (20%)	876 (20%)	160 (25%)	
2	4,045 (80%)	3,561 (80%)	484 (75%)	
**OHQ845**				<0.001
1	645 (10%)	580 (11%)	65 (9.2%)	
2	1,402 (23%)	1,275 (23%)	127 (18%)	
3	2,060 (33%)	1,829 (34%)	231 (33%)	
4	1,427 (23%)	1,247 (23%)	180 (25%)	
5	623 (10%)	520 (9.5%)	103 (15%)	

Characteristic	Overall<br>N = 6,164[1]	Control<br>N = 5,458[1]	Case<br>N = 706[1]	p-value[2]
**OHQ850**				0.069
1	1,315 (26%)	1,128 (25%)	187 (29%)	
2	3,796 (74%)	3,330 (75%)	466 (71%)	
**OHQ860**				0.2
1	882 (17%)	759 (17%)	123 (19%)	
2	4,209 (83%)	3,685 (83%)	524 (81%)	
**OHDEXSTS**				>0.9
1	6,103 (99%)	5,403 (99%)	700 (99%)	
2	1 (<0.1%)	1 (<0.1%)	0 (0%)	
3	60 (1.0%)	54 (1.0%)	6 (0.8%)	
**OHDDESTS**				>0.9
1	6,103 (99%)	5,403 (99%)	700 (99%)	
2	1 (<0.1%)	1 (<0.1%)	0 (0%)	
3	60 (1.0%)	54 (1.0%)	6 (0.8%)	
**OHXIMP**				0.7
1	279 (4.6%)	249 (4.6%)	30 (4.3%)	
2	5,825 (95%)	5,155 (95%)	670 (96%)	
**OHX01TC**				0.018
2	1,362 (22%)	1,228 (23%)	134 (19%)	
4	4,674 (77%)	4,121 (76%)	553 (79%)	
5	68 (1.1%)	55 (1.0%)	13 (1.9%)	
**OHX02TC**				<0.001
2	4,408 (72%)	3,987 (74%)	421 (60%)	
3	3 (<0.1%)	3 (<0.1%)	0 (0%)	
4	1,603 (26%)	1,344 (25%)	259 (37%)	
5	90 (1.5%)	70 (1.3%)	20 (2.9%)	
**OHX03TC**				<0.001
2	4,379 (72%)	3,960 (73%)	419 (60%)	
3	27 (0.4%)	24 (0.4%)	3 (0.4%)	

Characteristic	Overall<br>N = 6,164[1]	Control<br>N = 5,458[1]	Case<br>N = 706[1]	p-value[2]
4	1,605 (26%)	1,345 (25%)	260 (37%)	
5	93 (1.5%)	75 (1.4%)	18 (2.6%)	
**OHX04TC**				<0.001
1	7 (0.1%)	7 (0.1%)	0 (0%)	
2	4,602 (75%)	4,145 (77%)	457 (65%)	
3	36 (0.6%)	32 (0.6%)	4 (0.6%)	
4	1,346 (22%)	1,127 (21%)	219 (31%)	
5	113 (1.9%)	93 (1.7%)	20 (2.9%)	
**OHX05TC**				0.012
1	1 (<0.1%)	1 (<0.1%)	0 (0%)	
2	4,506 (74%)	4,026 (75%)	480 (69%)	
3	21 (0.3%)	19 (0.4%)	2 (0.3%)	
4	1,470 (24%)	1,268 (23%)	202 (29%)	
5	106 (1.7%)	90 (1.7%)	16 (2.3%)	
**OHX06TC**				<0.001
1	9 (0.1%)	9 (0.2%)	0 (0%)	
2	5,169 (85%)	4,620 (85%)	549 (78%)	
3	16 (0.3%)	13 (0.2%)	3 (0.4%)	
4	854 (14%)	719 (13%)	135 (19%)	
5	56 (0.9%)	43 (0.8%)	13 (1.9%)	
**OHX07TC**				<0.001
1	1 (<0.1%)	1 (<0.1%)	0 (0%)	
2	5,028 (82%)	4,496 (83%)	532 (76%)	
3	22 (0.4%)	22 (0.4%)	0 (0%)	
4	995 (16%)	840 (16%)	155 (22%)	
5	58 (1.0%)	45 (0.8%)	13 (1.9%)	
**OHX08TC**				<0.001
2	5,099 (84%)	4,554 (84%)	545 (78%)	
3	32 (0.5%)	30 (0.6%)	2 (0.3%)	

Characteristic	Overall<br>N = 6,164[1]	Control<br>N = 5,458[1]	Case<br>N = 706[1]	p-value[2]
4	935 (15%)	791 (15%)	144 (21%)	
5	38 (0.6%)	29 (0.5%)	9 (1.3%)	
**OHX09TC**				<0.001
1	1 (<0.1%)	1 (<0.1%)	0 (0%)	
2	5,077 (83%)	4,547 (84%)	530 (76%)	
3	34 (0.6%)	31 (0.6%)	3 (0.4%)	
4	947 (16%)	796 (15%)	151 (22%)	
5	45 (0.7%)	29 (0.5%)	16 (2.3%)	
**OHX10TC**				<0.001
1	2 (<0.1%)	2 (<0.1%)	0 (0%)	
2	5,012 (82%)	4,484 (83%)	528 (75%)	
3	24 (0.4%)	23 (0.4%)	1 (0.1%)	
4	1,007 (16%)	847 (16%)	160 (23%)	
5	59 (1.0%)	48 (0.9%)	11 (1.6%)	
**OHX11TC**				<0.001
1	8 (0.1%)	7 (0.1%)	1 (0.1%)	
2	5,184 (85%)	4,635 (86%)	549 (78%)	
3	15 (0.2%)	14 (0.3%)	1 (0.1%)	
4	835 (14%)	699 (13%)	136 (19%)	
5	62 (1.0%)	49 (0.9%)	13 (1.9%)	
**OHX12TC**				<0.001
2	4,492 (74%)	4,018 (74%)	474 (68%)	
3	26 (0.4%)	25 (0.5%)	1 (0.1%)	
4	1,472 (24%)	1,262 (23%)	210 (30%)	
5	114 (1.9%)	99 (1.8%)	15 (2.1%)	
**OHX13TC**				<0.001
1	8 (0.1%)	7 (0.1%)	1 (0.1%)	
2	4,591 (75%)	4,131 (76%)	460 (66%)	
3	23 (0.4%)	21 (0.4%)	2 (0.3%)	

Characteristic	Overall<br>N = 6,164[1]	Control<br>N = 5,458[1]	Case<br>N = 706[1]	p-value[2]
4	1,367 (22%)	1,148 (21%)	219 (31%)	
5	115 (1.9%)	97 (1.8%)	18 (2.6%)	
**OHX14TC**				<0.001
2	4,364 (71%)	3,953 (73%)	411 (59%)	
3	23 (0.4%)	20 (0.4%)	3 (0.4%)	
4	1,619 (27%)	1,349 (25%)	270 (39%)	
5	98 (1.6%)	82 (1.5%)	16 (2.3%)	
**OHX15TC**				<0.001
2	4,334 (71%)	3,915 (72%)	419 (60%)	
3	3 (<0.1%)	3 (<0.1%)	0 (0%)	
4	1,667 (27%)	1,399 (26%)	268 (38%)	
5	100 (1.6%)	87 (1.6%)	13 (1.9%)	
**OHX16TC**				0.048
2	1,419 (23%)	1,282 (24%)	137 (20%)	
4	4,625 (76%)	4,070 (75%)	555 (79%)	
5	60 (1.0%)	52 (1.0%)	8 (1.1%)	
**OHX17TC**				0.061
2	1,614 (26%)	1,454 (27%)	160 (23%)	
4	4,428 (73%)	3,897 (72%)	531 (76%)	
5	62 (1.0%)	53 (1.0%)	9 (1.3%)	
**OHX18TC**				<0.001
2	4,216 (69%)	3,797 (70%)	419 (60%)	
3	18 (0.3%)	18 (0.3%)	0 (0%)	
4	1,780 (29%)	1,513 (28%)	267 (38%)	
5	90 (1.5%)	76 (1.4%)	14 (2.0%)	
**OHX19TC**				<0.001
2	3,955 (65%)	3,575 (66%)	380 (54%)	
3	51 (0.8%)	47 (0.9%)	4 (0.6%)	
4	2,018 (33%)	1,713 (32%)	305 (44%)	

Characteristic	Overall<br>N = 6,164[1]	Control<br>N = 5,458[1]	Case<br>N = 706[1]	p-value[2]
5	80 (1.3%)	69 (1.3%)	11 (1.6%)	
**OHX20TC**				<0.001
1	13 (0.2%)	12 (0.2%)	1 (0.1%)	
2	4,986 (82%)	4,471 (83%)	515 (74%)	
3	15 (0.2%)	15 (0.3%)	0 (0%)	
4	1,019 (17%)	841 (16%)	178 (25%)	
5	71 (1.2%)	65 (1.2%)	6 (0.9%)	
**OHX21TC**				0.2
1	1 (<0.1%)	1 (<0.1%)	0 (0%)	
2	5,134 (84%)	4,565 (84%)	569 (81%)	
3	12 (0.2%)	10 (0.2%)	2 (0.3%)	
4	898 (15%)	775 (14%)	123 (18%)	
5	59 (1.0%)	53 (1.0%)	6 (0.9%)	
**OHX22TC**				<0.001
1	2 (<0.1%)	1 (<0.1%)	1 (0.1%)	
2	5,582 (91%)	4,974 (92%)	608 (87%)	
3	18 (0.3%)	14 (0.3%)	4 (0.6%)	
4	471 (7.7%)	388 (7.2%)	83 (12%)	
5	31 (0.5%)	27 (0.5%)	4 (0.6%)	
**OHX23TC**				<0.001
2	5,478 (90%)	4,884 (90%)	594 (85%)	
3	15 (0.2%)	14 (0.3%)	1 (0.1%)	
4	593 (9.7%)	491 (9.1%)	102 (15%)	
5	18 (0.3%)	15 (0.3%)	3 (0.4%)	
**OHX24TC**				<0.001
2	5,394 (88%)	4,812 (89%)	582 (83%)	
3	9 (0.1%)	9 (0.2%)	0 (0%)	
4	680 (11%)	566 (10%)	114 (16%)	
5	21 (0.3%)	17 (0.3%)	4 (0.6%)	

Characteristic	Overall<br>N = 6,164[1]	Control<br>N = 5,458[1]	Case<br>N = 706[1]	p-value[2]
**OHX25TC**				<0.001
2	5,407 (89%)	4,823 (89%)	584 (83%)	
3	8 (0.1%)	8 (0.1%)	0 (0%)	
4	672 (11%)	562 (10%)	110 (16%)	
5	17 (0.3%)	11 (0.2%)	6 (0.9%)	
**OHX26TC**				<0.001
2	5,477 (90%)	4,889 (90%)	588 (84%)	
3	13 (0.2%)	11 (0.2%)	2 (0.3%)	
4	590 (9.7%)	486 (9.0%)	104 (15%)	
5	24 (0.4%)	18 (0.3%)	6 (0.9%)	
**OHX27TC**				<0.001
1	2 (<0.1%)	2 (<0.1%)	0 (0%)	
2	5,589 (92%)	4,977 (92%)	612 (87%)	
3	14 (0.2%)	14 (0.3%)	0 (0%)	
4	464 (7.6%)	382 (7.1%)	82 (12%)	
5	35 (0.6%)	29 (0.5%)	6 (0.9%)	
**OHX28TC**				0.033
1	1 (<0.1%)	1 (<0.1%)	0 (0%)	
2	5,113 (84%)	4,551 (84%)	562 (80%)	
3	13 (0.2%)	12 (0.2%)	1 (0.1%)	
4	923 (15%)	797 (15%)	126 (18%)	
5	54 (0.9%)	43 (0.8%)	11 (1.6%)	
**OHX29TC**				<0.001
1	13 (0.2%)	12 (0.2%)	1 (0.1%)	
2	4,959 (81%)	4,450 (82%)	509 (73%)	
3	14 (0.2%)	12 (0.2%)	2 (0.3%)	
4	1,047 (17%)	872 (16%)	175 (25%)	
5	71 (1.2%)	58 (1.1%)	13 (1.9%)	
**OHX30TC**				<0.001

Characteristic	Overall<br>N = 6,164[1]	Control<br>N = 5,458[1]	Case<br>N = 706[1]	p-value[2]
2	4,003 (66%)	3,631 (67%)	372 (53%)	
3	41 (0.7%)	37 (0.7%)	4 (0.6%)	
4	1,969 (32%)	1,659 (31%)	310 (44%)	
5	91 (1.5%)	77 (1.4%)	14 (2.0%)	
**OHX31TC**				<0.001
2	4,224 (69%)	3,835 (71%)	389 (56%)	
3	20 (0.3%)	15 (0.3%)	5 (0.7%)	
4	1,760 (29%)	1,472 (27%)	288 (41%)	
5	100 (1.6%)	82 (1.5%)	18 (2.6%)	
**OHX32TC**				0.3
2	1,655 (27%)	1,482 (27%)	173 (25%)	
4	4,384 (72%)	3,866 (72%)	518 (74%)	
5	65 (1.1%)	56 (1.0%)	9 (1.3%)	
**OHXRCAR**				0.019
1	761 (13%)	657 (13%)	104 (16%)	
2	4,963 (87%)	4,428 (87%)	535 (84%)	
**OHXRCARO**				0.5
1	1,203 (21%)	1,062 (21%)	141 (22%)	
2	4,519 (79%)	4,021 (79%)	498 (78%)	
**OHXRRES**				0.6
1	272 (4.7%)	244 (4.8%)	28 (4.4%)	
2	5,455 (95%)	4,843 (95%)	612 (96%)	
**OHXRRESO**				0.14
1	242 (4.2%)	222 (4.4%)	20 (3.1%)	
2	5,485 (96%)	4,865 (96%)	620 (97%)	
**OHDRCSTS**				0.7
1	6,104 (99%)	5,404 (99%)	700 (99%)	
3	60 (1.0%)	54 (1.0%)	6 (0.8%)	
**OHAREC**				0.002

Characteristic	Overall N = 6,164[1]	Control N = 5,458[1]	Case N = 706[1]	p-value[2]
1	3 (<0.1%)	2 (<0.1%)	1 (0.1%)	
2	312 (5.1%)	262 (4.8%)	50 (7.1%)	
3	2,458 (40%)	2,153 (40%)	305 (44%)	
4	3,331 (55%)	2,987 (55%)	344 (49%)	
**OHAPOS**				<0.001
1	5,899 (97%)	5,288 (98%)	611 (87%)	
2	205 (3.4%)	116 (2.1%)	89 (13%)	
**PAQ605**				0.2
1	1,649 (27%)	1,474 (27%)	175 (25%)	
2	4,511 (73%)	3,980 (73%)	531 (75%)	
**PAQ620**				0.4
1	2,845 (46%)	2,531 (46%)	314 (45%)	
2	3,316 (54%)	2,925 (54%)	391 (55%)	
**PAQ635**				0.037
1	1,423 (23%)	1,282 (23%)	141 (20%)	
2	4,740 (77%)	4,175 (77%)	565 (80%)	
**PAQ650**				<0.001
1	1,558 (25%)	1,452 (27%)	106 (15%)	
2	4,606 (75%)	4,006 (73%)	600 (85%)	
**PAQ665**				<0.001
1	2,565 (42%)	2,329 (43%)	236 (33%)	
2	3,597 (58%)	3,127 (57%)	470 (67%)	
**LBDBCDLC**				0.2
0	5,741 (93%)	5,092 (93%)	649 (92%)	
1	416 (6.8%)	361 (6.6%)	55 (7.8%)	
**LBDTHGLC**				0.6
0	4,923 (80%)	4,366 (80%)	557 (79%)	
1	1,234 (20%)	1,087 (20%)	147 (21%)	
**PUQ100**				0.5

Characteristic	Overall<br>N = 6,164[1]	Control<br>N = 5,458[1]	Case<br>N = 706[1]	p-value[2]
1	567 (9.3%)	498 (9.2%)	69 (9.9%)	
2	5,526 (91%)	4,900 (91%)	626 (90%)	
**PUQ110**				0.5
1	356 (5.9%)	311 (5.8%)	45 (6.5%)	
2	5,679 (94%)	5,035 (94%)	644 (93%)	
**SLQ030**				<0.001
0	1,459 (25%)	1,323 (26%)	136 (21%)	
1	1,477 (26%)	1,344 (26%)	133 (21%)	
2	1,158 (20%)	1,045 (20%)	113 (17%)	
3	1,660 (29%)	1,395 (27%)	265 (41%)	
**SLQ040**				<0.001
0	4,356 (75%)	3,937 (76%)	419 (64%)	
1	767 (13%)	665 (13%)	102 (16%)	
2	378 (6.5%)	318 (6.1%)	60 (9.2%)	
3	336 (5.8%)	262 (5.1%)	74 (11%)	
**SLQ050**				<0.001
1	1,798 (29%)	1,525 (28%)	273 (39%)	
2	4,365 (71%)	3,932 (72%)	433 (61%)	
**SLQ120**				0.016
0	974 (16%)	887 (16%)	87 (12%)	
1	1,500 (24%)	1,339 (25%)	161 (23%)	
2	2,095 (34%)	1,844 (34%)	251 (36%)	
3	1,087 (18%)	951 (17%)	136 (19%)	
4	502 (8.2%)	431 (7.9%)	71 (10%)	
**SMQ020**				<0.001
1	2,567 (42%)	2,230 (41%)	337 (48%)	
2	3,595 (58%)	3,226 (59%)	369 (52%)	
**SMD460**				0.6
0	4,013 (69%)	3,549 (69%)	464 (68%)	

Characteristic	Overall<br>N = 6,164[1]	Control<br>N = 5,458[1]	Case<br>N = 706[1]	p-value[2]
1	1,188 (20%)	1,043 (20%)	145 (21%)	
2	650 (11%)	581 (11%)	69 (10%)	
**SMQ681**				0.3
1	1,343 (22%)	1,199 (22%)	144 (21%)	
2	4,720 (78%)	4,170 (78%)	550 (79%)	
**SMQ851**				0.032
1	119 (2.0%)	98 (1.8%)	21 (3.0%)	
2	5,943 (98%)	5,270 (98%)	673 (97%)	
**SMQ863**				0.8
1	24 (0.4%)	21 (0.4%)	3 (0.4%)	
2	6,038 (100%)	5,347 (100%)	691 (100%)	
**SMDANY**				0.9
1	1,445 (24%)	1,281 (24%)	164 (24%)	
2	4,617 (76%)	4,087 (76%)	530 (76%)	
**SMQ856**				<0.001
1	3,442 (56%)	3,107 (57%)	335 (48%)	
2	2,721 (44%)	2,351 (43%)	370 (52%)	
**SMQ860**				0.9
1	3,616 (59%)	3,200 (59%)	416 (59%)	
2	2,547 (41%)	2,257 (41%)	290 (41%)	
**SMQ866**				0.3
1	875 (14%)	783 (14%)	92 (13%)	
2	5,289 (86%)	4,675 (86%)	614 (87%)	
**SMQ870**				0.4
1	5,836 (95%)	5,172 (95%)	664 (94%)	
2	328 (5.3%)	286 (5.2%)	42 (5.9%)	
**SMQ872**				0.6
1	950 (16%)	837 (16%)	113 (17%)	
2	4,886 (84%)	4,335 (84%)	551 (83%)	

Characteristic	Overall<br>N = 6,164[1]	Control<br>N = 5,458[1]	Case<br>N = 706[1]	p-value[2]
**SMQ874**				0.033
1	2,868 (47%)	2,566 (47%)	302 (43%)	
2	3,295 (53%)	2,891 (53%)	404 (57%)	
**SMQ878**				0.5
1	3,722 (60%)	3,304 (61%)	418 (59%)	
2	2,440 (40%)	2,153 (39%)	287 (41%)	
**SMQ940**				0.4
1	882 (14%)	788 (14%)	94 (13%)	
2	5,267 (86%)	4,659 (86%)	608 (87%)	
**WHQ030**				<0.001
1	3,306 (54%)	2,791 (51%)	515 (73%)	
2	320 (5.2%)	290 (5.3%)	30 (4.3%)	
3	2,529 (41%)	2,371 (43%)	158 (22%)	
**WHQ040**				<0.001
1	525 (8.5%)	493 (9.0%)	32 (4.6%)	
2	3,951 (64%)	3,399 (62%)	552 (79%)	
3	1,685 (27%)	1,566 (29%)	119 (17%)	
**WHQ070**				<0.001
1	2,182 (41%)	1,893 (40%)	289 (49%)	
2	3,123 (59%)	2,820 (60%)	303 (51%)	
**WHQ225**				<0.001
1	1,767 (29%)	1,555 (29%)	212 (30%)	
2	939 (15%)	797 (15%)	142 (20%)	
3	340 (5.5%)	279 (5.1%)	61 (8.7%)	
4	311 (5.1%)	252 (4.6%)	59 (8.4%)	
5	2,787 (45%)	2,557 (47%)	230 (33%)	
**OHQ870**				
0	1,612 (31%)	1,373 (31%)	239 (36%)	
1	319 (6.2%)	279 (6.2%)	40 (6.1%)	

Characteristic	Overall<br>N = 6,164[1]	Control<br>N = 5,458[1]	Case<br>N = 706[1]	p-value[2]
2	460 (9.0%)	401 (9.0%)	59 (9.0%)	
3	415 (8.1%)	368 (8.2%)	47 (7.2%)	
4	281 (5.5%)	245 (5.5%)	36 (5.5%)	
5	204 (4.0%)	183 (4.1%)	21 (3.2%)	
6	67 (1.3%)	59 (1.3%)	8 (1.2%)	
7	1,774 (35%)	1,568 (35%)	206 (31%)	
9	2 (<0.1%)	2 (<0.1%)	0 (0%)	
**OHQ030**				<0.001
1	2,373 (39%)	2,152 (40%)	221 (31%)	
2	917 (15%)	824 (15%)	93 (13%)	
3	872 (14%)	772 (14%)	100 (14%)	
4	484 (7.9%)	416 (7.6%)	68 (9.7%)	
5	510 (8.3%)	443 (8.1%)	67 (9.5%)	
6	901 (15%)	763 (14%)	138 (20%)	
7	91 (1.5%)	76 (1.4%)	15 (2.1%)	
**HUQ051**				<0.001
0	998 (16%)	916 (17%)	82 (12%)	
1	1,126 (18%)	1,024 (19%)	102 (14%)	
2	1,844 (30%)	1,651 (30%)	193 (27%)	
3	960 (16%)	811 (15%)	149 (21%)	
4	420 (6.8%)	367 (6.7%)	53 (7.5%)	
5	192 (3.1%)	162 (3.0%)	30 (4.3%)	
6	304 (4.9%)	259 (4.8%)	45 (6.4%)	
7	91 (1.5%)	73 (1.3%)	18 (2.6%)	
8	217 (3.5%)	184 (3.4%)	33 (4.7%)	
**DR1HELP**				0.2
1	25 (0.4%)	22 (0.4%)	3 (0.4%)	
4	2 (<0.1%)	2 (<0.1%)	0 (0%)	
5	15 (0.2%)	11 (0.2%)	4 (0.6%)	

Characteristic	Overall<br>N = 6,164[1]	Control<br>N = 5,458[1]	Case<br>N = 706[1]	p-value[2]
6	28 (0.5%)	24 (0.4%)	4 (0.6%)	
7	1 (<0.1%)	0 (0%)	1 (0.1%)	
8	0 (0%)	0 (0%)	0 (0%)	
9	45 (0.7%)	42 (0.8%)	3 (0.4%)	
10	0 (0%)	0 (0%)	0 (0%)	
11	9 (0.1%)	8 (0.1%)	1 (0.1%)	
12	6,038 (98%)	5,348 (98%)	690 (98%)	
**DR1MRESP**				0.5
1	6,125 (99%)	5,424 (99%)	701 (99%)	
2	2 (<0.1%)	2 (<0.1%)	0 (0%)	
3	1 (<0.1%)	1 (<0.1%)	0 (0%)	
5	10 (0.2%)	8 (0.1%)	2 (0.3%)	
6	12 (0.2%)	12 (0.2%)	0 (0%)	
9	1 (<0.1%)	1 (<0.1%)	0 (0%)	
11	10 (0.2%)	8 (0.1%)	2 (0.3%)	
**DR1DAY**				0.3
1	1,072 (17%)	934 (17%)	138 (20%)	
2	479 (7.8%)	422 (7.7%)	57 (8.1%)	
3	561 (9.1%)	495 (9.1%)	66 (9.3%)	
4	495 (8.0%)	441 (8.1%)	54 (7.6%)	
5	665 (11%)	591 (11%)	74 (10%)	
6	1,540 (25%)	1,353 (25%)	187 (26%)	
7	1,352 (22%)	1,222 (22%)	130 (18%)	
**ALQ121**				0.001
0	1,143 (21%)	981 (20%)	162 (25%)	
1	170 (3.1%)	145 (3.0%)	25 (3.9%)	
2	210 (3.8%)	182 (3.7%)	28 (4.4%)	
3	410 (7.4%)	373 (7.6%)	37 (5.8%)	
4	443 (8.0%)	404 (8.3%)	39 (6.1%)	

Characteristic	Overall<br>N = 6,164[1]	Control<br>N = 5,458[1]	Case<br>N = 706[1]	p-value[2]
5	414 (7.5%)	376 (7.7%)	38 (5.9%)	
6	779 (14%)	713 (15%)	66 (10%)	
7	431 (7.8%)	383 (7.9%)	48 (7.5%)	
8	343 (6.2%)	300 (6.2%)	43 (6.7%)	
9	594 (11%)	518 (11%)	76 (12%)	
10	582 (11%)	501 (10%)	81 (13%)	
**AUQ054**				
1	2,183 (35%)	1,982 (36%)	201 (29%)	
2	2,447 (40%)	2,169 (40%)	278 (39%)	
3	933 (15%)	800 (15%)	133 (19%)	
4	437 (7.1%)	369 (6.8%)	68 (9.6%)	
5	150 (2.4%)	127 (2.3%)	23 (3.3%)	
6	11 (0.2%)	9 (0.2%)	2 (0.3%)	
**RIDAGEYR**	49.59 (17.46)	48.85 (17.57)	55.35 (15.49)	<0.001
**WTINTPRP**	27,412.09 (31,571.48)	27,675.56 (31,738.70)	25,375.21 (30,191.63)	<0.001
**WTMECPRP**	29,548.77 (34,196.15)	29,862.47 (34,445.25)	27,123.64 (32,125.27)	<0.001
**SDMVSTRA**	160.39 (6.89)	160.41 (6.90)	160.20 (6.82)	0.5
**INDFMPIR**	2.63 (1.64)	2.64 (1.65)	2.55 (1.57)	0.4
**URXUMA**	46.24 (266.57)	40.16 (251.78)	93.50 (358.26)	<0.001
**URXUMS**	46.24 (266.57)	40.16 (251.78)	93.50 (358.26)	<0.001
**URXUCR**	132.23 (87.44)	131.31 (87.58)	139.39 (86.10)	0.006
**URXCRS**	11,652.65 (7,702.16)	11,571.66 (7,713.35)	12,282.91 (7,590.46)	0.006
**URDACT**	45.68 (307.78)	40.88 (309.99)	83.01 (287.44)	<0.001
**LBXSATSI**	22.39 (17.69)	21.19 (15.38)	31.68 (28.42)	<0.001
**LBXSAL**	4.07 (0.34)	4.08 (0.33)	3.96 (0.36)	<0.001
**LBDSALSI**	40.70 (3.36)	40.83 (3.30)	39.64 (3.63)	<0.001
**LBXSAPSI**	77.73 (26.25)	76.55 (23.89)	86.93 (38.98)	<0.001
**LBXSASSI**	21.79 (13.10)	20.87 (9.82)	28.95 (26.37)	<0.001
**LBXSC3SI**	25.40 (2.40)	25.42 (2.37)	25.28 (2.62)	0.3

Characteristic	Overall<br>N = 6,164[1]	Control<br>N = 5,458[1]	Case<br>N = 706[1]	p-value[2]
**LBXSBU**	14.84 (5.66)	14.64 (5.35)	16.39 (7.49)	<0.001
**LBDSBUSI**	5.30 (2.02)	5.23 (1.91)	5.85 (2.67)	<0.001
**LBXSCLSI**	101.61 (2.91)	101.68 (2.81)	101.09 (3.52)	<0.001
**LBXSCK**	174.98 (330.09)	176.73 (346.14)	161.44 (157.20)	0.7
**LBXSCR**	0.90 (0.38)	0.89 (0.36)	0.96 (0.54)	<0.001
**LBDSCRSI**	79.13 (33.85)	78.36 (31.50)	85.13 (47.93)	<0.001
**LBXSGB**	3.09 (0.43)	3.07 (0.42)	3.23 (0.52)	<0.001
**LBDSGBSI**	30.89 (4.34)	30.72 (4.18)	32.25 (5.21)	<0.001
**LBXSGL**	102.26 (36.85)	99.95 (33.57)	120.02 (52.71)	<0.001
**LBDSGLSI**	5.66 (2.01)	5.53 (1.83)	6.62 (2.89)	<0.001
**LBXSGTSI**	32.15 (45.42)	28.79 (35.02)	58.23 (88.22)	<0.001
**LBXSIR**	86.56 (35.98)	86.94 (35.90)	83.57 (36.47)	0.003
**LBDSIRSI**	15.50 (6.37)	15.57 (6.36)	14.97 (6.44)	0.003
**LBXSLDSI**	158.09 (33.71)	156.76 (31.96)	168.29 (43.69)	<0.001
**LBXSOSSI**	281.38 (5.47)	281.24 (5.30)	282.46 (6.49)	<0.001
**LBXSPH**	3.56 (0.52)	3.57 (0.52)	3.53 (0.53)	0.089
**LBDSPHSI**	1.15 (0.17)	1.15 (0.17)	1.14 (0.17)	0.089
**LBXSKSI**	4.09 (0.36)	4.08 (0.36)	4.13 (0.40)	0.005
**LBXSNASI**	140.55 (2.60)	140.58 (2.55)	140.31 (2.99)	0.082
**LBXSTB**	0.46 (0.28)	0.45 (0.27)	0.49 (0.29)	<0.001
**LBDSTBSI**	7.84 (4.72)	7.77 (4.67)	8.36 (5.03)	<0.001
**LBXSCA**	9.27 (0.37)	9.28 (0.37)	9.25 (0.41)	0.2
**LBDSCASI**	2.32 (0.09)	2.32 (0.09)	2.31 (0.10)	0.2
**LBXSCH**	186.28 (40.66)	187.20 (40.17)	179.17 (43.67)	<0.001
**LBDSCHSI**	4.82 (1.05)	4.84 (1.04)	4.63 (1.13)	<0.001
**LBXSTP**	7.16 (0.44)	7.16 (0.44)	7.19 (0.49)	0.2
**LBDSTPSI**	71.37 (4.38)	71.33 (4.31)	71.68 (4.90)	0.2
**LBXSTR**	139.78 (110.41)	136.31 (105.91)	166.66 (137.61)	<0.001
**LBDSTRSI**	1.57 (1.24)	1.53 (1.19)	1.87 (1.54)	<0.001

Characteristic	Overall<br>N = 6,164[1]	Control<br>N = 5,458[1]	Case<br>N = 706[1]	p-value[2]
**LBXSUA**	5.41 (1.47)	5.33 (1.43)	6.00 (1.62)	<0.001
**LBDSUASI**	321.79 (87.28)	317.22 (84.96)	357.10 (96.49)	<0.001
**BMXWT**	84.00 (22.86)	81.57 (20.59)	102.85 (29.80)	<0.001
**BMXHT**	167.02 (9.91)	166.84 (9.89)	168.35 (9.92)	<0.001
**BMXBMI**	30.02 (7.43)	29.22 (6.63)	36.21 (9.96)	<0.001
**BMXLEG**	39.24 (3.78)	39.29 (3.74)	38.84 (4.03)	0.009
**BMXARML**	37.68 (2.84)	37.58 (2.81)	38.45 (2.93)	<0.001
**BMXARMC**	33.79 (5.34)	33.33 (5.00)	37.39 (6.42)	<0.001
**BMXWAIST**	100.96 (17.18)	99.02 (15.86)	116.26 (19.37)	<0.001
**BMXHIP**	107.60 (14.73)	106.19 (13.36)	118.60 (19.53)	<0.001
**BPXOSY1**	124.59 (19.11)	124.01 (18.99)	129.00 (19.49)	<0.001
**BPXODI1**	74.99 (11.76)	74.70 (11.55)	77.23 (13.10)	<0.001
**BPXOSY2**	124.36 (18.96)	123.81 (18.88)	128.58 (19.08)	<0.001
**BPXODI2**	74.42 (11.75)	74.14 (11.54)	76.55 (13.10)	<0.001
**BPXOSY3**	124.39 (18.90)	123.87 (18.85)	128.42 (18.82)	<0.001
**BPXODI3**	74.14 (11.81)	73.87 (11.60)	76.22 (13.15)	<0.001
**BPXOPLS1**	69.25 (11.69)	68.90 (11.32)	72.02 (14.01)	<0.001
**BPXOPLS2**	69.89 (11.74)	69.56 (11.37)	72.46 (14.02)	<0.001
**BPXOPLS3**	70.26 (11.80)	69.95 (11.43)	72.68 (14.18)	<0.001
**LBXWBCSI**	7.22 (5.45)	7.17 (5.73)	7.60 (2.31)	<0.001
**LBXLYPCT**	31.48 (8.91)	31.63 (8.87)	30.30 (9.21)	<0.001
**LBXMOPCT**	8.19 (2.24)	8.16 (2.22)	8.43 (2.36)	0.026
**LBXNEPCT**	56.86 (9.64)	56.77 (9.60)	57.59 (9.92)	0.008
**LBXEOPCT**	2.78 (2.09)	2.76 (2.06)	2.98 (2.27)	0.001
**LBXBAPCT**	0.80 (0.33)	0.80 (0.33)	0.82 (0.34)	0.13
**LBDLYMNO**	2.25 (4.62)	2.25 (4.90)	2.25 (0.85)	0.073
**LBDMONO**	0.57 (0.21)	0.57 (0.21)	0.62 (0.22)	<0.001
**LBDNENO**	4.15 (1.71)	4.11 (1.70)	4.44 (1.73)	<0.001
**LBDEONO**	0.20 (0.17)	0.19 (0.17)	0.22 (0.18)	<0.001

Characteristic	Overall<br>N = 6,164[1]	Control<br>N = 5,458[1]	Case<br>N = 706[1]	p-value[2]
**LBDBANO**				<0.001
0	2,899 (47%)	2,627 (48%)	272 (39%)	
0.1	3,211 (52%)	2,786 (51%)	425 (60%)	
0.2	46 (0.7%)	38 (0.7%)	8 (1.1%)	
0.3	1 (<0.1%)	1 (<0.1%)	0 (0%)	
0.4	1 (<0.1%)	1 (<0.1%)	0 (0%)	
**LBXRBCSI**	4.74 (0.50)	4.73 (0.49)	4.79 (0.54)	<0.001
**LBXHGB**	14.03 (1.55)	14.02 (1.53)	14.09 (1.69)	0.3
**LBXHCT**	41.75 (4.19)	41.71 (4.14)	42.02 (4.53)	0.2
**LBXMCVSI**	88.41 (6.14)	88.46 (6.08)	88.06 (6.57)	0.028
**LBXMC**	33.55 (0.95)	33.56 (0.94)	33.49 (1.01)	0.032
**LBXMCHSI**	29.69 (2.49)	29.71 (2.47)	29.52 (2.66)	0.013
**LBXRDW**	13.88 (1.36)	13.84 (1.32)	14.22 (1.58)	<0.001
**LBXPLTSI**	246.49 (65.32)	247.87 (63.94)	235.78 (74.29)	<0.001
**LBXMPSI**	8.26 (0.90)	8.25 (0.90)	8.38 (0.91)	<0.001
**LBXNRBC**	0.08 (0.08)	0.08 (0.08)	0.08 (0.08)	0.2
**WTDRD1PP**	31,744.16 (40,506.30)	32,309.08 (41,350.06)	27,376.83 (32,968.34)	<0.001
**LBXCOT**	57.54 (130.65)	57.27 (128.66)	59.62 (145.25)	>0.9
**LBXHCOT**	22.97 (62.51)	22.42 (58.39)	27.28 (88.03)	0.3
**DBD895**	4.30 (70.88)	4.40 (75.31)	3.52 (3.94)	0.5
**DBD905**	2.09 (5.22)	2.11 (5.34)	1.93 (4.16)	0.5
**DBD910**	3.44 (85.16)	3.57 (90.47)	2.41 (6.20)	>0.9
**WTDR2DPP**	31,762.27 (48,490.31)	32,387.04 (49,423.02)	26,932.26 (40,266.24)	0.016
**DR1EXMER**	72.74 (21.38)	72.74 (21.28)	72.76 (22.11)	0.11
**DR1DBIH**	11.79 (12.61)	11.86 (12.77)	11.20 (11.25)	0.6
**DR1TNUMF**	15.42 (6.00)	15.45 (6.00)	15.18 (5.95)	0.14
**DR1TKCAL**	2,133.91 (1,018.62)	2,128.58 (1,004.95)	2,175.14 (1,118.67)	0.5
**DR1TPROT**	79.72 (42.83)	79.54 (42.90)	81.16 (42.27)	0.2
**DR1TCARB**	246.16 (126.42)	245.65 (124.33)	250.06 (141.57)	0.8

Characteristic	Overall<br>N = 6,164[1]	Control<br>N = 5,458[1]	Case<br>N = 706[1]	p-value[2]
**DR1TSUGR**	106.14 (75.88)	105.53 (74.80)	110.82 (83.65)	0.3
**DR1TFIBE**	16.36 (10.63)	16.44 (10.64)	15.73 (10.51)	0.045
**DR1TTFAT**	86.98 (49.74)	86.70 (49.68)	89.14 (50.22)	0.10
**DR1TSFAT**	27.80 (17.57)	27.63 (17.38)	29.14 (18.93)	0.036
**DR1TMFAT**	29.81 (17.81)	29.71 (17.82)	30.58 (17.75)	0.075
**DR1TPFAT**	20.64 (14.26)	20.67 (14.41)	20.42 (12.97)	0.8
**DR1TCHOL**	318.87 (259.02)	315.51 (256.23)	344.72 (278.38)	0.007
**DR1TATOC**	9.09 (6.84)	9.11 (6.87)	8.95 (6.58)	0.7
**DR1TATOA**	0.74 (3.30)	0.74 (3.23)	0.73 (3.75)	0.2
**DR1TRET**	383.51 (502.72)	380.42 (472.26)	407.30 (693.96)	0.6
**DR1TVARA**	589.54 (652.69)	588.31 (634.06)	599.02 (781.94)	0.9
**DR1TACAR**	349.00 (964.49)	356.37 (994.10)	292.05 (692.19)	0.8
**DR1TBCAR**	2,257.16 (4,418.97)	2,276.15 (4,472.12)	2,110.47 (3,984.47)	0.8
**DR1TCRYP**	90.62 (243.84)	88.35 (224.98)	108.17 (356.96)	0.088
**DR1TLYCO**	4,622.12 (8,866.78)	4,660.34 (8,988.30)	4,326.36 (7,862.75)	0.4
**DR1TLZ**	1,531.69 (3,225.51)	1,558.83 (3,339.81)	1,321.55 (2,133.17)	0.3
**DR1TVB1**	1.54 (0.91)	1.54 (0.92)	1.55 (0.88)	0.5
**DR1TVB2**	1.94 (1.26)	1.94 (1.25)	1.95 (1.32)	>0.9
**DR1TNIAC**	25.02 (16.44)	24.98 (16.44)	25.33 (16.49)	0.6
**DR1TVB6**	2.03 (1.88)	2.03 (1.88)	2.07 (1.91)	0.6
**DR1TFOLA**	360.84 (229.67)	361.38 (231.26)	356.69 (217.10)	0.7
**DR1TFA**	152.50 (158.80)	152.24 (160.91)	154.50 (141.61)	0.3
**DR1TFF**	209.82 (144.12)	210.75 (144.94)	202.64 (137.52)	0.3
**DR1TFDFE**	466.78 (324.10)	467.32 (326.96)	462.55 (301.14)	0.8
**DR1TCHL**	335.88 (206.28)	334.09 (205.43)	349.74 (212.40)	0.045
**DR1TVB12**	4.59 (5.95)	4.51 (5.65)	5.16 (7.86)	0.004
**DR1TB12A**	0.80 (2.80)	0.78 (2.72)	0.93 (3.35)	0.8
**DR1TVC**	78.26 (96.05)	78.75 (97.62)	74.42 (82.94)	0.4
**DR1TVD**	4.32 (5.48)	4.32 (5.55)	4.38 (4.90)	0.043

Characteristic	Overall N = 6,164[1]	Control N = 5,458[1]	Case N = 706[1]	p-value[2]
**DR1TVK**	122.80 (175.40)	124.29 (178.53)	111.30 (148.62)	0.3
**DR1TCALC**	906.66 (572.66)	905.11 (569.93)	918.65 (593.56)	0.7
**DR1TPHOS**	1,337.90 (698.79)	1,335.82 (698.61)	1,353.96 (700.42)	0.5
**DR1TMAGN**	293.83 (152.64)	295.06 (153.26)	284.40 (147.47)	0.087
**DR1TIRON**	13.51 (8.19)	13.48 (8.19)	13.73 (8.21)	0.4
**DR1TZINC**	10.45 (6.82)	10.41 (6.84)	10.72 (6.62)	0.13
**DR1TCOPP**	1.18 (1.03)	1.18 (0.99)	1.18 (1.29)	0.4
**DR1TSODI**	3,443.63 (1,865.45)	3,441.03 (1,871.52)	3,463.70 (1,819.01)	0.5
**DR1TPOTA**	2,527.36 (1,281.03)	2,535.37 (1,290.31)	2,465.56 (1,206.03)	0.3
**DR1TSELE**	111.91 (65.04)	111.51 (65.04)	115.00 (65.01)	0.058
**DR1TCAFF**	142.05 (198.41)	141.26 (196.48)	148.11 (212.80)	0.057
**DR1TTHEO**	34.29 (72.88)	34.24 (73.31)	34.65 (69.53)	0.7
**DR1TALCO**	10.01 (31.35)	10.03 (28.22)	9.84 (49.31)	0.014
**DR1TMOIS**	2,888.88 (1,526.43)	2,872.49 (1,512.56)	3,015.62 (1,625.27)	0.041
**DR1TS040**	0.45 (0.45)	0.45 (0.44)	0.48 (0.52)	0.3
**DR1TS060**	0.30 (0.31)	0.30 (0.30)	0.32 (0.35)	0.4
**DR1TS080**	0.26 (0.31)	0.26 (0.31)	0.27 (0.31)	0.5
**DR1TS100**	0.52 (0.49)	0.52 (0.49)	0.54 (0.54)	0.4
**DR1TS120**	0.99 (1.62)	0.99 (1.64)	1.00 (1.51)	0.6
**DR1TS140**	2.29 (1.94)	2.28 (1.91)	2.40 (2.20)	0.2
**DR1TS160**	15.36 (9.34)	15.28 (9.27)	15.98 (9.82)	0.049
**DR1TS180**	6.63 (4.33)	6.57 (4.28)	7.09 (4.63)	0.002
**DR1TM161**	1.21 (0.95)	1.21 (0.96)	1.23 (0.85)	0.2
**DR1TM181**	28.04 (16.79)	27.94 (16.80)	28.76 (16.74)	0.076
**DR1TM201**	0.34 (0.30)	0.34 (0.30)	0.35 (0.27)	0.083
**DR1TM221**	0.03 (0.14)	0.04 (0.14)	0.03 (0.06)	0.8
**DR1TP182**	18.29 (12.71)	18.32 (12.85)	18.10 (11.59)	0.8
**DR1TP183**	1.92 (1.56)	1.92 (1.58)	1.88 (1.41)	>0.9
**DR1TP184**	0.01 (0.03)	0.01 (0.03)	0.01 (0.03)	0.14

Characteristic	Overall<br>N = 6,164[1]	Control<br>N = 5,458[1]	Case<br>N = 706[1]	p-value[2]
**DR1TP204**	0.15 (0.13)	0.15 (0.13)	0.16 (0.14)	0.028
**DR1TP205**	0.04 (0.14)	0.04 (0.15)	0.03 (0.12)	0.10
**DR1TP225**	0.03 (0.07)	0.03 (0.06)	0.03 (0.09)	0.004
**DR1TP226**	0.07 (0.24)	0.07 (0.22)	0.07 (0.36)	0.8
**DR1_320Z**	1,212.62 (1,224.99)	1,207.78 (1,216.31)	1,250.09 (1,290.36)	0.9
**DR1_330Z**	563.14 (1,035.13)	557.33 (1,023.15)	607.98 (1,123.20)	0.6
**DR1BWATZ**	649.49 (1,007.33)	650.44 (1,007.69)	642.11 (1,005.25)	0.2
**DR1TWSZ**				0.2
1	3,942 (66%)	3,493 (66%)	449 (65%)	
4	1,523 (25%)	1,353 (26%)	170 (24%)	
91	532 (8.9%)	457 (8.6%)	75 (11%)	
**DS1DSCNT**	1.06 (1.85)	1.06 (1.85)	1.12 (1.88)	0.13
**DSDCOUNT**	1.40 (2.05)	1.41 (2.07)	1.38 (1.89)	0.6
**PHAFSTHR**	9.06 (4.83)	9.06 (4.82)	9.09 (4.90)	0.9
**PHAFSTMN**	29.28 (17.33)	29.25 (17.36)	29.48 (17.07)	0.7
**LBXFER**	157.47 (181.13)	148.24 (151.05)	228.57 (322.74)	<0.001
**LBDFERSI**	157.47 (181.13)	148.24 (151.05)	228.57 (322.74)	<0.001
**LBXIRN**	85.83 (35.99)	86.20 (35.93)	82.91 (36.34)	0.003
**LBDIRNSI**	15.38 (6.38)	15.45 (6.37)	14.85 (6.45)	0.002
**LBXUIB**	238.50 (63.58)	238.19 (63.10)	240.95 (67.18)	0.064
**LBDUIBSI**	42.71 (11.40)	42.66 (11.31)	43.11 (12.08)	0.072
**LBDTIB**	324.34 (50.89)	324.43 (50.67)	323.63 (52.56)	>0.9
**LBDTIBSI**	58.09 (9.11)	58.11 (9.08)	57.96 (9.41)	>0.9
**LBDPCT**	27.11 (11.55)	27.22 (11.46)	26.25 (12.18)	0.001
**LBXGH**	5.84 (1.10)	5.76 (1.02)	6.46 (1.49)	<0.001
**LBDHDD**	53.11 (15.79)	53.76 (15.67)	48.17 (15.81)	<0.001
**LBDHDDSI**	1.38 (0.41)	1.40 (0.41)	1.25 (0.41)	<0.001
**LBXHSCRP**	4.08 (8.19)	3.81 (7.79)	6.21 (10.56)	<0.001
**LBXIHG**	0.22 (0.57)	0.23 (0.60)	0.20 (0.15)	0.003

Characteristic	Overall<br>N = 6,164[1]	Control<br>N = 5,458[1]	Case<br>N = 706[1]	p-value[2]
**LBDIHGSI**	1.11 (2.83)	1.13 (2.99)	0.98 (0.75)	0.003
**LBXBGE**	0.05 (0.01)	0.05 (0.01)	0.05 (0.00)	0.6
**LBDBGESI**	0.25 (0.07)	0.25 (0.07)	0.25 (0.01)	0.6
**LBXBGM**	1.12 (2.09)	1.15 (2.17)	0.90 (1.29)	0.048
**LBDBGMSI**	5.58 (10.43)	5.71 (10.83)	4.51 (6.45)	0.048
**LUANMVGP**	11.11 (2.32)	11.07 (2.13)	11.44 (3.46)	<0.001
**LUANMTGP**	14.54 (5.66)	14.13 (5.27)	17.74 (7.30)	<0.001
**LUXSIQR**	1.02 (2.39)	0.71 (1.00)	3.44 (5.97)	<0.001
**LUXSIQRM**	15.30 (21.23)	14.36 (19.75)	22.55 (29.35)	<0.001
**LUXCAPM**	266.04 (63.06)	259.96 (60.36)	313.08 (63.73)	<0.001
**LUXCPIQR**	37.01 (19.98)	37.34 (19.80)	34.49 (21.14)	<0.001
**PAD680**	333.44 (202.75)	329.52 (201.25)	363.65 (211.71)	<0.001
**LBXBPB**	1.17 (1.15)	1.16 (1.15)	1.24 (1.18)	0.2
**LBDBPBSI**	0.06 (0.06)	0.06 (0.06)	0.06 (0.06)	0.2
**LBXBCD**	0.46 (0.52)	0.46 (0.52)	0.45 (0.54)	0.2
**LBDBCDSI**	4.09 (4.66)	4.10 (4.64)	3.99 (4.84)	0.2
**LBXTHG**	1.32 (2.39)	1.35 (2.49)	1.07 (1.40)	0.016
**LBDTHGSI**	6.59 (11.93)	6.76 (12.42)	5.34 (6.99)	0.016
**LBXBSE**	186.44 (27.59)	186.76 (27.21)	183.95 (30.22)	0.004
**LBDBSESI**	2.37 (0.35)	2.37 (0.35)	2.34 (0.38)	0.004
**LBXBMN**	9.72 (3.58)	9.69 (3.50)	9.93 (4.18)	0.7
**LBDBMNSI**	176.85 (65.22)	176.34 (63.67)	180.78 (76.08)	0.7
**SLD012**	7.54 (1.64)	7.54 (1.63)	7.55 (1.69)	0.8
**SLD013**	8.23 (1.77)	8.23 (1.76)	8.23 (1.86)	0.7
**LBXTC**	185.95 (40.68)	186.85 (40.21)	179.03 (43.58)	<0.001
**LBDTCSI**	4.81 (1.05)	4.83 (1.04)	4.63 (1.13)	<0.001
**URXVOL1**	107.87 (80.09)	108.33 (80.55)	104.28 (76.33)	0.4
**URDFLOW1**	0.93 (1.26)	0.93 (1.10)	0.92 (2.15)	0.11
**URDTIME1**	149.79 (107.77)	149.70 (109.29)	150.46 (95.24)	0.071

Characteristic	Overall<br>N = 6,164[1]	Control<br>N = 5,458[1]	Case<br>N = 706[1]	p-value[2]
**WHD010**	66.43 (4.08)	66.35 (4.07)	67.04 (4.09)	<0.001
**WHD020**	183.68 (48.27)	178.57 (43.55)	223.28 (62.57)	<0.001
**WHD050**	184.93 (50.66)	179.83 (46.27)	224.55 (64.08)	<0.001
**WHD120**	156.18 (41.50)	153.66 (39.32)	174.15 (51.09)	<0.001
**WHD140**	202.60 (56.45)	196.90 (51.63)	246.65 (70.91)	<0.001
**WHQ150**	41.19 (16.42)	40.37 (16.31)	47.53 (15.83)	<0.001
**SLQ300**	16.13 (9.36)	16.09 (9.38)	16.43 (9.21)	0.3
**SLQ310**	6.56 (2.39)	6.56 (2.38)	6.55 (2.47)	0.9
**SLQ320**	13.18 (10.49)	13.11 (10.50)	13.74 (10.41)	0.2
**SLQ330**	7.65 (2.16)	7.67 (2.19)	7.47 (1.97)	0.042

[1]n (%); Mean (SD)

[2]Pearson's Chi-squared test; Fisher's exact test; Wilcoxon rank sum test
