# Supplementary material for: Integrative and interpretable machine learning framework for early non-invasive detection of clinically significant liver fibrosis
Source: Front Med (Lausanne). 2026 Jun 23;13:1736295. doi: 10.3389/fmed.2026.1736295 (PMC13337473; doi:10.3389/fmed.2026.1736295)
Supplement: Supplementary file 10 — We employed the Recursive Feature Elimination (RFE) method for feature screening. Using the Area Under the Curve (AUC) as the core indicator, we evaluated the predictive performance of seven machine learning models (Gbm-RFE, LightGBM-RFE, Ranger-RFE, Rpart-RFE, Svm-RFE, Xgboost-RFE, and CatBoost-RFE) with changes in the number of features. The chart intuitively presents the changing trends of AUC values for different models as the number of features decreases during the feature selection process, providing a basis for selecting the optimal feature combination and model. [file Data_Sheet_10.pdf]

	classif. catboost	classif. lightgbm	classif. xgboost	classif. rpart	classif. gbm	classif. ranger	classif. svm
Model	classif.catboost	classif.lightgbm	classif.xgboost	classif.rpart	classif.gbm	classif.ranger	classif.svm
ce（分类错误率）	c(classif.ce = 0.249290027254521)	c(classif.ce = 0.252137447109666)	c(classif.ce = 0.271951935673307)	c(classif.ce = 0.271244221589797)	c(classif.ce = 0.251409668445982)	c(classif.ce = 0.243643335087587)	c(classif.ce = 0.288246497757061)
best	c("ALQ111", "ALQ121", "ALQ151", "AUQ054", "BMDSTATS", "BMXARMC", "BMXARML", "BMXBMI", "BMXHT", "BMXLEG", "BMXWAIST", "BMXWT", "BPQ080", "CBQ596", "DBD895", "DBD905", "DBD910", "DBQ095Z", "DBQ197", "DBQ229", "DBQ700", "DBQ930", "DBQ935", "DBQ940", "DBQ945", "DIQ010", "DIQ160", "DIQ180", "DMDBORN4", "DMDEDUC2", "DMDMARTZ", "DPQ010", "DPQ020", "DPQ030", "DPQ040", "DPQ050", "DPQ060", "DPQ070", "DPQ080", "DPQ090", "DR1BWATZ", "DR1DAY", "DR1DBIH", "DR1EXMER", "DR1HELP", "DR1LANG", "DR1MRESP", "DR1STY", "DR1TACAR", "DR1TALCO", "DR1TATOA", "DR1TATOC", "DR1TB12A", "DR1TBCAR", "DR1TCAFF", "DR1TCALC", "DR1TCARB", "DR1TCHL", "DR1TCHOL", "DR1TCOPP", "DR1TCRYP", "DR1TFA", "DR1TFDFE", "DR1TFF", "DR1TFIBE", "DR1TFOLA", "DR1TIRON", "DR1TKCAL", "DR1TLYCO", "DR1TLZ", "DR1TM161", "DR1TM181", "DR1TM201", "DR1TM221", "DR1TMAGN", "DR1TMFAT", "DR1TMOIS", "DR1TNIAC", "DR1TNUMF", "DR1TP182", "DR1TP183", "DR1TP184", "DR1TP204", "DR1TP205", "DR1TP225", "DR1TP226", "DR1TPFAT", "DR1TPHOS", "DR1TPOTA", "DR1TPROT", "DR1TRET", "DR1TS040", "DR1TS060", "DR1TS080", "DR1TS100", "DR1TS120", "DR1TS140", "DR1TS160", "DR1TS180", "DR1TSELE", "DR1TSFAT", "DR1TSODI", "DR1TSUGR", "DR1TTFAT", "DR1TTHEO", "DR1TVARA", "DR1TVB1", "DR1TVB12", "DR1TVB2", "DR1TVB6", "DR1TVC", "DR1TVD", "DR1TVK", "DR1TWSZ", "DR1TZINC", "DR1_300", "DR1_320Z", "DR1_330Z", "DR2DRSTZ", "DRD340", "DRD360", "DRDINT", "DRQSDIET", "DRQSPREP", "DS1AN", "DS1ANCNT", "DS1DS", "DS1DSCNT", "DSD010", "DSD010AN", "DSDANCNT", "DSDCOUNT", "FIAINTRP", "FIALANG", "FIAPROXY", "FSD151", "FSD162", "FSDAD", "FSDHH", "FSQ165", "HEQ010", "HEQ030", "HIQ011", "HIQ210", "HIQ270", "HSQ590", "HUQ010", "HUQ030", "HUQ051", "HUQ071", "HUQ090", "IMQ011", "IMQ020", "INDFMMPC", "INDFMPIR", "KIQ005", "KIQ022", "KIQ026", "KIQ042", "KIQ044", "KIQ046", "KIQ480", "LBDBANO", "LBDBCDLC", "LBDBCDSI", "LBDBGELC", "LBDBGESI", "LBDBGMLC", "LBDBGMSI", "LBDBMNSI", "LBDBPBSI", "LBDBSESI", "LBDCOTLC", "LBDEONO", "LBDFERSI", "LBDHCI", "LBDHCOLC", "LBDHDD", "LBDHDDSI", "LBDHEG", "LBDHEM", "LBDHRPLC", "LBDIHGLC", "LBDIHGSI", "LBDIRNSI", "LBDLYMNO", "LBDMONO", "LBDNENO", "LBDPCT", "LBDSALSI", "LBDSBUSI", "LBDSCASI", "LBDSCHSI", "LBDSCRSI", "LBDSGBSI", "LBDSGLSI", "LBDSGTLC", "LBDSIRSI", "LBDSPHSI", "LBDSTBLC", "LBDSTBSI", "LBDSTPSI", "LBDSTRSI", "LBDSUASI", "LBDTCSI", "LBDTHGLC", "LBDTHGSI", "LBDTIB", "LBDTIBSI", "LBXBAPCT", "LBXBCD", "LBXBGE", "LBXBGM", "LBXBMN", "LBXBPB", "LBXBSE", "LBXCOT", "LBXEOPCT", "LBXFER", "LBXGH", "LBXHA", "LBXHBC", "LBXHBS", "LBXHCR", "LBXHCT", "LBXHGB", "LBXHSCRP", "LBXIHG", "LBXIRN", "LBXLYPCT", "LBXMC", "LBXMCHSI", "LBXMCVSI", "LBXMOPCT", "LBXMPSI", "LBXNEPCT", "LBXNRBC", "LBXPLTSI", "LBXRBCSI", "LBXRDW", "LBXSAL", "LBXSAPSI", "LBXSASSI", "LBXSATSI", "LBXSBU", "LBXSC3SI", "LBXSCA", "LBXSCH", "LBXSCK", "LBXSCLSI", "LBXSCR", "LBXSGB", "LBXSGL", "LBXSGTSI", "LBXSIR", "LBXSKSI", "LBXSLDSI", "LBXSNASI", "LBXSOSSI", "LBXSPH", "LBXSTB", "LBXSTP", "LBXSTR", "LBXSUA", "LBXTC", "LBXTHG", "LBXWBCSI", "MCQ010", "MCQ053", "MCQ080", "MCQ092", "MCQ160A", "MCQ160B", "MCQ160C", "MCQ160D", "MCQ160E", "MCQ160F", "MCQ160L", "MCQ160M", "MCQ160P", "MCQ220", "MCQ300A", "MCQ300B", "MCQ300C", "MCQ366A", "MCQ366B", "MCQ366C", "MCQ366D", "MCQ371A", "MCQ371B", "MCQ371C", "MCQ371D", "MCQ520", "MCQ550", "MCQ560", "MIAINTRP", "MIALANG", "MIAPROXY", "OCD150", "OHAPOS", "OHAREC", "OHDDESTS", "OHDEXSTS", "OHDRCSTS", "OHQ030", "OHQ033", "OHQ620", "OHQ640", "OHQ770", "OHQ835", "OHQ845", "OHQ850", "OHQ860", "OHQ870", "OHX01TC", "OHX02TC", "OHX03TC", "OHX04TC", "OHX05TC", "OHX06TC", "OHX07TC", "OHX08TC", "OHX09TC", "OHX10TC", "OHX11TC", "OHX12TC", "OHX13TC", "OHX14TC", "OHX15TC", "OHX16TC", "OHX17TC", "OHX18TC", "OHX19TC", "OHX20TC", "OHX21TC", "OHX22TC", "OHX23TC", "OHX24TC", "OHX25TC", "OHX26TC", "OHX27TC", "OHX28TC", "OHX29TC", "OHX30TC", "OHX31TC", "OHX32TC", "OHXIMP", "OHXRCAR", "OHXRCARO", "OHXRRES", "OHXRRESO", "PAD680", "PAQ605", "PAQ620", "PAQ635", "PAQ650", "PAQ665", "PHAFSTHR", "PHAFSTMN", "PHDSESN", "PHQ020", "PHQ030", "PHQ040", "PHQ050", "PHQ060", "PUQ100", "PUQ110", "RIAGENDR", "RIDAGEYR", "RIDEXMON", "RIDRETH1", "RIDRETH3", "SDMVPSU", "SDMVSTRA", "SIAINTRP", "SIALANG", "SIAPROXY", "SLD012", "SLD013", "SLQ030", "SLQ040", "SLQ050", "SLQ120", "SLQ300", "SLQ310", "SLQ320", "SLQ330", "SMD460", "SMDANY", "SMQ020", "SMQ681", "SMQ851", "SMQ856", "SMQ860", "SMQ863", "SMQ866", "SMQ870", "SMQ872", "SMQ874", "SMQ878", "URDACT", "URDFLOW1", "URDTIME1", "URDUMALC", "URXCRS", "URXUCR", "URXUMA", "URXUMS", "URXVOL1", "WHD010", "WHD020", "WHD050", "WHD120", "WHD140", "WHQ030", "WHQ040", "WHQ070", "WHQ150", "WHQ225")	c("BMXBMI", "BMXWAIST", "BMXWT", "DR1TP182", "DR1TRET", "DR1TVB12", "DR1TZINC", "LBDBSESI", "LBDFERSI", "LBDHDDSI", "LBDSCHSI", "LBDSTPSI", "LBDSTRSI", "LBDTIB", "LBXCOT", "LBXEOPCT", "LBXGH", "LBXHGB", "LBXPLTSI", "LBXSASSI", "LBXSCK", "LBXSGTSI", "RIDAGEYR", "URDACT", "URDTIME1", "URXUMA", "WHD050", "WHQ150")	c("BMXARMC", "BMXWAIST", "LBDSCHSI", "LBXGH", "LBXSASSI", "URDTIME1", "WHQ150")	c("BMXARMC", "BMXBMI", "BMXWAIST", "BMXWT", "DIQ010", "LBDFERSI", "LBDSCHSI", "LBDSGLSI", "LBDTCSI", "LBXFER", "LBXGH", "LBXSASSI", "LBXSATSI", "LBXSCH", "LBXSGL", "LBXSGTSI", "LBXTC", "URDACT", "WHD020", "WHD050", "WHD140", "WHQ150")	c("BMXBMI", "BMXWAIST", "BMXWT", "DR1TFA", "LBDBSESI", "LBDEONO", "LBDFERSI", "LBDSCHSI", "LBDSTPSI", "LBXBPB", "LBXEOPCT", "LBXGH", "LBXMOPCT", "LBXPLTSI", "LBXSASSI", "LBXSCH", "LBXSGTSI", "URDACT", "WHQ150")	c("BMXARMC", "BMXBMI", "BMXWAIST", "BMXWT", "DR1TFA", "LBDSGLSI", "LBXBSE", "LBXEOPCT", "LBXGH", "LBXPLTSI", "LBXSASSI", "LBXSATSI", "LBXSCH", "LBXSCK", "LBXSGL", "LBXSGTSI", "LBXSLDSI", "LBXSTR", "LBXSUA", "RIDAGEYR", "URDACT", "URXUMA", "URXUMS", "WHD020", "WHD050", "WHD140", "WHQ150")	c("BMXARMC", "BMXBMI", "BMXLEG", "BMXWAIST", "DBD895", "DBQ700", "DIQ160", "DPQ070", "DPQ080", "DR1HELP", "DR1LANG", "DR1MRESP", "DR1TATOA", "DR1TFDFE", "DR1TFF", "DR1TFIBE", "DR1TM181", "DR1TMAGN", "DR1TMFAT", "DR1TP182", "DR1TP183", "DR1TPOTA", "DR1TPROT", "DR1TS040", "DR1TS060", "DR1TS080", "DR1TS120", "DR1TS140", "DR1TS160", "DR1TS180", "DR1TSFAT", "DR1TZINC", "DRD340", "DS1DSCNT", "DSD010AN", "DSDANCNT", "FIAPROXY", "FSDAD", "FSDHH", "HEQ030", "HUQ090", "KIQ042", "LBDBGELC", "LBDBGESI", "LBDBSESI", "LBDCOTLC", "LBDHCOLC", "LBDSALSI", "LBDSBUSI", "LBDSCHSI", "LBDSGBSI", "LBDSIRSI", "LBDSTPSI", "LBDSTRSI", "LBDTCSI", "LBDTHGLC", "LBXBAPCT", "LBXBGE", "LBXBPB", "LBXHA", "LBXHCT", "LBXHGB", "LBXHSCRP", "LBXIRN", "LBXMC", "LBXMCVSI", "LBXPLTSI", "LBXRDW", "LBXSASSI", "LBXSCH", "LBXSCK", "LBXSCLSI", "LBXSCR", "LBXSGB", "LBXSGTSI", "LBXSIR", "LBXSOSSI", "LBXSTP", "LBXSTR", "LBXTC", "MCQ160M", "MCQ160P", "MCQ366B", "MIAPROXY", "OCD150", "OHAPOS", "OHQ835", "OHQ845", "OHX03TC", "OHX08TC", "OHX09TC", "OHX10TC", "OHX11TC", "OHX21TC", "OHX25TC", "OHX32TC", "OHXRCARO", "OHXRRES", "PHAFSTHR", "PHQ030", "PHQ040", "RIAGENDR", "RIDAGEYR", "SDMVPSU", "SLD013", "SLQ320", "SMQ856", "URXUMA", "URXUMS", "WHD020")
auc（评估模型）	0.831149442409864	0.855498109930204	0.796613169416269	0.783584984458608	0.842170828748879	0.842835928117601	0.867312684300067

importance（模型变量重要性的评分）	c(DBD895 = 380.666666666667, SIAINTRP = 370.333333333333, DBQ095Z = 369.666666666667, OHX25TC = 361.666666666667, LBDEONO = 360, MCQ371C = 357.333333333333, SIAPROXY = 357.333333333333, MCQ300A = 352.333333333333, DR1TVD = 349, LBDIRNSI = 347.333333333333, SMQ870 = 344, OHQ850 = 342.666666666667, OHXRRESO = 341.666666666667, MCQ300B = 340.333333333333, LBXSTR = 337.333333333333, OHX01TC = 336.333333333333, OHX18TC = 336, WHD010 = 331.333333333333, DS1DS = 331, OHXRRES = 331, DS1DSCNT = 327.666666666667, DR1TCHL = 327.333333333333, DR1TPROT = 325, MIALANG = 324.333333333333, URDUMALC = 323.666666666667, LBDSPHSI = 323, WHD050 = 318.666666666667, RIDEXMON = 318, DR1TVARA = 311, DR1TP182 = 310, PHQ040 = 308.333333333333, FSD151 = 307.333333333333, SMQ860 = 306.666666666667, DRQSDIET = 303.666666666667, HUQ071 = 303.666666666667, LBDBGMSI = 302.666666666667, DR1_330Z = 302, MIAPROXY = 301, MCQ220 = 300, DR1TFF = 299, OHX29TC = 299, LBXSGTSI = 298.666666666667, MCQ160B = 298, MCQ366B = 296.333333333333, LBXSUA = 294.666666666667, MCQ160A = 294, LBXMC = 292.666666666667, LBDBGMLC = 292, SMQ863 = 292, FSDHH = 291.666666666667, SLQ120 = 290.666666666667, OHX32TC = 290.333333333333, INDFMMPC = 290, LBDNENO = 289.333333333333, LBXRDW = 289.333333333333, LBDTCSI = 288.333333333333, PAQ665 = 287.666666666667, KIQ044 = 287.333333333333, IMQ020 = 286.666666666667, MCQ366D = 286.666666666667, LBXTHG = 286.333333333333, DMDBORN4 = 285.333333333333, DR1HELP = 285, LBXGH = 284.333333333333, DRDINT = 281.666666666667, OHX16TC = 280.333333333333, DR1EXMER = 280, RIDRETH1 = 280, DR1TP225 = 277, LBDFERSI = 276, SLQ040 = 275.333333333333, DR1TSELE = 275, LBXSCLSI = 273.666666666667, DBQ930 = 273.333333333333, WHD140 = 273, DBQ700 = 272.666666666667, SLQ330 = 272.333333333333, DR1TACAR = 272, DR1TKCAL = 272, LBXCOT = 271.333333333333, DRD340 = 270, URXVOL1 = 269, URXCRS = 267.333333333333, LBXHBS = 266.666666666667, IMQ011 = 264.666666666667, DPQ050 = 263.666666666667, LBXIHG = 263.666666666667, OHQ845 = 263.666666666667, PHQ050 = 263.666666666667, DMDMARTZ = 263, LBDHDDSI = 262.666666666667, DR1TVB1 = 261.333333333333, OHX06TC = 259, SMQ681 = 259, LBDPCT = 258, LBXBGM = 257.666666666667, HIQ270 = 257.333333333333, LBXTC = 255.666666666667, SLQ030 = 255.333333333333, LBDSGBSI = 254.666666666667, SLQ050 = 254.666666666667, LBDBGELC = 254, DR1TSUGR = 253.333333333333, LBXPLTSI = 253.333333333333, MCQ080 = 253.333333333333, OHX22TC = 253, DR1TP226 = 252.333333333333, OHQ640 = 252.333333333333, LBDHEM = 251.333333333333, PHAFSTHR = 251.333333333333, OHQ620 = 251, DR1TNIAC = 250.666666666667, LBDTIB = 250.333333333333, LBDHDD = 250, DR1TSFAT = 247, OHXRCARO = 246.333333333333, DR1TS180 = 245.666666666667, DIQ160 = 245.333333333333, DR1TLYCO = 245, HUQ010 = 244.333333333333, DR1TM161 = 243.666666666667, DR1TS120 = 243.666666666667, DR1TRET = 243.333333333333, LBDSCRSI = 242.666666666667, MCQ371B = 242.666666666667, OHX08TC = 242.666666666667, DR1TCOPP = 242.333333333333, LBXHCT = 242.333333333333, MCQ160M = 242.333333333333, BMXHT = 241.666666666667, LBXSKSI = 241.666666666667, BMXBMI = 240.666666666667, LBXSCH = 240.333333333333, LBDBGESI = 239.333333333333, MCQ010 = 239, WHQ225 = 237.333333333333, MCQ300C = 237, OHX27TC = 237, PHQ020 = 237, DMDEDUC2 = 236, DIQ010 = 235.666666666667, DR1TM181 = 235.666666666667, DR1TS080 = 235.666666666667, OHX04TC = 235, DSDCOUNT = 233.666666666667, LBDCOTLC = 233, LBXBSE = 233, LBDSUASI = 232.666666666667, DR1TFOLA = 232.333333333333, MCQ371A = 232.333333333333, KIQ046 = 232, DR1DAY = 231.666666666667, DR1TCHOL = 231, LBDSCASI = 231, MCQ560 = 231, WHQ150 = 230.333333333333, MCQ160L = 229.666666666667, PHQ030 = 229.666666666667, BMXARML = 228.666666666667, OHX05TC = 228.666666666667, SMD460 = 228.666666666667, OHQ030 = 228, DR1TZINC = 227.666666666667, DR1TS160 = 227.333333333333, LBXHCR = 227.333333333333, MCQ160D = 227, HIQ011 = 226.666666666667, DPQ010 = 226, DPQ020 = 226, LBXSGB = 226, PAQ620 = 225, BMXARMC = 224, DR1TM201 = 224, LBDSTBSI = 224, WHQ040 = 224, PAQ635 = 223.666666666667, SMQ851 = 223.666666666667,DRD360 = 222.666666666667, LBXBPB = 222.666666666667, SLD012 = 222.666666666667, DR1 300 = 221.666666666667,LBDHCI = 221.666666666667, LBXHBC = 221, LBXSPH = 220.333333333333, MCQ092 = 220.333333333333, DBQ229 = 220, DSDANCNT = 219.666666666667, OHXRCAR = 218.666666666667, PAQ605 = 218, DBQ945 = 217.666666666667, OHX19TC = 217.333333333333, DR1TVB2 = 217, DR1TP183 = 216.666666666667, LBDSGTLC = 214.666666666667, DR1TVC = 212.666666666667, SLD013 = 212.666666666667, DR1TBCAR = 212.333333333333, HUQ030 = 211.666666666667, KIQ005 = 211.333333333333, LBDBPBSI = 211.333333333333, URXUCR = 210.666666666667, LBXSTP = 209.666666666667, KIQ480 = 209.333333333333, LBDSTPSI = 209.333333333333, DR1TMFAT = 209, LBXSLDSI = 209, OHX28TC = 209, LBDBCDSI = 207.666666666667, LBDBSESI = 207.666666666667, OHDRCSTS = 207.333333333333, RIAGENDR = 207.333333333333, URDTIME1 = 207, DBQ935 = 206.666666666667, LBDSTRSI = 206.333333333333, LBXSOSSI = 206.333333333333, WHD120 =	c(BMXWAIST = 28, LBXSASSI = 27, LBXGH = 25.6666666666667, LBXSGTSI = 24.6666666666667, URDTIME1 = 18.6666666666667, WHD050 = 18, LBXEOPCT = 16, LBXPLTSI = 16, LBDSCHSI = 15.6666666666667, URXUMA = 15.3333333333333, BMXWT = 15, LBDFERSI = 13.6666666666667, BMXBMI = 13.3333333333333, DR1TVB12 = 13.3333333333333, WHQ150 = 13.3333333333333, DR1TRET = 13, LBDTIB = 13, DR1TP182 = 11.6666666666667, LBXCOT = 10.6666666666667, LBDBSESI = 10.3333333333333, LBXSCK = 10.3333333333333, DR1TZINC = 10, RIDAGEYR = 10, URDACT = 10, LBXHGB = 9, LBDHDDSI = 8.33333333333333, LBDSTPSI = 8, LBDSTRSI = 8)	c(BMXWAIST = 7, LBXSASSI = 6, LBXGH = 5, BMXARMC = 3, URDTIME1 = 2.66666666666667, LBDSCHSI = 2.33333333333333, WHQ150 = 2)	c(BMXWAIST = 22, BMXBMI = 20.6666666666667, BMXWT = 20.3333333333333, WHD020 = 19, BMXARMC = 18, LBXSASSI = 16.3333333333333, LBXSGTSI = 15, LBXSATSI = 13.6666666666667, WHD050 = 11.3333333333333, LBDFERSI = 10.3333333333333, LBXFER = 10, LBXGH = 9.66666666666667, LBDSCHSI = 8, WHD140 = 7.66666666666667, LBDTCSI = 7, LBXSCH = 7, DIQ010 = 6.66666666666667, LBXSGL = 6.66666666666667, LBXTC = 6.66666666666667, LBDSGLSI = 6.33333333333333, WHQ150 = 5.66666666666667, URDACT = 5)	c(BMXWAIST = 19, LBXSASSI = 17.4, LBXGH = 17, BMXBMI = 15.8, LBXSGTSI = 15.2, WHQ150 = 14, URDACT = 12, LBXMOPCT = 11.8, LBXPLTSI = 9, LBDBSESI = 8.6, LBXEOPCT = 8, LBDSTPSI = 7.8, LBDSCHSI = 6.6, LBXBPB = 6.6, LBDFERSI = 5.4, BMXWT = 5.2, LBDEONO = 4, LBXSCH = 3.4, DR1TFA = 3.2)	c(BMXWAIST = 27, LBXSASSI = 25, BMXBMI = 24.8, BMXWT = 24.2, LBXSGTSI = 24, WHD020 = 21.6, LBXGH = 21.2, WHD050 = 19.6, BMXARMC = 18.4, LBXSATSI = 16.4, WHD140 = 16.4, RIDAGEYR = 15.8, URDACT = 13.8, LBXSCH = 13.2, WHQ150 = 12.4, LBXSGL = 11, LBXPLTSI = 10.4, LBDSGLSI = 10.2, URXUMS = 9.4, URXUMA = 9, LBXEOPCT = 8.2, DR1TFA = 6.2, LBXBSE = 5, LBXSCK = 4.4, LBXSTR = 3.8, LBXSLDSI = 3.6, LBXSUA = 3)	c(WHD020 = 108.6, DR1TS160 = 106.4, LBDSTRSI = 104.2, BMXWAIST = 104, DR1TMFAT = 103.2, DR1TM181 = 102.6, DR1TS140 = 101.8, LBXSASSI = 100.2, FSDAD = 98.4, OHX09TC = 97.4, LBDSTPSI = 96.2, FSDHH = 94.8, MIAPROXY = 94.4, DR1TSFAT = 92.8, LBDBGELC = 90.8, BMXLEG = 88, DR1HELP = 87.8, DR1TPOTA = 85.2, LBXSTR = 85.2, RIDAGEYR = 82.8, DR1TMAGN = 78.4, LBDHCOLC = 76.2, LBXSCK = 73.2, DR1TP182 = 73, DR1LANG = 72.4, DR1TS060 = 72.2, DIQ160 = 71.2, DR1TS180 = 69.8, OHX08TC = 68.8, LBDSIRSI = 68.4, DR1TS040 = 68.2, OHX11TC = 67.6, LBXTC = 66, LBXSIR = 65.2, DR1TS080 = 63, LBXMC = 63, DPQ070 = 62.8, DSD010AN = 61.8, OHX10TC = 61.8, LBXBAPCT = 60.4, LBDTHGLC = 60, LBDSCHSI = 59.8, LBXIRN = 58.4, LBDSBUSI = 58.2, LBXSCH = 58.2, DBD895 = 58, BMXARMC = 57.8, OCD150 = 57.6, URXUMS = 57.6, DSDANCNT = 57.2, DR1TP183 = 56.6, LBDCOTLC = 56.6, LBXPLTSI = 56.4, OHXRRES = 53.4, DR1MRESP = 53.2, RIAGENDR = 53.2, LBDTCSI = 53, HEQ030 = 52.6, MCQ160M = 52.2, DR1TPROT = 50.6, LBXHGB = 50.4, DR1TFDFE = 50, OHX03TC = 50, LBXBPB = 49.4, FIAPROXY = 49, LBXMCVSI = 48.8, LBDSALSI = 48.6, HUQ090 = 44.4, OHAPOS = 43.8, OHXRCARO = 42.4, SDMVPSU = 41.4, LBXRDW = 41, LBXSGB = 39.8, DPQ080 = 38.8, LBDSGBSI = 38.8, LBDBSESI = 38.2, DR1TS120 = 38, LBXSTP = 38, KIQ042 = 37.4, LBXSGTSI = 37.2, PHAFSTHR = 37, LBXHSCRP = 36.8, OHX21TC = 36.8, OHX32TC = 35.4, URXUMA = 35.4, SMQ856 = 35, MCQ160P = 34.4, OHQ835 = 34.2, DR1TZINC = 33.2, LBXHA = 32.2, SLD013 = 31.8, OHQ845 = 31.4, BMXBMI = 28.2, DR1TFF = 28.2, DR1TATOA = 27.2, MCQ366B = 27.2, LBXSCLSI = 26.6, DR1TFIBE = 26, LBXSOSSI = 25.8, DBQ700 = 25.6, PHQ040 = 24.2, PHQ030 = 23.6, LBXHCT = 23, OHX25TC = 23,LBXBGE = 22.2, DRD340 = 22, LBDBGESI = 21, SLQ320 = 16.6, DS1DSCNT = 13.8, LBXSCR = 13)

	206.333333333333, BMXWT = 206, DR1TB12A = 205.666666666667, DR1TM221 = 205.333333333333, OHX15TC = 205.333333333333, LBXEOPCT = 205, LBXIRN = 205, SLQ300 = 205, LBXNEPCT = 204.333333333333, DR1TMOIS = 204, PAQ650 = 204, MCQ366A = 202.666666666667, OHX02TC = 202.333333333333, OHX26TC = 202, OHX17TC = 200.333333333333, OHX14TC = 200, HUQ090 = 199.666666666667, SDMVSTRA = 199.666666666667, DR1TCARB = 199.333333333333, LBXBAPCT = 199.333333333333, FIAINTRP = 198.666666666667, SDMVPSU = 198.666666666667, OHQ835 = 197.333333333333, SMQ020 = 195.333333333333, DR1TPOTA = 195, OHX30TC = 195, BMDSTATS = 194.333333333333, LBXSCA = 194.333333333333, LBXMCVSI = 192.666666666667, LBXSNASI = 192.666666666667, DR1LANG = 192.333333333333, LBXSTB = 192.333333333333, LBDSCHSI = 192, LBXBCD = 191.666666666667, DR1TP204 = 190.333333333333, BMXLEG = 189.333333333333, LBDIHGLC = 189, OHX13TC = 189, LBDBCDLC = 188.333333333333, LBDSALSI = 188.333333333333, WHQ030 = 188.333333333333, DR1TLZ = 188, LBDHCOLC = 188, DR1TFIBE = 187.666666666667, ALQ111 = 187, KIQ022 = 187, DPQ080 = 186.333333333333, LBXSAPSI = 186.333333333333, BPQ080 = 185.333333333333, OHX12TC = 185, DR1TVB6 = 184.666666666667, DR1TATOC = 184.333333333333, LBXSC3SI = 183.666666666667, LBXHA = 183.333333333333, OHQ033 = 182.666666666667, DBQ940 = 182.333333333333, LBXSBU = 182.333333333333, HUQ051 = 182, OHAPOS = 182, URXUMA = 181.666666666667, LBXMPSI = 181.333333333333, DR1STY = 181, DR1TIRON = 180.666666666667, FSQ165 = 179.666666666667, DR1TMAGN = 179.333333333333, AUQ054 = 179, DR1MRESP = 178.666666666667, PHQ060 = 178.333333333333, LBDBMNSI = 177.666666666667, DPQ090 = 177.333333333333, MCQ160F = 177.333333333333, OHX09TC = 177.333333333333, LBXSATSI = 177, LBXBGE = 176.666666666667, DR1TCRYP = 176.333333333333, KIQ026 = 176, LBXSAL = 174.666666666667, LBXBMN = 174, PHAFSTMN = 172.666666666667, FIAPROXY = 172, OHX07TC = 172, DPQ030 = 171.333333333333, LBDSBUSI = 171, DR1TATOA = 170.666666666667, DR1TP205 = 170.666666666667, MCQ160E = 170.333333333333, INDFMPIR = 170, SLQ320 = 170, DBQ197 = 169.666666666667, MCQ160P = 169.333333333333, DR1TWSZ = 169, DR1TFA = 168.666666666667, FIALANG = 168.666666666667, URXUMS = 168.666666666667, PUQ110 = 168, DR1TP184 = 167.666666666667, DR1TVB12 = 167, HIQ210 = 166.333333333333, LBXMCHSI = 166.333333333333, OHDEXSTS = 164.333333333333, LBXWBCSI = 163, URDFLOW1 = 161.666666666667, LBXHSCRP = 161.333333333333, LBDHEG = 160.666666666667, LBXRBCSI = 160, OHX03TC = 160, OHX24TC = 160, LBDTHGLC = 159.666666666667, BMXWAIST = 159, DBD910 = 158.333333333333, DR1TTHEO = 158.333333333333, HEQ030 = 158.333333333333, OHX10TC = 158, DR1TNUMF = 157, DR1TTFAT = 156.666666666667, OHQ860 = 156.666666666667, DIQ180 = 156.333333333333, DSD010 = 156.333333333333, DBD905 = 156, RIDAGEYR = 156, LBDMONO = 155.666666666667, LBXSCK = 155.666666666667, WHD020 = 155.666666666667, OHX20TC = 155, SMQ872 = 153, LBXLYPCT = 152.333333333333, DR1TCALC = 152, LBDLYMNO = 151.333333333333, LBXMOPCT = 151, OHXIMP = 148.333333333333, DRQSPREP = 147.666666666667, DR1TS140 = 147.333333333333, FSD162 = 146.333333333333, LBXFER = 146, SLQ310 = 145.666666666667, SMDANY = 145.666666666667, LBDSGLSI = 145.333333333333, SMQ856 = 145, URDACT = 145, SMQ874 = 144.666666666667, DR1TSODI = 144.333333333333, MCQ053 = 143.666666666667, ALQ121 = 143.333333333333, DR1TPHOS = 143.333333333333, DR1BWATZ = 143, DR1 320Z = 142.666666666667, DR1TS040 = 142, ALQ151 = 140, DPQ060 = 138, LBDHRPLC = 137.666666666667, LBXSCR = 135.666666666667, SMQ866 = 135.666666666667, DR1DBIH = 135, SIALANG = 131.666666666667, OHX23TC = 130.333333333333, DR1TPFAT = 130, OHX31TC = 129.666666666667, CBQ596 = 128.333333333333, MCQ550 = 128.333333333333, DR2DRSTZ = 128, DR1TS100 = 127.333333333333, LBDSTBLC = 126.666666666667, LBDTIBSI = 124, DR1TALCO = 123.666666666667, DPQ070 = 123.333333333333, RIDRETH3 = 121, LBXHGB = 119.333333333333, MCQ366C = 119.333333333333, WHQ070 = 119.333333333333, LBXSASSI = 117, LBDBANO = 116, OHX21TC = 115.666666666667, LBXSIR = 115.333333333333, OHX11TC = 112.333333333333, OHQ770 = 111.666666666667, LBDSIRSI = 111, DPQ040 = 110.666666666667, MCQ520 = 109.333333333333, LBXSGL = 108.333333333333, LBDIHGSI = 103.666666666667, PUQ100 = 101, LBDTHGSI = 99, OHQ870 = 98.3333333333333, HSQ590 = 97.6666666666667, DR1TFDFE = 94.3333333333333, MIAINTRP = 93.3333333333333, FSDAD = 93, HEQ010 = 92.6666666666667, PAD680 = 92.6666666666667, PHDSESN = 91, DSD010AN = 90.6666666666667, DS1ANCNT = 84.3333333333333, MCQ160C = 82, SMQ878 = 81.6666666666667, OHDDESTS = 81, DR1TS060 = 80, DR1TVK = 79, LBXNRBC = 79, OHAREC = 78.3333333333333, KIQ042 = 72.6666666666667, OCD150 = 65.6666666666667, DS1AN = 52, DR1TCAFF = 39.6666666666667, MCQ371D = 36.3333333333333)						
