# Supplementary material for: Integrative and interpretable machine learning framework for early non-invasive detection of clinically significant liver fibrosis
Source: Front Med (Lausanne). 2026 Jun 23;13:1736295. doi: 10.3389/fmed.2026.1736295 (PMC13337473; doi:10.3389/fmed.2026.1736295)
Supplement: Supplementary file 12 — The figure shows that during the benchmark model selection process, we used the Precision-Recall curve (PR curve) and the Receiver Operating Characteristic curve (ROC curve) to compare the clinically significant liver fibrosis classification performance of 29 machine learning classification models. All models were constructed based on the eight key variables identified through feature selection in the study and internally evaluated via five-fold cross-validation, aiming to intuitively demonstrate the classification and discriminative predictive ability of different models for clinically significant liver fibrosis patients in the population at the benchmark level. [file Data_Sheet_12.pdf]

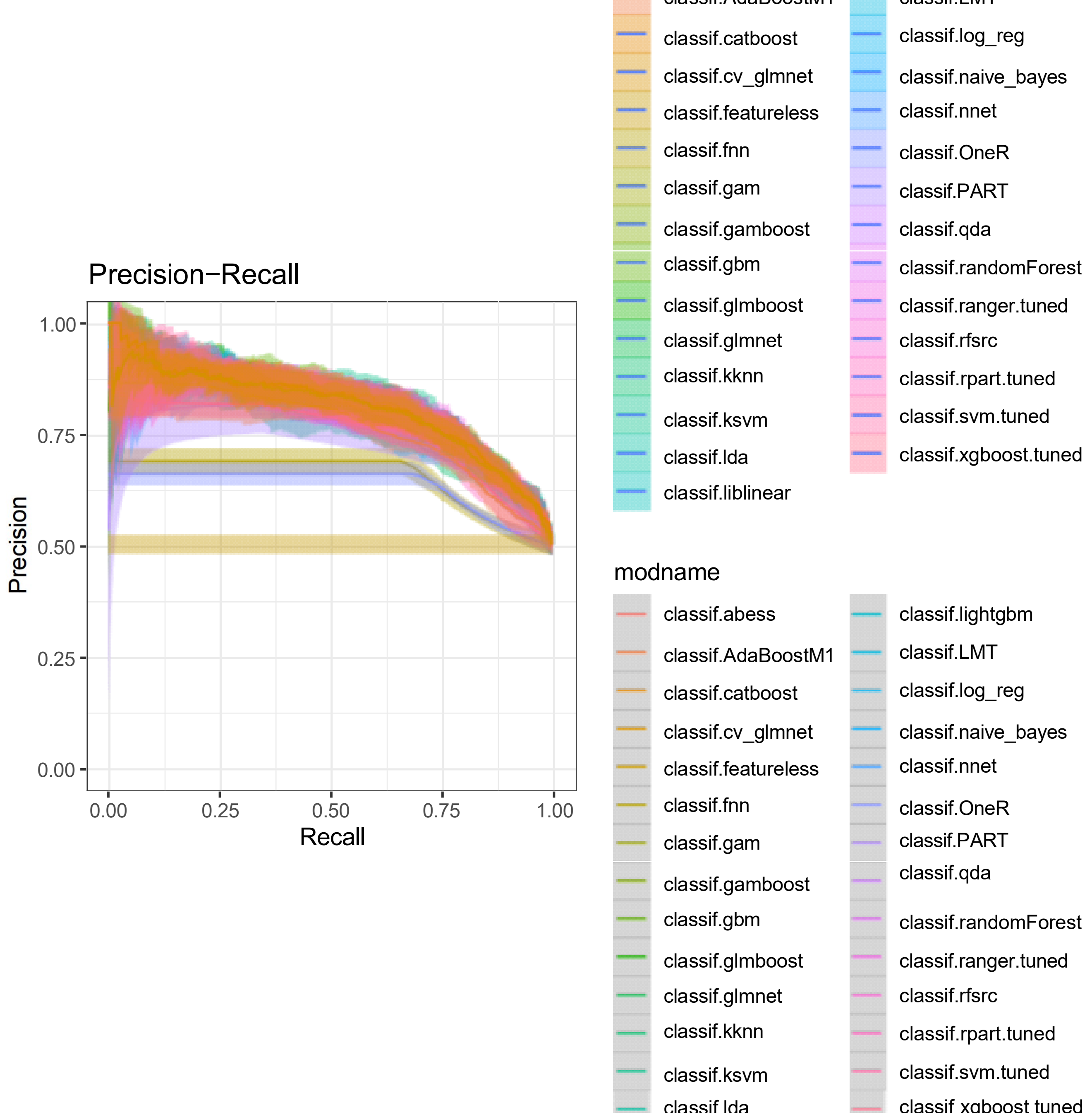
Precision−Recall
Precision
Recall
1.00
0.75
0.50
0.25
0.00
0.00
0.25
0.50
0.75
1.00
classif.catboost
classif.cv_glmnet
classif.featureless
classif.fnn
classif.gam
classif.gamboost
classif.gbm
classif.glmboost
classif.glmnet
classif.kknn
classif.ksvm
classif.lda
classif.liblinear
classif.log_reg
classif.naive_bayes
classif.nnet
classif.OneR
classif.PART
classif.qda
classif.randomForest
classif.ranger.tuned
classif.rfsrc
classif.rpart.tuned
classif.svm.tuned
classif.xgboost.tuned
modname
classif.abess
classif.AdaBoostM1
classif.catboost
classif.cv_glmnet
classif.featureless
classif.fnn
classif.gam
classif.gamboost
classif.gbm
classif.glmboost
classif.glmnet
classif.kknn
classif.ksvm
classif.lda
classif.lightgbm
classif.LMT
classif.log_reg
classif.naive_bayes
classif.nnet
classif.OneR
classif.PART
classif.qda
classif.randomForest
classif.ranger.tuned
classif.rfsrc
classif.rpart.tuned
classif.svm.tuned
classif.xgboost.tuned

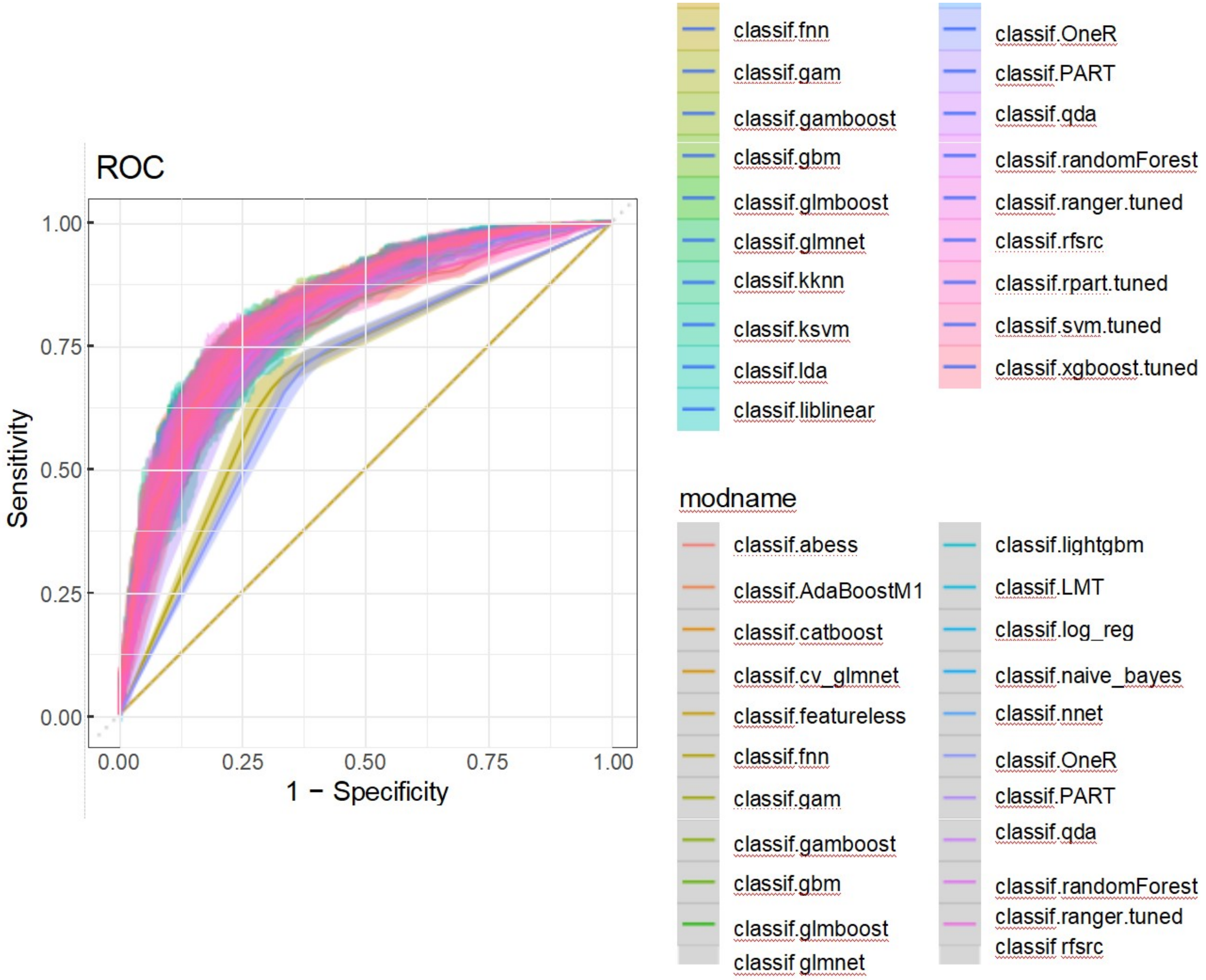
ROC
Sensitivity
1.00
0.75
0.50
0.25
0.00
0.00
0.25
0.50
0.75
1.00
1 − Specificity
classif.fnn
classif.gam
classif.gamboost
classif.gbm
classif.glmboost
classif.glmnet
classif.kknn
classif.ksvm
classif.lda
classif.liblinear
classif.OneR
classif.PART
classif.qda
classif.randomForest
classif.ranger.tuned
classif.rfsrc
classif.rpart.tuned
classif.svm.tuned
classif.xgboost.tuned
modname
classif.abess
classif.AdaBoostM1
classif.catboost
classif.cv_glmnet
classif.featureless
classif.fnn
classif.gam
classif.gamboost
classif.gbm
classif.glmboost
classif glmnet
classif.lightgbm
classif.LMT
classif.log_reg
classif.naive_bayes
classif.nnet
classif.OneR
classif.PART
classif.qda
classif.randomForest
classif.ranger.tuned
classif rfsrc
