# Supplementary material for: Integrative and interpretable machine learning framework for early non-invasive detection of clinically significant liver fibrosis
Source: Front Med (Lausanne). 2026 Jun 23;13:1736295. doi: 10.3389/fmed.2026.1736295 (PMC13337473; doi:10.3389/fmed.2026.1736295)
Supplement: Supplementary file 13 — Calibration plots of the Gamboost model across internal and external cohorts. (A) Calibration curve in the internal testing set, demonstrating initial model fit (Brier score = 0.1483; Hosmer-Lemeshow [H-L] test P = 0.0309). (B) Calibration curve in the external validation cohort prior to prevalence adjustment (Brier score = 0.1579; H-L test P < 0.001). (C) Recalibrated curve for the external validation cohort following Bayesian prior-correction. The mathematically adjusted model exhibited excellent agreement between predicted probabilities and actual observed frequencies of clinically significant liver fibrosis, evidenced by a substantially minimized Brier score (0.0727) and a non-significant H-L goodness-of-fit test (P = 0.6829). [file Data_Sheet_13.pdf]

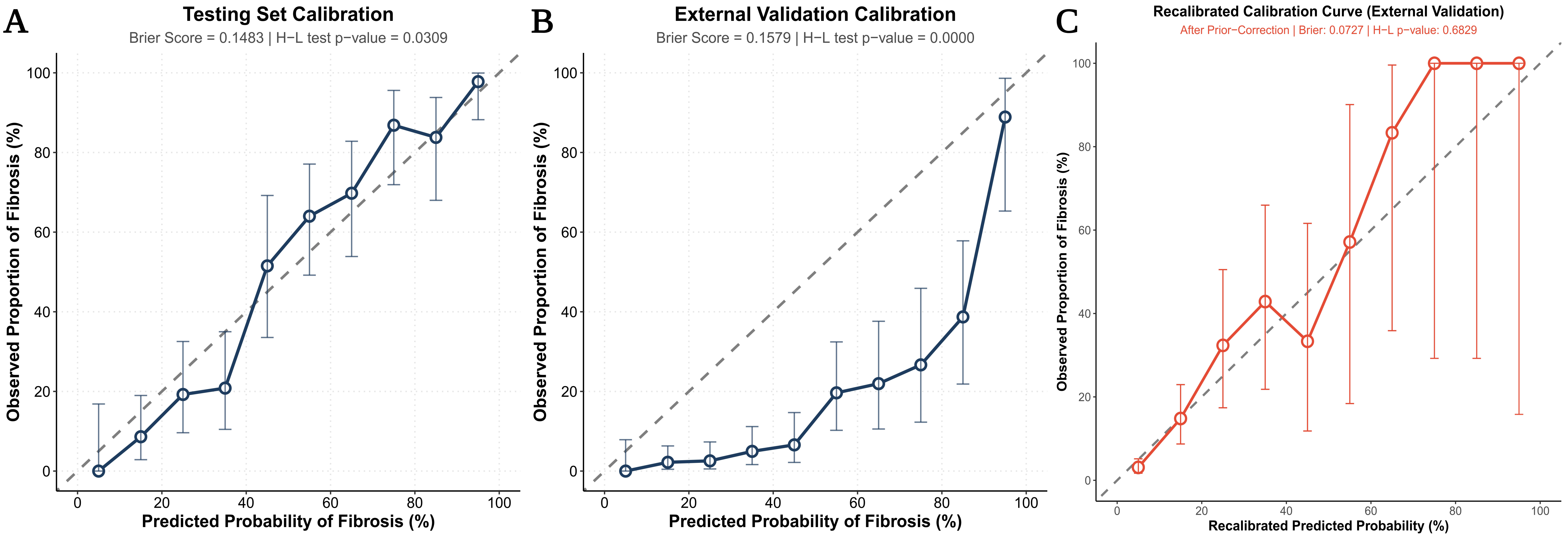
A
Testing Set Calibration
Brier Score = 0.1483 | H−L test p−value = 0.0309
Observed Proportion of Fibrosis (%)
Predicted Probability of Fibrosis (%)
B
External Validation Calibration
Brier Score = 0.1579 | H−L test p−value = 0.0000
Observed Proportion of Fibrosis (%)
Predicted Probability of Fibrosis (%)
C
Recalibrated Calibration Curve (External Validation)
After Prior−Correction | Brier: 0.0727 | H−L p−value: 0.6829
Observed Proportion of Fibrosis (%)
Recalibrated Predicted Probability (%)
0
20
40
60
80
100
